# Supplementary figures and images for: Venomix: a simple bioinformatic pipeline for identifying and characterizing toxin gene candidates from transcriptomic data
Source: PeerJ. 2018 Jul 31;6:e5361. doi: 10.7717/peerj.5361 (PMC6074769; doi:10.7717/peerj.5361)

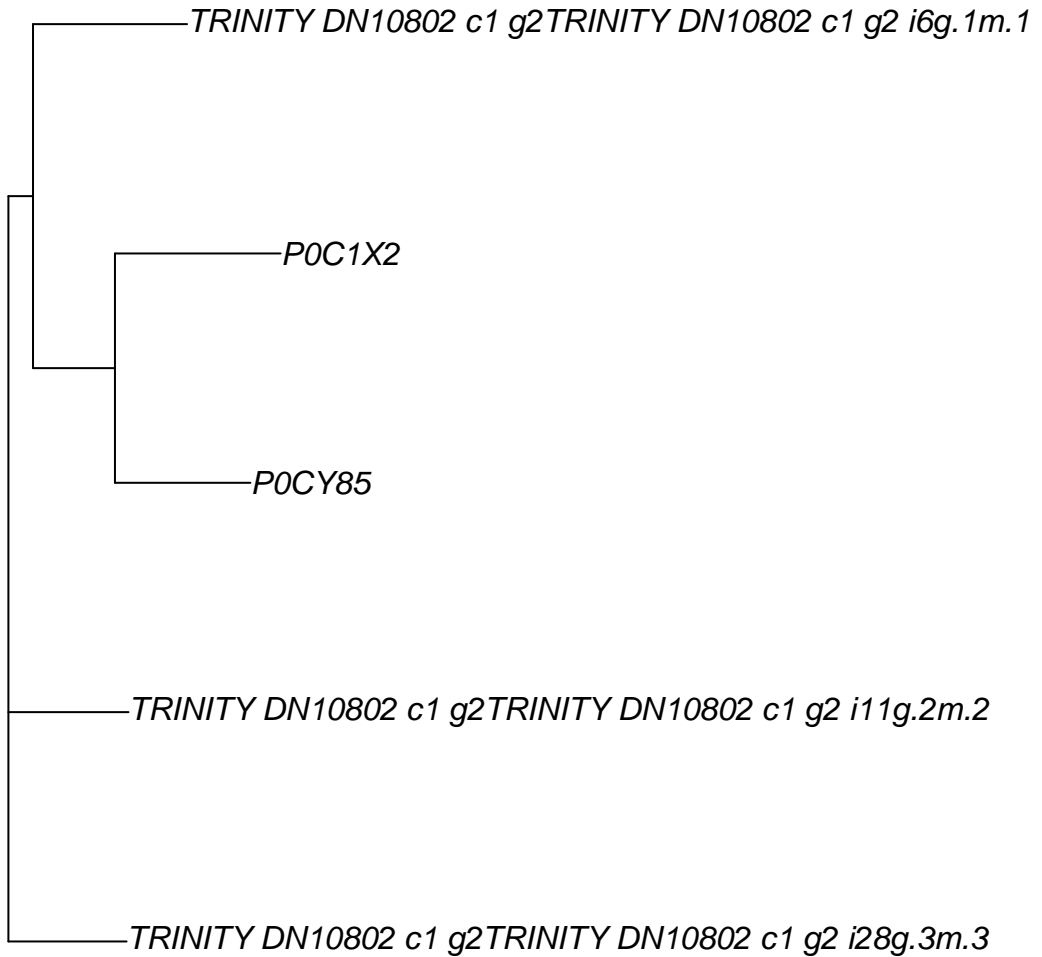

Supplement: Supplemental Information 2 [file peerj-06-5361-s002.gz › FinalOutput_1E-20/Kunitz-type_conkunitzin-S1_2/finaltree.pdf]

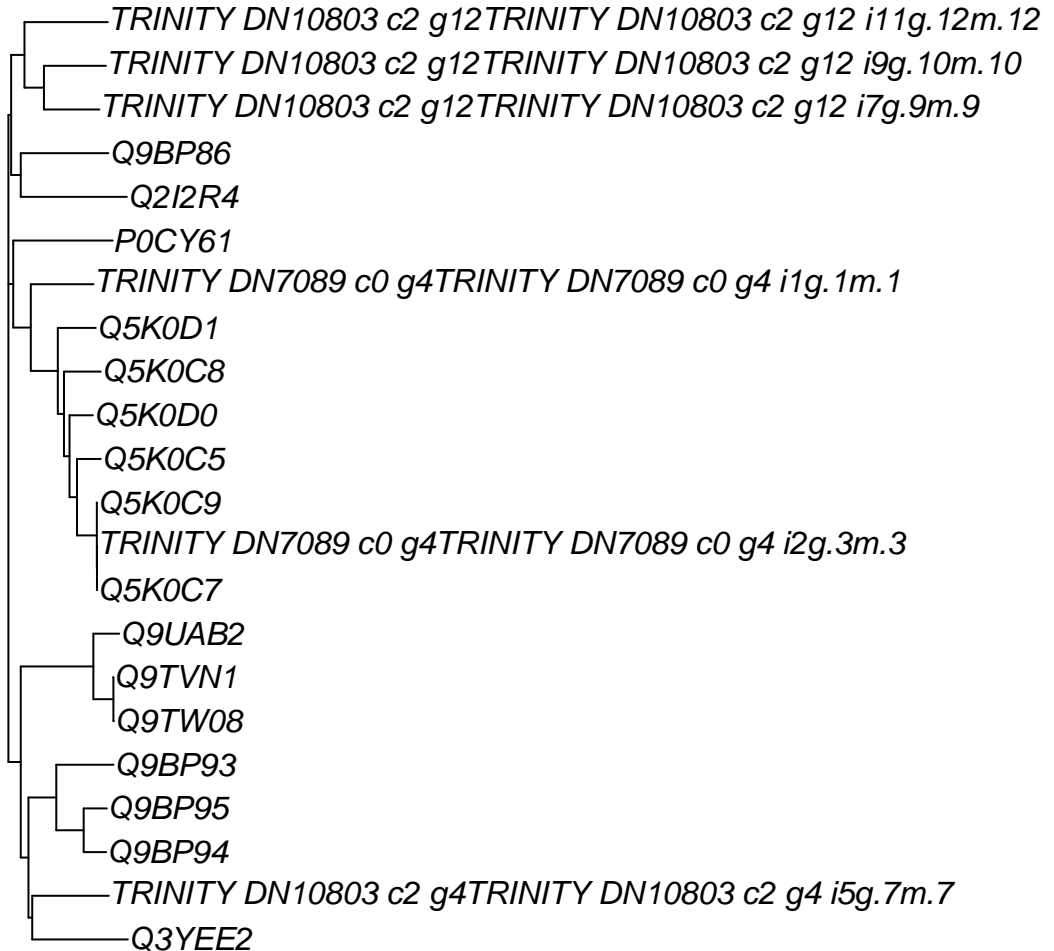

Supplement: Supplemental Information 2 [file peerj-06-5361-s002.gz › FinalOutput_1E-20/Conotoxin_MiEr92_6/finaltree.pdf]

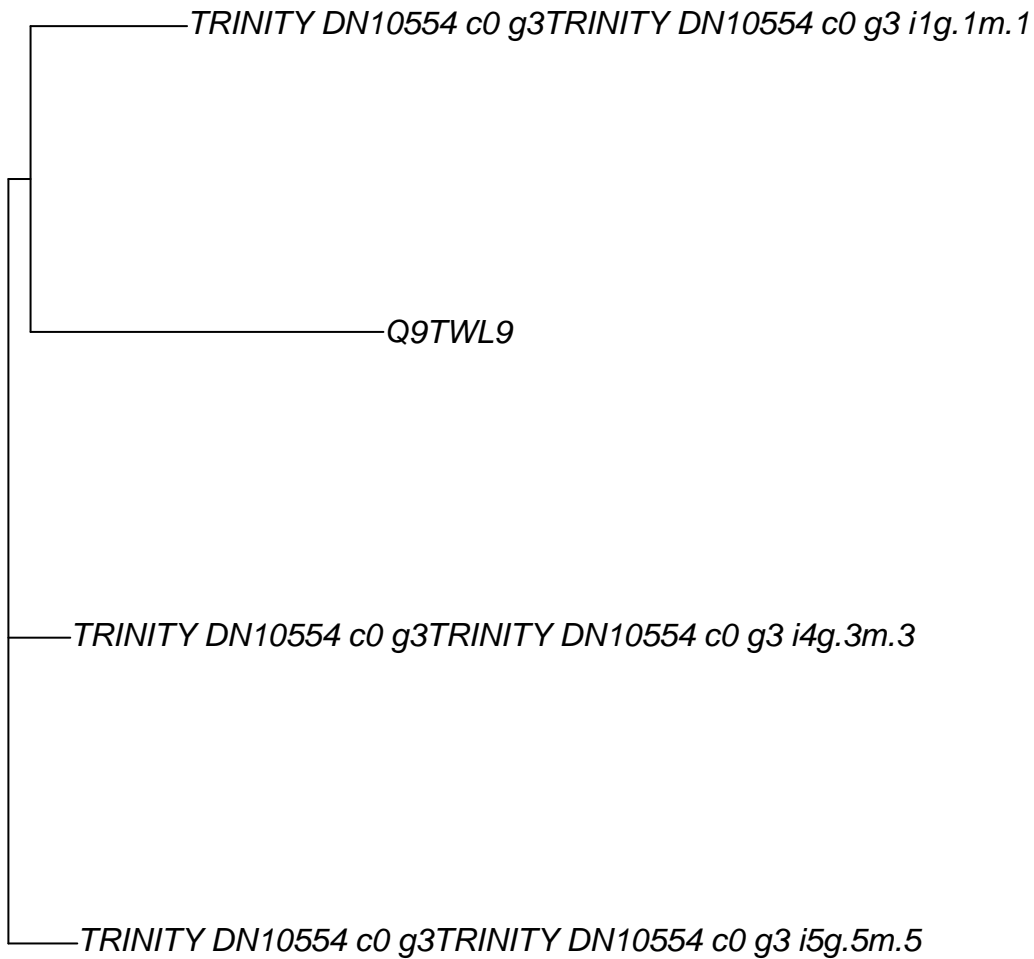

Supplement: Supplemental Information 2 [file peerj-06-5361-s002.gz › FinalOutput_1E-20/Conodipine-M_alpha_chain_1/finaltree.pdf]

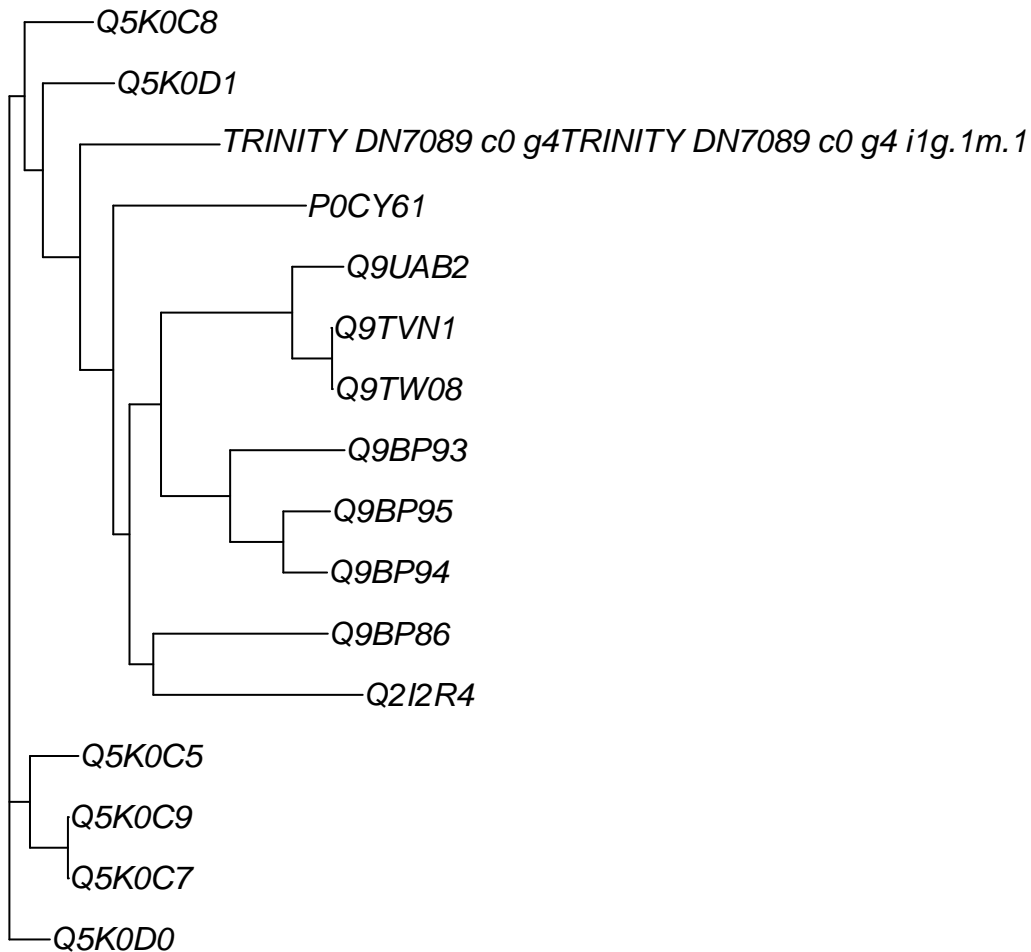

Supplement: Supplemental Information 2 [file peerj-06-5361-s002.gz › FinalOutput_1E-20/Conotoxin_ArMKLT2-0313_21/finaltree.pdf]

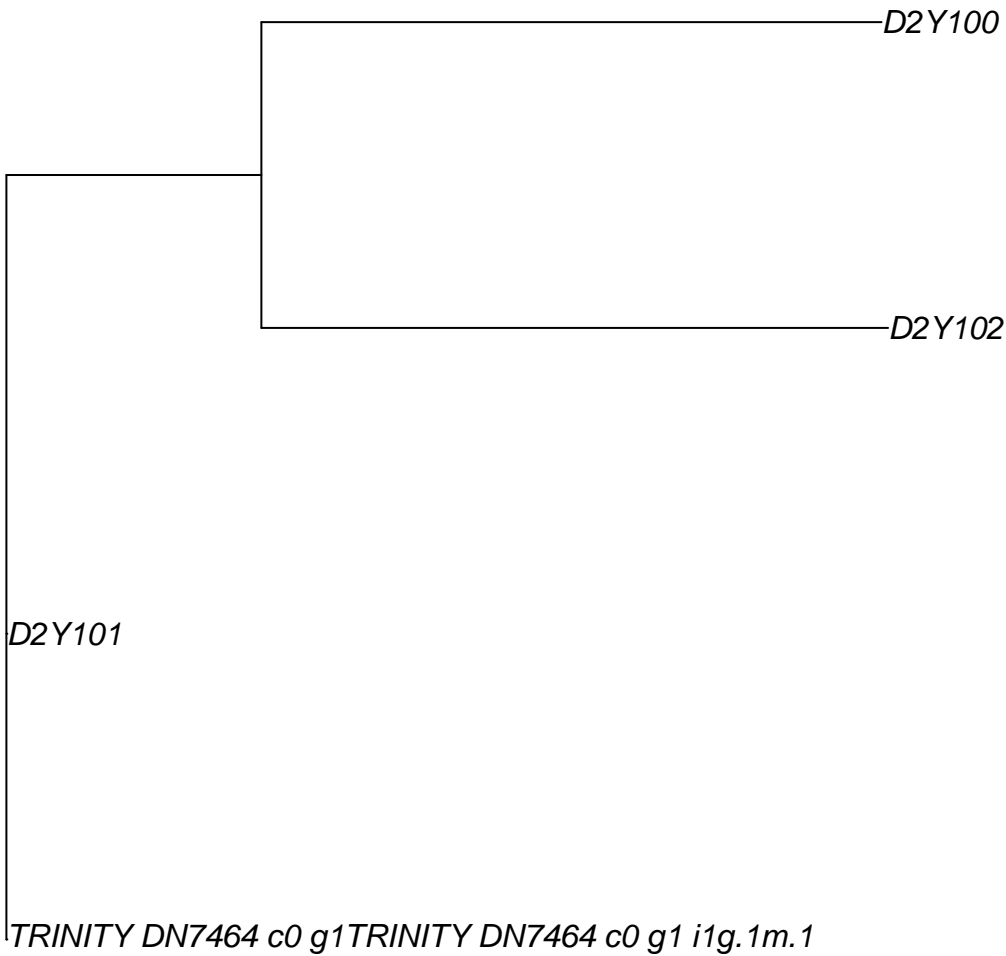

Supplement: Supplemental Information 2 [file peerj-06-5361-s002.gz › FinalOutput_1E-20/Conotoxin_Cal14/finaltree.pdf]

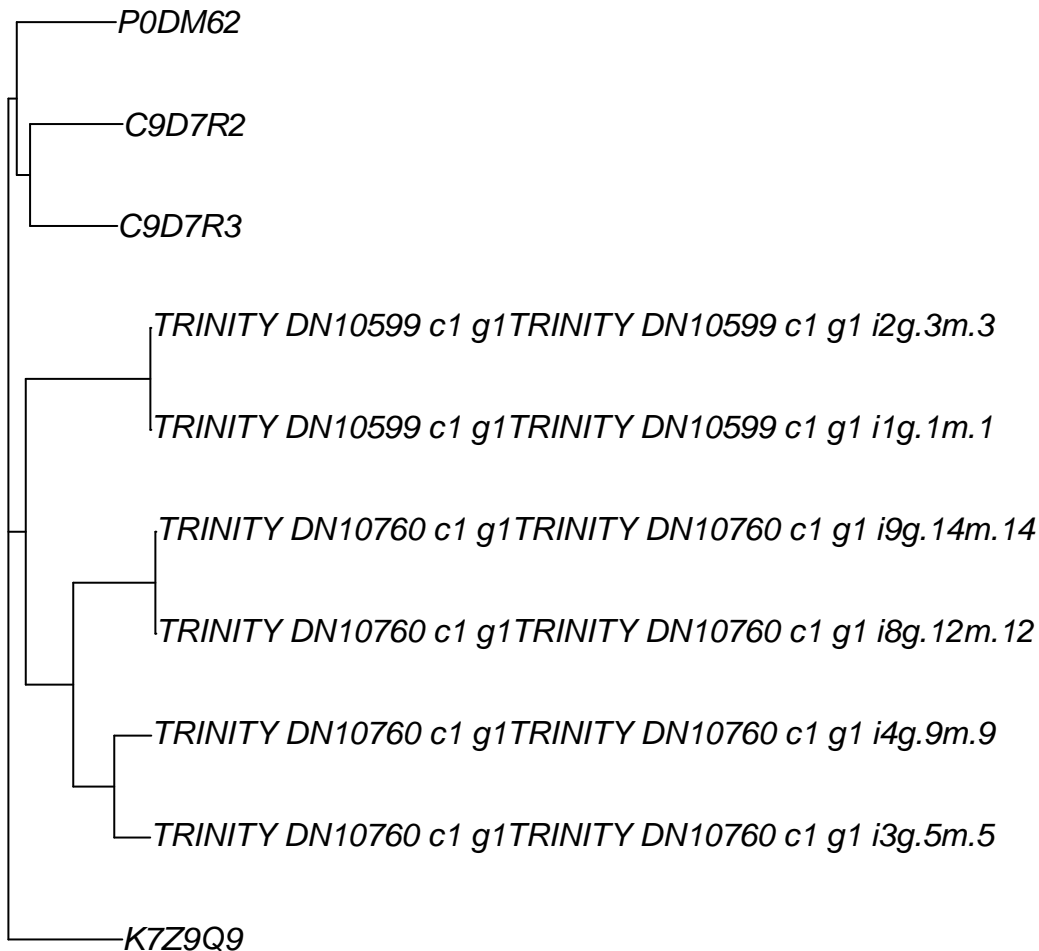

Supplement: Supplemental Information 2 [file peerj-06-5361-s002.gz › FinalOutput_1E-20/Nematocyte_expressed_protein_6_1/finaltree.pdf]

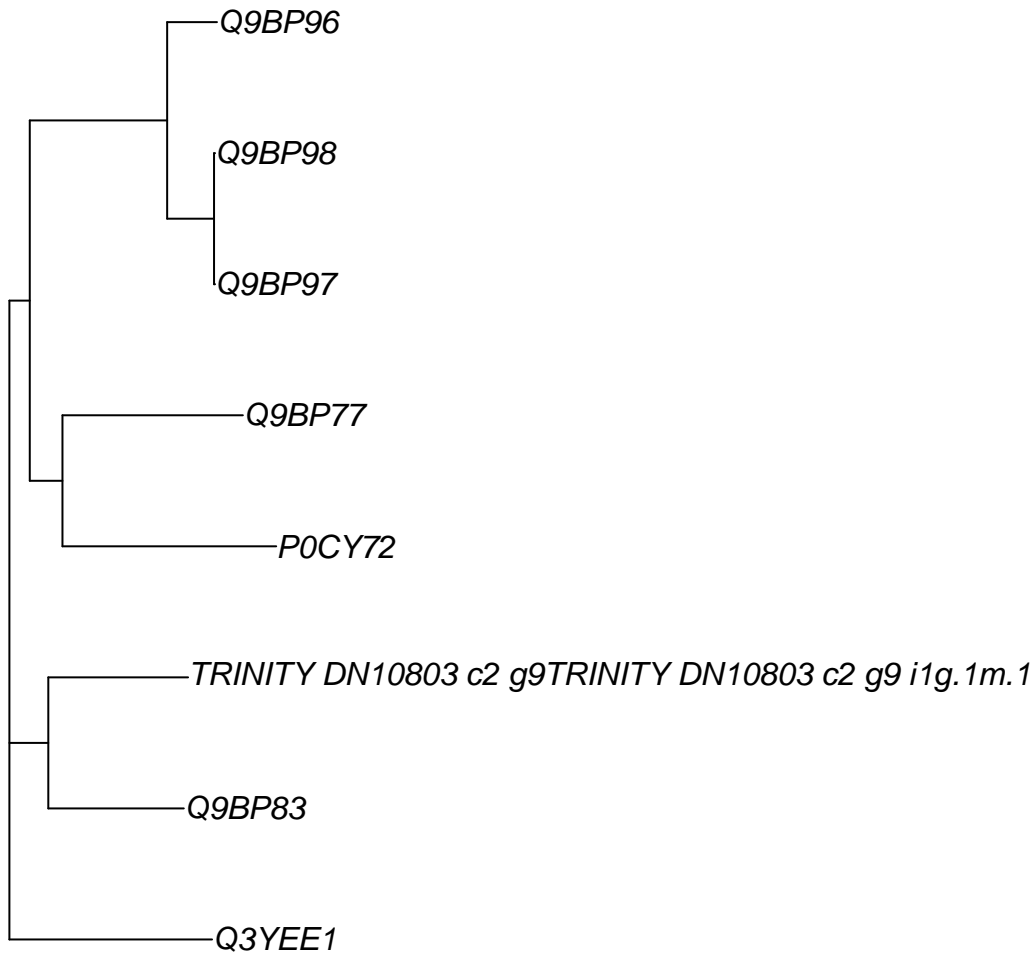

Supplement: Supplemental Information 2 [file peerj-06-5361-s002.gz › FinalOutput_1E-20/Conotoxin_MaIr94_14/finaltree.pdf]

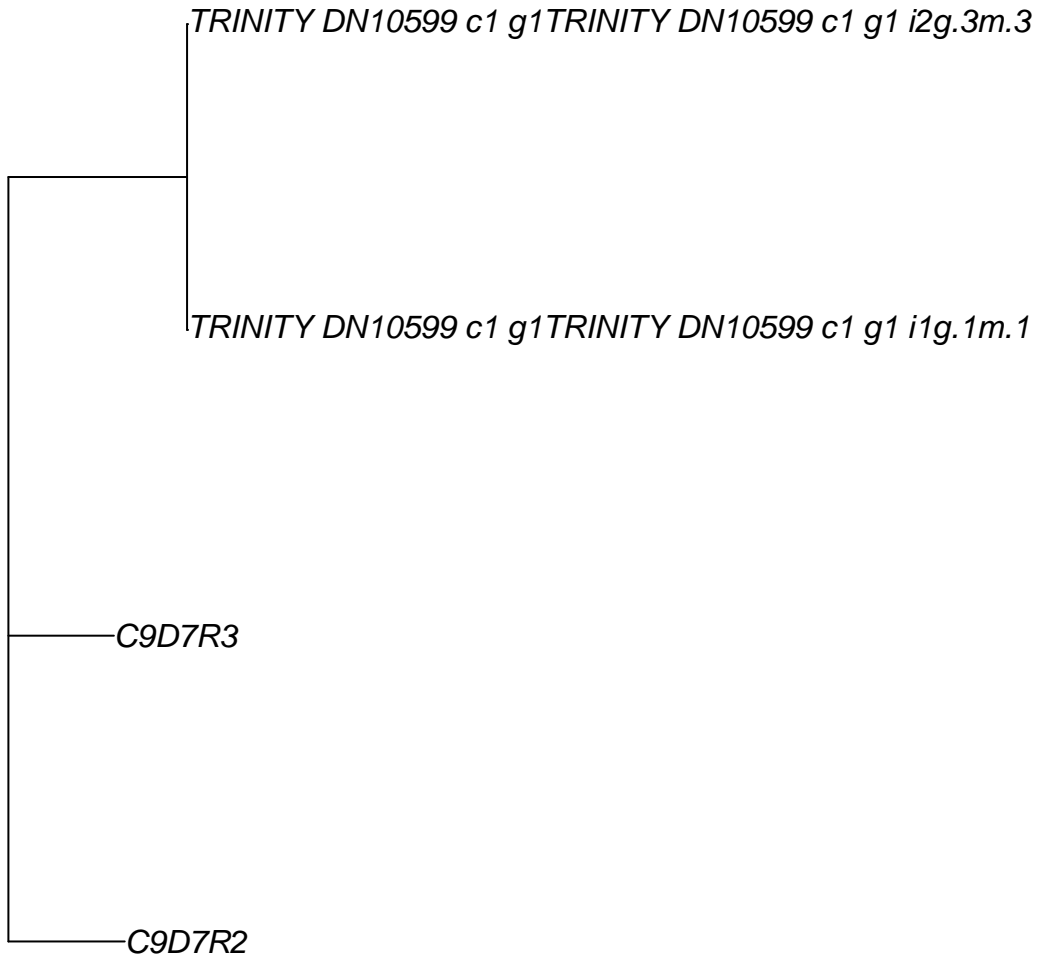

Supplement: Supplemental Information 2 [file peerj-06-5361-s002.gz › FinalOutput_1E-20/Astacin-like_metalloprotease_toxin_3_2/finaltree.pdf]

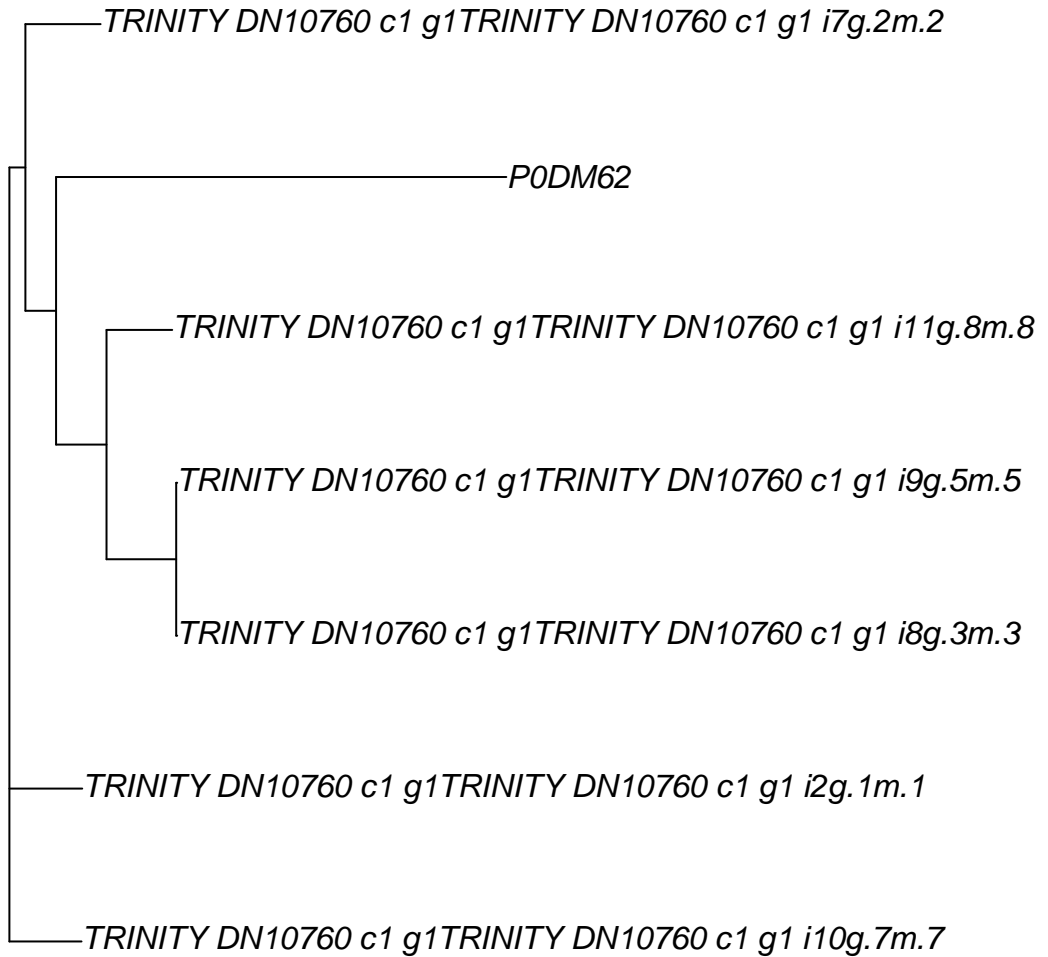

Supplement: Supplemental Information 2 [file peerj-06-5361-s002.gz › FinalOutput_1E-20/Astacin-like_metalloprotease_toxin_5_1/finaltree.pdf]

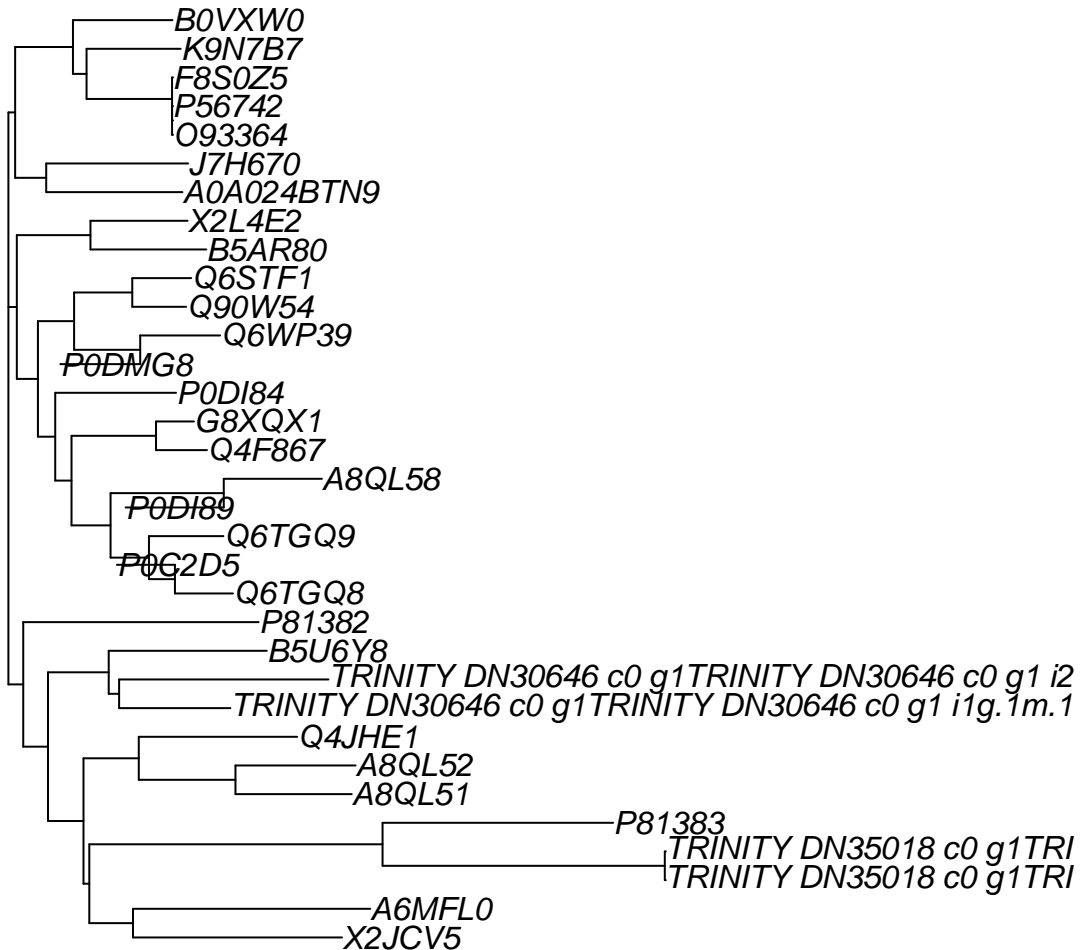

Supplement: Supplemental Information 3 [file peerj-06-5361-s003.gz › FinalOutput_E-20/L-amino-acid_oxidase_31/finaltree.pdf]

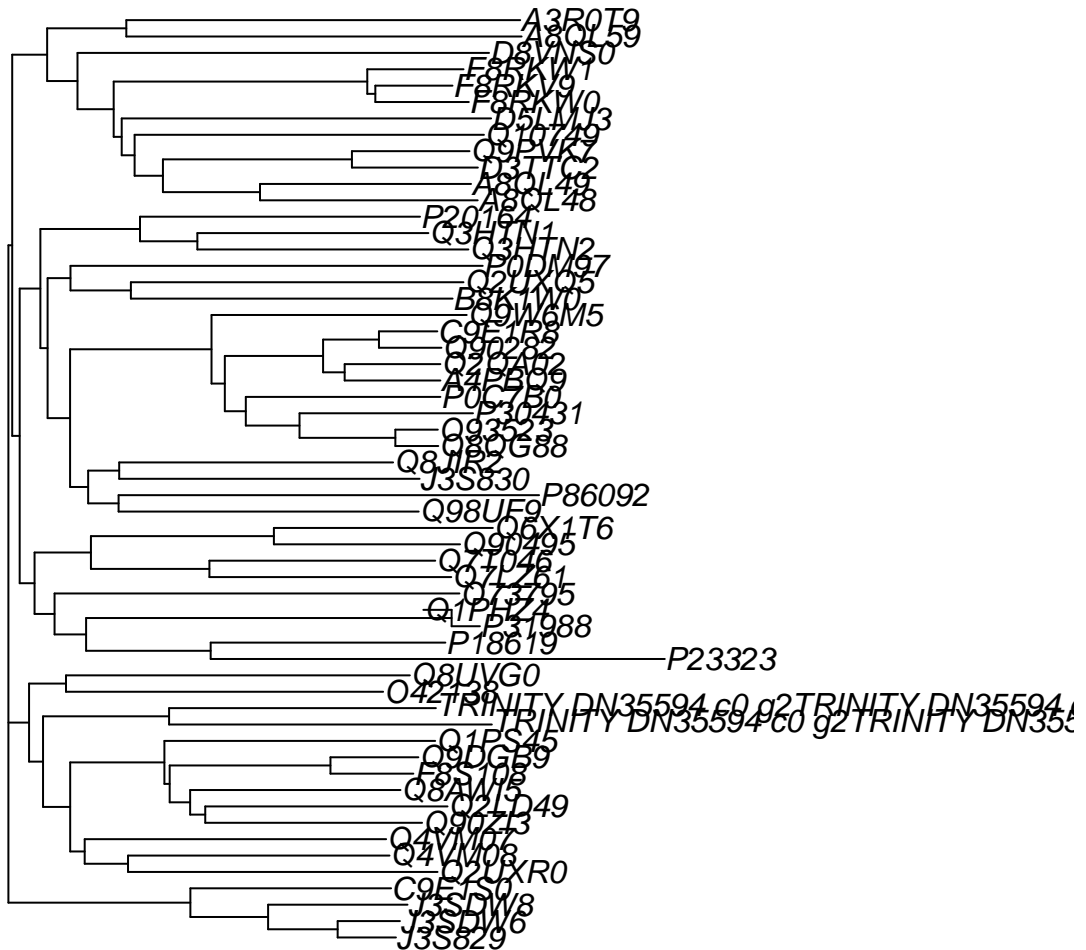

Supplement: Supplemental Information 3 [file peerj-06-5361-s003.gz › FinalOutput_E-20/Zinc_metalloproteinase-disintegrin-like_mikarin_1/finaltree.pdf]

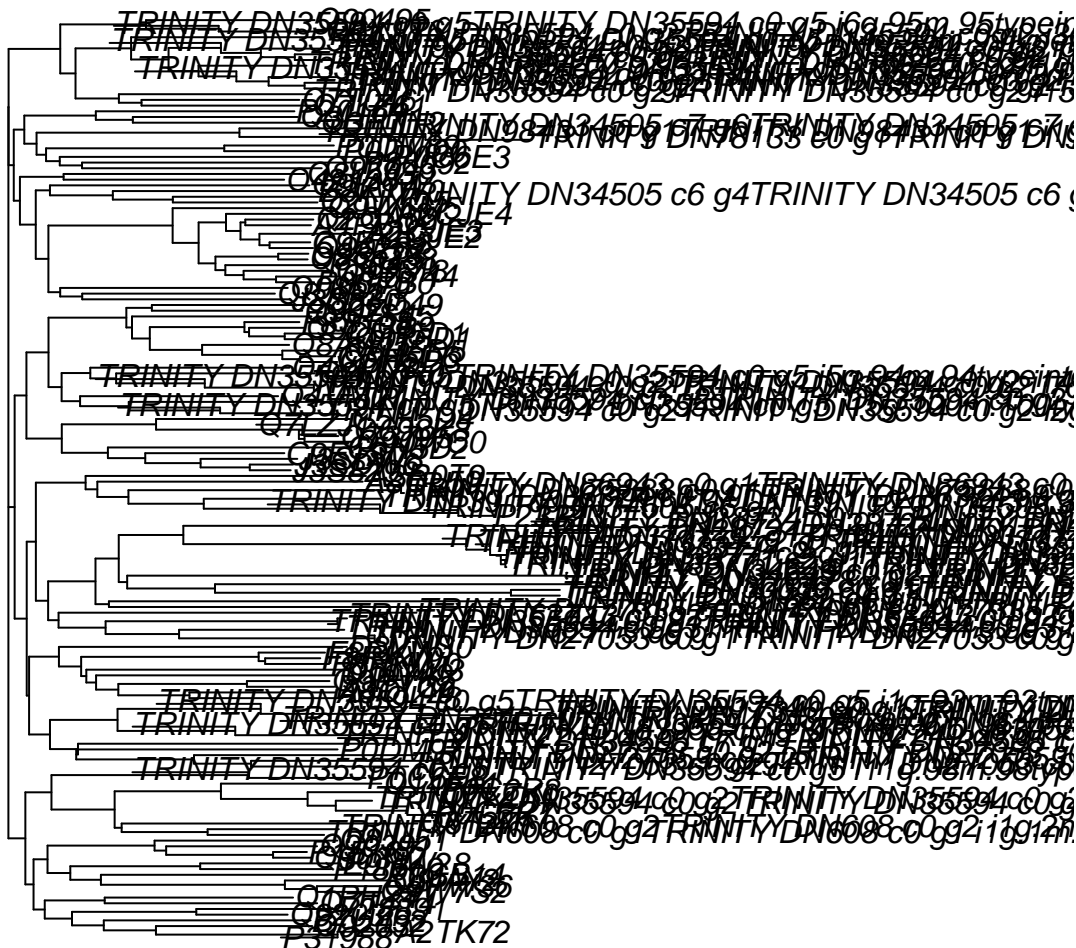

Supplement: Supplemental Information 3 [file peerj-06-5361-s003.gz › FinalOutput_E-20/Zinc_metalloproteinase-disintegrin-like_VAP2B_272/finaltree.pdf]

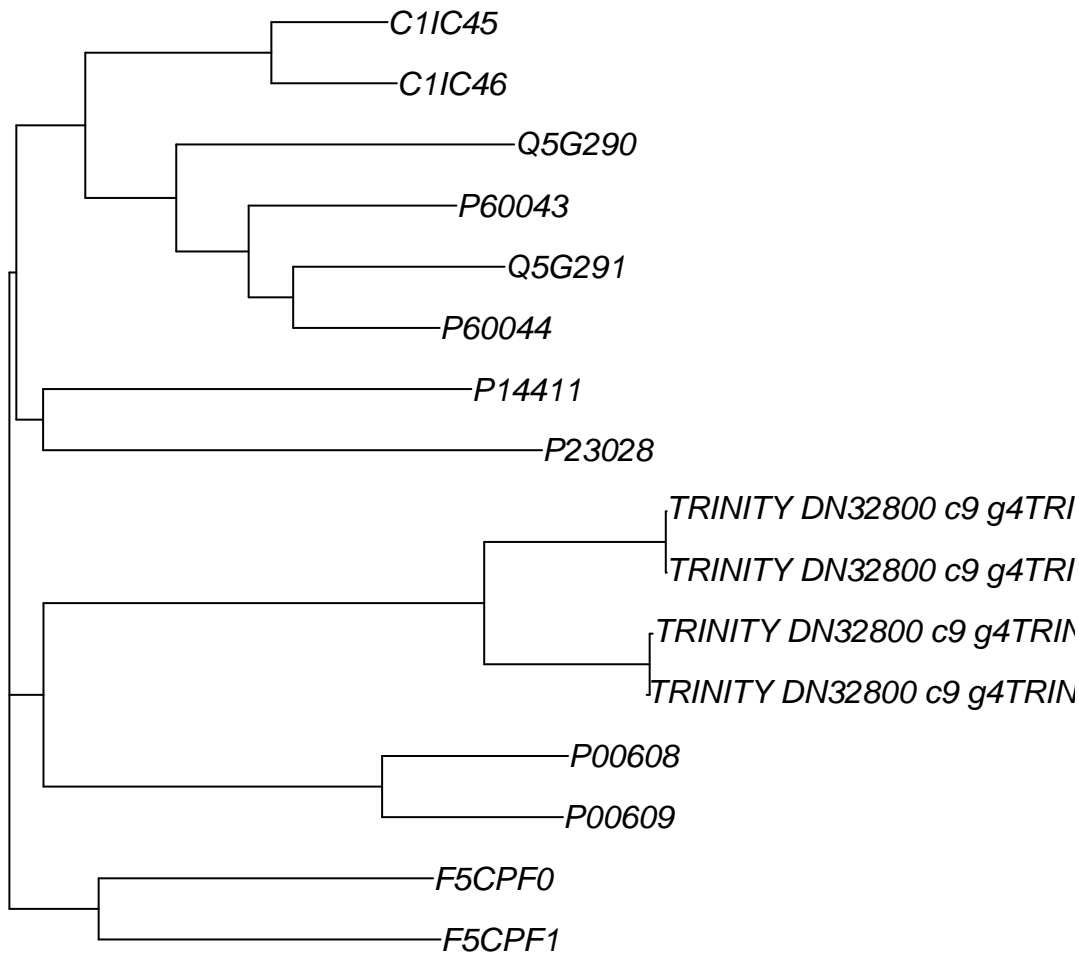

Supplement: Supplemental Information 3 [file peerj-06-5361-s003.gz › FinalOutput_E-20/Neutral_phospholipase_A2_3_21/finaltree.pdf]

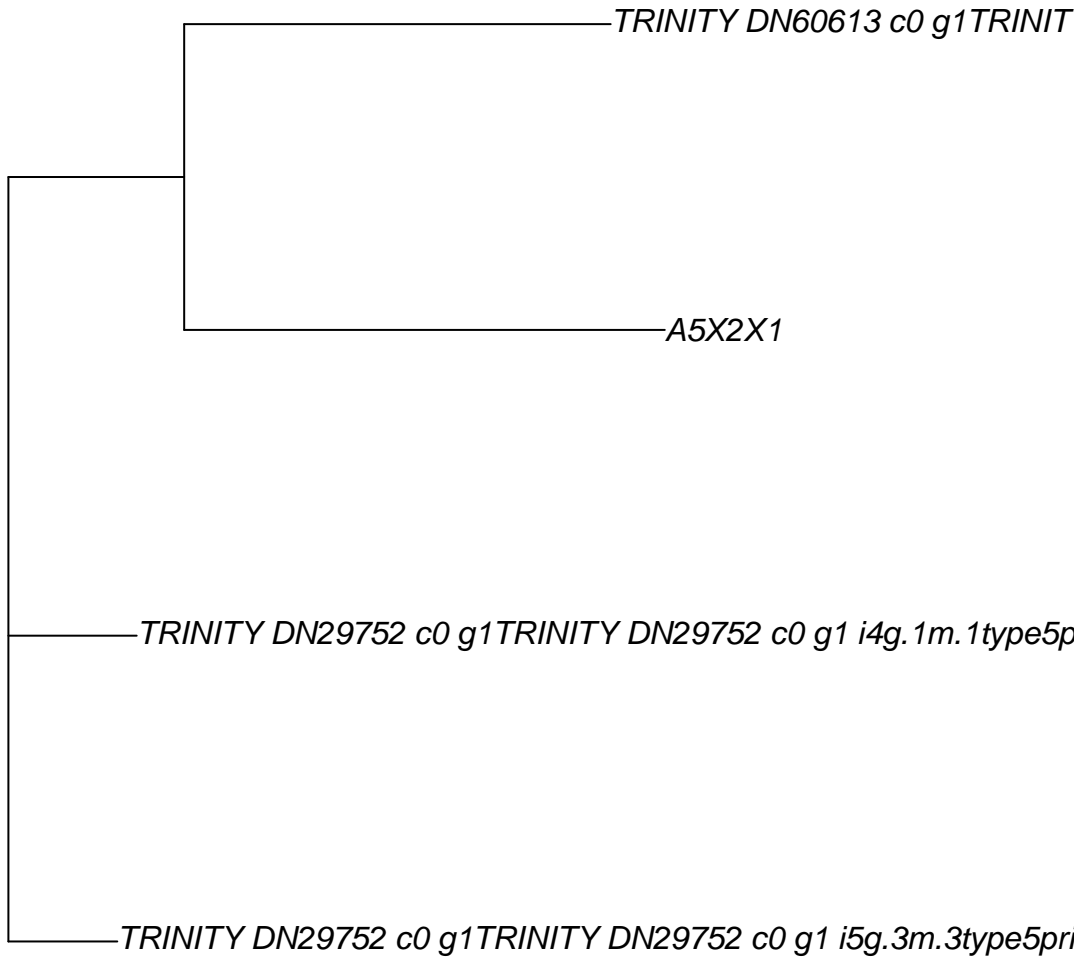

Supplement: Supplemental Information 3 [file peerj-06-5361-s003.gz › FinalOutput_E-20/Kunitz-type_serine_protease_inhibitor_homolog_dendrotoxin_K_154/finaltree.pdf]

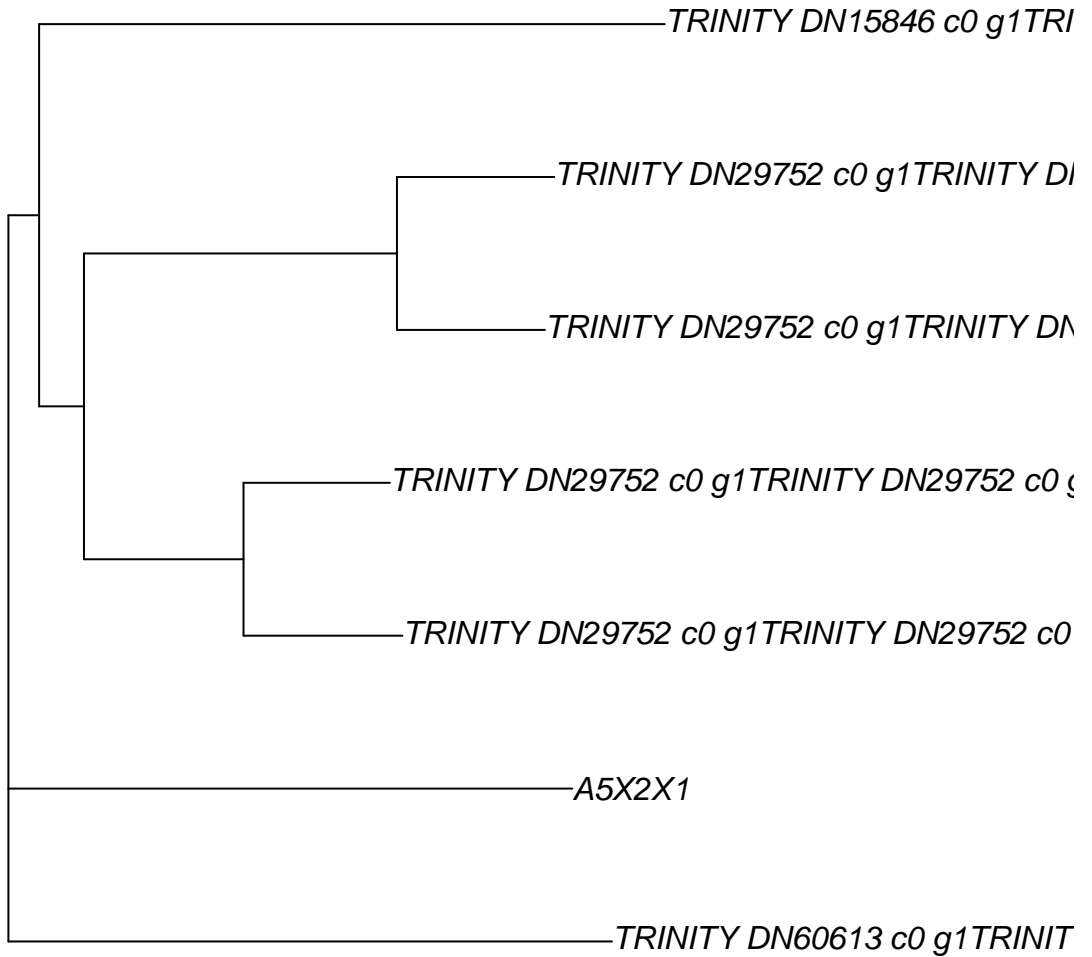

Supplement: Supplemental Information 3 [file peerj-06-5361-s003.gz › FinalOutput_E-20/Kunitz-type_serine_protease_inhibitor_PIVL_13/finaltree.pdf]

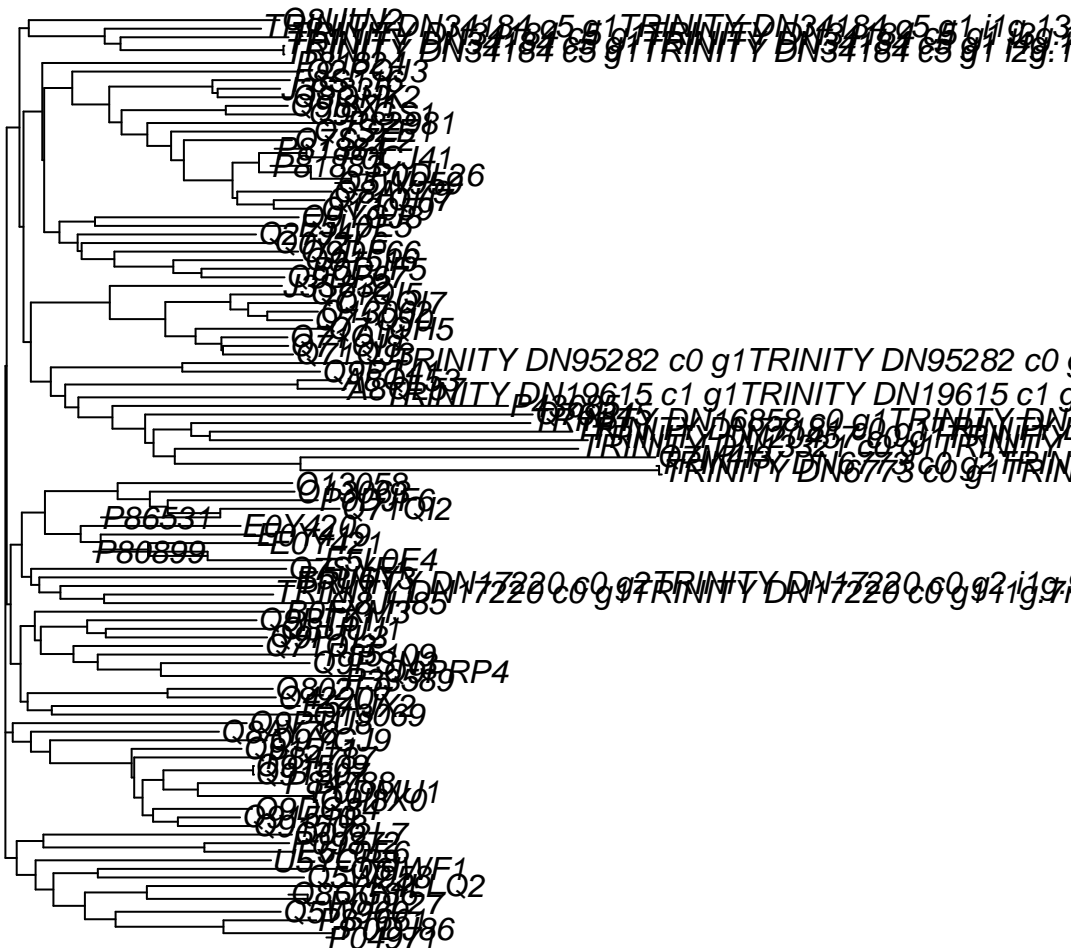

Supplement: Supplemental Information 3 [file peerj-06-5361-s003.gz › FinalOutput_E-20/Factor_V_activator_RVV-V_gamma_142/finaltree.pdf]

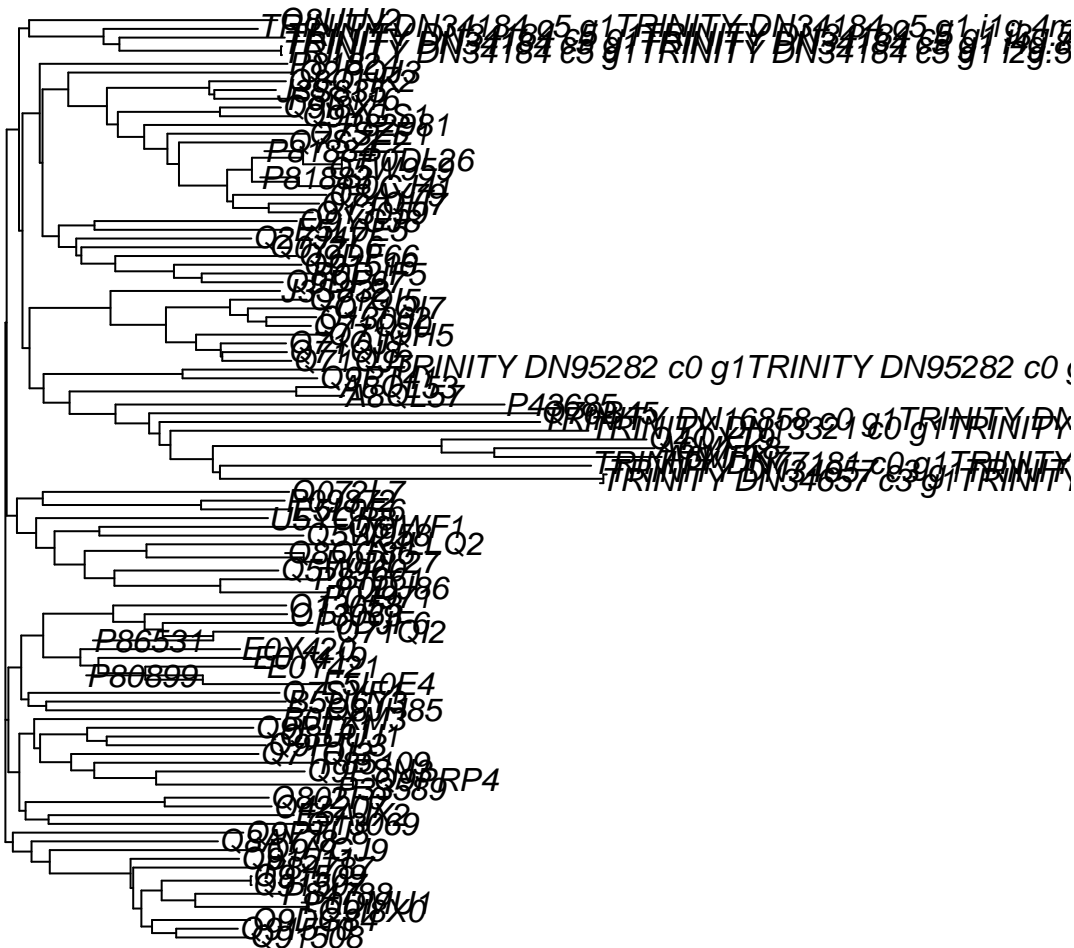

Supplement: Supplemental Information 3 [file peerj-06-5361-s003.gz › FinalOutput_E-20/Thrombin-like_enzyme_TLBm_1/finaltree.pdf]

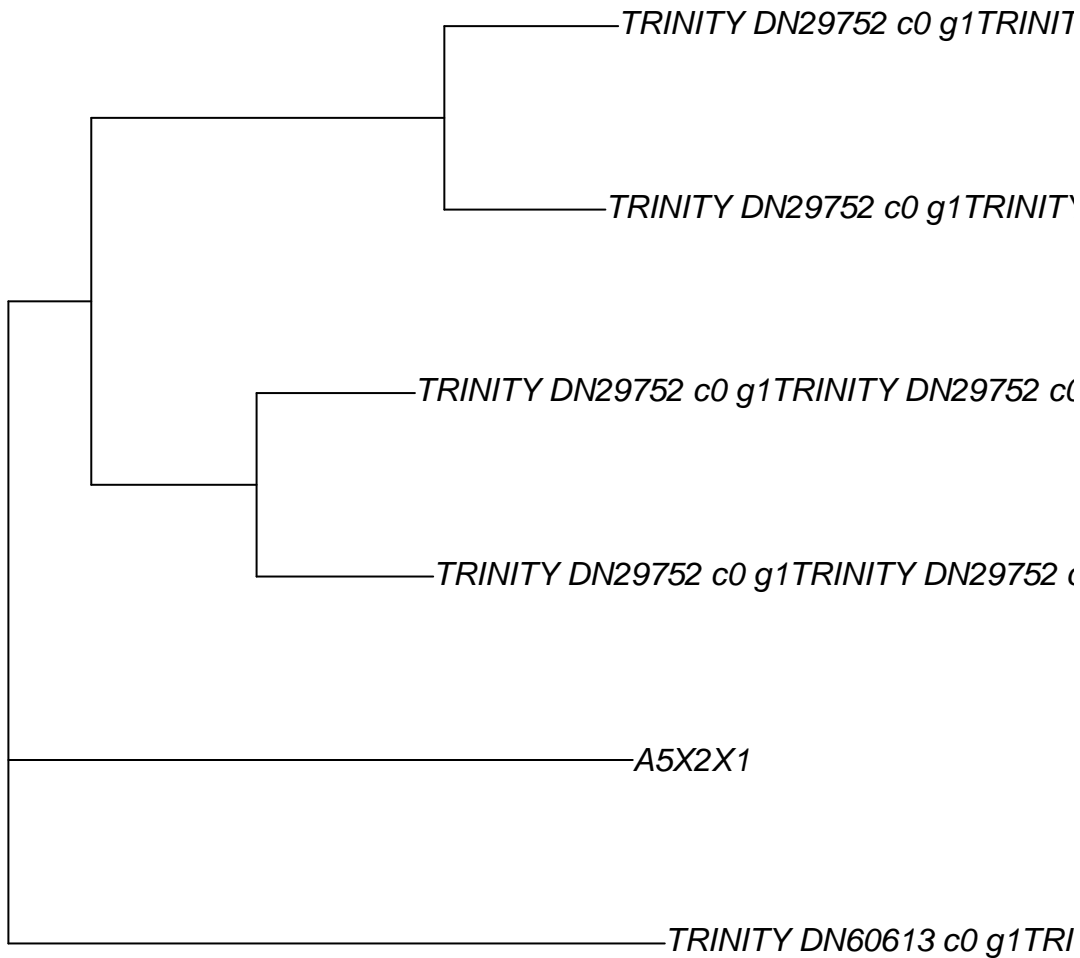

Supplement: Supplemental Information 3 [file peerj-06-5361-s003.gz › FinalOutput_E-20/Protease_inhibitor_1_6/finaltree.pdf]

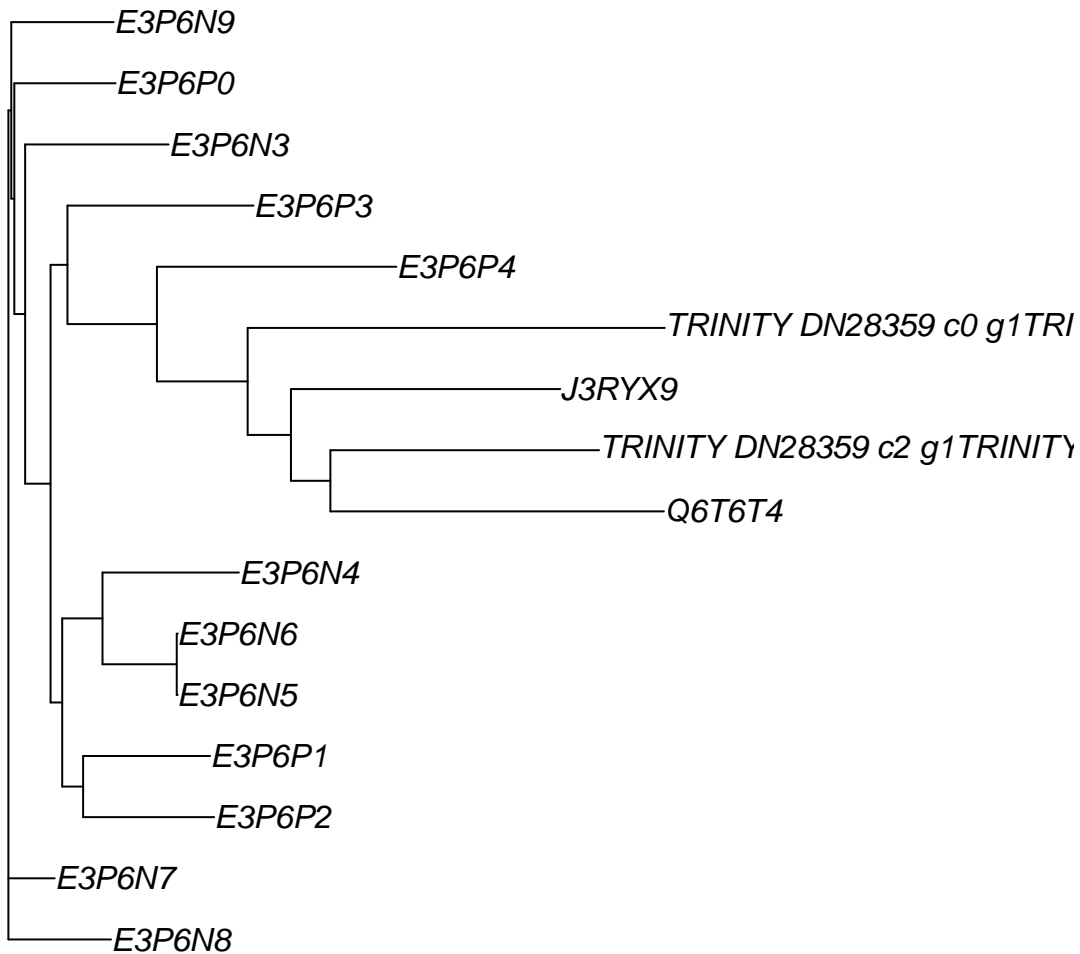

Supplement: Supplemental Information 3 [file peerj-06-5361-s003.gz › FinalOutput_E-20/Cystatin_2/finaltree.pdf]

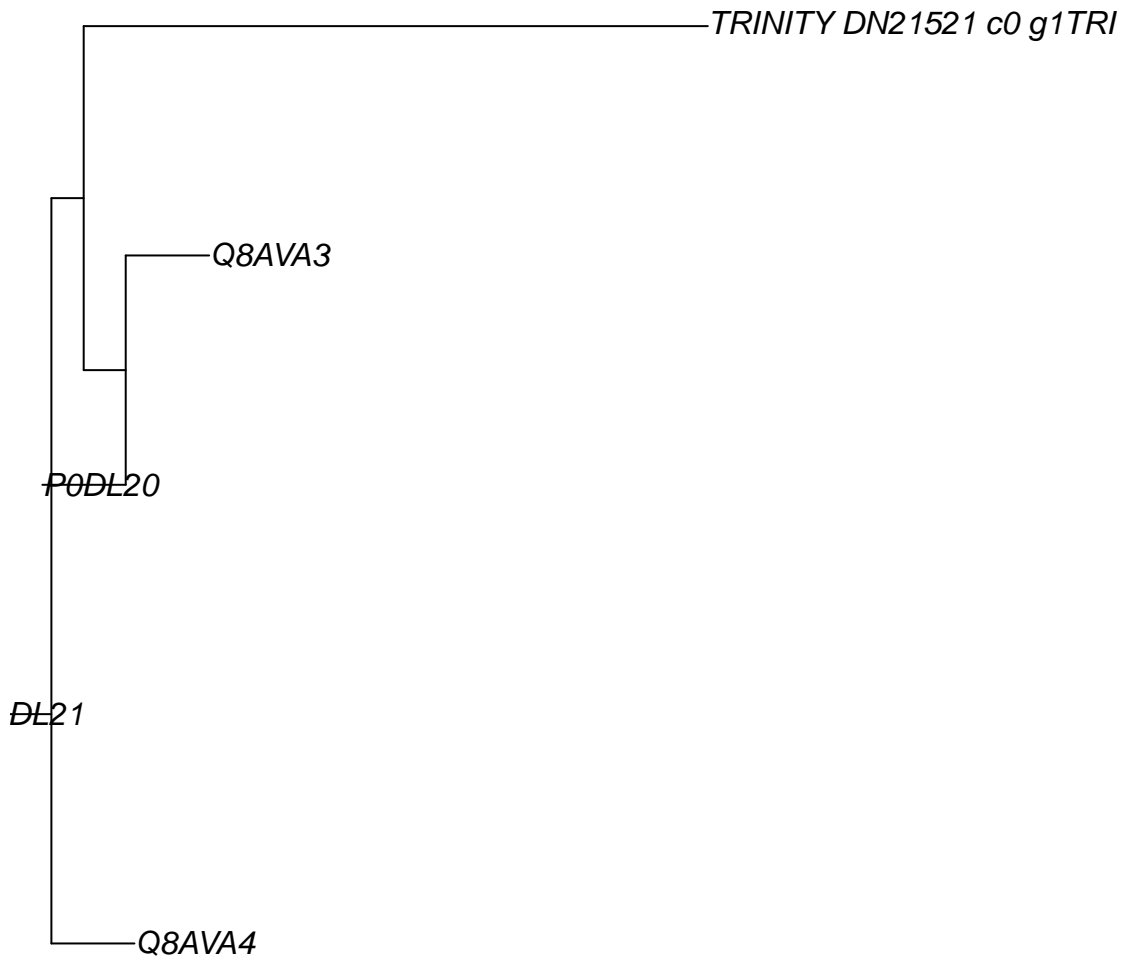

Supplement: Supplemental Information 3 [file peerj-06-5361-s003.gz › FinalOutput_E-20/Cysteine-rich_venom_protein_bucarin_1/finaltree.pdf]

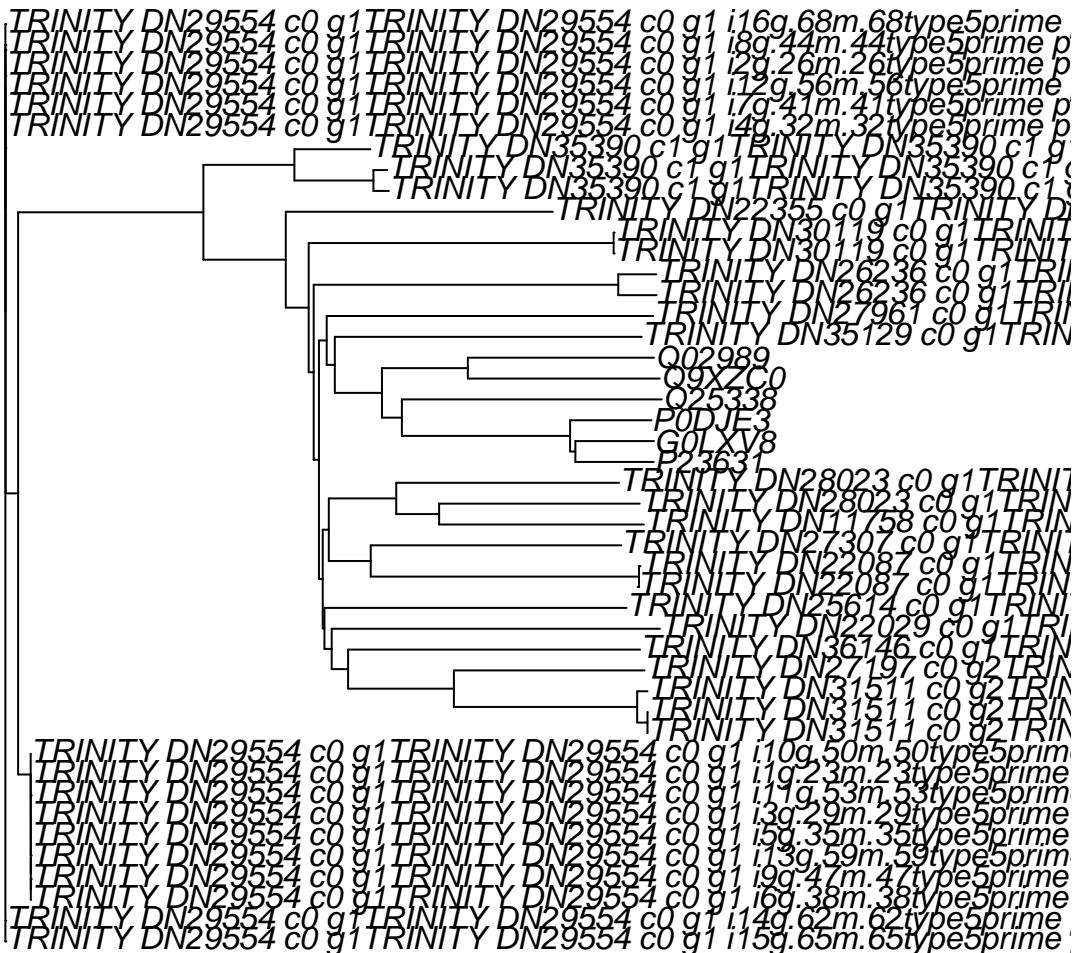

Supplement: Supplemental Information 3 [file peerj-06-5361-s003.gz › FinalOutput_E-20/Alpha-latroinsectotoxin-Lt1a_2/finaltree.pdf]

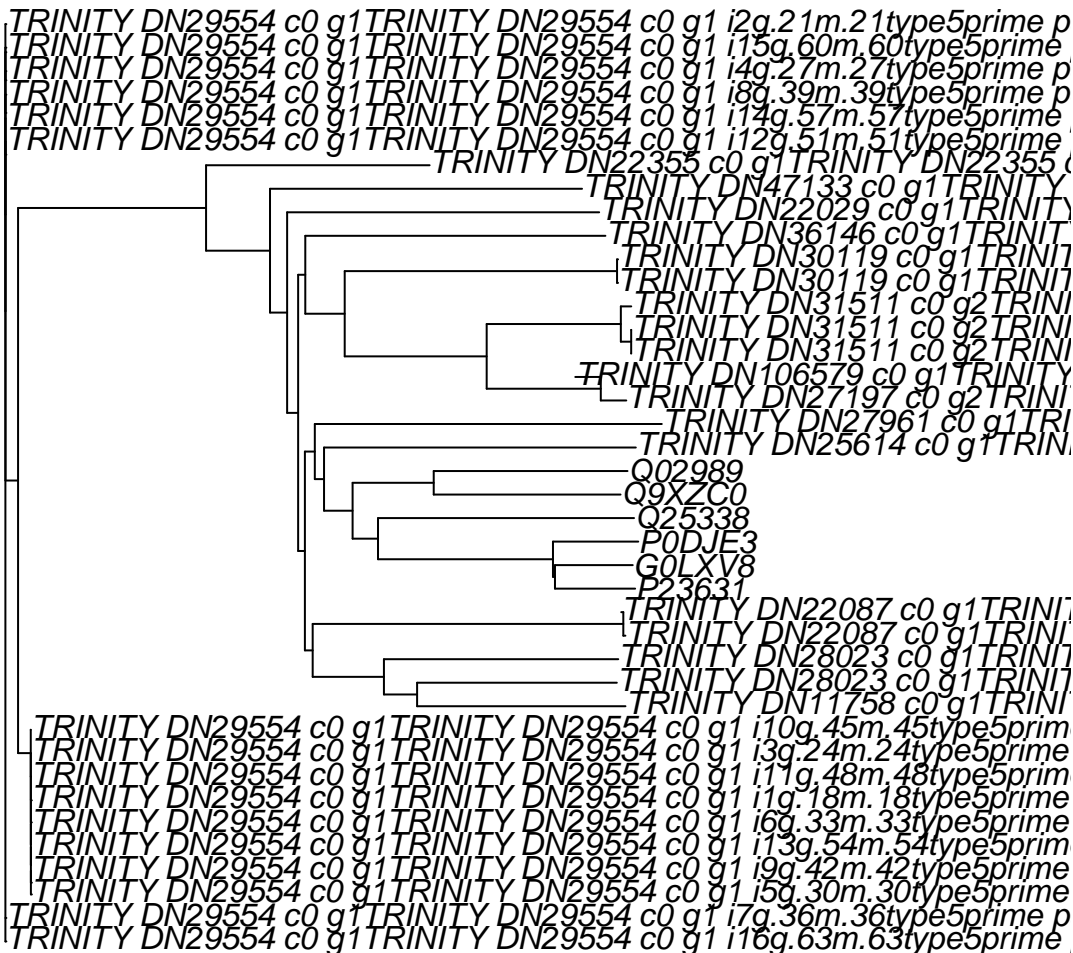

Supplement: Supplemental Information 3 [file peerj-06-5361-s003.gz › FinalOutput_E-20/Delta-latroinsectotoxin-Lt1a_1/finaltree.pdf]

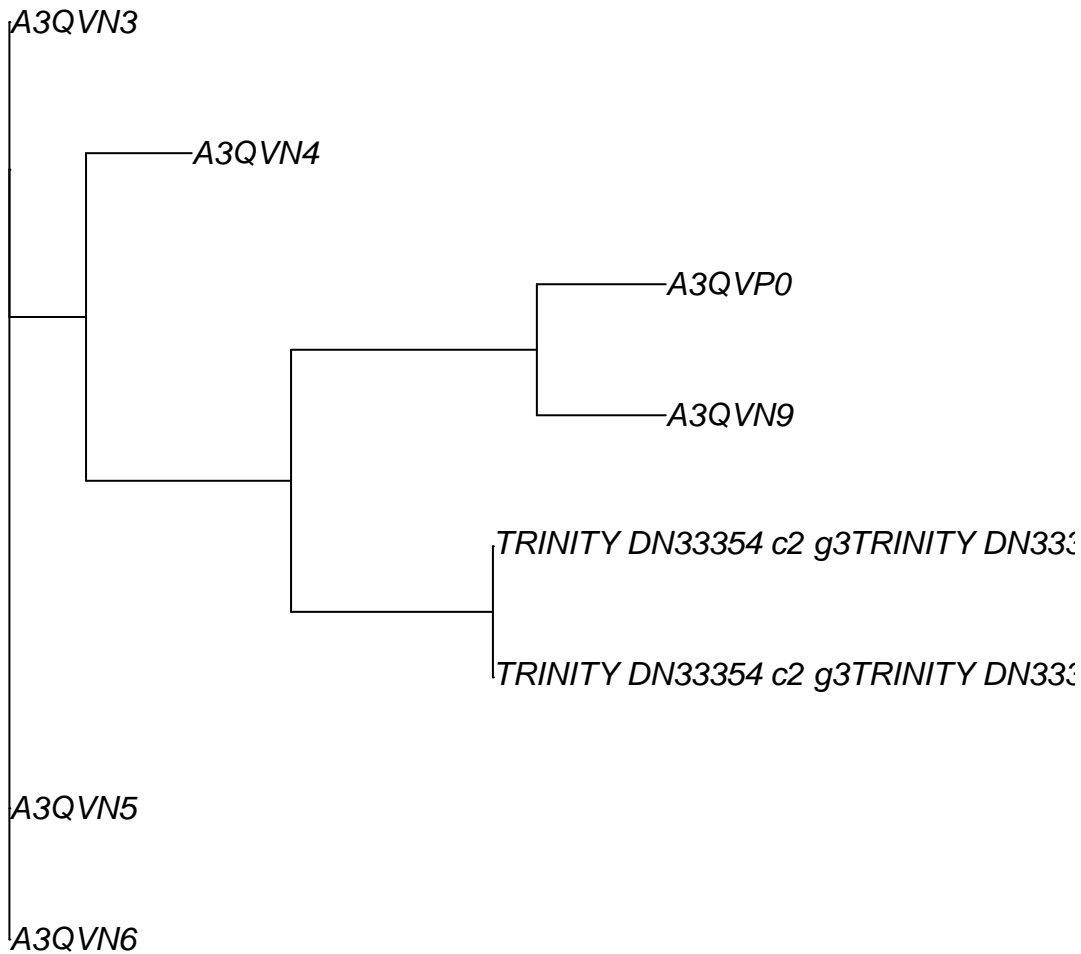

Supplement: Supplemental Information 3 [file peerj-06-5361-s003.gz › FinalOutput_E-20/Hyaluronidase_CdtHya1_1/finaltree.pdf]

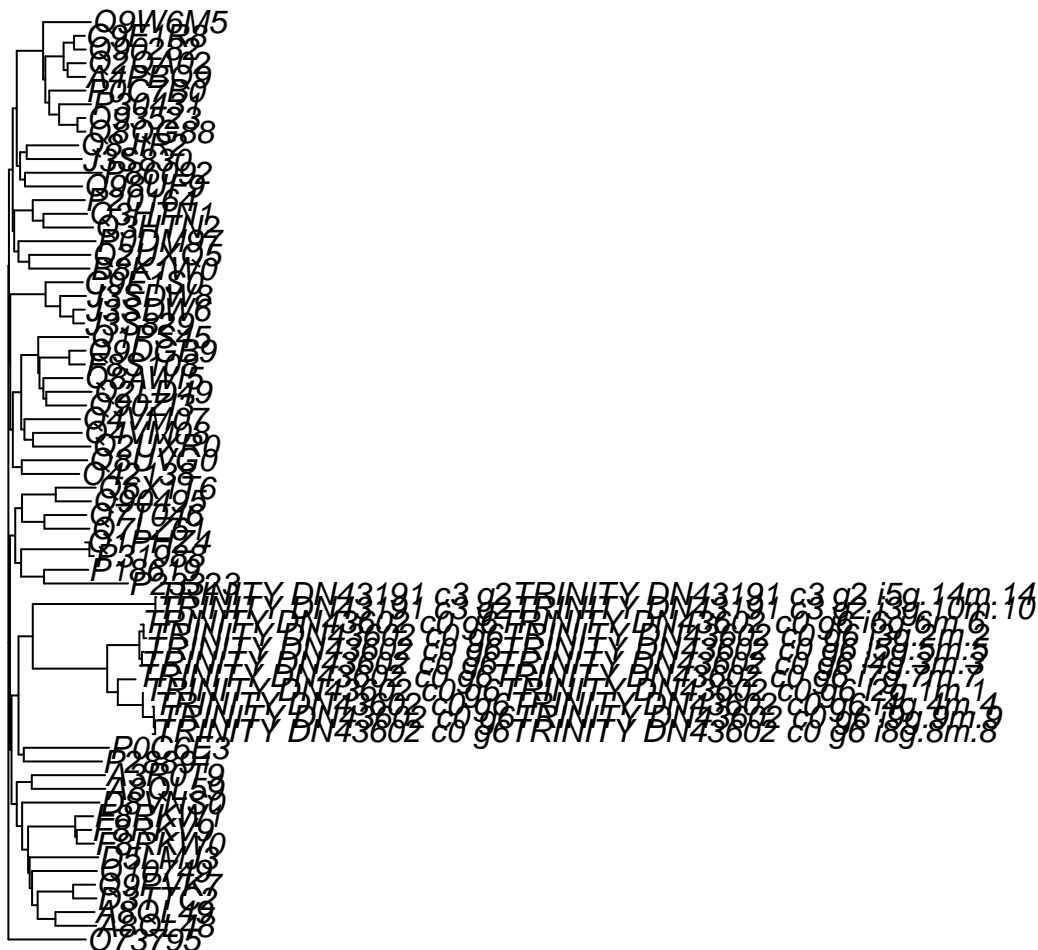

Supplement: Supplemental Information 4 [file peerj-06-5361-s004.gz › FinalOutput_E-20/Zinc_metalloproteinase-disintegrin-like_VAP2B_272/finaltree.pdf]

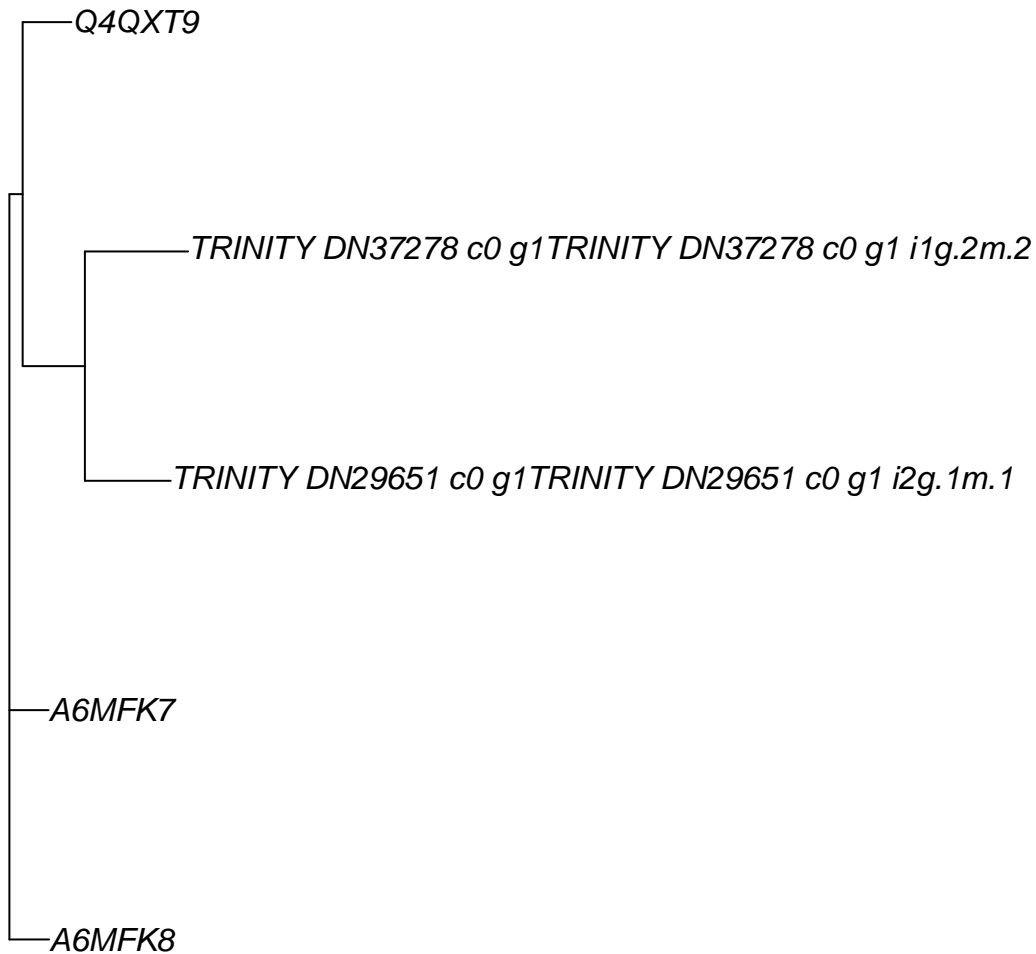

Supplement: Supplemental Information 4 [file peerj-06-5361-s004.gz › FinalOutput_E-20/Factor_V_activator_RVV-V_gamma_142/finaltree.pdf]

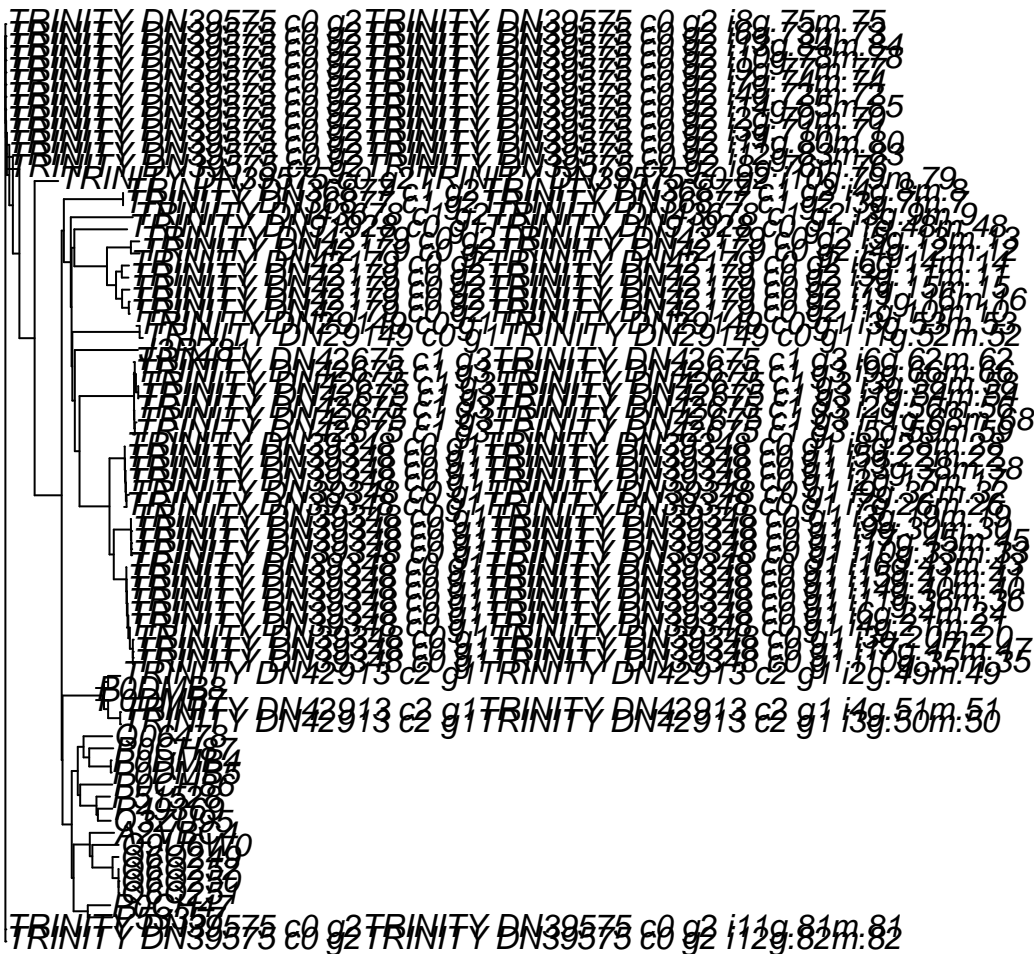

Supplement: Supplemental Information 4 [file peerj-06-5361-s004.gz › FinalOutput_E-20/Phospholipase_A1_19/finaltree.pdf]

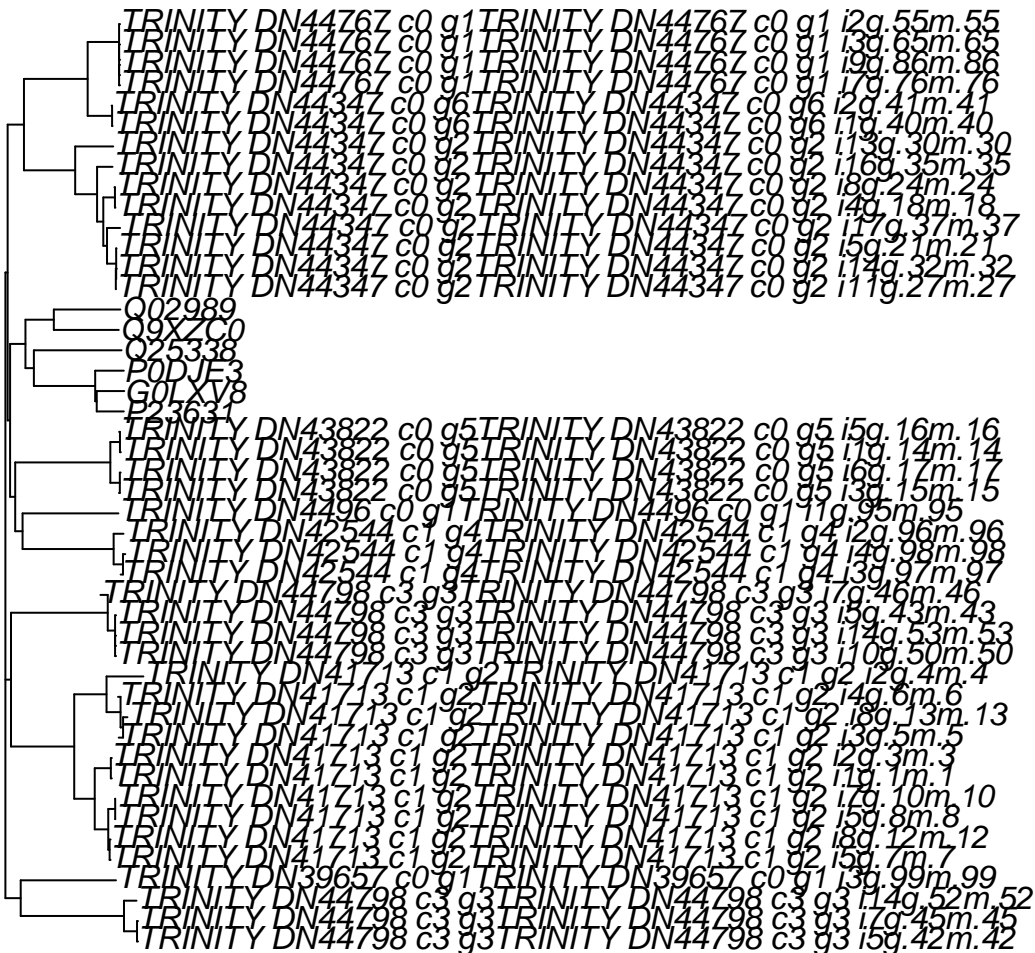

Supplement: Supplemental Information 4 [file peerj-06-5361-s004.gz › FinalOutput_E-20/Alpha-latroinsectotoxin-Lt1a_2/finaltree.pdf]

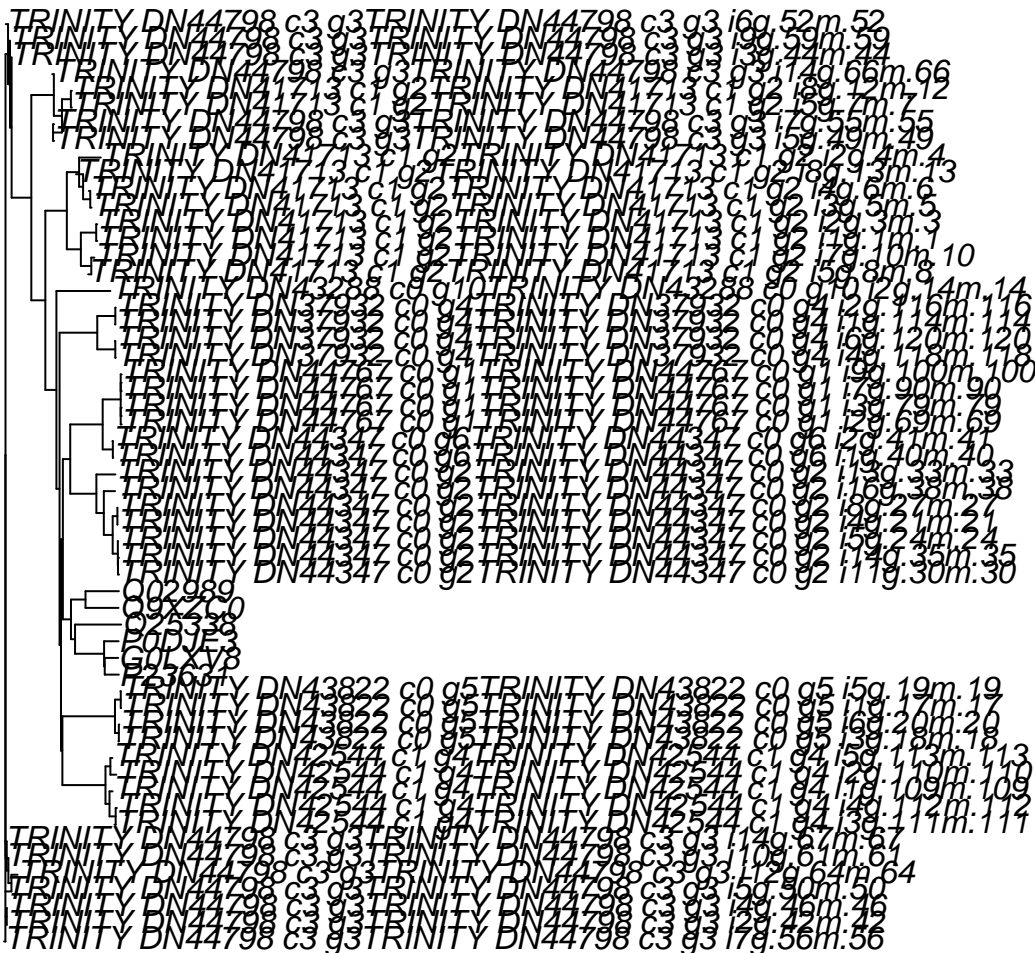

Supplement: Supplemental Information 4 [file peerj-06-5361-s004.gz › FinalOutput_E-20/Delta-latroinsectotoxin-Lt1a_1/finaltree.pdf]

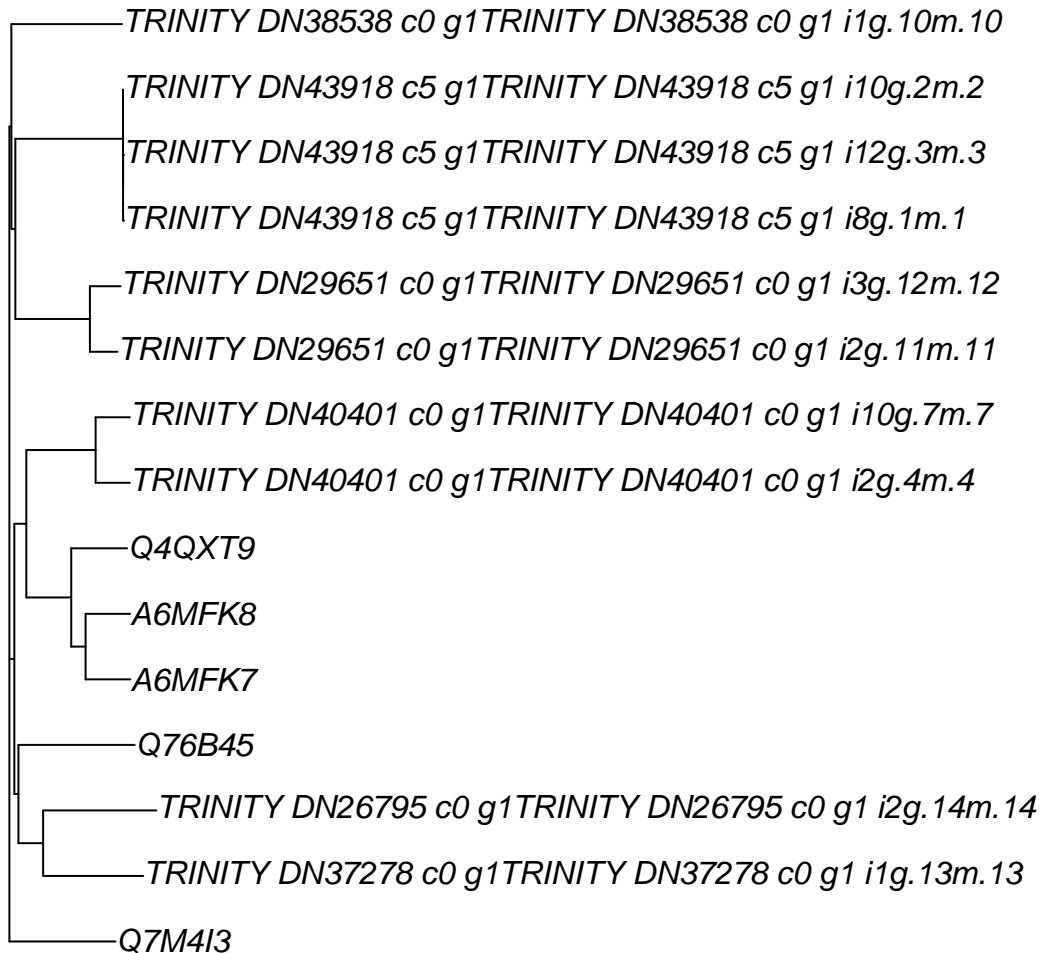

Supplement: Supplemental Information 4 [file peerj-06-5361-s004.gz › FinalOutput_E-20/Venom_peptide_isomerase_heavy_chain_2/finaltree.pdf]

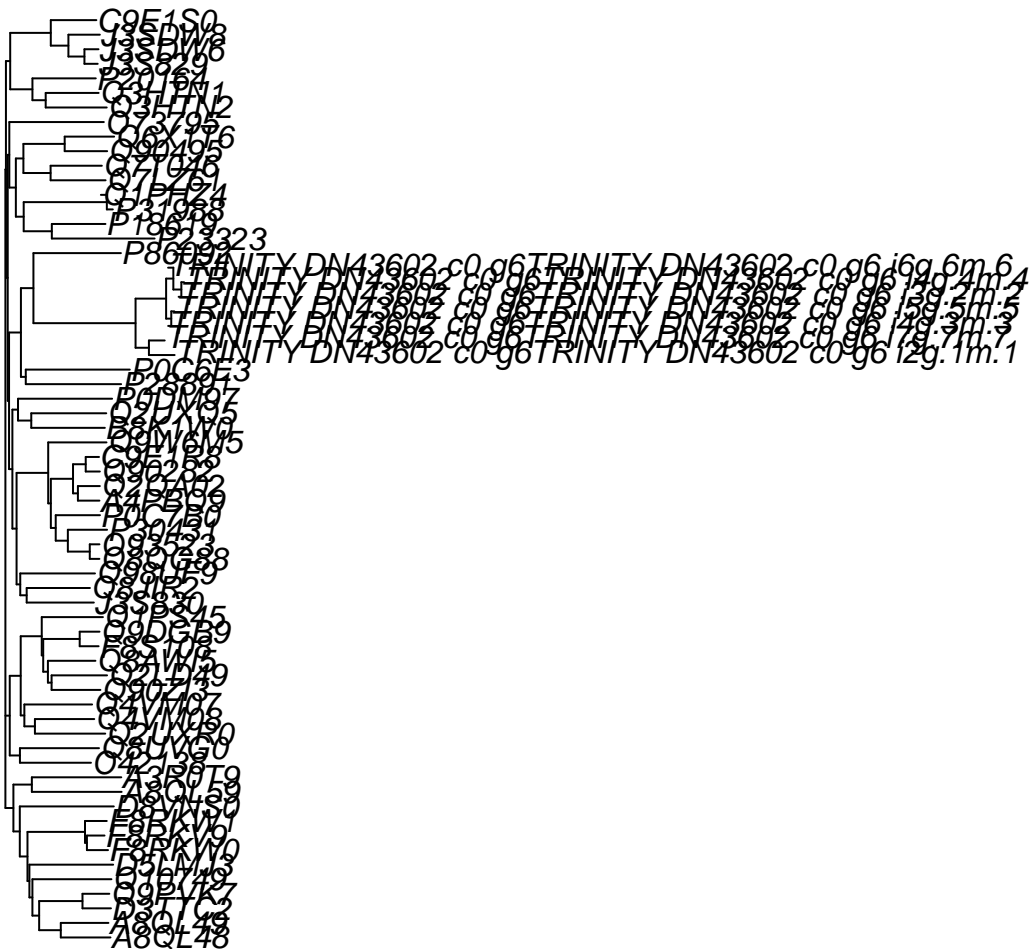

Supplement: Supplemental Information 4 [file peerj-06-5361-s004.gz › FinalOutput_E-20/Snake_venom_metalloproteinase_acutolysin-C_1/finaltree.pdf]

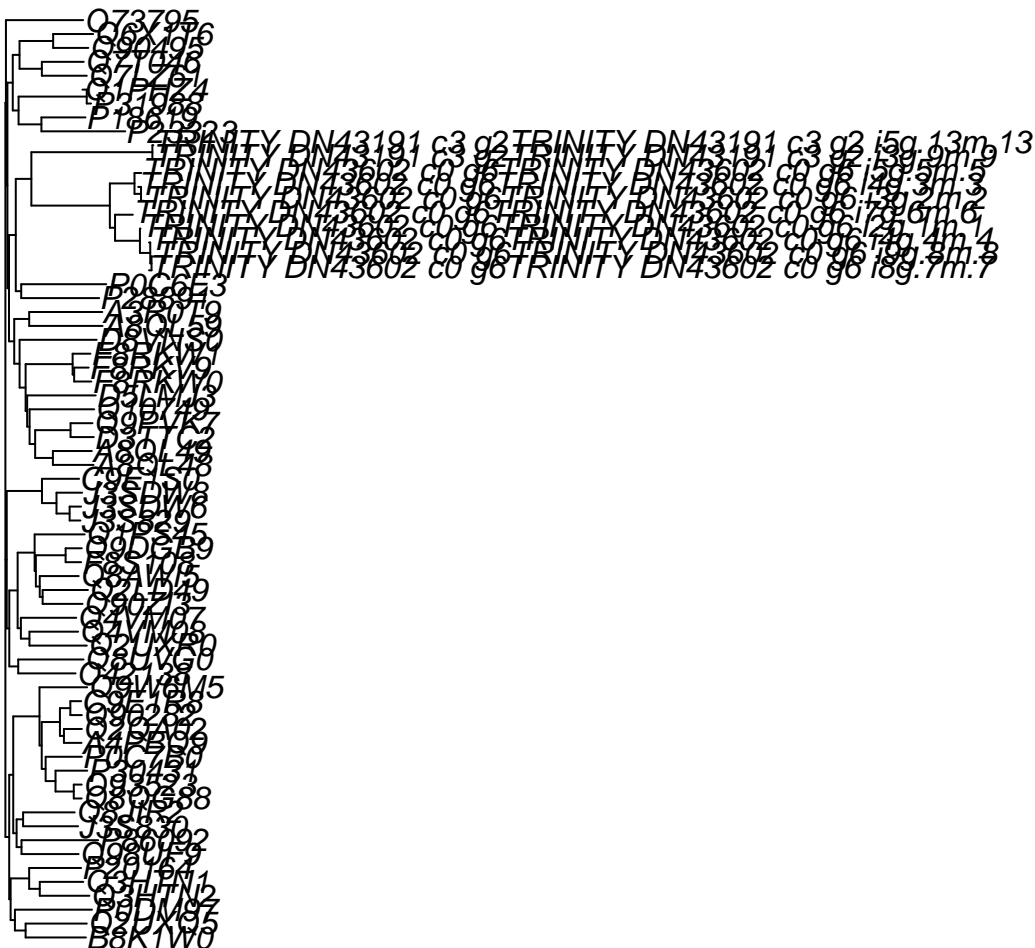

Supplement: Supplemental Information 4 [file peerj-06-5361-s004.gz › FinalOutput_E-20/Zinc_metalloproteinase@disintegrin_107/finaltree.pdf]

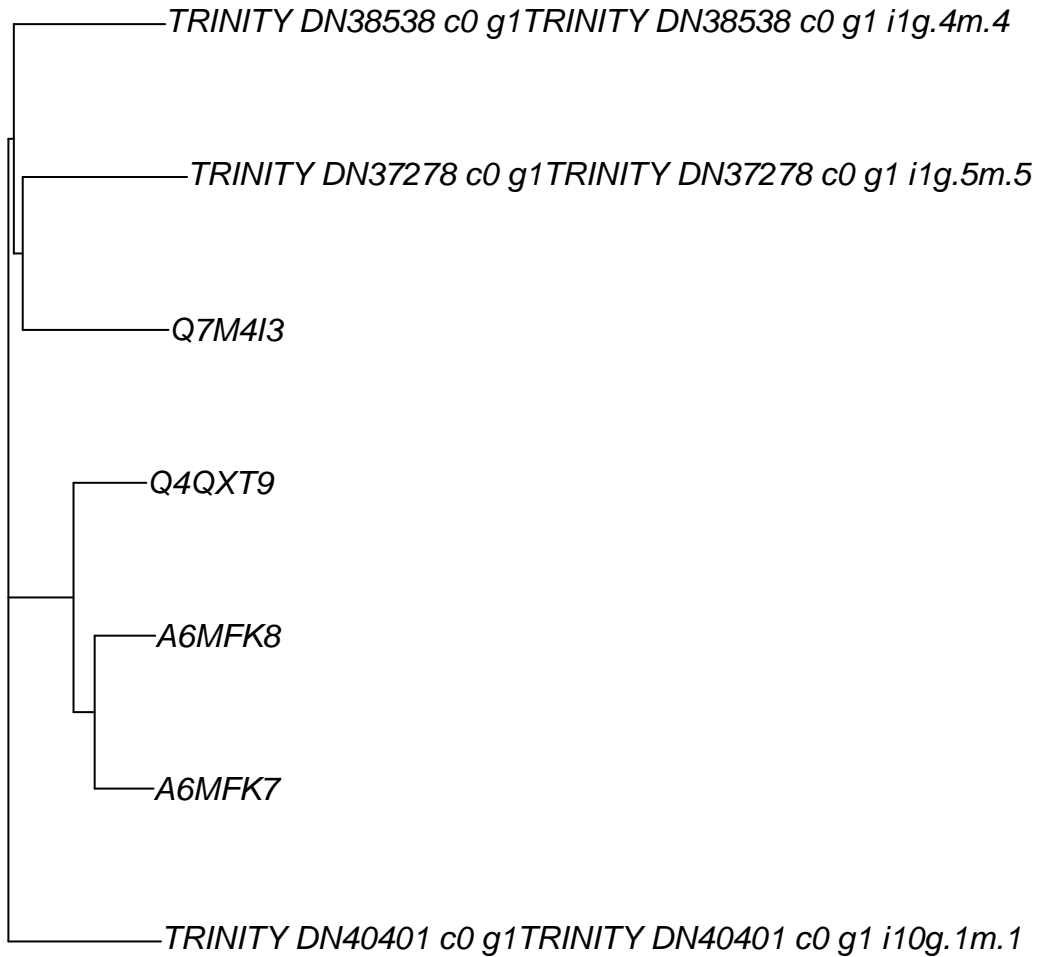

Supplement: Supplemental Information 4 [file peerj-06-5361-s004.gz › FinalOutput_E-20/Thrombin-like_enzyme_ancrod_11/finaltree.pdf]

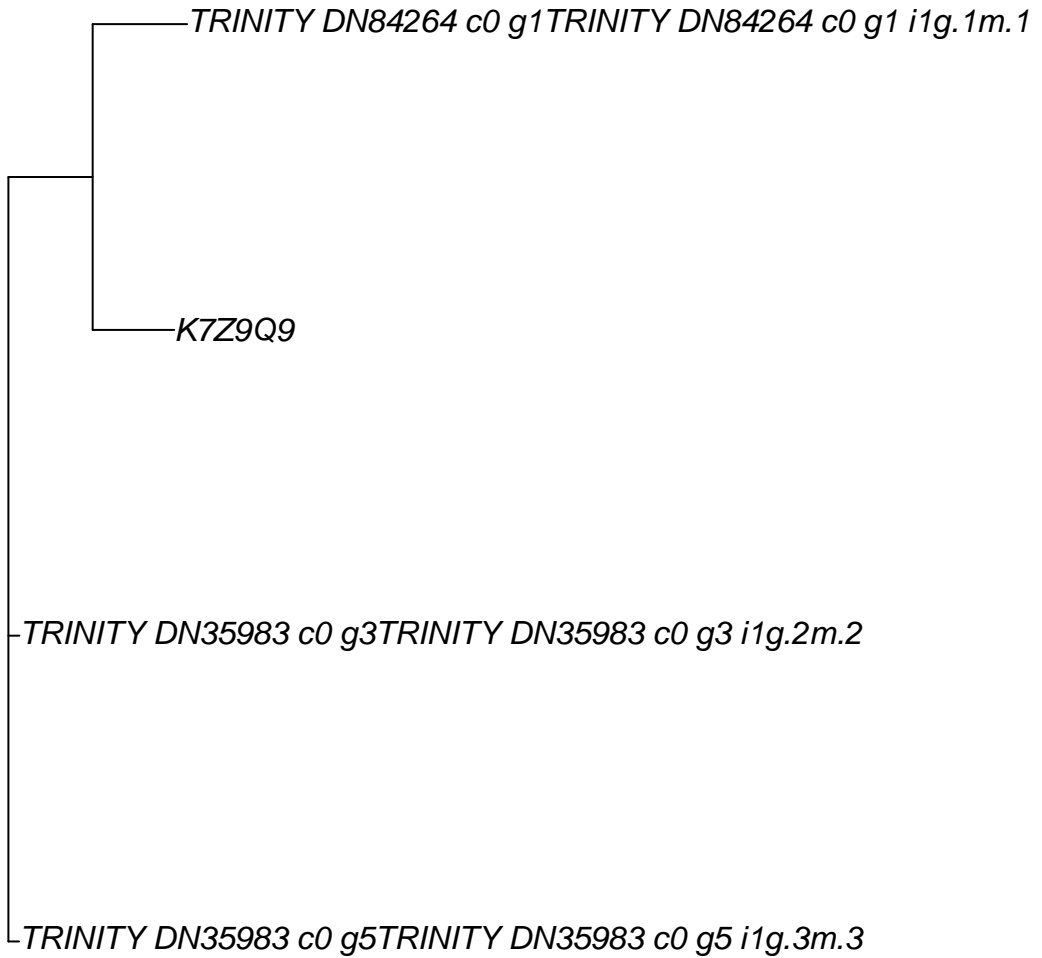

Supplement: Supplemental Information 4 [file peerj-06-5361-s004.gz › FinalOutput_E-20/Nematocyte_expressed_protein_6_1/finaltree.pdf]

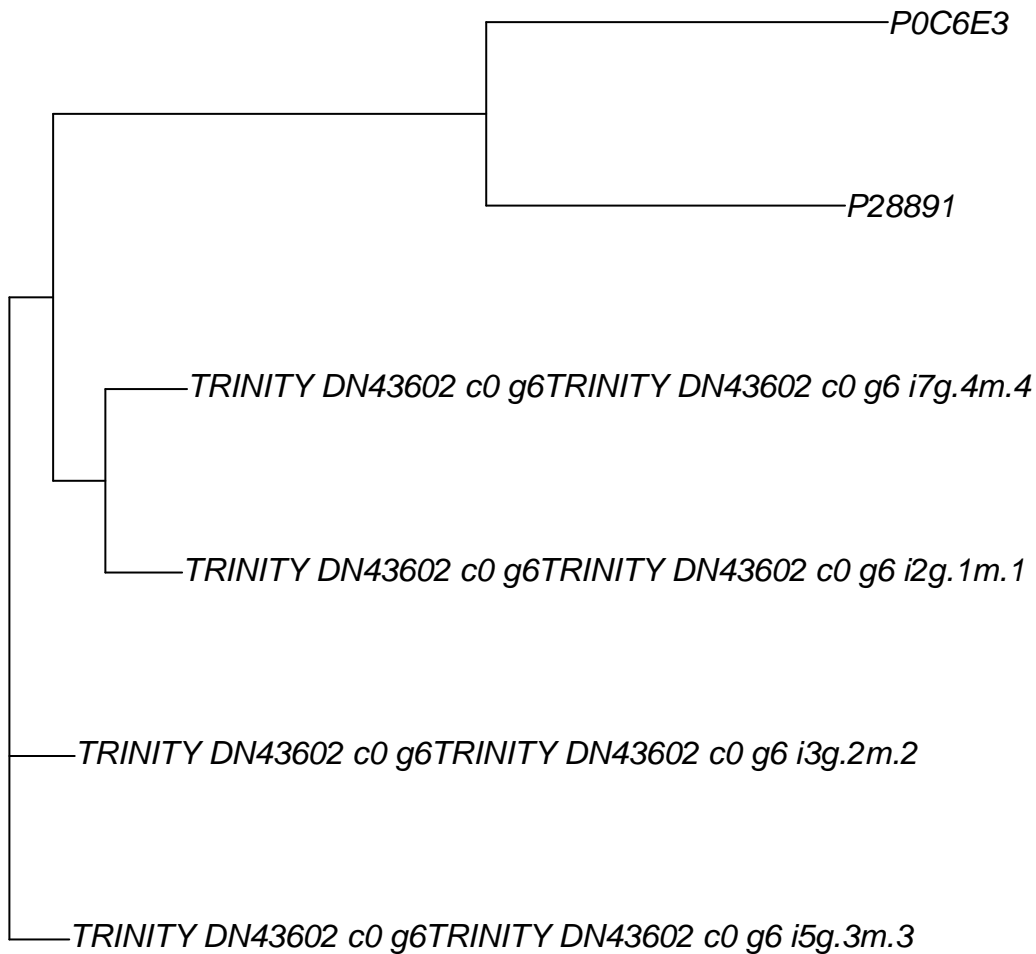

Supplement: Supplemental Information 4 [file peerj-06-5361-s004.gz › FinalOutput_E-20/Zinc_metalloproteinase_recombinant_fibrinogenase_II_1/finaltree.pdf]

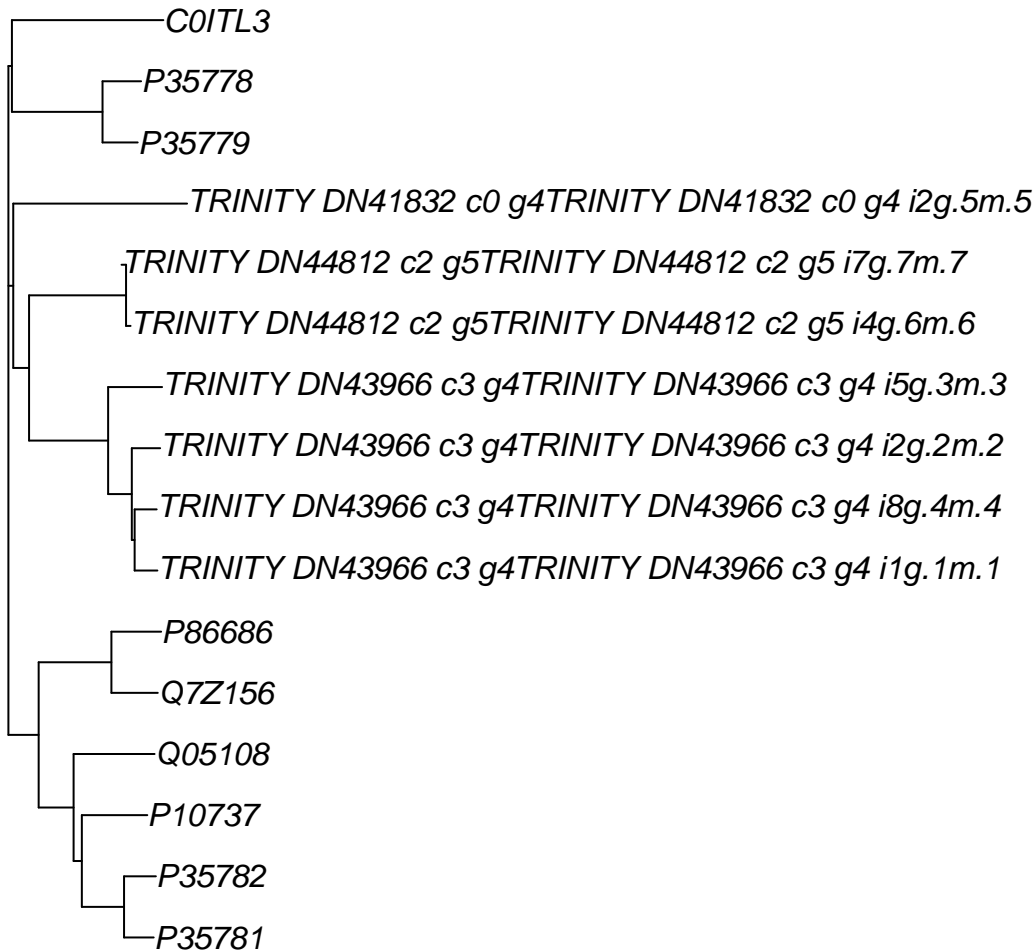

Supplement: Supplemental Information 4 [file peerj-06-5361-s004.gz › FinalOutput_E-20/Venom_allergen_5/finaltree.pdf]

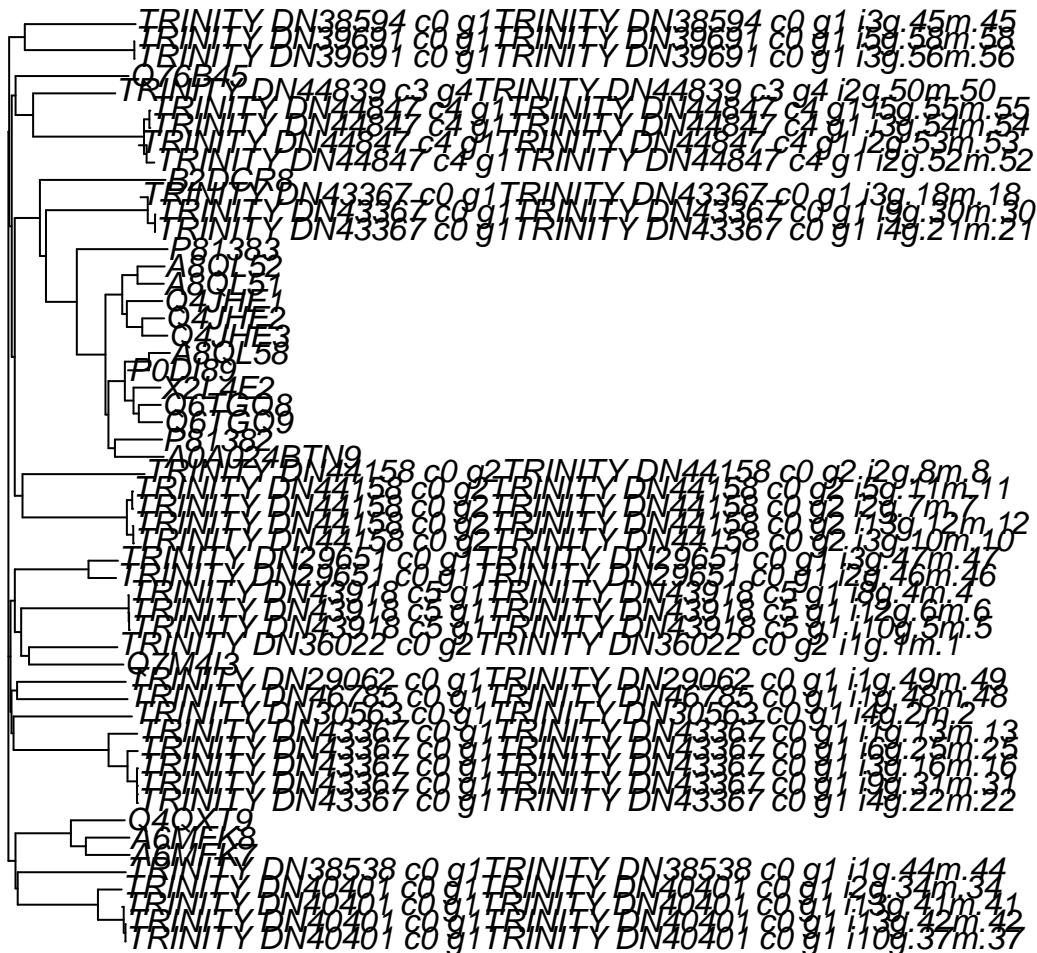

Supplement: Supplemental Information 4 [file peerj-06-5361-s004.gz › FinalOutput_E-20/Venom_prothrombin_activator_pseutarin-C_catalytic_subunit_12/finaltree.pdf]

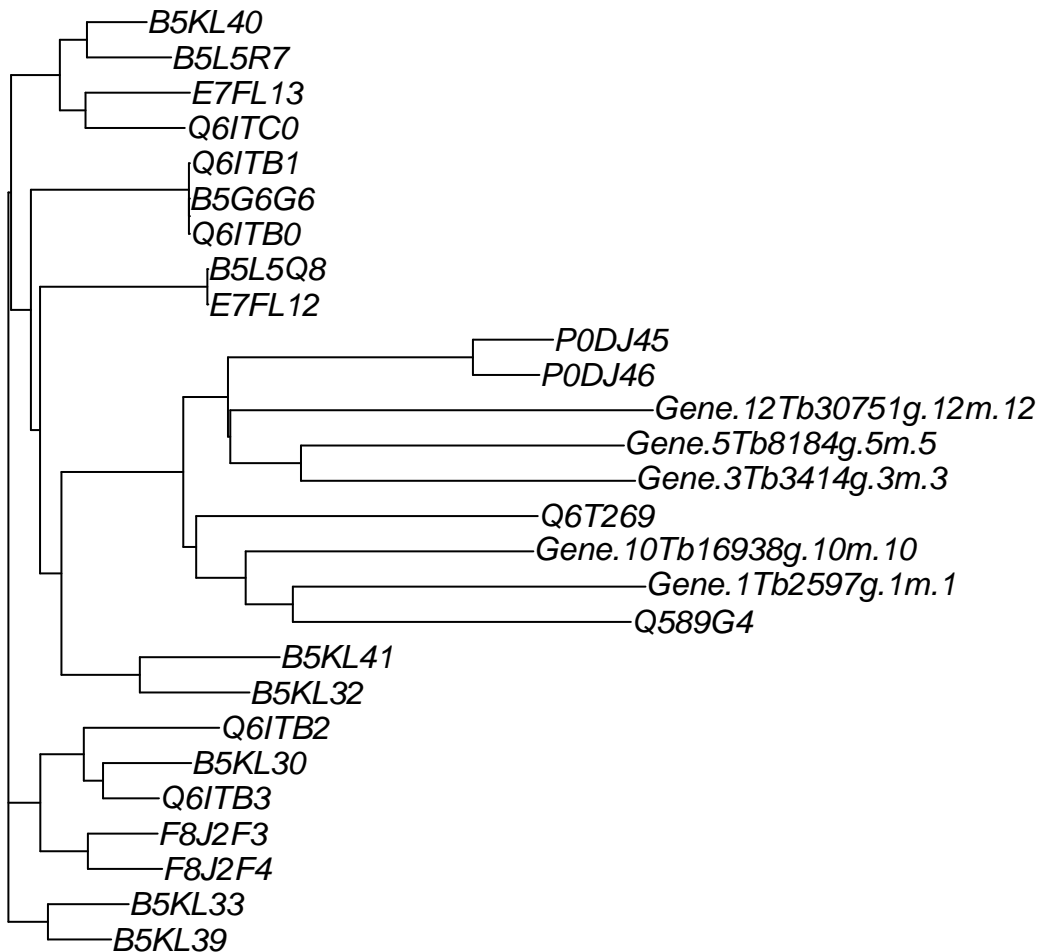

Supplement: Supplemental Information 5 [file peerj-06-5361-s005.gz › FinalOutput_GAS_1E-6/Kunitz-type_serine_protease_inhibitor_U1-aranetoxin-Av1a_1/finaltree.pdf]

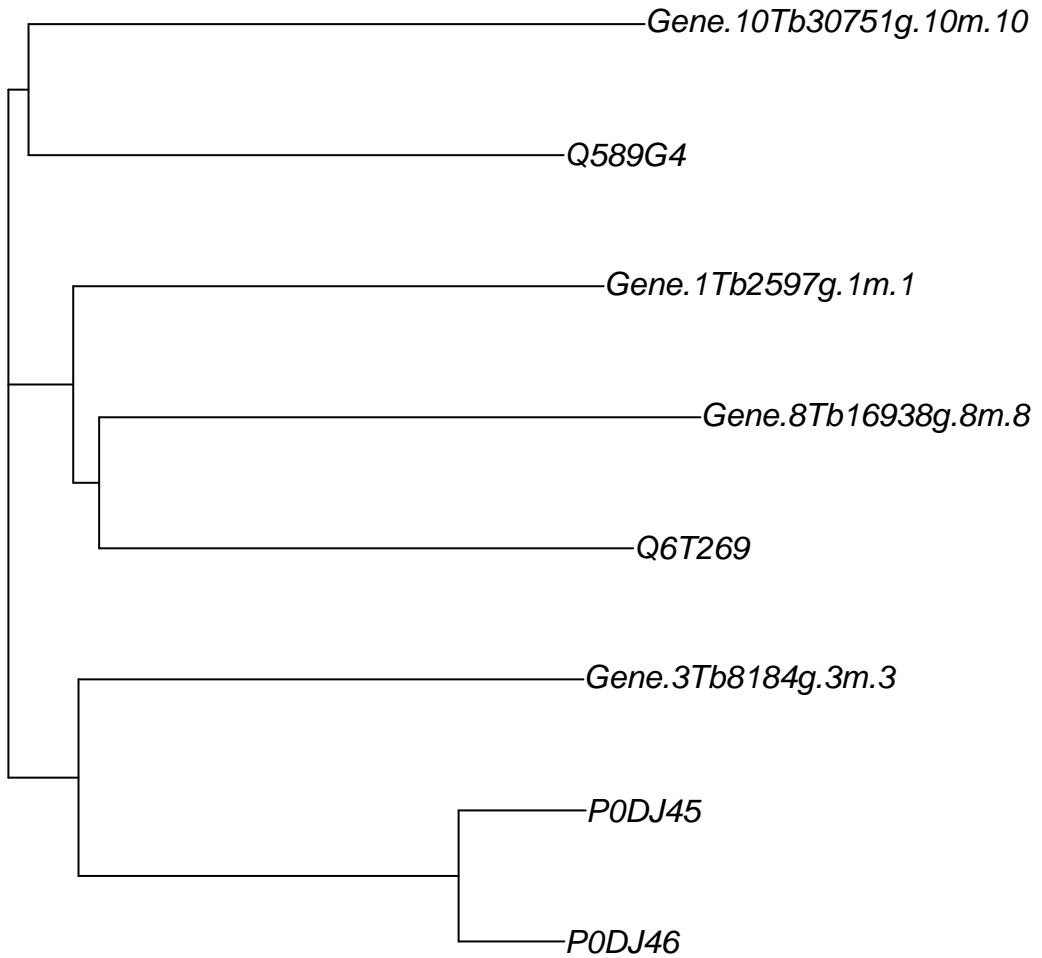

Supplement: Supplemental Information 5 [file peerj-06-5361-s005.gz › FinalOutput_GAS_1E-6/Kunitz-type_serine_protease_inhibitor_BmKTT-3_1/finaltree.pdf]

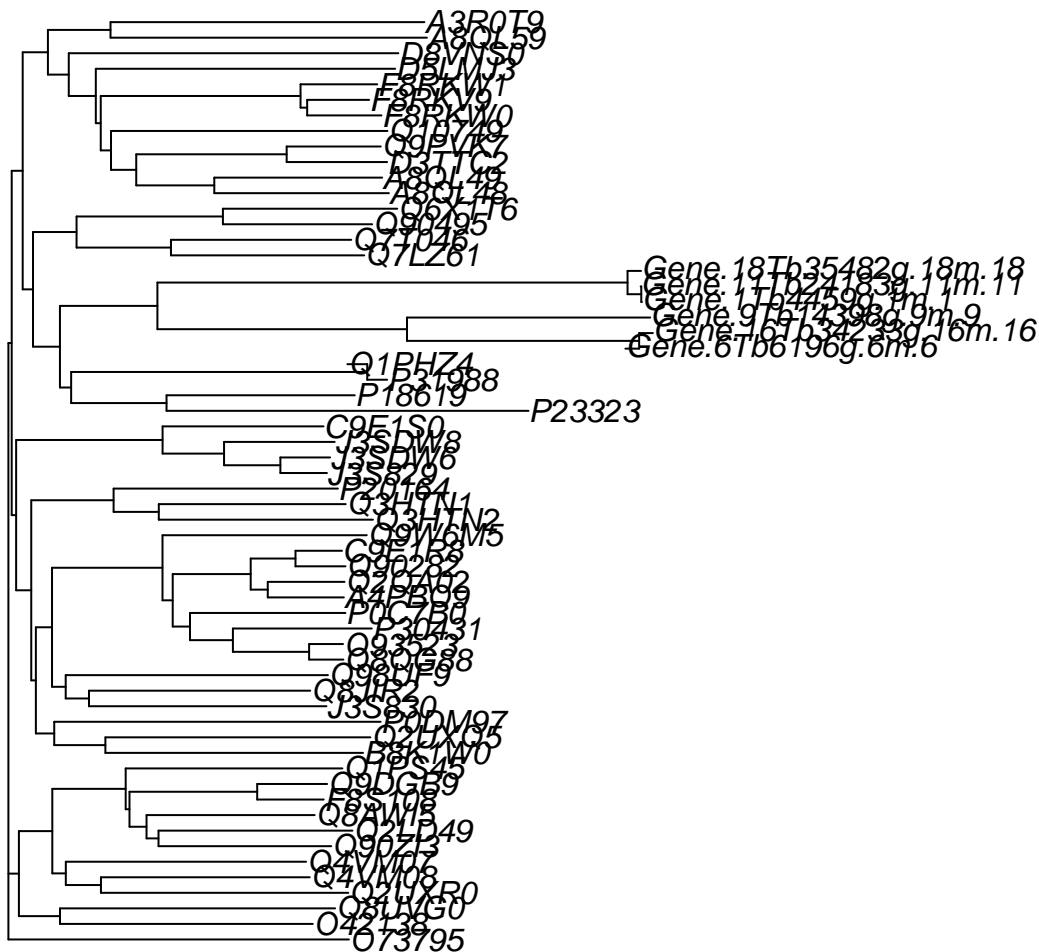

Supplement: Supplemental Information 5 [file peerj-06-5361-s005.gz › FinalOutput_GAS_1E-6/Zinc_metalloproteinase-disintegrin-like_mikarin_1/finaltree.pdf]

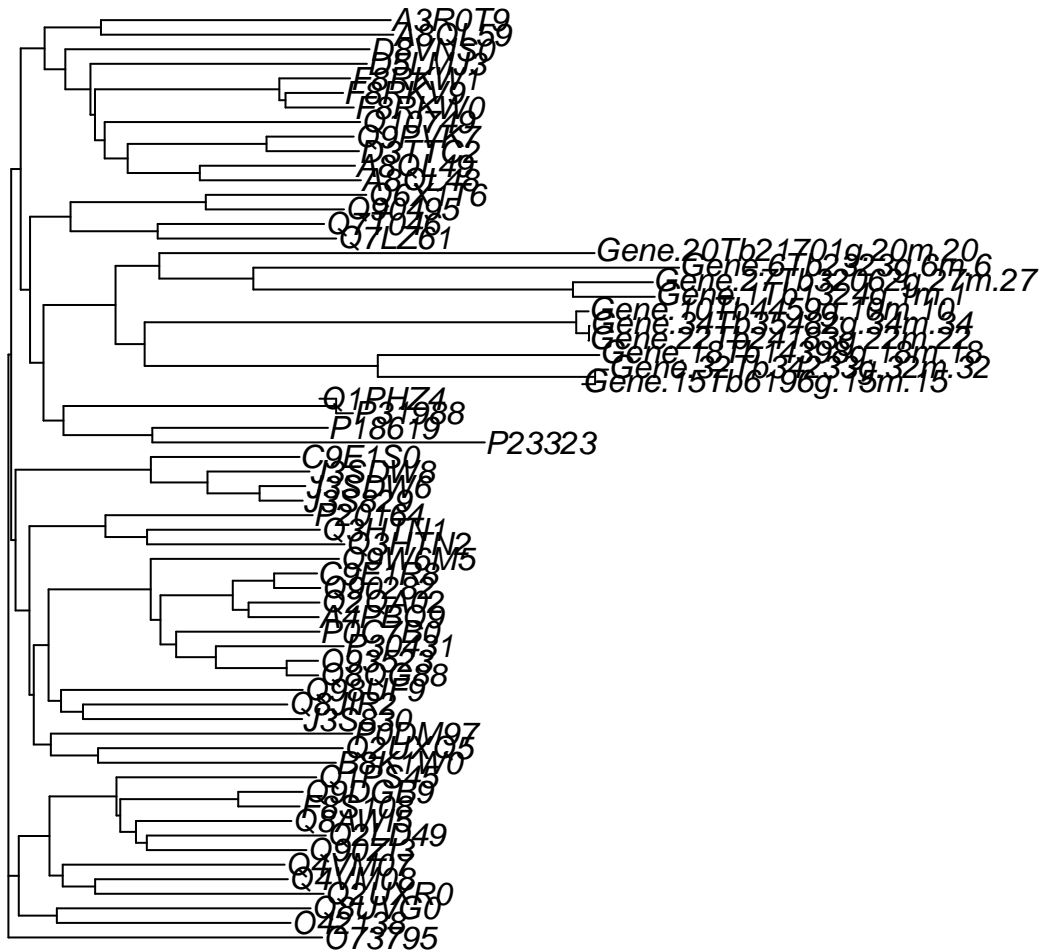

Supplement: Supplemental Information 5 [file peerj-06-5361-s005.gz › FinalOutput_GAS_1E-6/Zinc_metalloproteinase-disintegrin-like_VAP2B_272/finaltree.pdf]

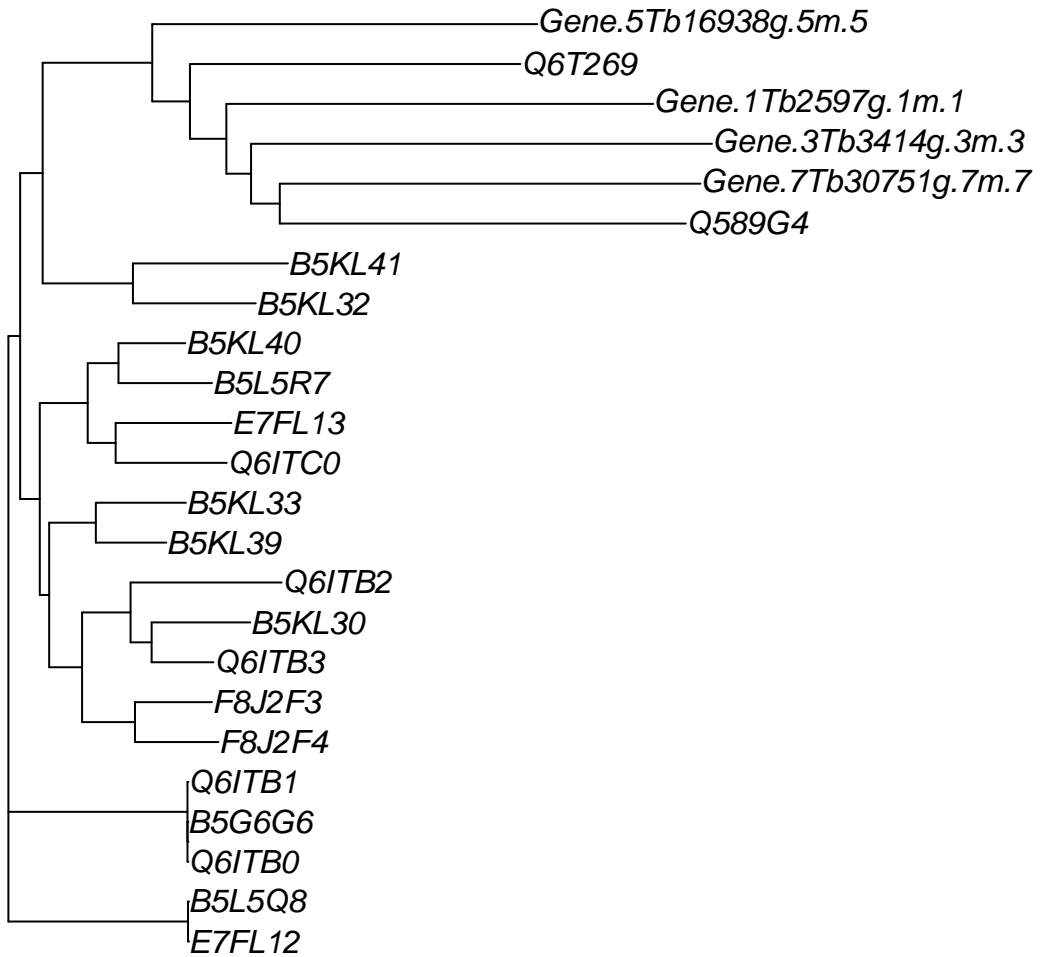

Supplement: Supplemental Information 5 [file peerj-06-5361-s005.gz › FinalOutput_GAS_1E-6/Kunitz-type_serine_protease_inhibitor_homolog_dendrotoxin_K_154/finaltree.pdf]

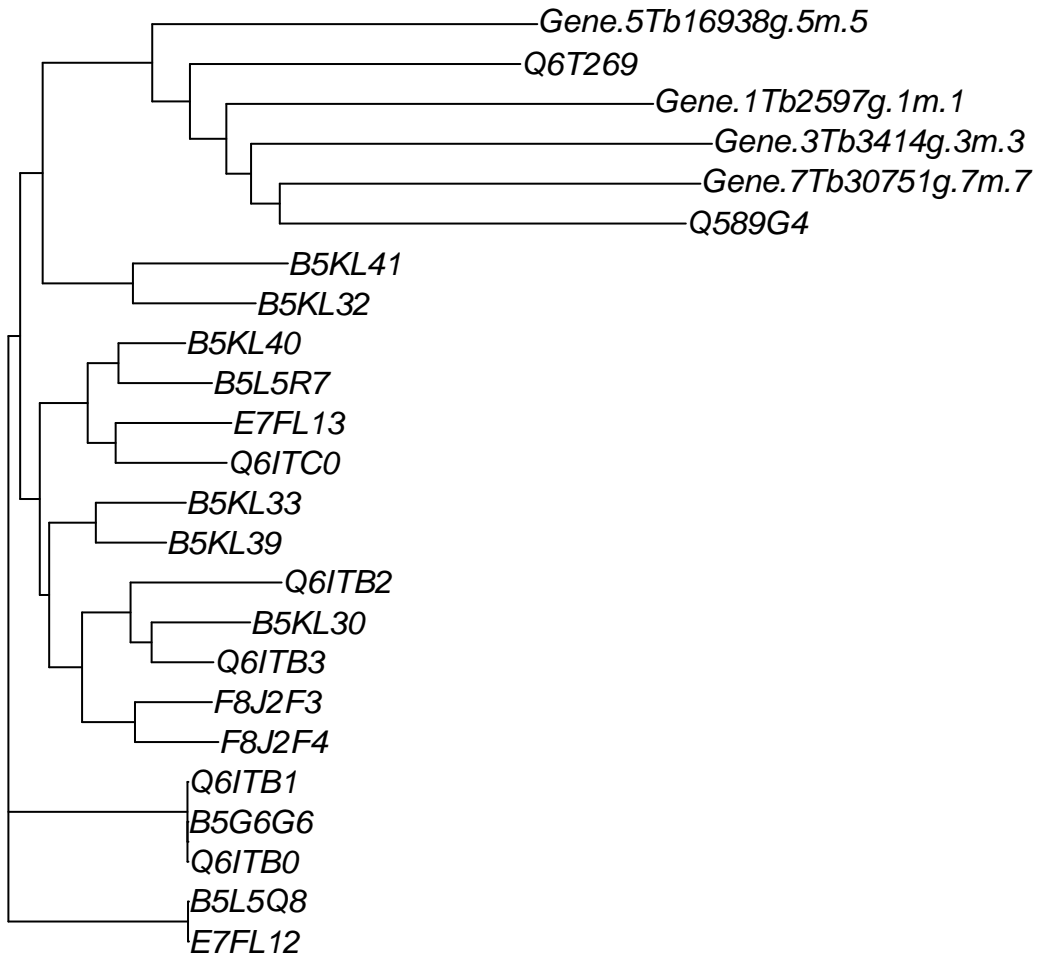

Supplement: Supplemental Information 5 [file peerj-06-5361-s005.gz › FinalOutput_GAS_1E-6/Kunitz-type_serine_protease_inhibitor_PIVL_13/finaltree.pdf]

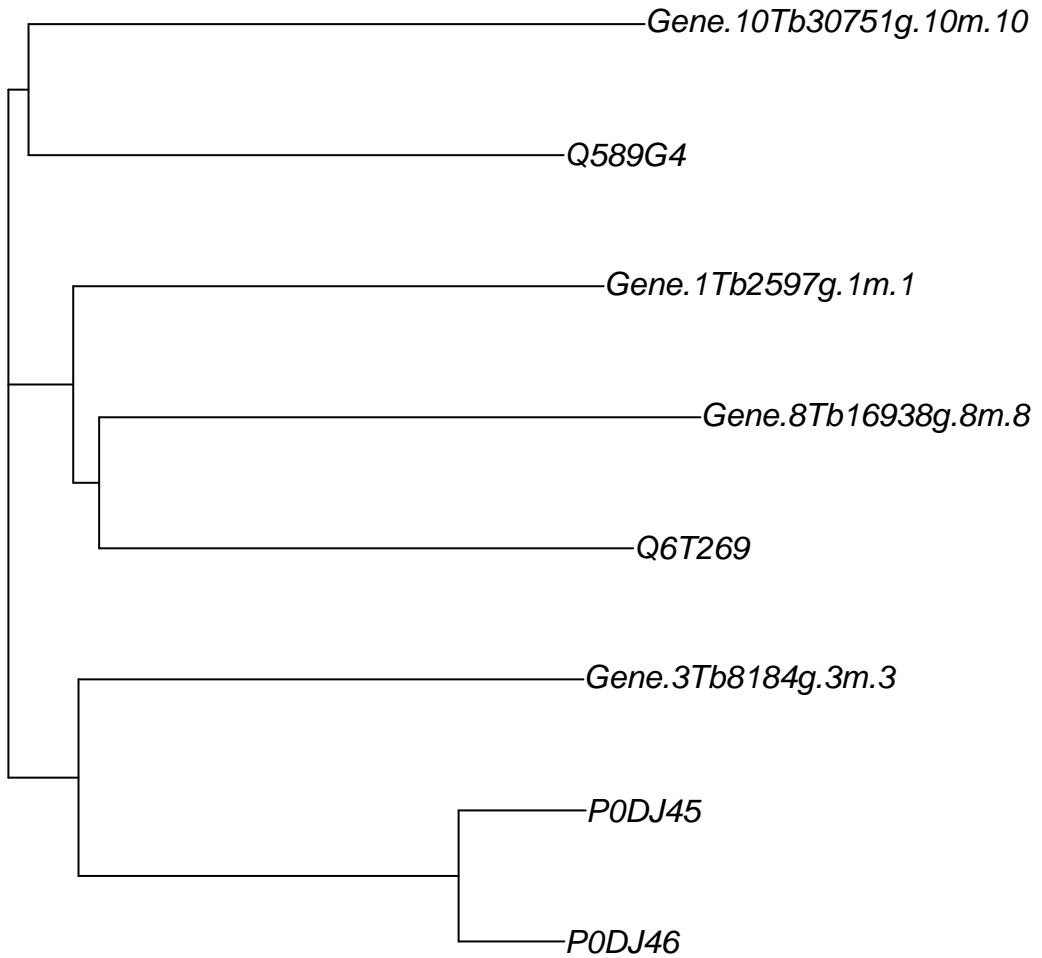

Supplement: Supplemental Information 5 [file peerj-06-5361-s005.gz › FinalOutput_GAS_1E-6/Kunitz-type_serine_protease_inhibitor_conotoxin_Cal9/finaltree.pdf]

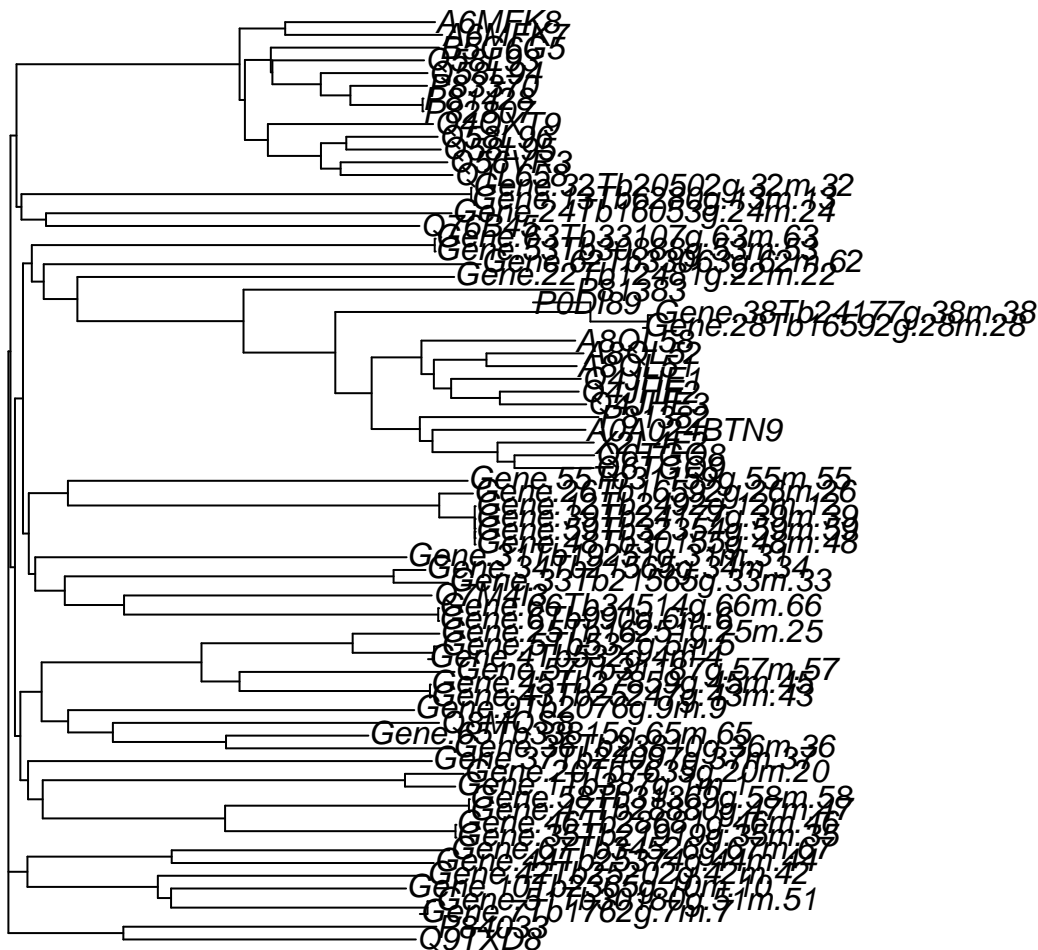

Supplement: Supplemental Information 5 [file peerj-06-5361-s005.gz › FinalOutput_GAS_1E-6/Factor_V_activator_RVV-V_gamma_142/finaltree.pdf]

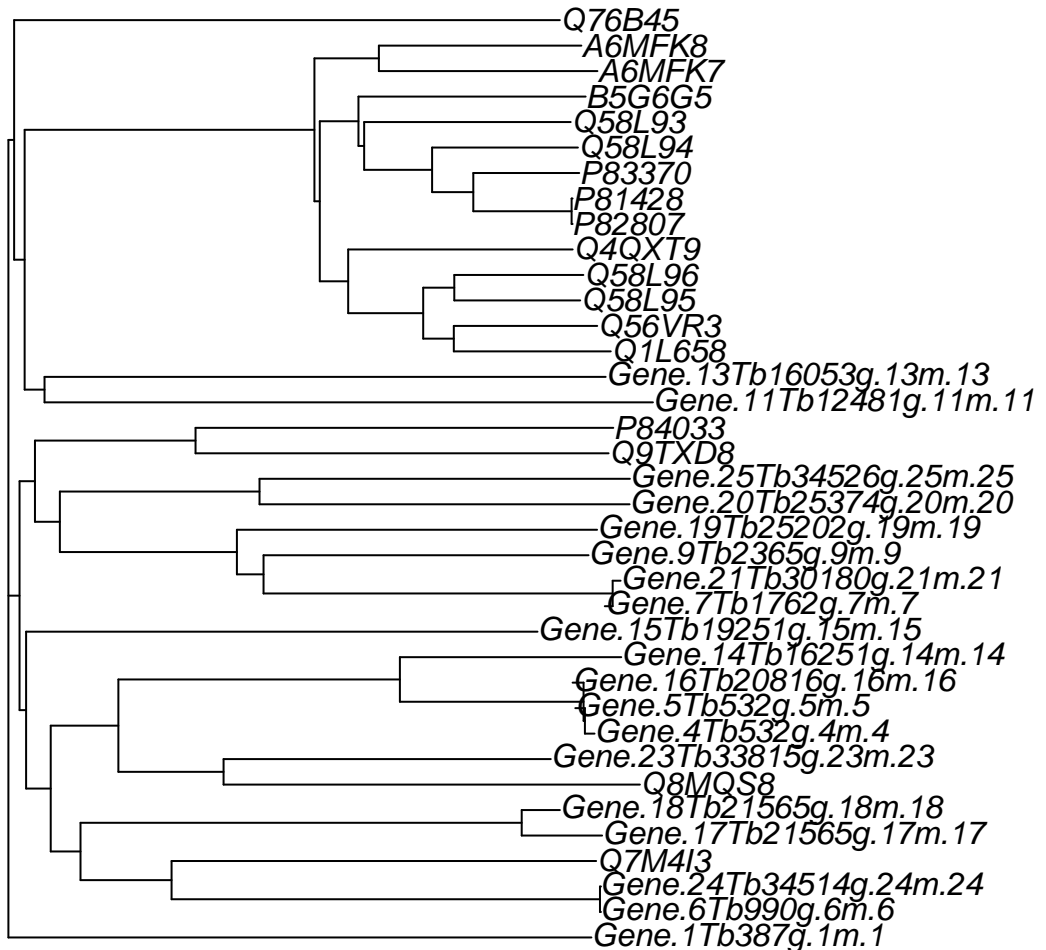

Supplement: Supplemental Information 5 [file peerj-06-5361-s005.gz › FinalOutput_GAS_1E-6/Thrombin-like_enzyme_TLBm_1/finaltree.pdf]

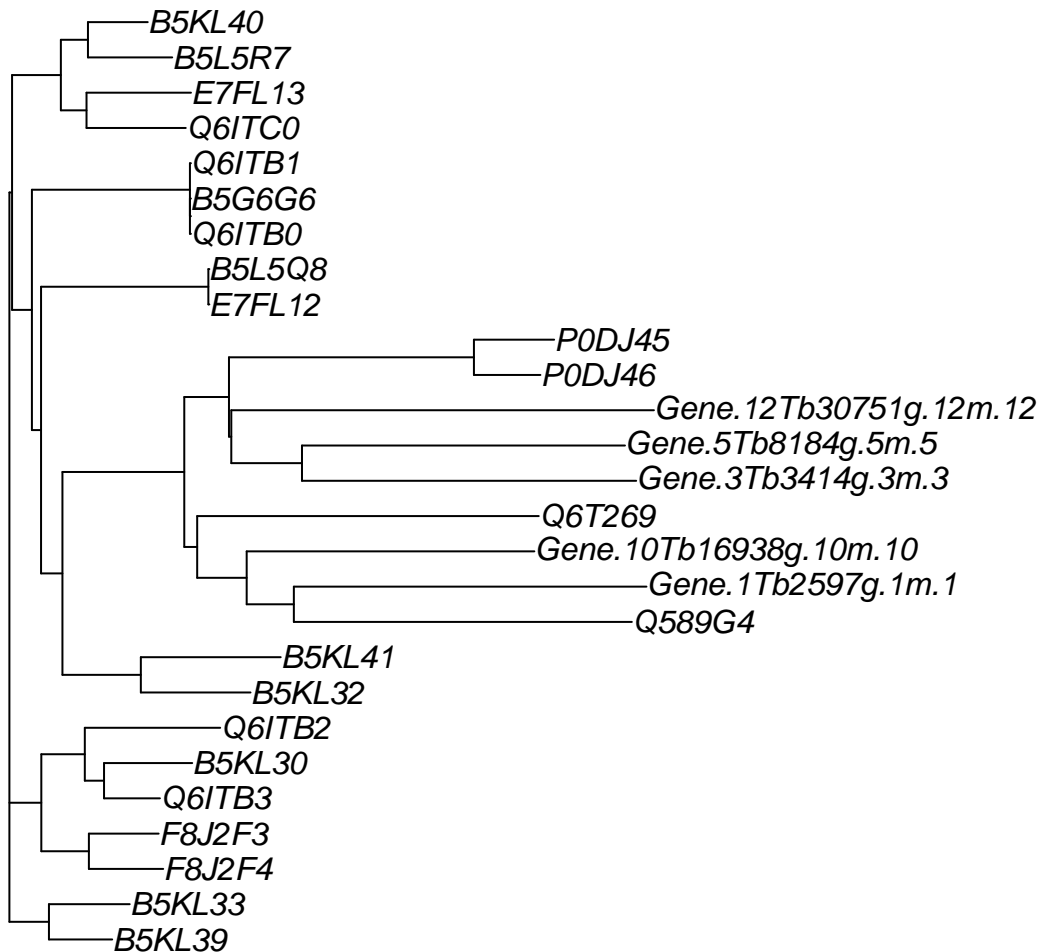

Supplement: Supplemental Information 5 [file peerj-06-5361-s005.gz › FinalOutput_GAS_1E-6/Protease_inhibitor_1_6/finaltree.pdf]

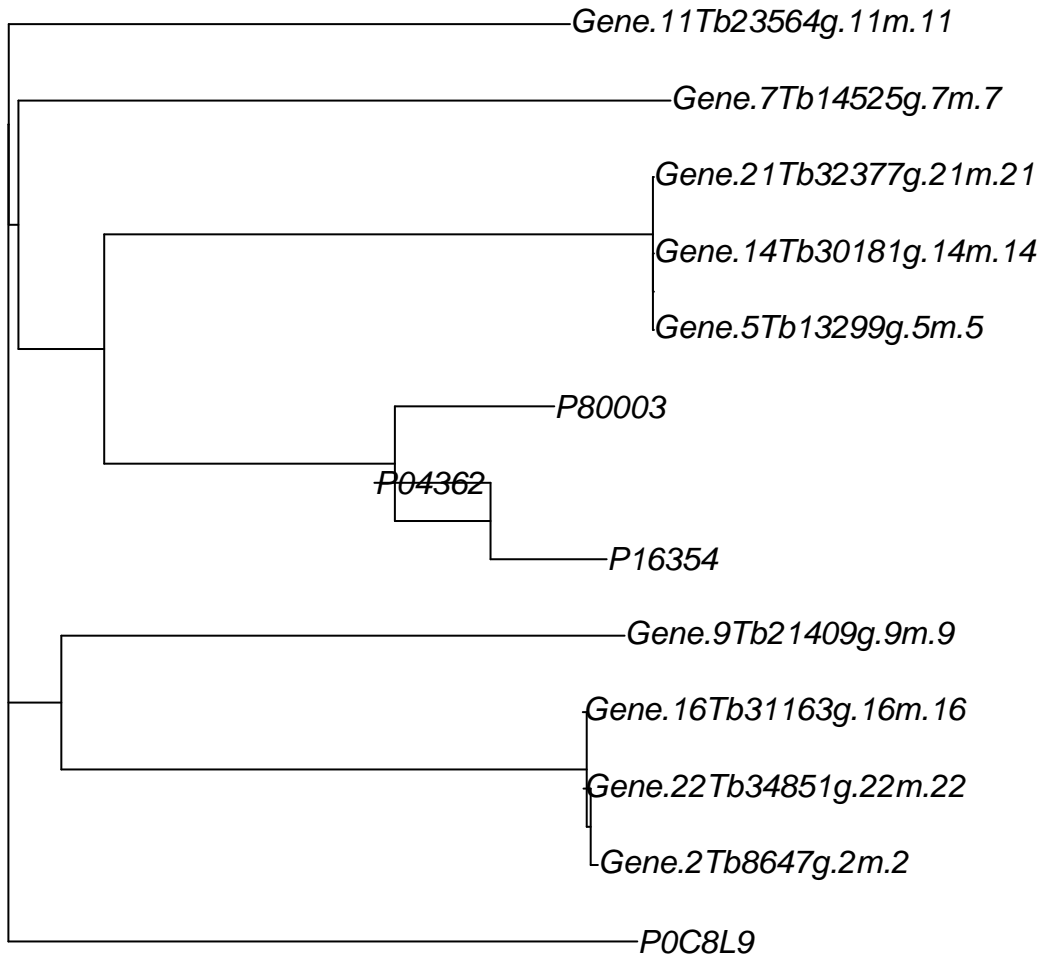

Supplement: Supplemental Information 5 [file peerj-06-5361-s005.gz › FinalOutput_GAS_1E-6/Phospholipase_A2_phaiodactylipin_6/finaltree.pdf]

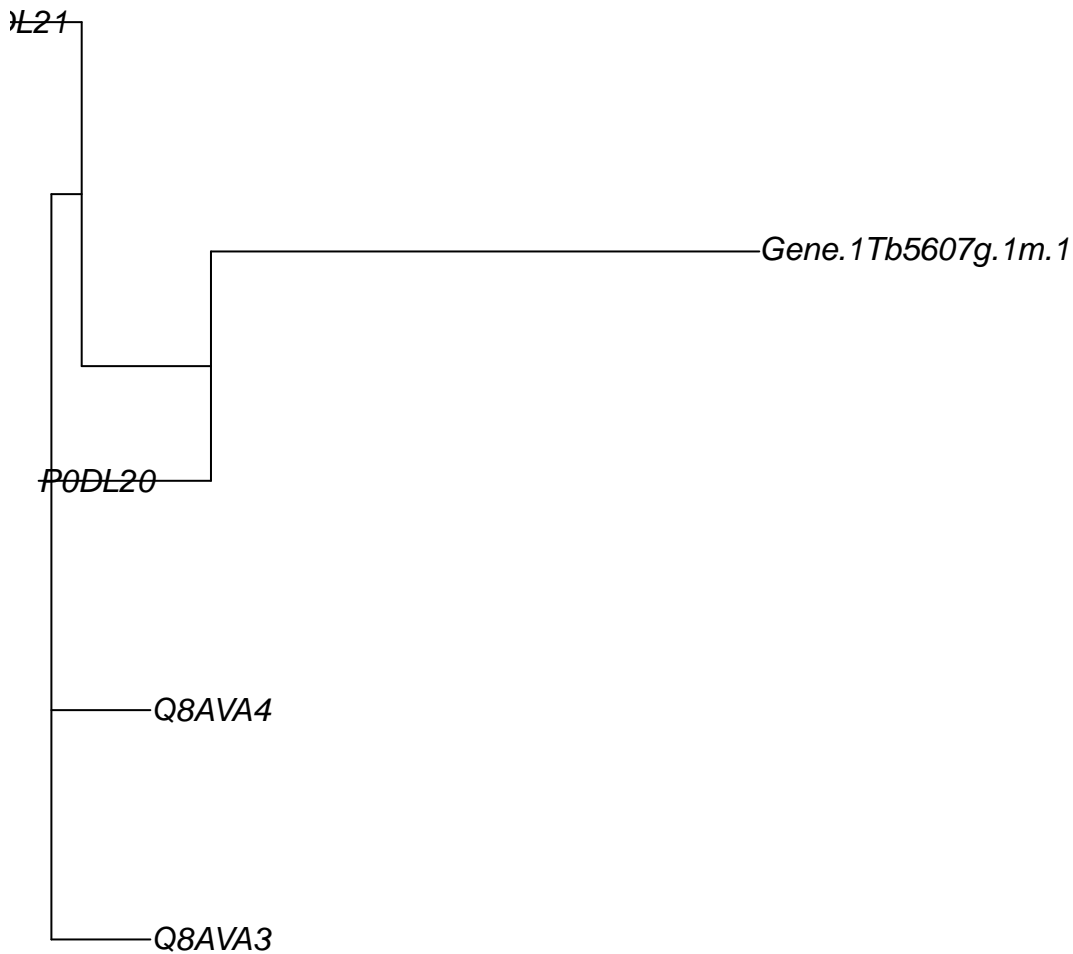

Supplement: Supplemental Information 5 [file peerj-06-5361-s005.gz › FinalOutput_GAS_1E-6/Cysteine-rich_venom_protein_bucarin_1/finaltree.pdf]

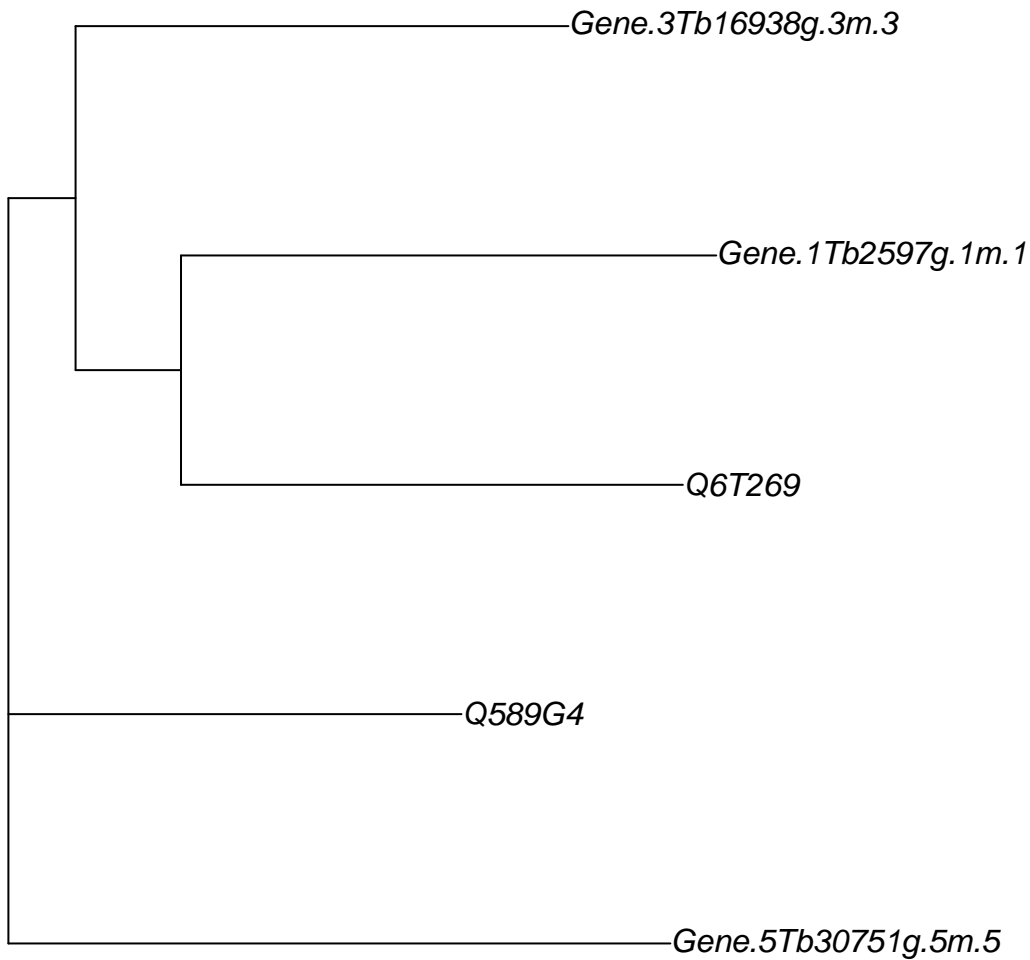

Supplement: Supplemental Information 5 [file peerj-06-5361-s005.gz › FinalOutput_GAS_1E-6/Kunitz-type_serine_protease_inhibitor_homolog_alpha-dendrotoxin_3/finaltree.pdf]

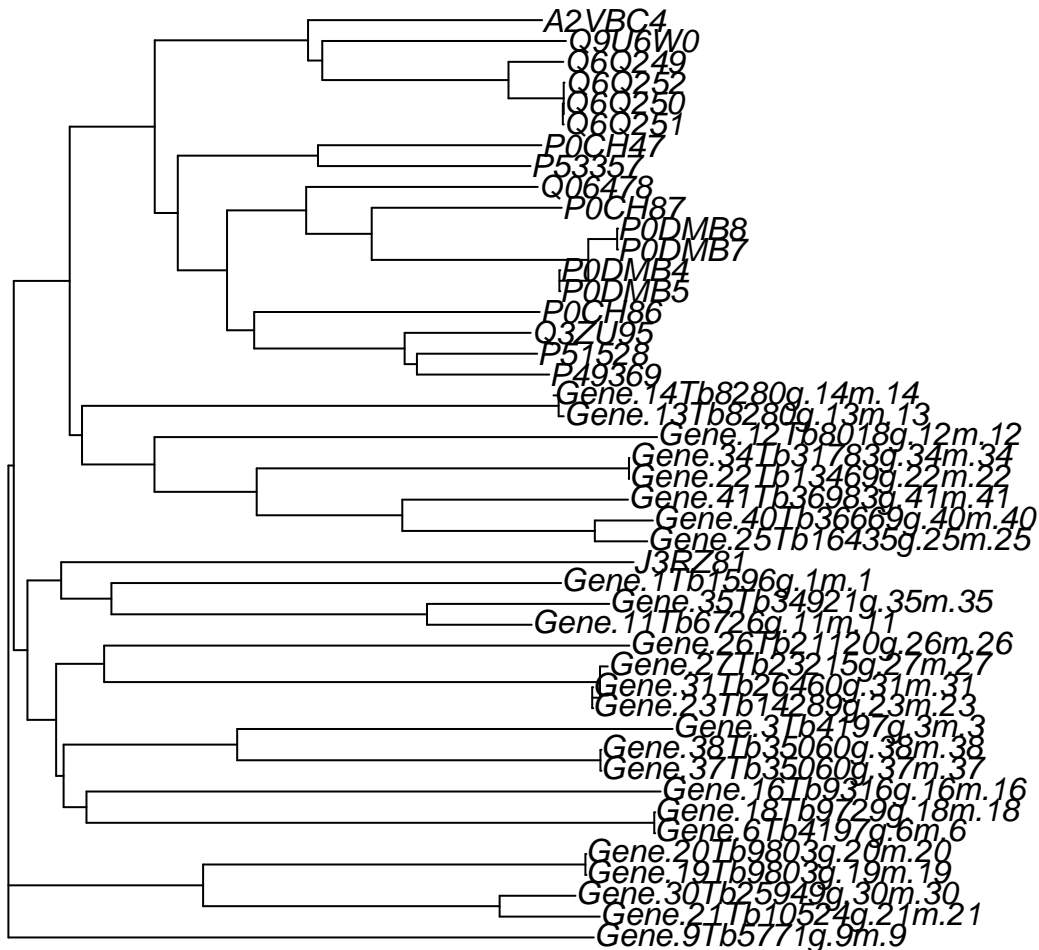

Supplement: Supplemental Information 5 [file peerj-06-5361-s005.gz › FinalOutput_GAS_1E-6/Phospholipase_A1_19/finaltree.pdf]

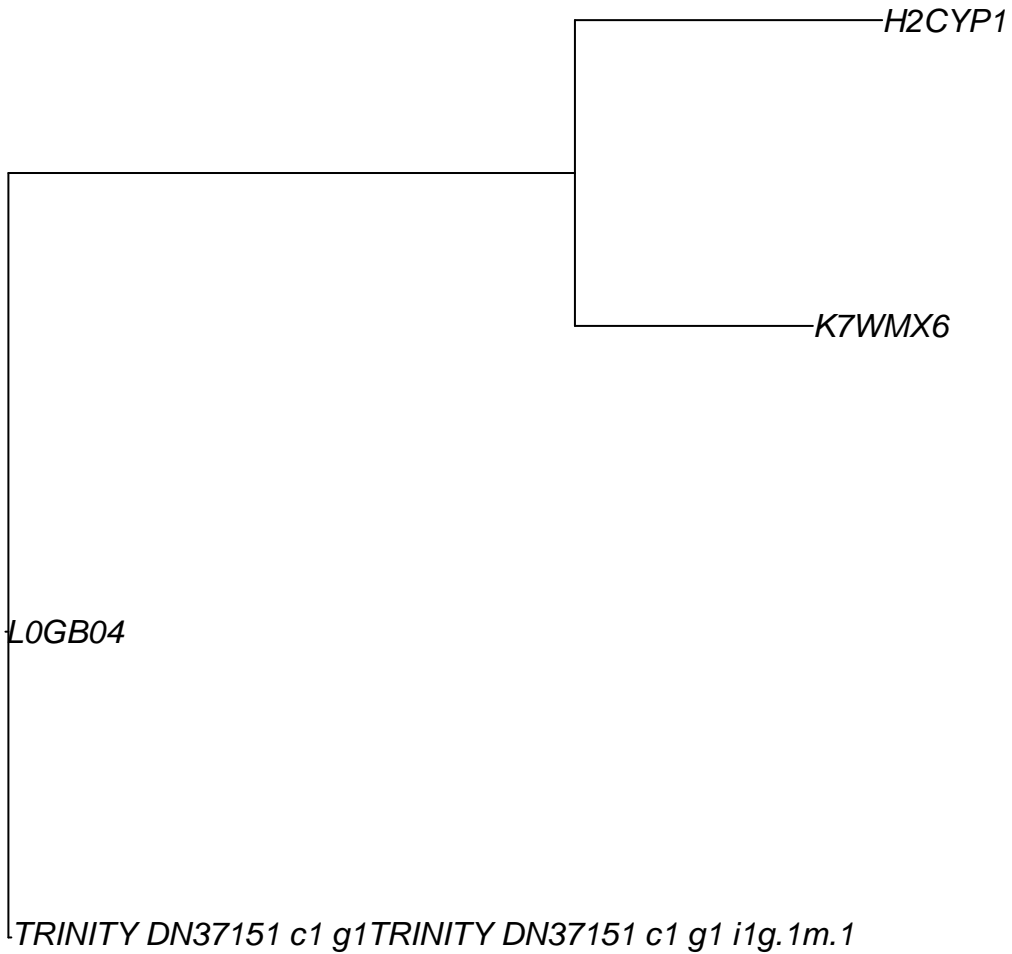

Supplement: Supplemental Information 6 [file peerj-06-5361-s006.gz › FinalOutput_1E-20/La1-like_protein_15_3/finaltree.pdf]

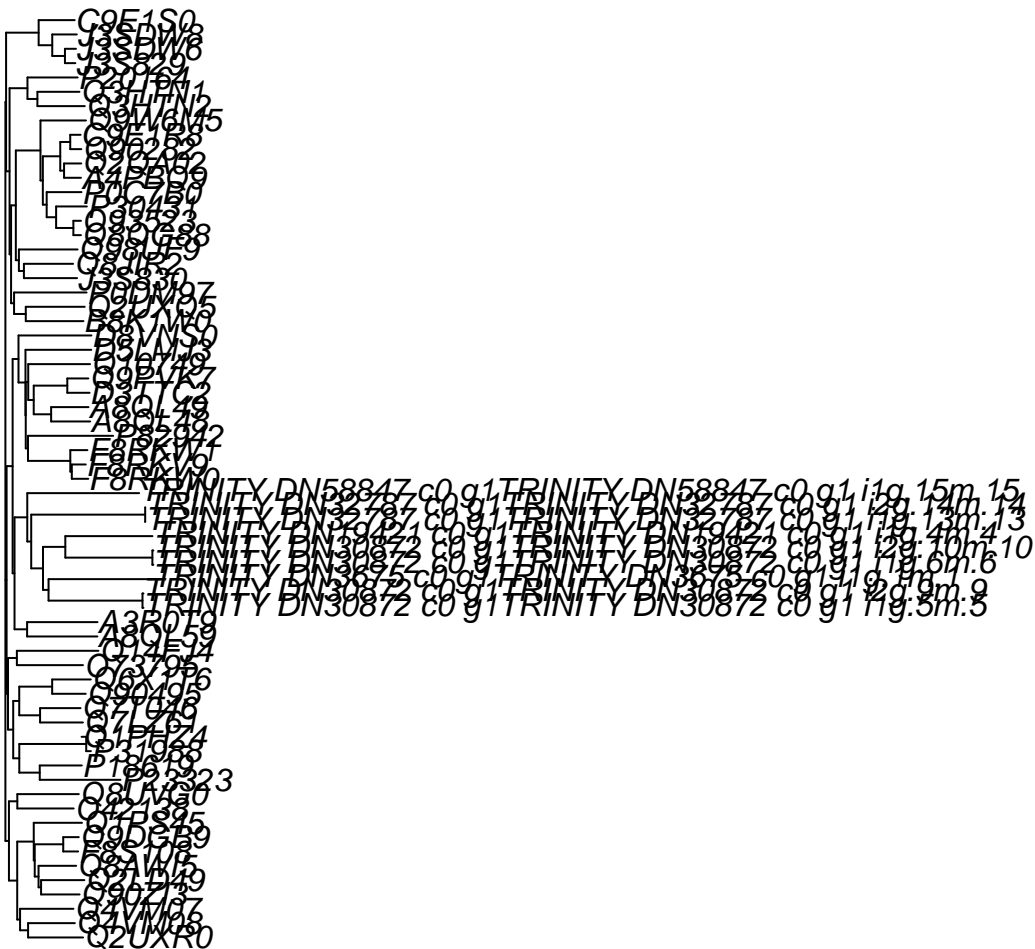

Supplement: Supplemental Information 6 [file peerj-06-5361-s006.gz › FinalOutput_1E-20/Zinc_metalloproteinase-disintegrin-like_VAP2B_272/finaltree.pdf]

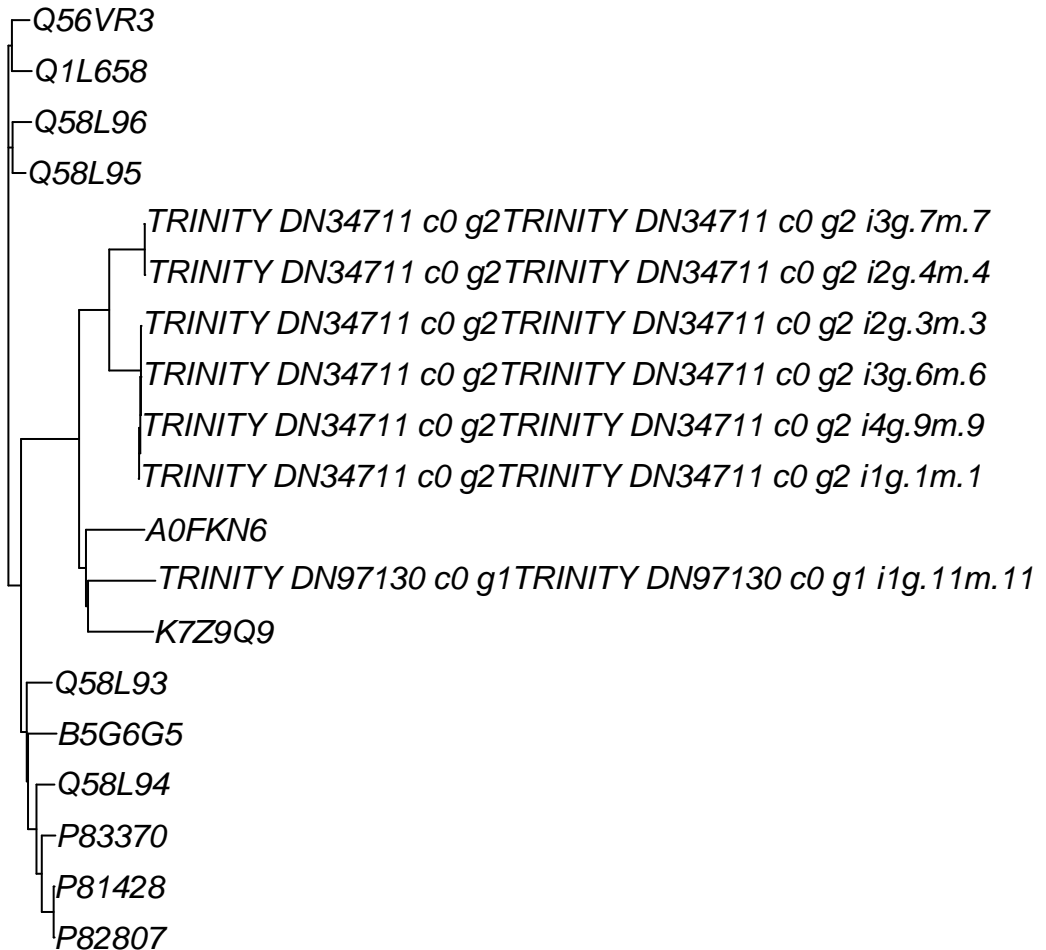

Supplement: Supplemental Information 6 [file peerj-06-5361-s006.gz › FinalOutput_1E-20/Astacin-like_metalloprotease_toxin_4_1/finaltree.pdf]

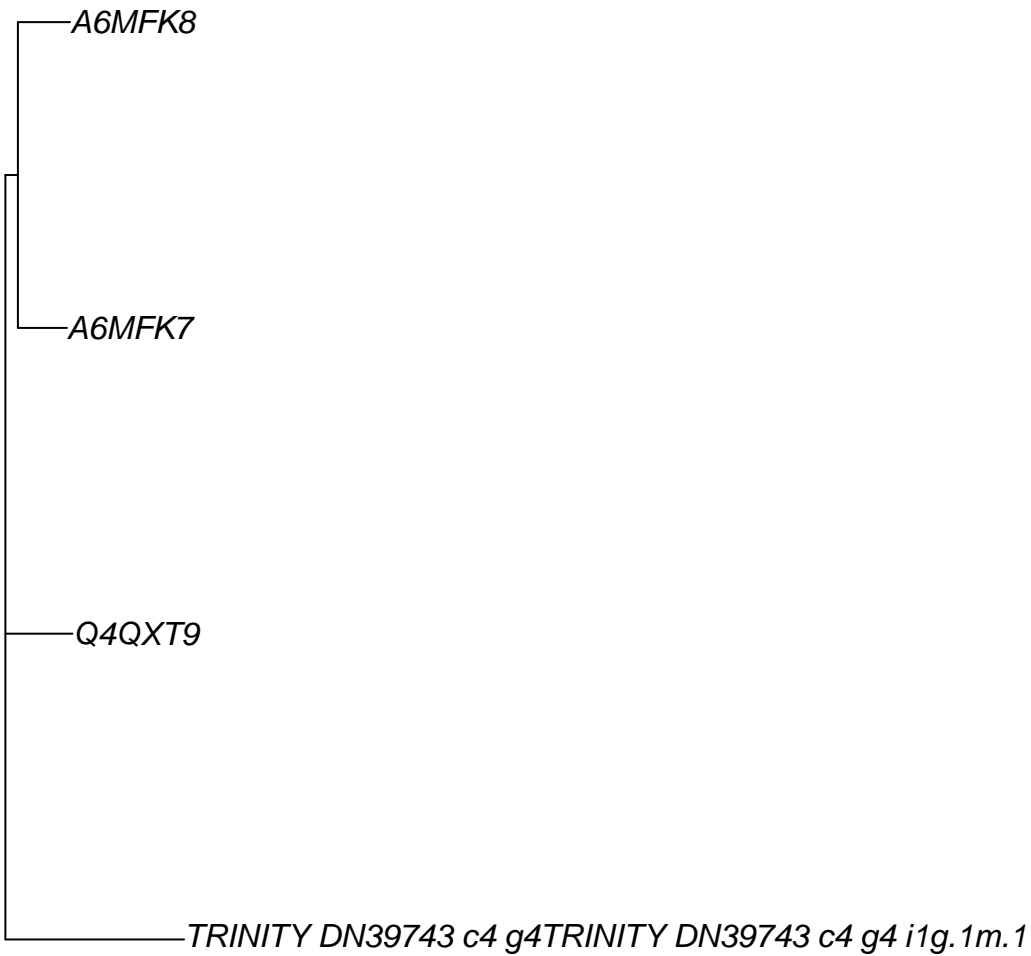

Supplement: Supplemental Information 6 [file peerj-06-5361-s006.gz › FinalOutput_1E-20/Factor_V_activator_RVV-V_gamma_142/finaltree.pdf]

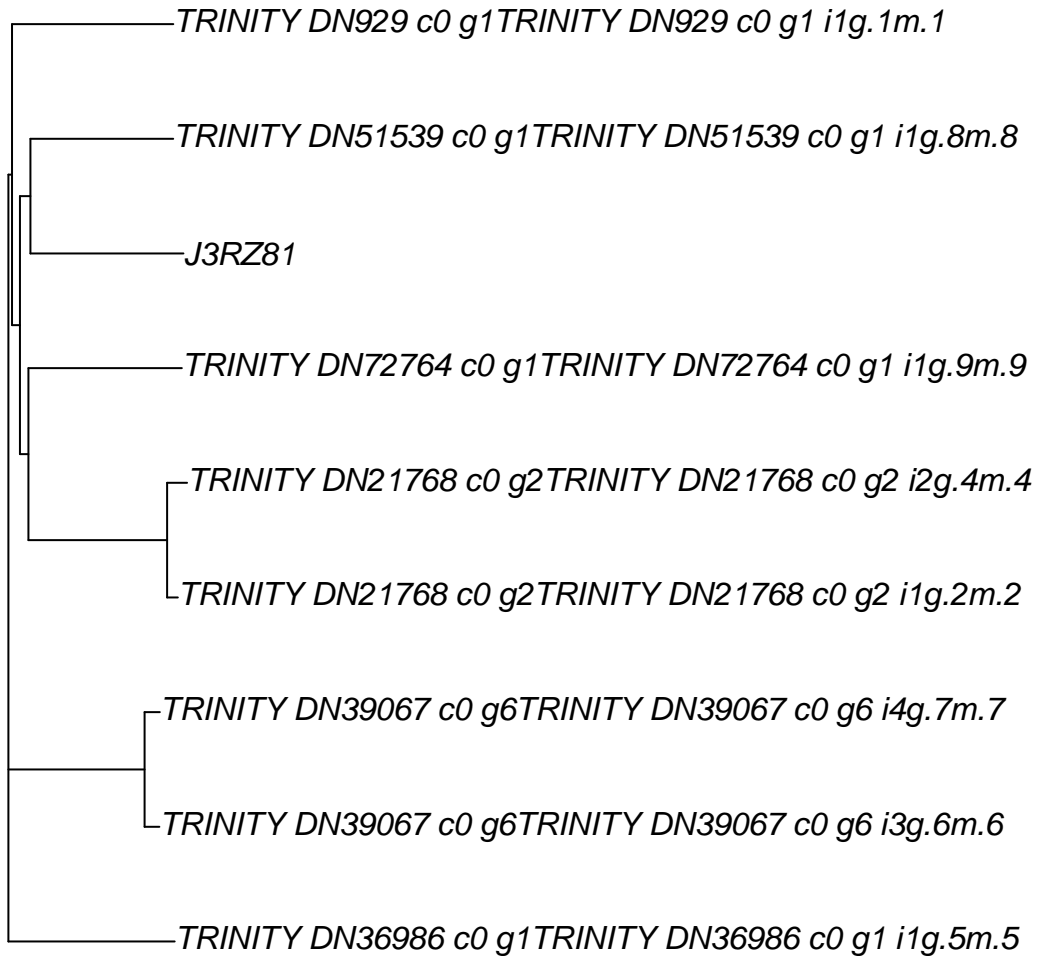

Supplement: Supplemental Information 6 [file peerj-06-5361-s006.gz › FinalOutput_1E-20/Phospholipase_A1_19/finaltree.pdf]

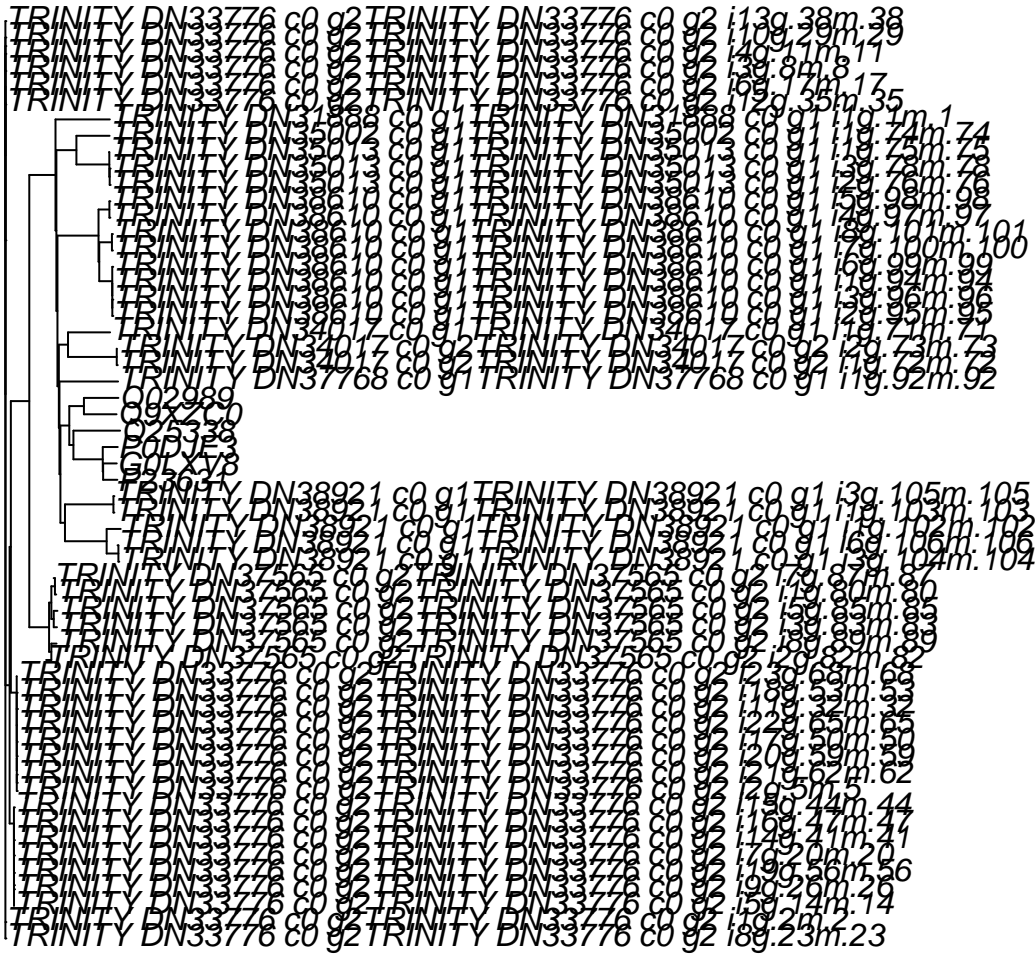

Supplement: Supplemental Information 6 [file peerj-06-5361-s006.gz › FinalOutput_1E-20/Alpha-latroinsectotoxin-Lt1a_2/finaltree.pdf]

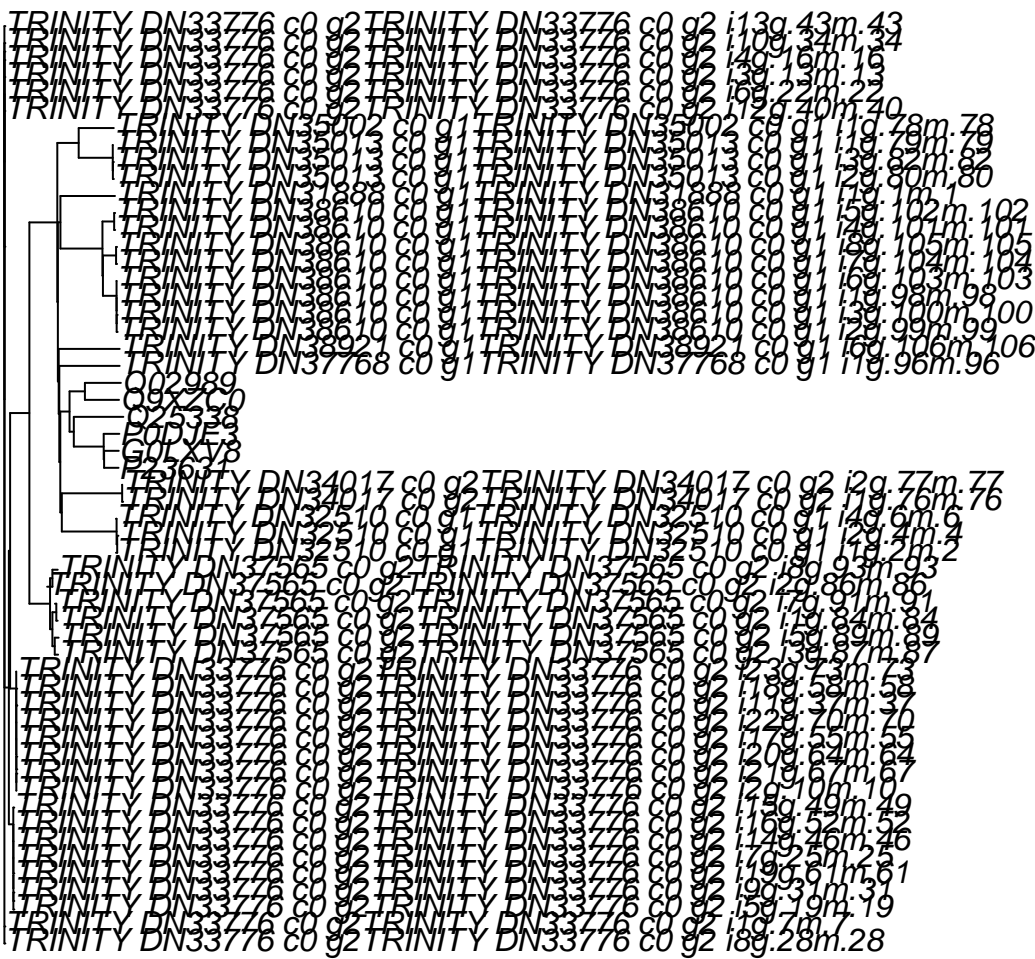

Supplement: Supplemental Information 6 [file peerj-06-5361-s006.gz › FinalOutput_1E-20/Delta-latroinsectotoxin-Lt1a_1/finaltree.pdf]

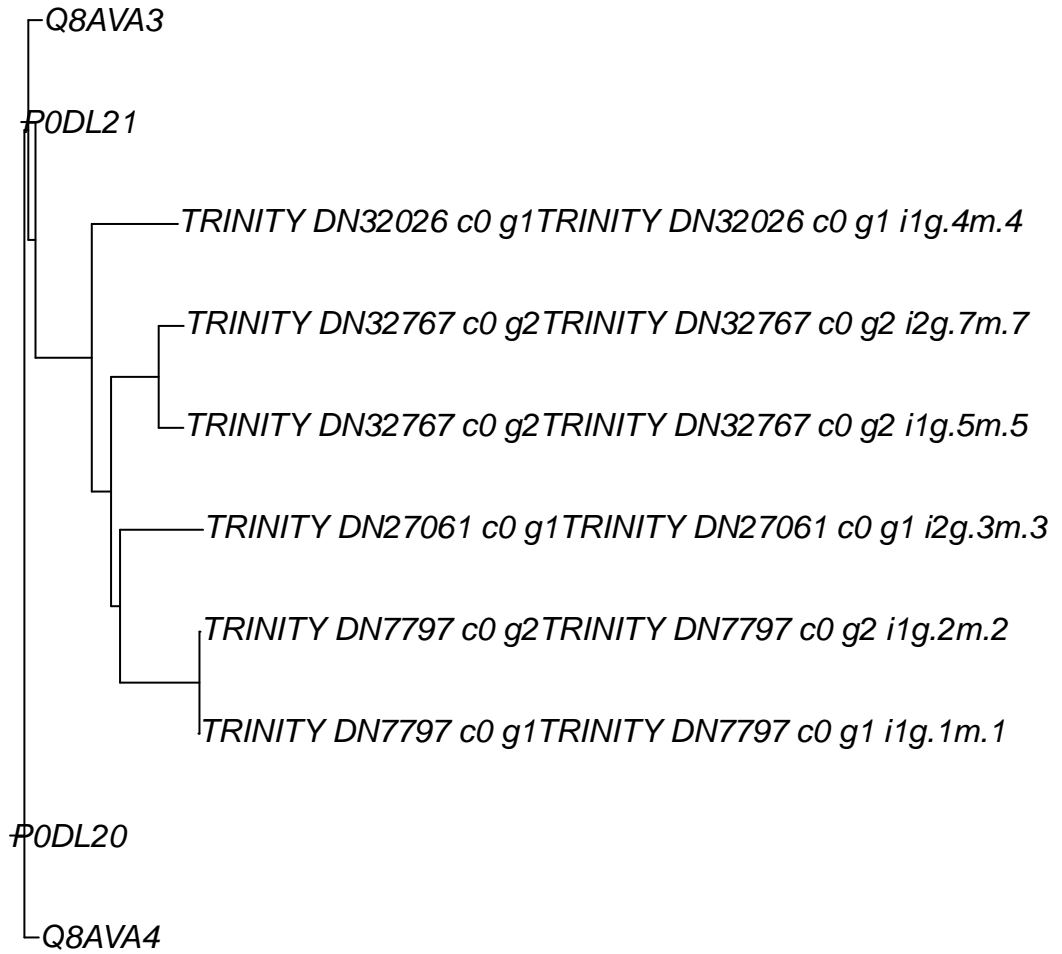

Supplement: Supplemental Information 6 [file peerj-06-5361-s006.gz › FinalOutput_1E-20/Cysteine-rich_venom_protein_Mr30_2/finaltree.pdf]
