# Supplementary material for: Venomix: a simple bioinformatic pipeline for identifying and characterizing toxin gene candidates from transcriptomic data
Source: PeerJ. 2018 Jul 31;6:e5361. doi: 10.7717/peerj.5361 (PMC6074769; doi:10.7717/peerj.5361)
Supplement: Supplemental Information 3 [file peerj-06-5361-s003.gz › FinalOutput_E-20/Astacin-like_metalloprotease_toxin_4_1/finaltree.pdf]

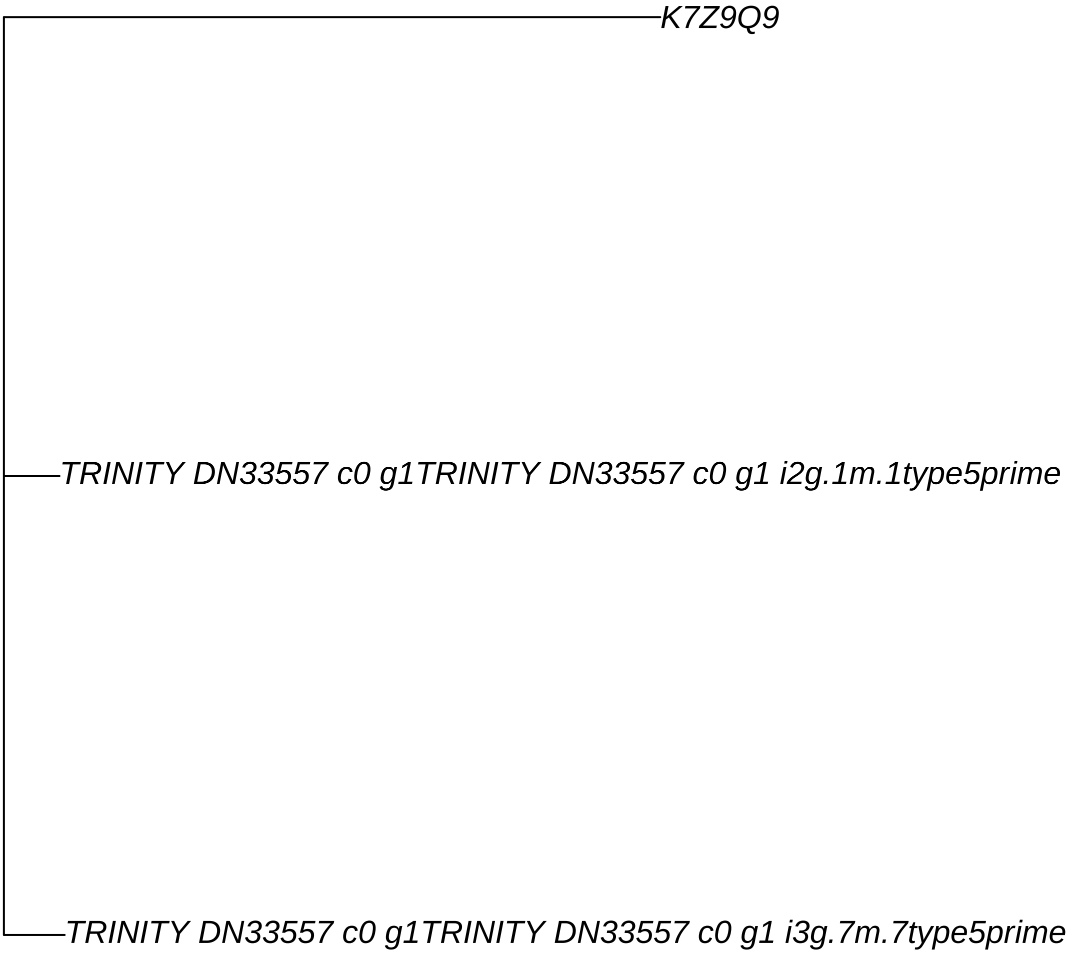
K7Z9Q9
TRINITY DN33557 c0 g1TRINITY DN33557 c0 g1 i2g.1m.1type5prime
TRINITY DN33557 c0 g1TRINITY DN33557 c0 g1 i3g.7m.7type5prime
