# Supplementary material for: Venomix: a simple bioinformatic pipeline for identifying and characterizing toxin gene candidates from transcriptomic data
Source: PeerJ. 2018 Jul 31;6:e5361. doi: 10.7717/peerj.5361 (PMC6074769; doi:10.7717/peerj.5361)
Supplement: Supplemental Information 4 [file peerj-06-5361-s004.gz › FinalOutput_E-20/Cysteine-rich_venom_protein_Mr30_2/finaltree.pdf]

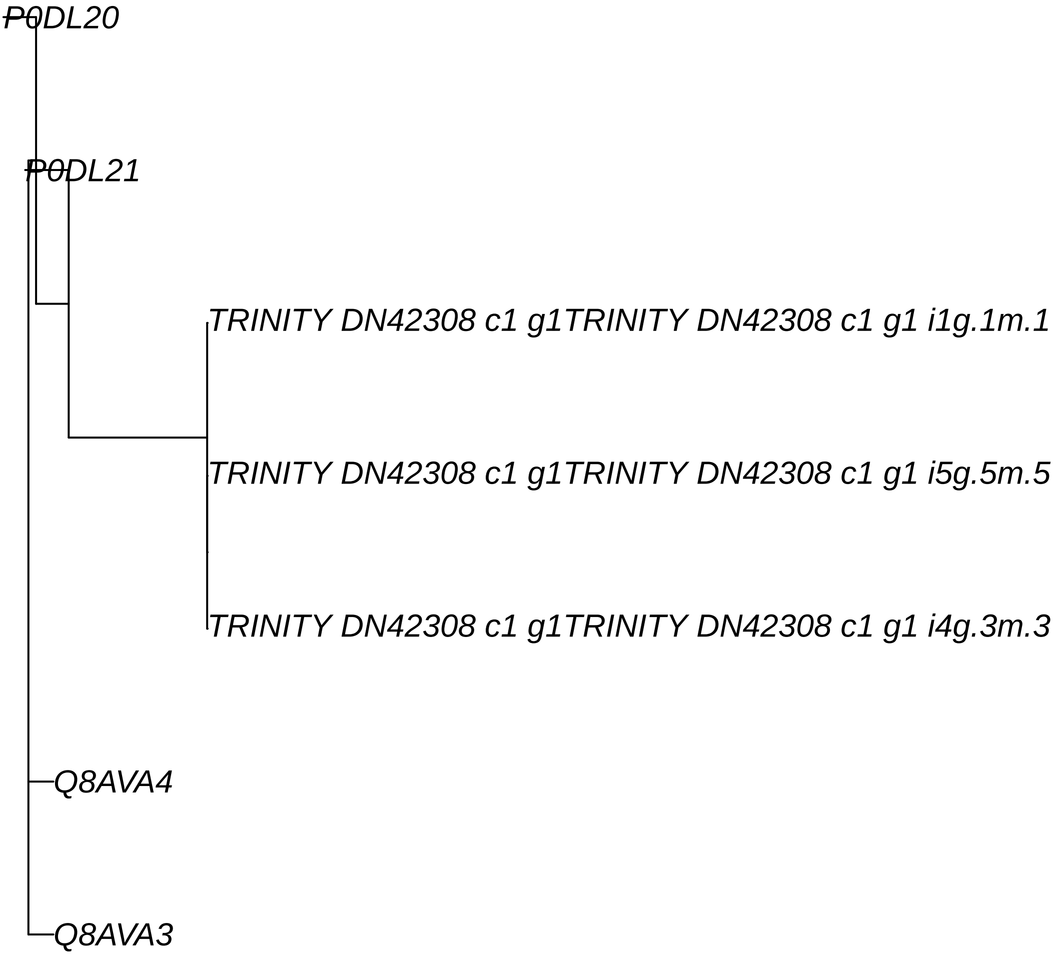
P0DL20
P0DL21
TRINITY DN42308 c1 g1TRINITY DN42308 c1 g1 i1g.1m.1
TRINITY DN42308 c1 g1TRINITY DN42308 c1 g1 i5g.5m.5
TRINITY DN42308 c1 g1TRINITY DN42308 c1 g1 i4g.3m.3
Q8AVA4
Q8AVA3
