# Supplementary material for: Venomix: a simple bioinformatic pipeline for identifying and characterizing toxin gene candidates from transcriptomic data
Source: PeerJ. 2018 Jul 31;6:e5361. doi: 10.7717/peerj.5361 (PMC6074769; doi:10.7717/peerj.5361)
Supplement: Supplemental Information 4 [file peerj-06-5361-s004.gz › FinalOutput_E-20/Hyaluronidase_conohyal-ad1_1/finaltree.pdf]

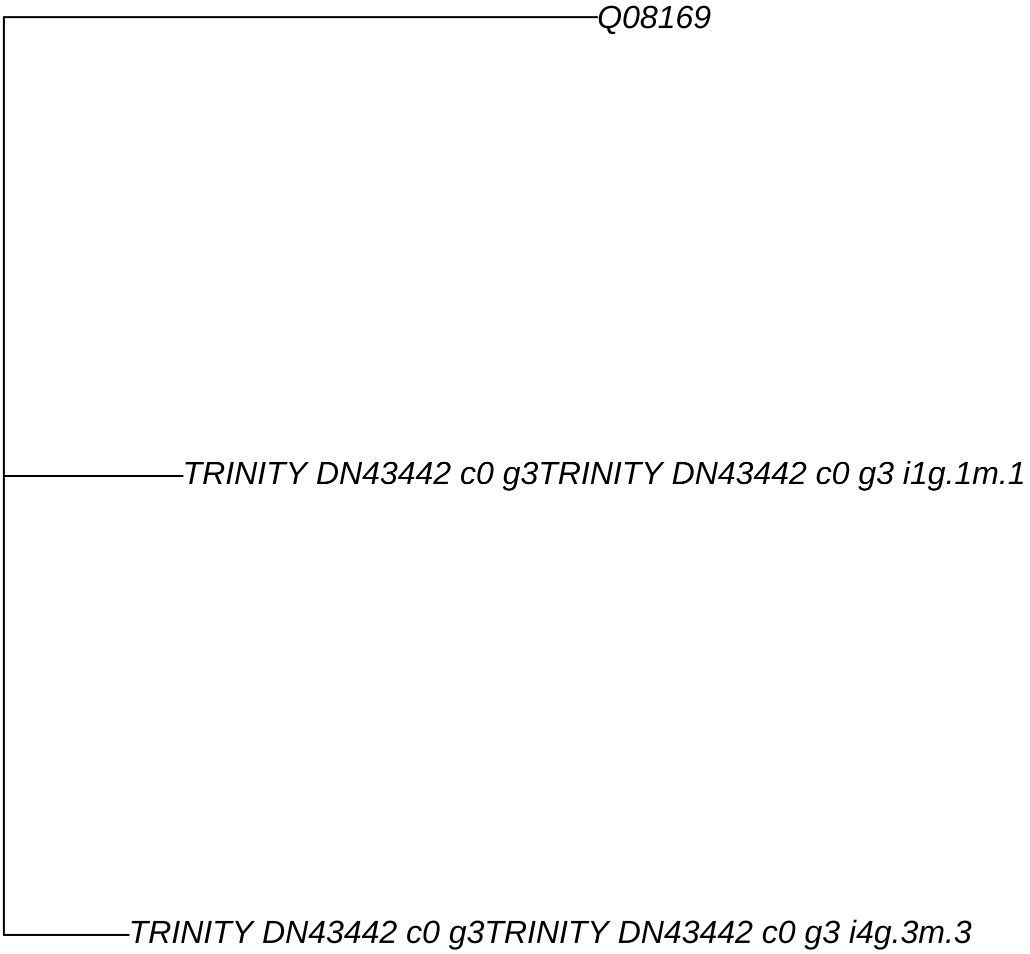
Q08169
TRINITY DN43442 c0 g3TRINITY DN43442 c0 g3 i1g.1m.1
TRINITY DN43442 c0 g3TRINITY DN43442 c0 g3 i4g.3m.3
