# Supplementary material for: Venomix: a simple bioinformatic pipeline for identifying and characterizing toxin gene candidates from transcriptomic data
Source: PeerJ. 2018 Jul 31;6:e5361. doi: 10.7717/peerj.5361 (PMC6074769; doi:10.7717/peerj.5361)
Supplement: Supplemental Information 6 [file peerj-06-5361-s006.gz › FinalOutput_1E-20/Phospholipase_D_StSicTox-betaIF1_2/finaltree.pdf]

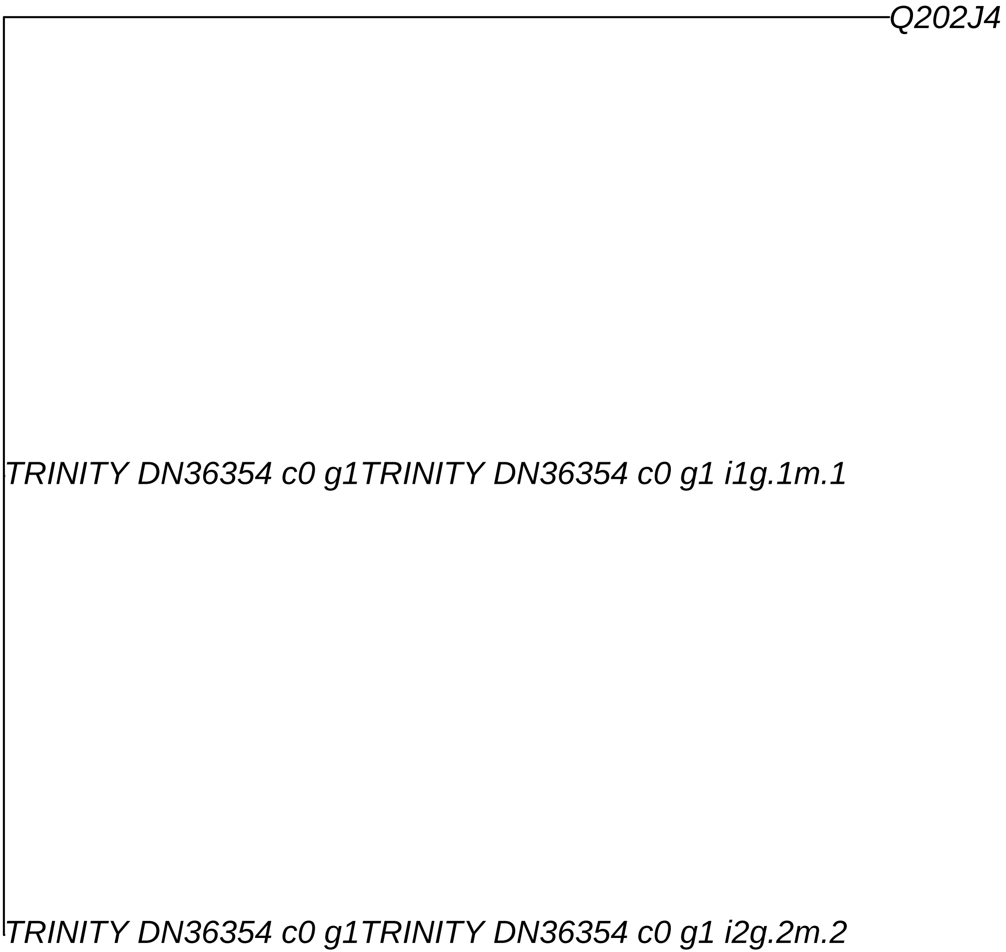

Q202J4
TRINITY DN36354 c0 g1TRINITY DN36354 c0 g1 i1g.1m.1
TRINITY DN36354 c0 g1TRINITY DN36354 c0 g1 i2g.2m.2
