# Supplementary material for: Venomix: a simple bioinformatic pipeline for identifying and characterizing toxin gene candidates from transcriptomic data
Source: PeerJ. 2018 Jul 31;6:e5361. doi: 10.7717/peerj.5361 (PMC6074769; doi:10.7717/peerj.5361)
Supplement: Supplemental Information 2 [file peerj-06-5361-s002.gz › FinalOutput_1E-20/Neprilysin-1_1/finaltree.pdf]

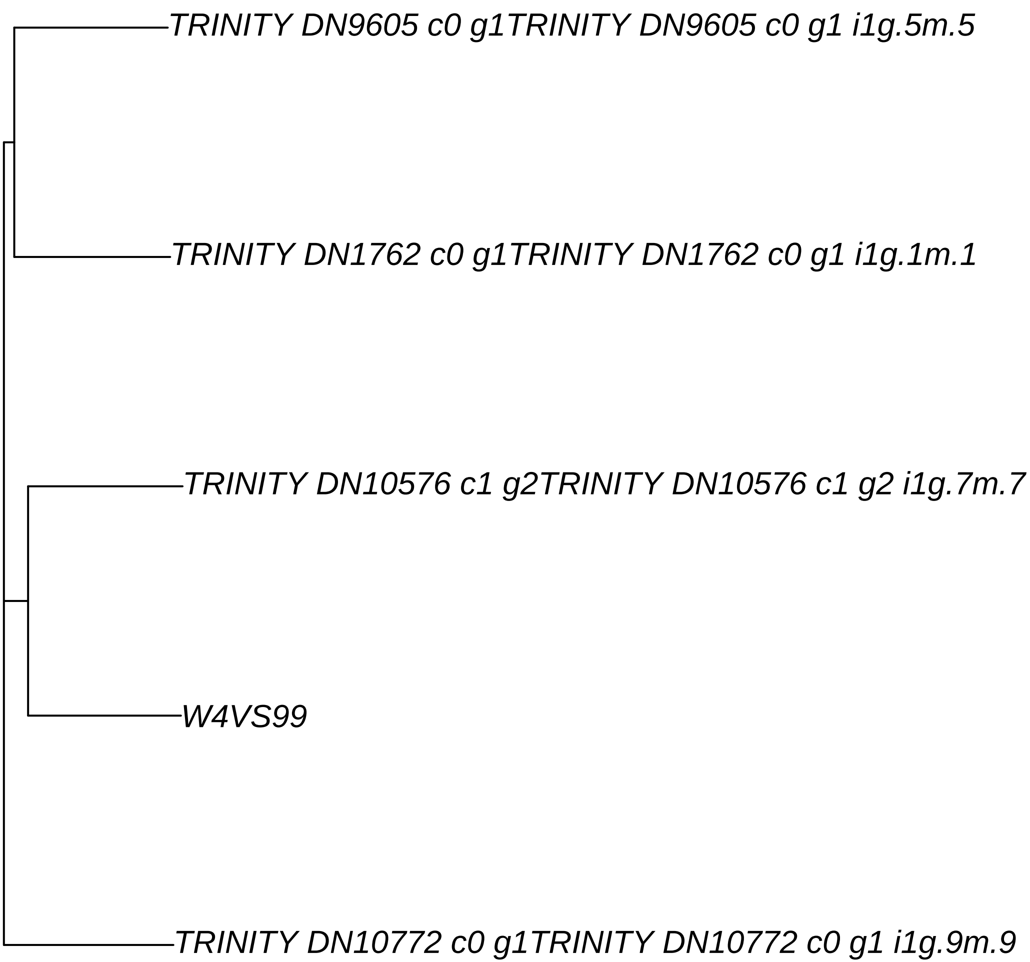

TRINITY DN9605 c0 g1TRINITY DN9605 c0 g1 i1g.5m.5
TRINITY DN1762 c0 g1TRINITY DN1762 c0 g1 i1g.1m.1
TRINITY DN10576 c1 g2TRINITY DN10576 c1 g2 i1g.7m.7
W4VS99
TRINITY DN10772 c0 g1TRINITY DN10772 c0 g1 i1g.9m.9
