# Supplementary material for: Venomix: a simple bioinformatic pipeline for identifying and characterizing toxin gene candidates from transcriptomic data
Source: PeerJ. 2018 Jul 31;6:e5361. doi: 10.7717/peerj.5361 (PMC6074769; doi:10.7717/peerj.5361)
Supplement: Supplemental Information 2 [file peerj-06-5361-s002.gz › FinalOutput_1E-20/Kunitz-type_serine_protease_inhibitor_Kunitz-1_1/finaltree.pdf]

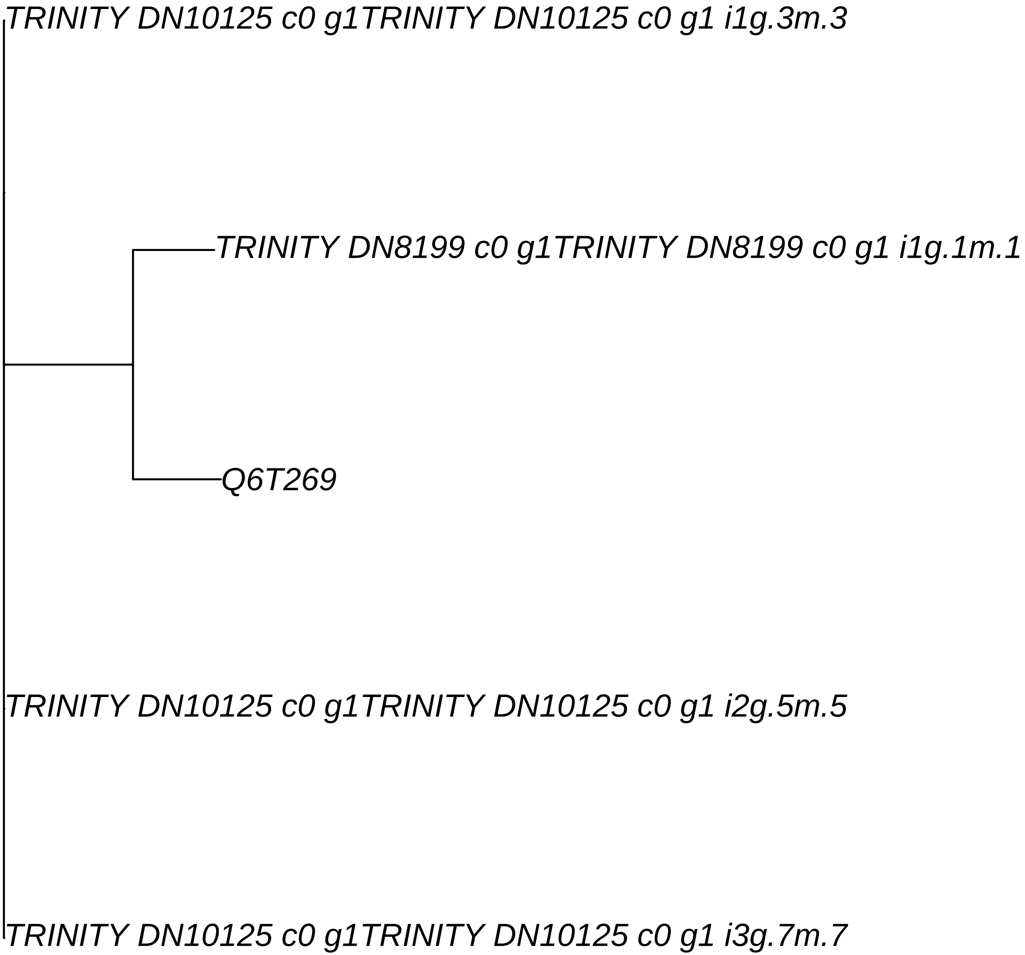
TRINITY DN10125 c0 g1TRINITY DN10125 c0 g1 i1g.3m.3
TRINITY DN8199 c0 g1TRINITY DN8199 c0 g1 i1g.1m.1
Q6T269
TRINITY DN10125 c0 g1TRINITY DN10125 c0 g1 i2g.5m.5
TRINITY DN10125 c0 g1TRINITY DN10125 c0 g1 i3g.7m.7
