# Supplementary material for: Venomix: a simple bioinformatic pipeline for identifying and characterizing toxin gene candidates from transcriptomic data
Source: PeerJ. 2018 Jul 31;6:e5361. doi: 10.7717/peerj.5361 (PMC6074769; doi:10.7717/peerj.5361)
Supplement: Supplemental Information 2 [file peerj-06-5361-s002.gz › FinalOutput_1E-6/Iota-conotoxin_RXIA_13/finaltree.pdf]

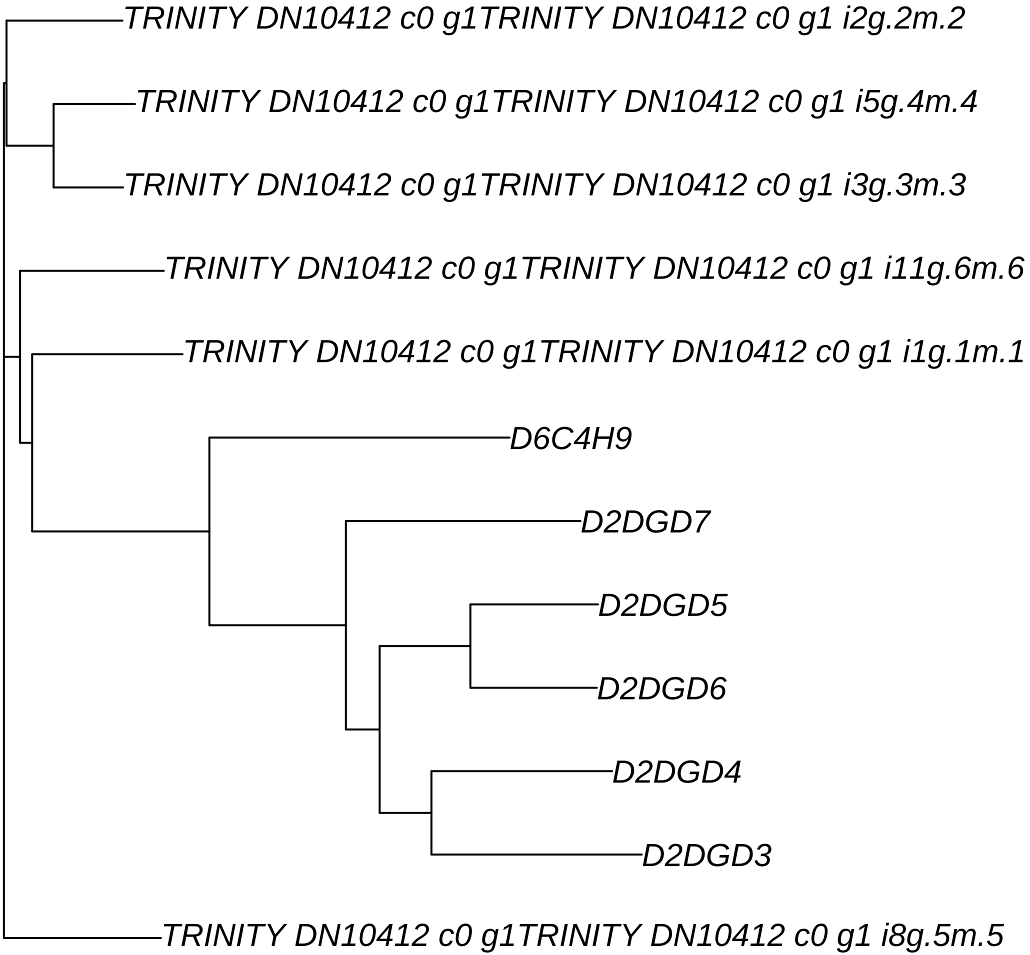
TRINITY DN10412 c0 g1TRINITY DN10412 c0 g1 i2g.2m.2
TRINITY DN10412 c0 g1TRINITY DN10412 c0 g1 i5g.4m.4
TRINITY DN10412 c0 g1TRINITY DN10412 c0 g1 i3g.3m.3
TRINITY DN10412 c0 g1TRINITY DN10412 c0 g1 i11g.6m.6
TRINITY DN10412 c0 g1TRINITY DN10412 c0 g1 i1g.1m.1
D6C4H9
D2DGD7
D2DGD5
D2DGD6
D2DGD4
D2DGD3
TRINITY DN10412 c0 g1TRINITY DN10412 c0 g1 i8g.5m.5
