# Supplementary material for: Venomix: a simple bioinformatic pipeline for identifying and characterizing toxin gene candidates from transcriptomic data
Source: PeerJ. 2018 Jul 31;6:e5361. doi: 10.7717/peerj.5361 (PMC6074769; doi:10.7717/peerj.5361)
Supplement: Supplemental Information 2 [file peerj-06-5361-s002.gz › FinalOutput_1E-6/Conotoxin_MiEr92_6/finaltree.pdf]

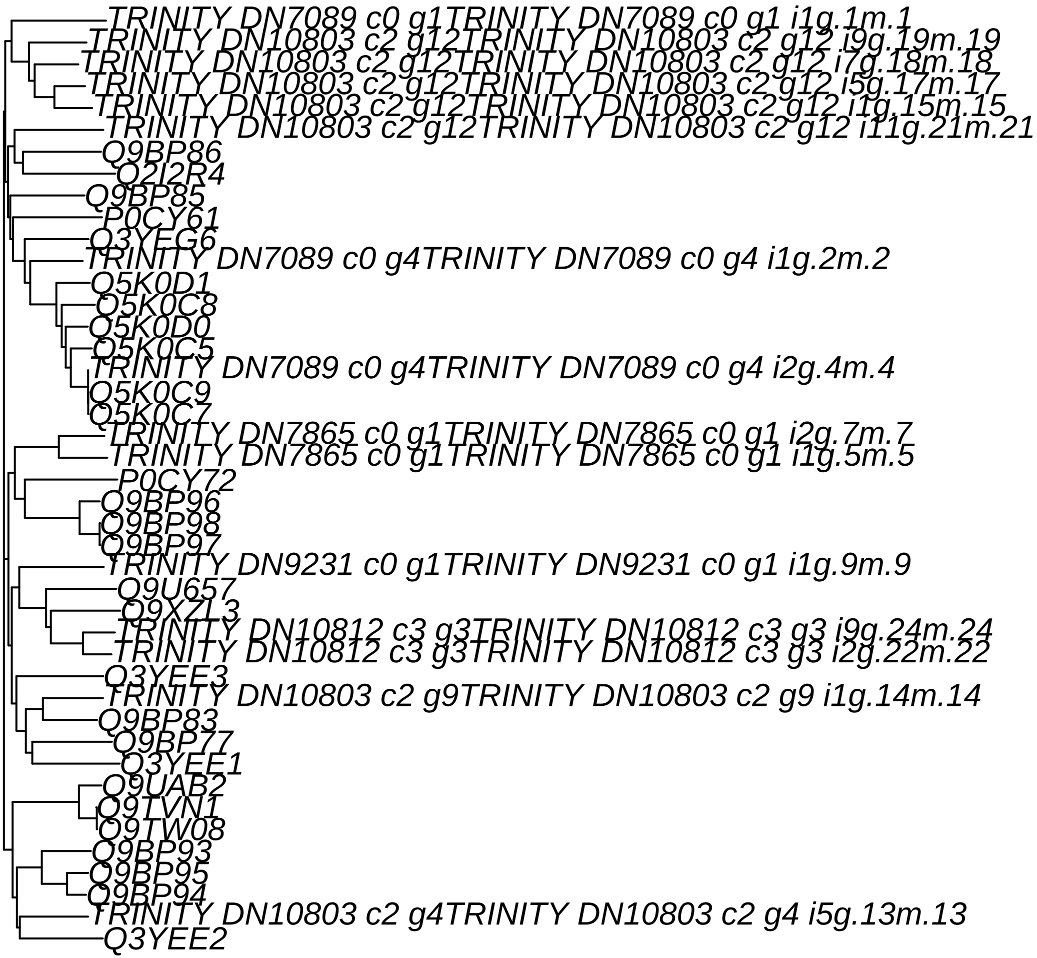
TRINITY DN7089 c0 g1TRINITY DN7089 c0 g1 i1g.1m.1
TRINITY DN10803 c2 g12TRINITY DN10803 c2 g12 i9g.19m.19
TRINITY DN10803 c2 g12TRINITY DN10803 c2 g12 i7g.18m.18
TRINITY DN10803 c2 g12TRINITY DN10803 c2 g12 i5g.17m.17
TRINITY DN10803 c2 g12TRINITY DN10803 c2 g12 i1g.15m.15
TRINITY DN10803 c2 g12TRINITY DN10803 c2 g12 i11g.21m.21
Q9BP86
Q2I2R4
Q9BP85
P0CY61
Q3YEG6
TRINITY DN7089 c0 g4TRINITY DN7089 c0 g4 i1g.2m.2
Q5K0D1
Q5K0C8
Q5K0D0
Q5K0C5
TRINITY DN7089 c0 g4TRINITY DN7089 c0 g4 i2g.4m.4
Q5K0C9
Q5K0C7
TRINITY DN7865 c0 g1TRINITY DN7865 c0 g1 i2g.7m.7
TRINITY DN7865 c0 g1TRINITY DN7865 c0 g1 i1g.5m.5
P0CY72
Q9BP96
Q9BP98
Q9BP97
TRINITY DN9231 c0 g1TRINITY DN9231 c0 g1 i1g.9m.9
Q9U657
Q9XZL3
TRINITY DN10812 c3 g3TRINITY DN10812 c3 g3 i9g.24m.24
TRINITY DN10812 c3 g3TRINITY DN10812 c3 g3 i2g.22m.22
Q3YEE3
TRINITY DN10803 c2 g9TRINITY DN10803 c2 g9 i1g.14m.14
Q9BP83
Q9BP77
Q3YEE1
Q9UAB2
Q9TVN1
Q9TW08
Q9BP93
Q9BP95
Q9BP94
TRINITY DN10803 c2 g4TRINITY DN10803 c2 g4 i5g.13m.13
Q3YEE2
