# Supplementary material for: Venomix: a simple bioinformatic pipeline for identifying and characterizing toxin gene candidates from transcriptomic data
Source: PeerJ. 2018 Jul 31;6:e5361. doi: 10.7717/peerj.5361 (PMC6074769; doi:10.7717/peerj.5361)
Supplement: Supplemental Information 2 [file peerj-06-5361-s002.gz › FinalOutput_1E-6/Kunitz-type_serine_protease_inhibitor_2_4/finaltree.pdf]

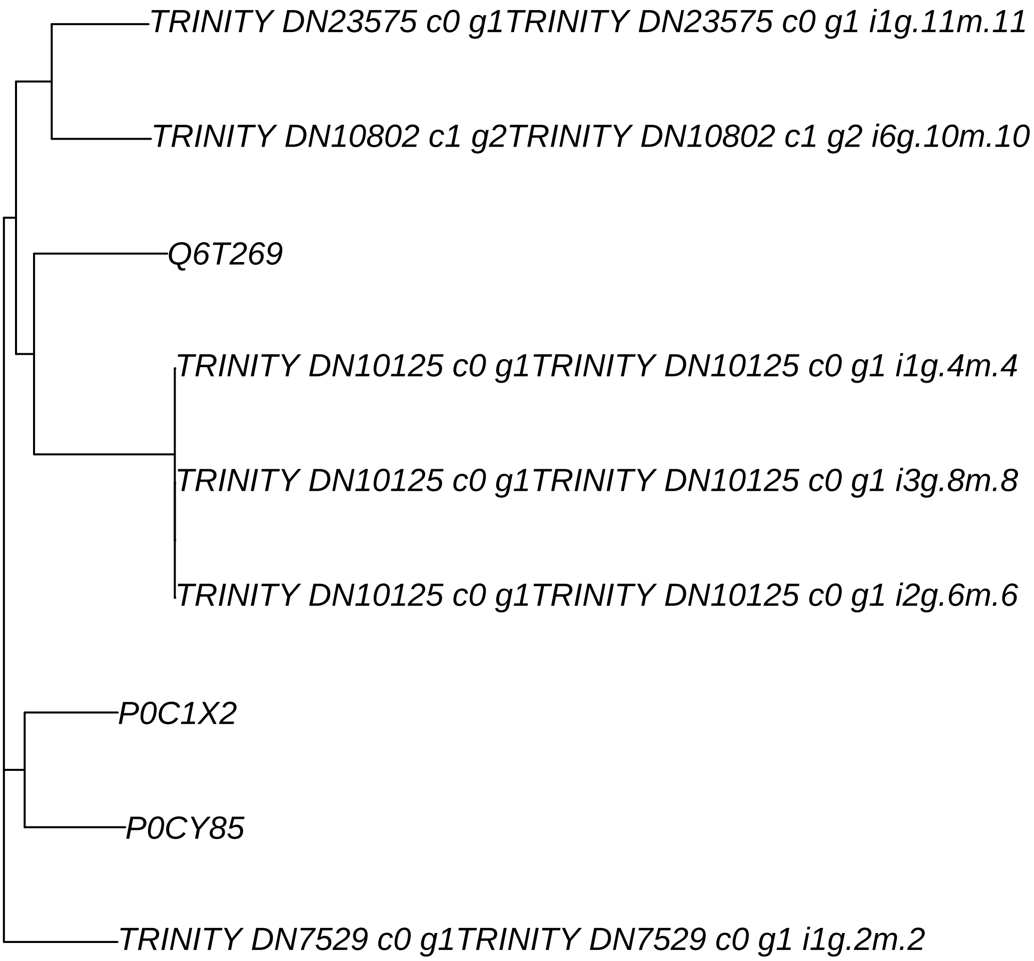

TRINITY DN23575 c0 g1TRINITY DN23575 c0 g1 i1g.11m.11
TRINITY DN10802 c1 g2TRINITY DN10802 c1 g2 i6g.10m.10
Q6T269
TRINITY DN10125 c0 g1TRINITY DN10125 c0 g1 i1g.4m.4
TRINITY DN10125 c0 g1TRINITY DN10125 c0 g1 i3g.8m.8
TRINITY DN10125 c0 g1TRINITY DN10125 c0 g1 i2g.6m.6
P0C1X2
P0CY85
TRINITY DN7529 c0 g1TRINITY DN7529 c0 g1 i1g.2m.2
