# Supplementary material for: Venomix: a simple bioinformatic pipeline for identifying and characterizing toxin gene candidates from transcriptomic data
Source: PeerJ. 2018 Jul 31;6:e5361. doi: 10.7717/peerj.5361 (PMC6074769; doi:10.7717/peerj.5361)
Supplement: Supplemental Information 2 [file peerj-06-5361-s002.gz › FinalOutput_1E-6/Conodipine-M_alpha_chain_1/finaltree.pdf]

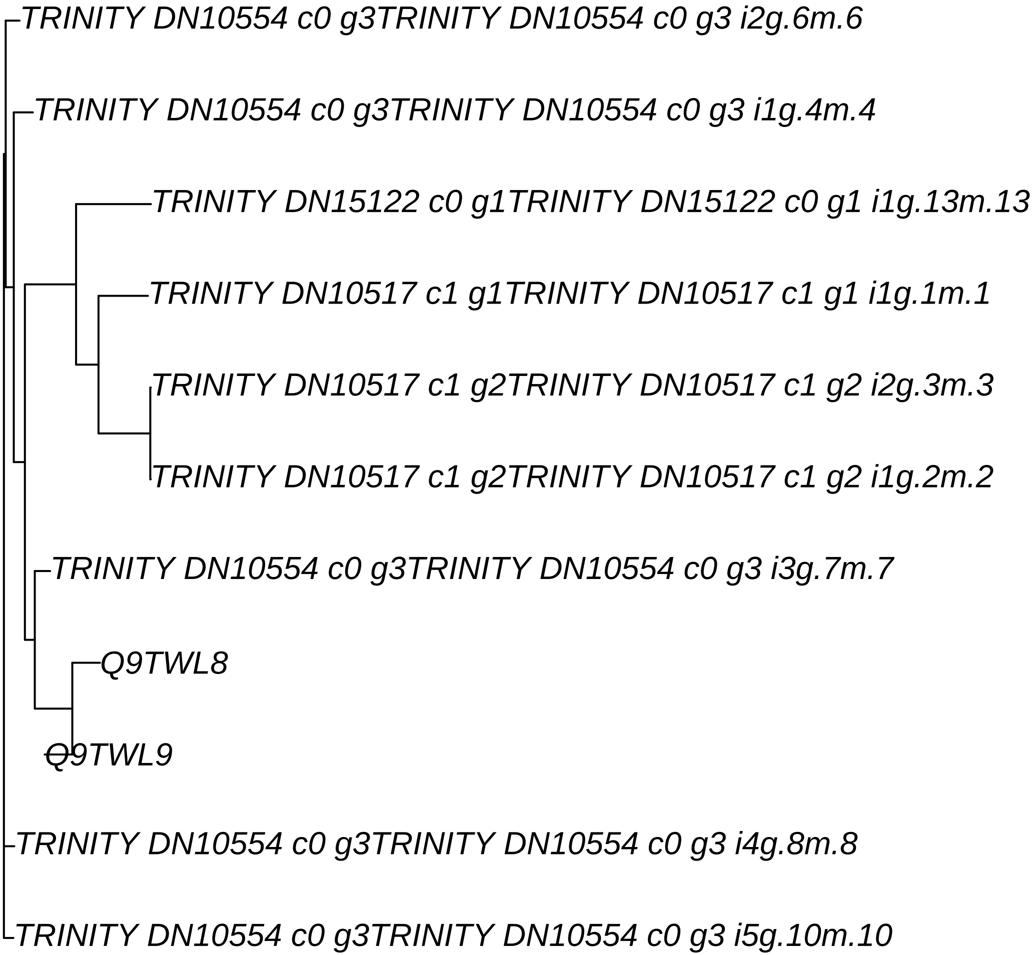
TRINITY DN10554 c0 g3TRINITY DN10554 c0 g3 i2g.6m.6
TRINITY DN10554 c0 g3TRINITY DN10554 c0 g3 i1g.4m.4
TRINITY DN15122 c0 g1TRINITY DN15122 c0 g1 i1g.13m.13
TRINITY DN10517 c1 g1TRINITY DN10517 c1 g1 i1g.1m.1
TRINITY DN10517 c1 g2TRINITY DN10517 c1 g2 i2g.3m.3
TRINITY DN10517 c1 g2TRINITY DN10517 c1 g2 i1g.2m.2
TRINITY DN10554 c0 g3TRINITY DN10554 c0 g3 i3g.7m.7
Q9TWL8
Q9TWL9
TRINITY DN10554 c0 g3TRINITY DN10554 c0 g3 i4g.8m.8
TRINITY DN10554 c0 g3TRINITY DN10554 c0 g3 i5g.10m.10
