# Supplementary material for: Venomix: a simple bioinformatic pipeline for identifying and characterizing toxin gene candidates from transcriptomic data
Source: PeerJ. 2018 Jul 31;6:e5361. doi: 10.7717/peerj.5361 (PMC6074769; doi:10.7717/peerj.5361)
Supplement: Supplemental Information 2 [file peerj-06-5361-s002.gz › FinalOutput_1E-6/Alpha-latroinsectotoxin-Lt1a_2/finaltree.pdf]

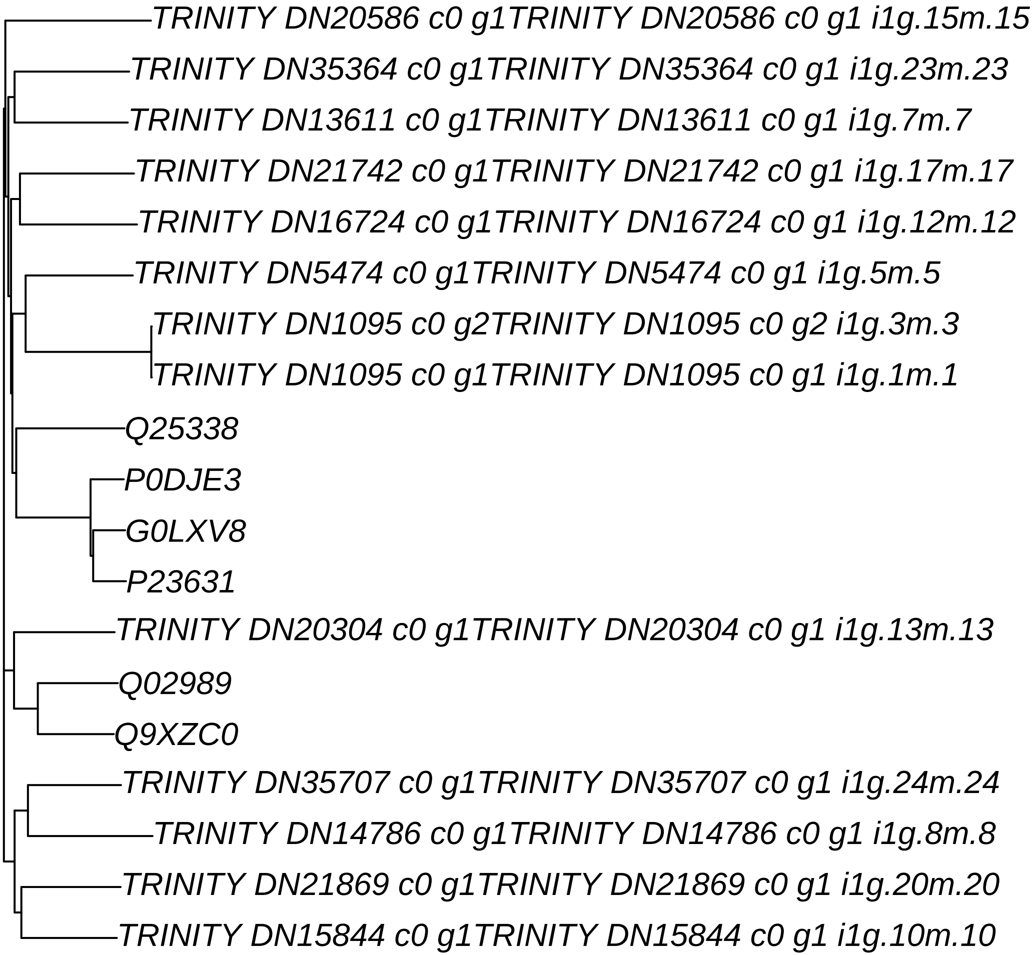
TRINITY DN20586 c0 g1TRINITY DN20586 c0 g1 i1g.15m.15
TRINITY DN35364 c0 g1TRINITY DN35364 c0 g1 i1g.23m.23
TRINITY DN13611 c0 g1TRINITY DN13611 c0 g1 i1g.7m.7
TRINITY DN21742 c0 g1TRINITY DN21742 c0 g1 i1g.17m.17
TRINITY DN16724 c0 g1TRINITY DN16724 c0 g1 i1g.12m.12
TRINITY DN5474 c0 g1TRINITY DN5474 c0 g1 i1g.5m.5
TRINITY DN1095 c0 g2TRINITY DN1095 c0 g2 i1g.3m.3
TRINITY DN1095 c0 g1TRINITY DN1095 c0 g1 i1g.1m.1
Q25338
P0DJE3
G0LXV8
P23631
TRINITY DN20304 c0 g1TRINITY DN20304 c0 g1 i1g.13m.13
Q02989
Q9XZC0
TRINITY DN35707 c0 g1TRINITY DN35707 c0 g1 i1g.24m.24
TRINITY DN14786 c0 g1TRINITY DN14786 c0 g1 i1g.8m.8
TRINITY DN21869 c0 g1TRINITY DN21869 c0 g1 i1g.20m.20
TRINITY DN15844 c0 g1TRINITY DN15844 c0 g1 i1g.10m.10
