# Supplementary material for: Venomix: a simple bioinformatic pipeline for identifying and characterizing toxin gene candidates from transcriptomic data
Source: PeerJ. 2018 Jul 31;6:e5361. doi: 10.7717/peerj.5361 (PMC6074769; doi:10.7717/peerj.5361)
Supplement: Supplemental Information 2 [file peerj-06-5361-s002.gz › FinalOutput_1E-6/Delta-latroinsectotoxin-Lt1a_1/finaltree.pdf]

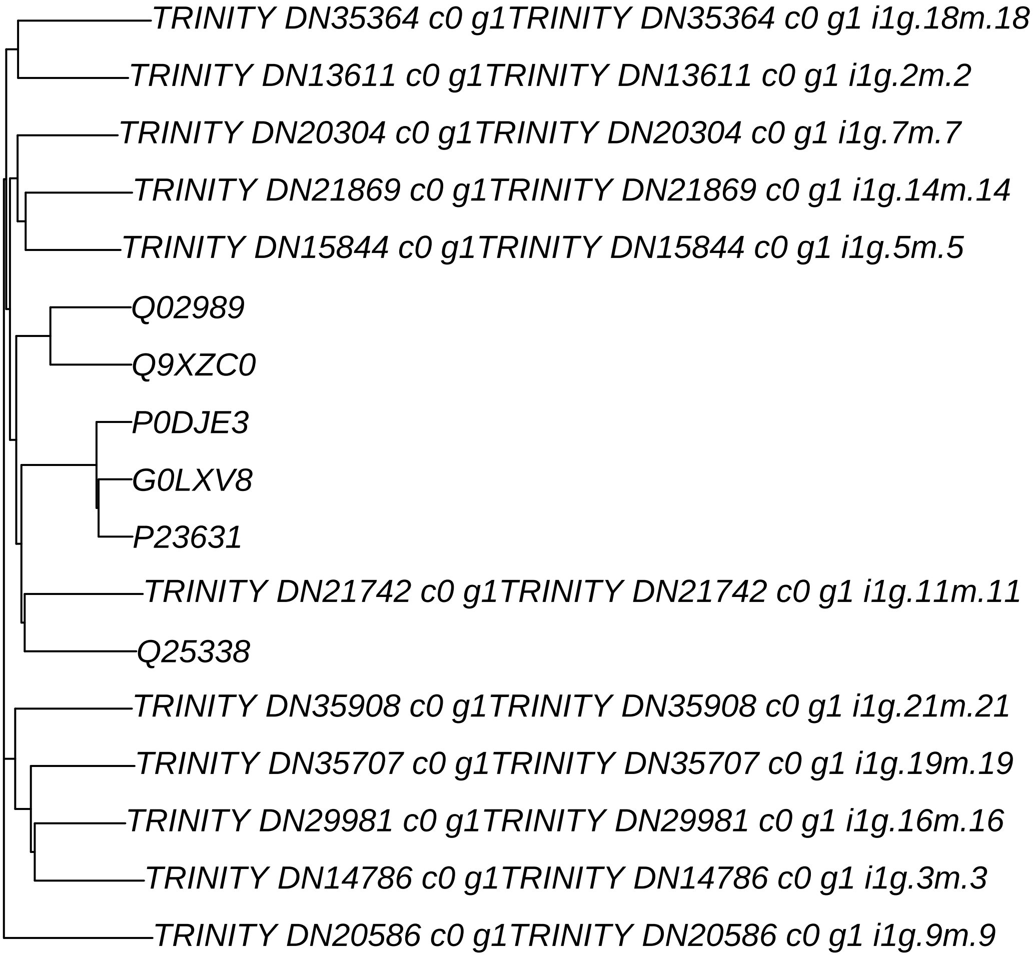
TRINITY DN35364 c0 g1TRINITY DN35364 c0 g1 i1g.18m.18
TRINITY DN13611 c0 g1TRINITY DN13611 c0 g1 i1g.2m.2
TRINITY DN20304 c0 g1TRINITY DN20304 c0 g1 i1g.7m.7
TRINITY DN21869 c0 g1TRINITY DN21869 c0 g1 i1g.14m.14
TRINITY DN15844 c0 g1TRINITY DN15844 c0 g1 i1g.5m.5
Q02989
Q9XZC0
P0DJE3
G0LXV8
P23631
TRINITY DN21742 c0 g1TRINITY DN21742 c0 g1 i1g.11m.11
Q25338
TRINITY DN35908 c0 g1TRINITY DN35908 c0 g1 i1g.21m.21
TRINITY DN35707 c0 g1TRINITY DN35707 c0 g1 i1g.19m.19
TRINITY DN29981 c0 g1TRINITY DN29981 c0 g1 i1g.16m.16
TRINITY DN14786 c0 g1TRINITY DN14786 c0 g1 i1g.3m.3
TRINITY DN20586 c0 g1TRINITY DN20586 c0 g1 i1g.9m.9
