# Supplementary material for: Venomix: a simple bioinformatic pipeline for identifying and characterizing toxin gene candidates from transcriptomic data
Source: PeerJ. 2018 Jul 31;6:e5361. doi: 10.7717/peerj.5361 (PMC6074769; doi:10.7717/peerj.5361)
Supplement: Supplemental Information 2 [file peerj-06-5361-s002.gz › FinalOutput_1E-6/Alpha@kappa-conotoxin_pl14a_27/finaltree.pdf]

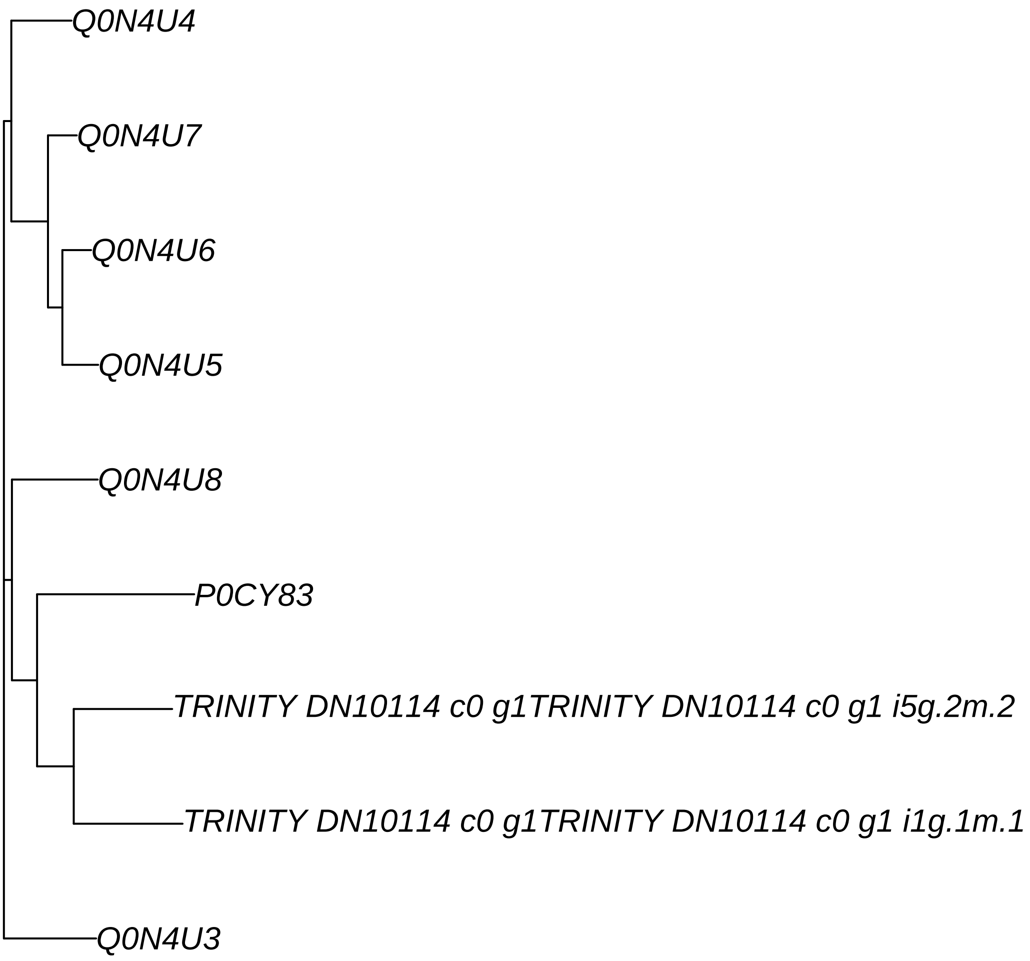

Q0N4U4
Q0N4U7
Q0N4U6
Q0N4U5
Q0N4U8
P0CY83
TRINITY DN10114 c0 g1TRINITY DN10114 c0 g1 i5g.2m.2
TRINITY DN10114 c0 g1TRINITY DN10114 c0 g1 i1g.1m.1
Q0N4U3
