# Supplementary material for: Venomix: a simple bioinformatic pipeline for identifying and characterizing toxin gene candidates from transcriptomic data
Source: PeerJ. 2018 Jul 31;6:e5361. doi: 10.7717/peerj.5361 (PMC6074769; doi:10.7717/peerj.5361)
Supplement: Supplemental Information 2 [file peerj-06-5361-s002.gz › FinalOutput_1E-6/Conotoxin_Lt6/finaltree.pdf]

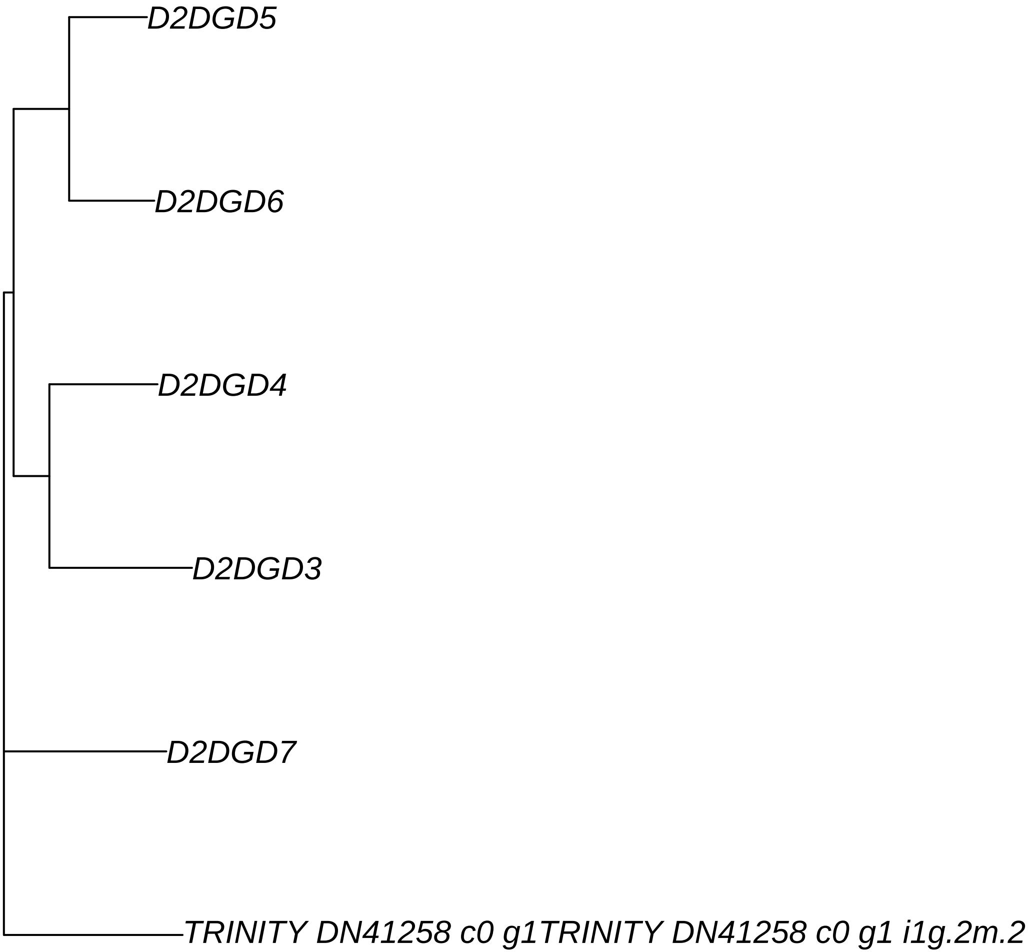
D2DGD5
D2DGD6
D2DGD4
D2DGD3
D2DGD7
TRINITY DN41258 c0 g1TRINITY DN41258 c0 g1 i1g.2m.2
