# Supplementary material for: Venomix: a simple bioinformatic pipeline for identifying and characterizing toxin gene candidates from transcriptomic data
Source: PeerJ. 2018 Jul 31;6:e5361. doi: 10.7717/peerj.5361 (PMC6074769; doi:10.7717/peerj.5361)
Supplement: Supplemental Information 2 [file peerj-06-5361-s002.gz › FinalOutput_1E-6/Conotoxin_Bu6_2/finaltree.pdf]

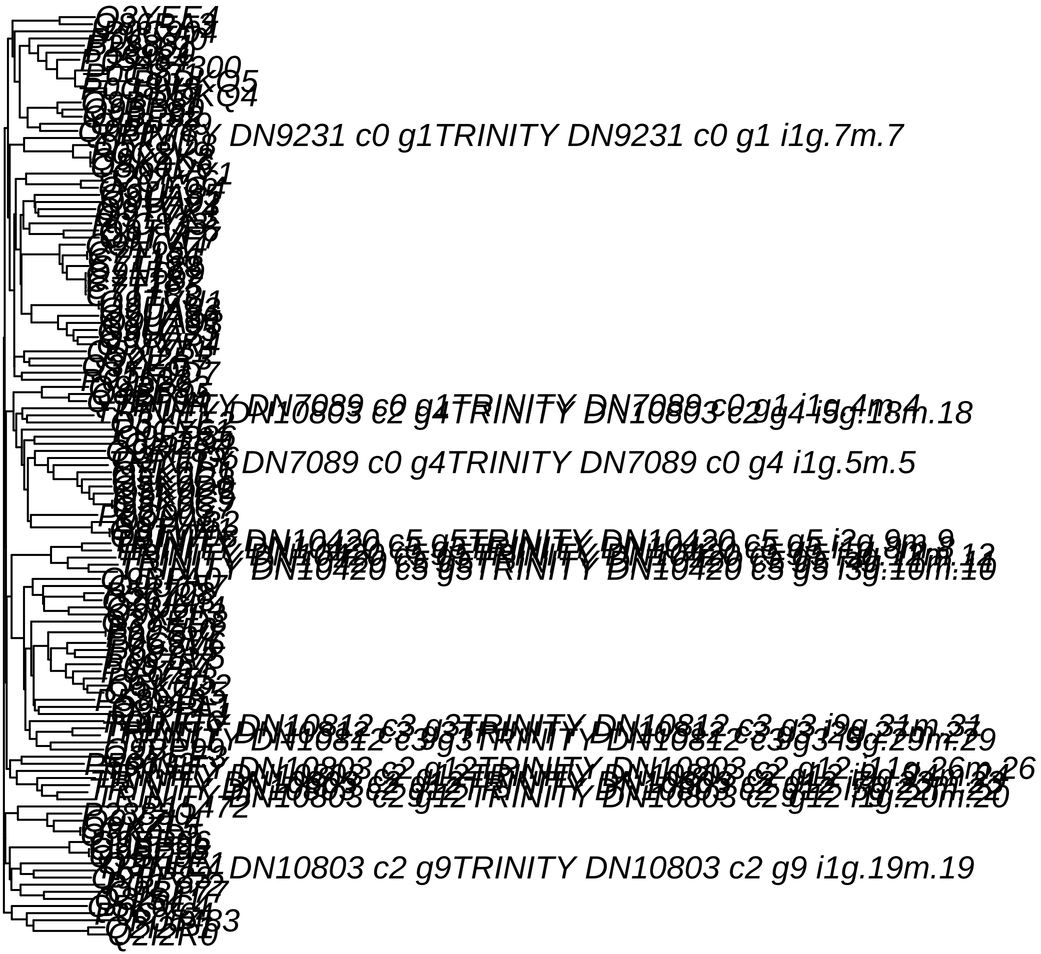
Q3YEF4
DN9231 c0 g1TRINITY DN9231 c0 g1 i1g.7m.7
DN7089 c0 g4TRINITY DN7089 c0 g4 i1g.5m.5
DN10803 c2 g9TRINITY DN10803 c2 g9 i1g.19m.19
Q2I2R0
