# Supplementary material for: Venomix: a simple bioinformatic pipeline for identifying and characterizing toxin gene candidates from transcriptomic data
Source: PeerJ. 2018 Jul 31;6:e5361. doi: 10.7717/peerj.5361 (PMC6074769; doi:10.7717/peerj.5361)
Supplement: Supplemental Information 2 [file peerj-06-5361-s002.gz › FinalOutput_1E-6/Delta-conotoxin_TxVIA_96/finaltree.pdf]

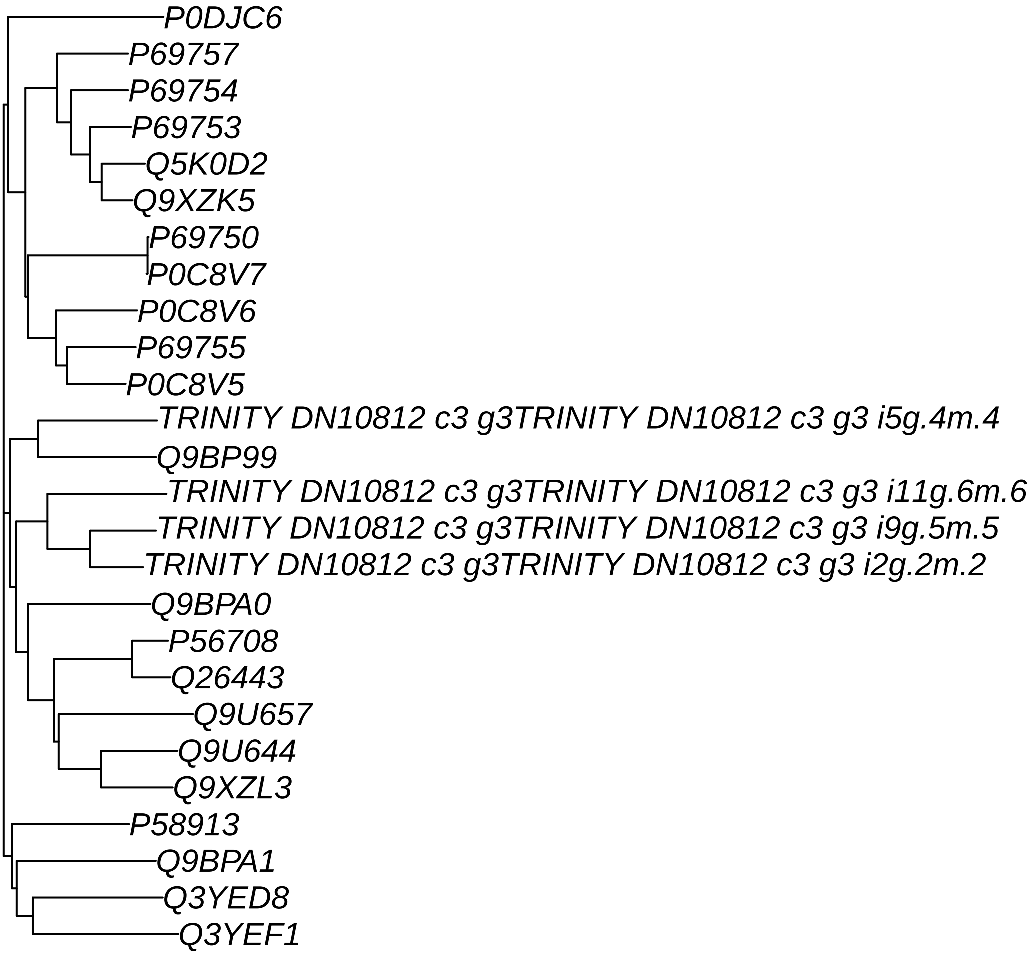
P0DJC6
P69757
P69754
P69753
Q5K0D2
Q9XZK5
P69750
P0C8V7
P0C8V6
P69755
P0C8V5
TRINITY DN10812 c3 g3TRINITY DN10812 c3 g3 i5g.4m.4
Q9BP99
TRINITY DN10812 c3 g3TRINITY DN10812 c3 g3 i11g.6m.6
TRINITY DN10812 c3 g3TRINITY DN10812 c3 g3 i9g.5m.5
TRINITY DN10812 c3 g3TRINITY DN10812 c3 g3 i2g.2m.2
Q9BPA0
P56708
Q26443
Q9U657
Q9U644
Q9XZL3
P58913
Q9BPA1
Q3YED8
Q3YEF1
