# Supplementary material for: Venomix: a simple bioinformatic pipeline for identifying and characterizing toxin gene candidates from transcriptomic data
Source: PeerJ. 2018 Jul 31;6:e5361. doi: 10.7717/peerj.5361 (PMC6074769; doi:10.7717/peerj.5361)
Supplement: Supplemental Information 2 [file peerj-06-5361-s002.gz › FinalOutput_1E-6/Conotoxin_ArMKLT2-0313_21/finaltree.pdf]

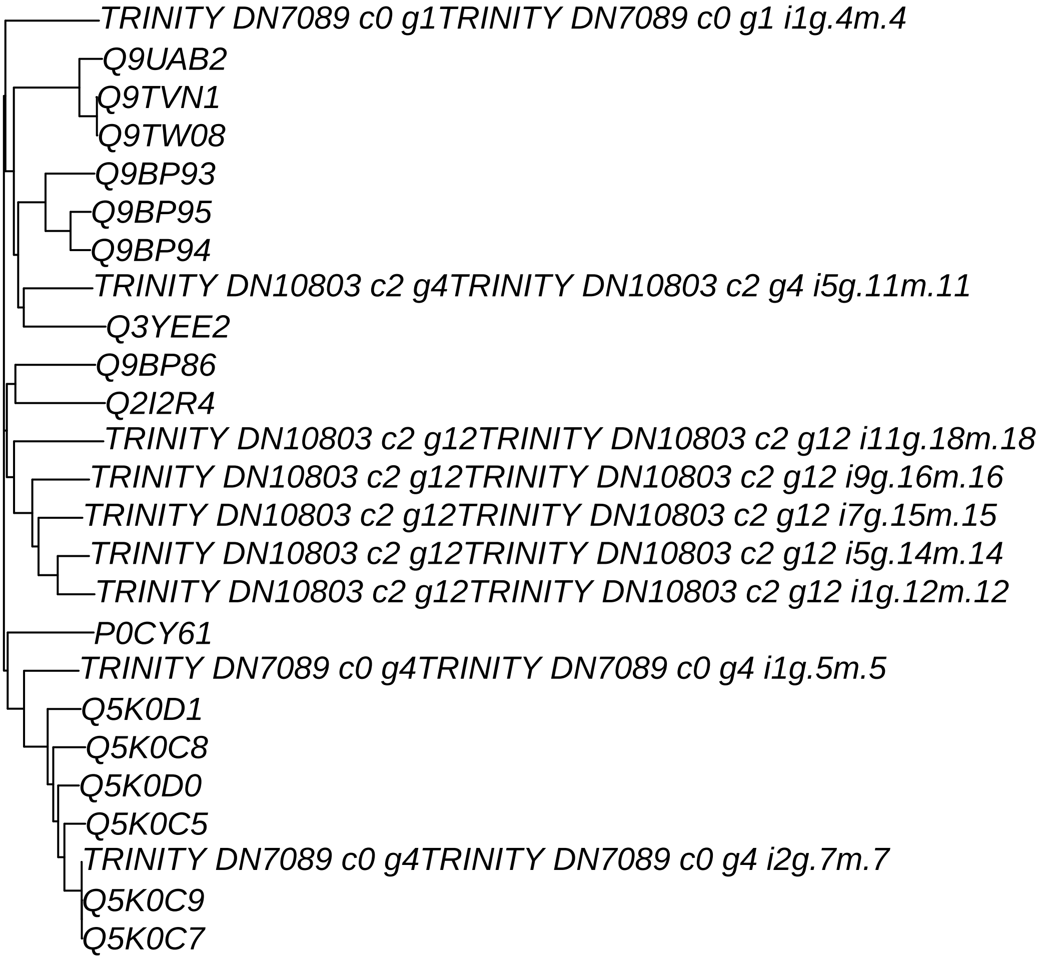
TRINITY DN7089 c0 g1TRINITY DN7089 c0 g1 i1g.4m.4
Q9UAB2
Q9TVN1
Q9TW08
Q9BP93
Q9BP95
Q9BP94
TRINITY DN10803 c2 g4TRINITY DN10803 c2 g4 i5g.11m.11
Q3YEE2
Q9BP86
Q2I2R4
TRINITY DN10803 c2 g12TRINITY DN10803 c2 g12 i11g.18m.18
TRINITY DN10803 c2 g12TRINITY DN10803 c2 g12 i9g.16m.16
TRINITY DN10803 c2 g12TRINITY DN10803 c2 g12 i7g.15m.15
TRINITY DN10803 c2 g12TRINITY DN10803 c2 g12 i5g.14m.14
TRINITY DN10803 c2 g12TRINITY DN10803 c2 g12 i1g.12m.12
P0CY61
TRINITY DN7089 c0 g4TRINITY DN7089 c0 g4 i1g.5m.5
Q5K0D1
Q5K0C8
Q5K0D0
Q5K0C5
TRINITY DN7089 c0 g4TRINITY DN7089 c0 g4 i2g.7m.7
Q5K0C9
Q5K0C7
