# Supplementary material for: Venomix: a simple bioinformatic pipeline for identifying and characterizing toxin gene candidates from transcriptomic data
Source: PeerJ. 2018 Jul 31;6:e5361. doi: 10.7717/peerj.5361 (PMC6074769; doi:10.7717/peerj.5361)
Supplement: Supplemental Information 2 [file peerj-06-5361-s002.gz › FinalOutput_1E-6/Conophysin-R_1/finaltree.pdf]

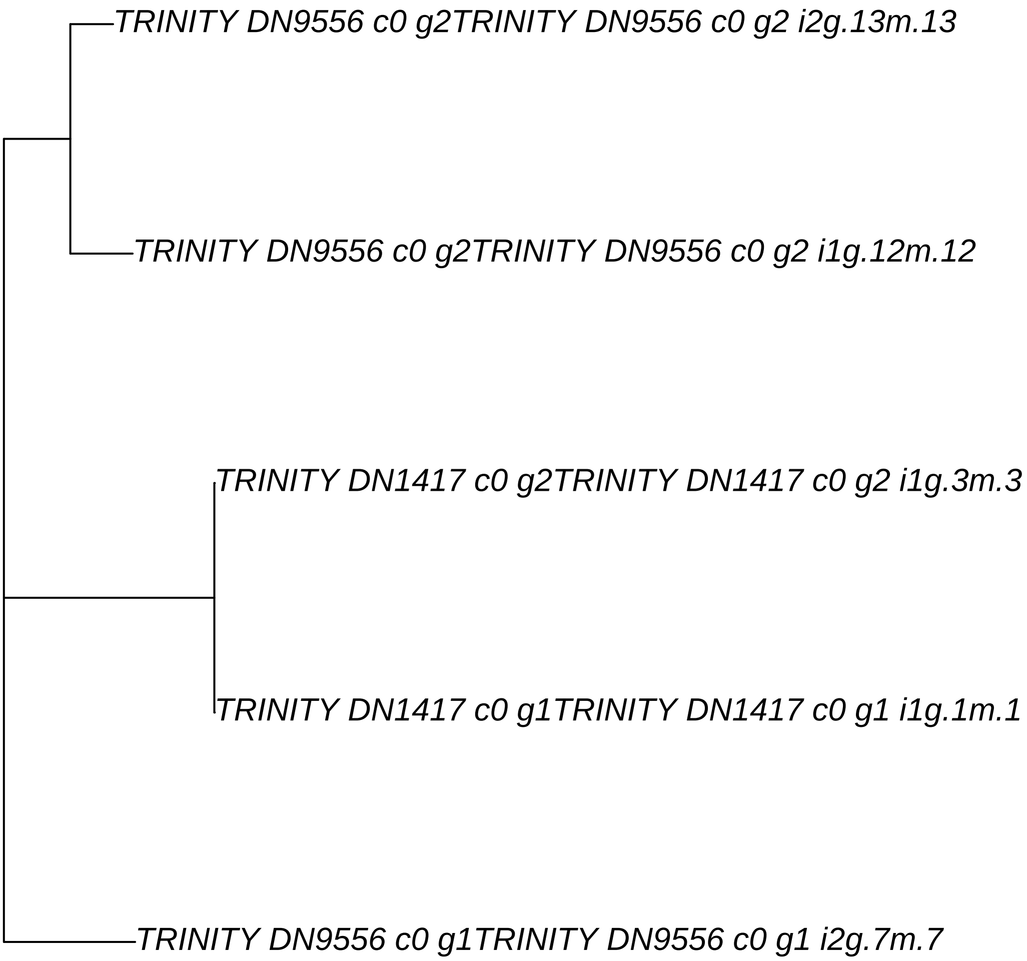
TRINITY DN9556 c0 g2TRINITY DN9556 c0 g2 i2g.13m.13
TRINITY DN9556 c0 g2TRINITY DN9556 c0 g2 i1g.12m.12
TRINITY DN1417 c0 g2TRINITY DN1417 c0 g2 i1g.3m.3
TRINITY DN1417 c0 g1TRINITY DN1417 c0 g1 i1g.1m.1
TRINITY DN9556 c0 g1TRINITY DN9556 c0 g1 i2g.7m.7
