# Supplementary material for: Venomix: a simple bioinformatic pipeline for identifying and characterizing toxin gene candidates from transcriptomic data
Source: PeerJ. 2018 Jul 31;6:e5361. doi: 10.7717/peerj.5361 (PMC6074769; doi:10.7717/peerj.5361)
Supplement: Supplemental Information 2 [file peerj-06-5361-s002.gz › FinalOutput_1E-6/Nematocyte_expressed_protein_6_1/finaltree.pdf]

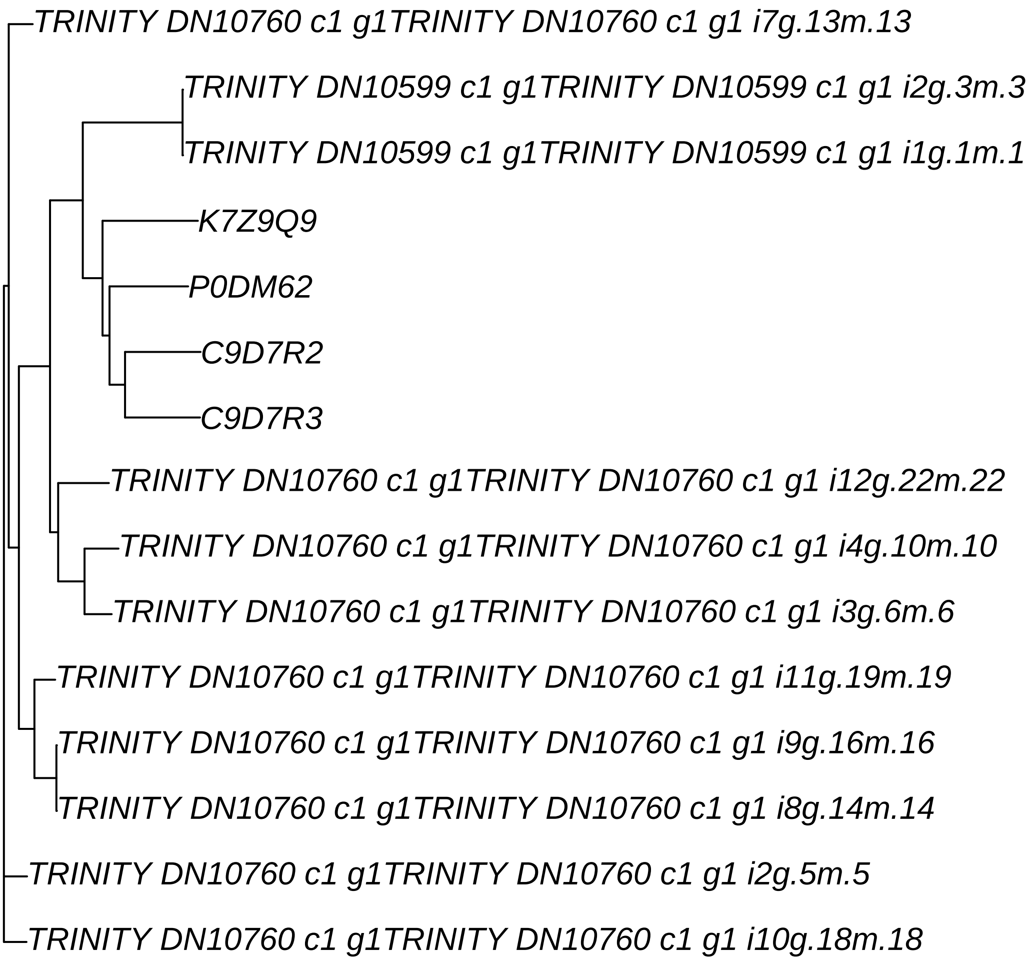
TRINITY DN10760 c1 g1TRINITY DN10760 c1 g1 i7g.13m.13
TRINITY DN10599 c1 g1TRINITY DN10599 c1 g1 i2g.3m.3
TRINITY DN10599 c1 g1TRINITY DN10599 c1 g1 i1g.1m.1
K7Z9Q9
P0DM62
C9D7R2
C9D7R3
TRINITY DN10760 c1 g1TRINITY DN10760 c1 g1 i12g.22m.22
TRINITY DN10760 c1 g1TRINITY DN10760 c1 g1 i4g.10m.10
TRINITY DN10760 c1 g1TRINITY DN10760 c1 g1 i3g.6m.6
TRINITY DN10760 c1 g1TRINITY DN10760 c1 g1 i11g.19m.19
TRINITY DN10760 c1 g1TRINITY DN10760 c1 g1 i9g.16m.16
TRINITY DN10760 c1 g1TRINITY DN10760 c1 g1 i8g.14m.14
TRINITY DN10760 c1 g1TRINITY DN10760 c1 g1 i2g.5m.5
TRINITY DN10760 c1 g1TRINITY DN10760 c1 g1 i10g.18m.18
