# Supplementary material for: Venomix: a simple bioinformatic pipeline for identifying and characterizing toxin gene candidates from transcriptomic data
Source: PeerJ. 2018 Jul 31;6:e5361. doi: 10.7717/peerj.5361 (PMC6074769; doi:10.7717/peerj.5361)
Supplement: Supplemental Information 2 [file peerj-06-5361-s002.gz › FinalOutput_1E-6/Snaclec_coagulation_factor_X-activating_enzyme_light_chain_2_135/finaltree.pdf]

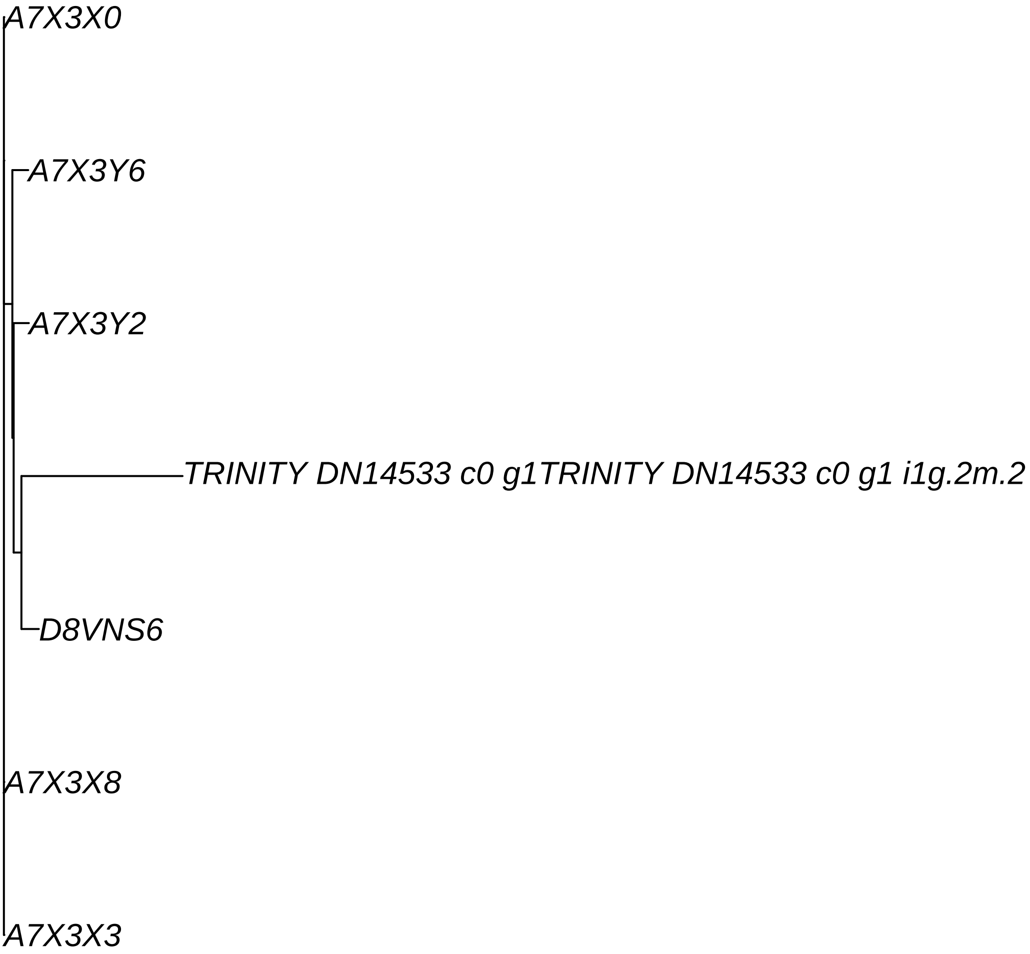
A7X3X0
A7X3Y6
A7X3Y2
TRINITY DN14533 c0 g1TRINITY DN14533 c0 g1 i1g.2m.2
D8VNS6
A7X3X8
A7X3X3
