# Supplementary material for: Venomix: a simple bioinformatic pipeline for identifying and characterizing toxin gene candidates from transcriptomic data
Source: PeerJ. 2018 Jul 31;6:e5361. doi: 10.7717/peerj.5361 (PMC6074769; doi:10.7717/peerj.5361)
Supplement: Supplemental Information 2 [file peerj-06-5361-s002.gz › FinalOutput_1E-6/Venom_prothrombin_activator_pseutarin-C_catalytic_subunit_12/finaltree.pdf]

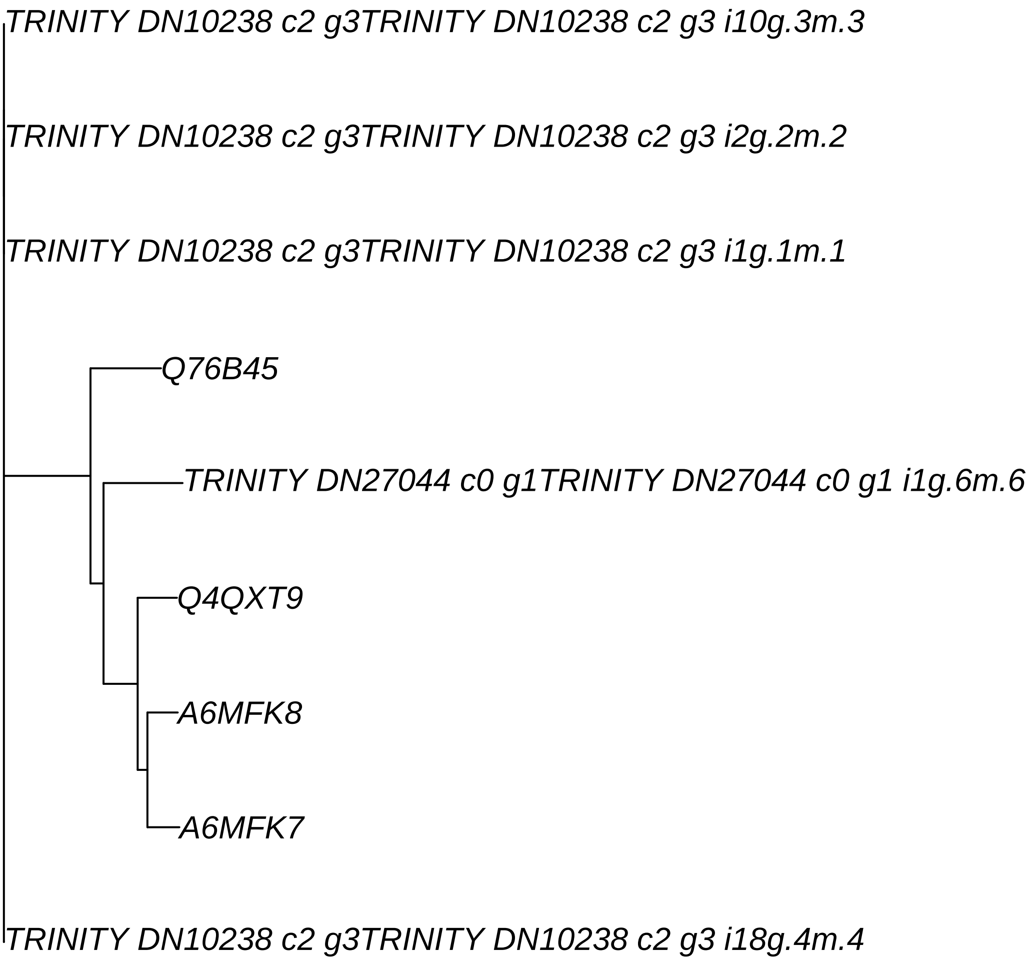
TRINITY DN10238 c2 g3TRINITY DN10238 c2 g3 i10g.3m.3
TRINITY DN10238 c2 g3TRINITY DN10238 c2 g3 i2g.2m.2
TRINITY DN10238 c2 g3TRINITY DN10238 c2 g3 i1g.1m.1
Q76B45
TRINITY DN27044 c0 g1TRINITY DN27044 c0 g1 i1g.6m.6
Q4QXT9
A6MFK8
A6MFK7
TRINITY DN10238 c2 g3TRINITY DN10238 c2 g3 i18g.4m.4
