# Supplementary material for: Venomix: a simple bioinformatic pipeline for identifying and characterizing toxin gene candidates from transcriptomic data
Source: PeerJ. 2018 Jul 31;6:e5361. doi: 10.7717/peerj.5361 (PMC6074769; doi:10.7717/peerj.5361)
Supplement: Supplemental Information 2 [file peerj-06-5361-s002.gz › FinalOutput_1E-6/Conotoxin_tx3a-a_227/finaltree.pdf]

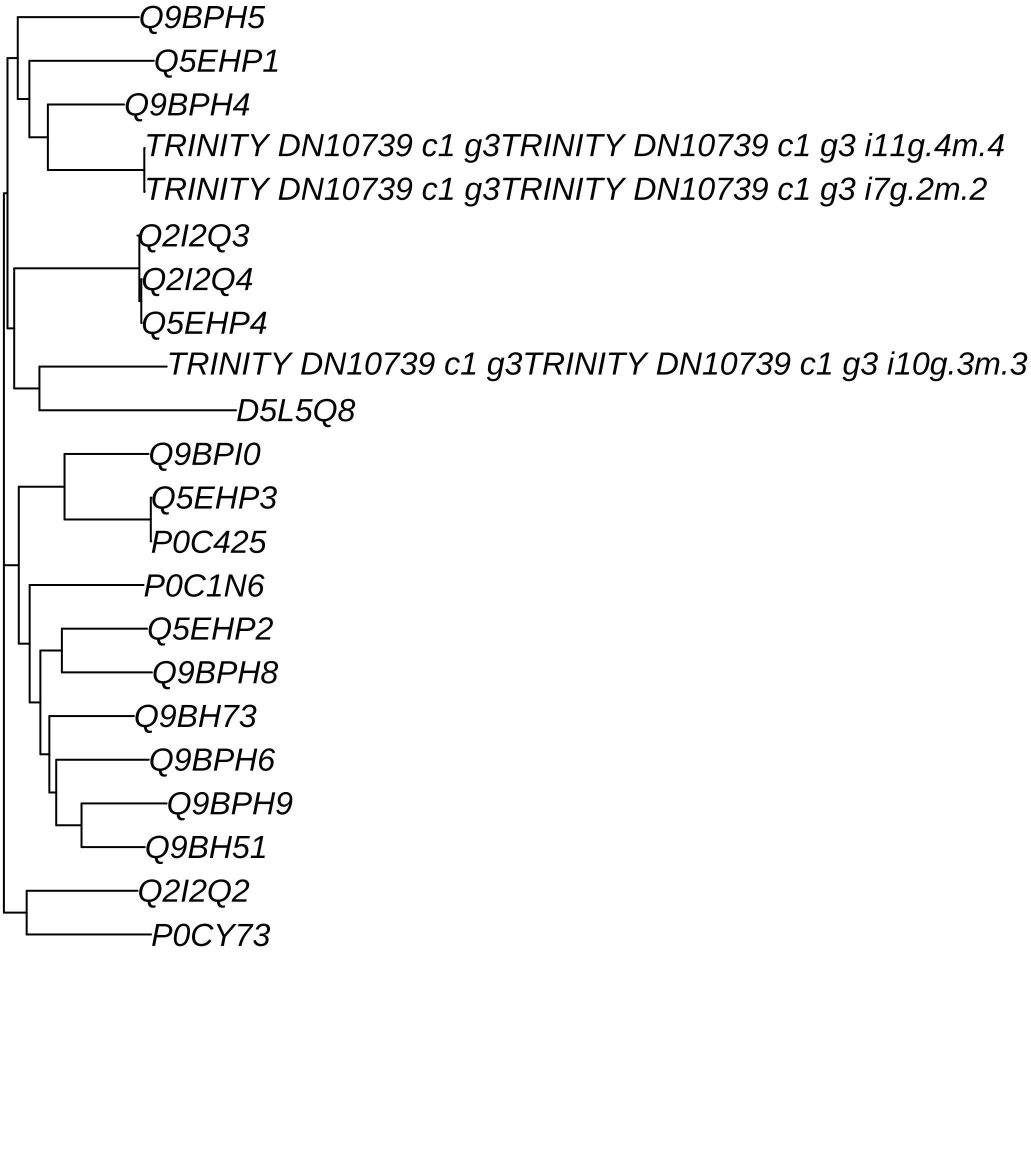
Q9BPH5
Q5EHP1
Q9BPH4
TRINITY DN10739 c1 g3TRINITY DN10739 c1 g3 i11g.4m.4
TRINITY DN10739 c1 g3TRINITY DN10739 c1 g3 i7g.2m.2
Q2I2Q3
Q2I2Q4
Q5EHP4
TRINITY DN10739 c1 g3TRINITY DN10739 c1 g3 i10g.3m.3
D5L5Q8
Q9BPI0
Q5EHP3
P0C425
P0C1N6
Q5EHP2
Q9BPH8
Q9BH73
Q9BPH6
Q9BPH9
Q9BH51
Q2I2Q2
P0CY73
