# Supplementary material for: Venomix: a simple bioinformatic pipeline for identifying and characterizing toxin gene candidates from transcriptomic data
Source: PeerJ. 2018 Jul 31;6:e5361. doi: 10.7717/peerj.5361 (PMC6074769; doi:10.7717/peerj.5361)
Supplement: Supplemental Information 2 [file peerj-06-5361-s002.gz › FinalOutput_1E-6/Conotoxin_Cl5/finaltree.pdf]

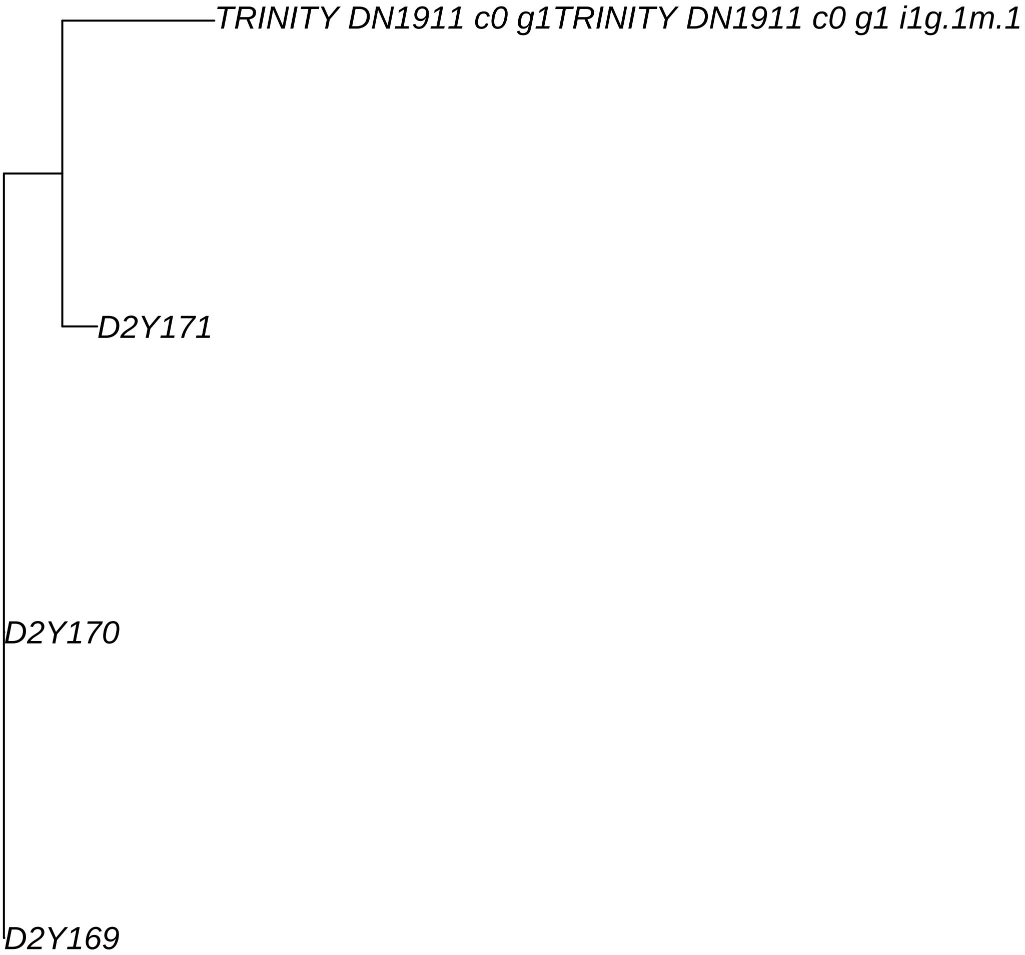
TRINITY DN1911 c0 g1TRINITY DN1911 c0 g1 i1g.1m.1
D2Y171
D2Y170
D2Y169
