# Supplementary material for: Venomix: a simple bioinformatic pipeline for identifying and characterizing toxin gene candidates from transcriptomic data
Source: PeerJ. 2018 Jul 31;6:e5361. doi: 10.7717/peerj.5361 (PMC6074769; doi:10.7717/peerj.5361)
Supplement: Supplemental Information 2 [file peerj-06-5361-s002.gz › FinalOutput_1E-6/Kunitz-type_serine_protease_inhibitor_bicolin_1/finaltree.pdf]

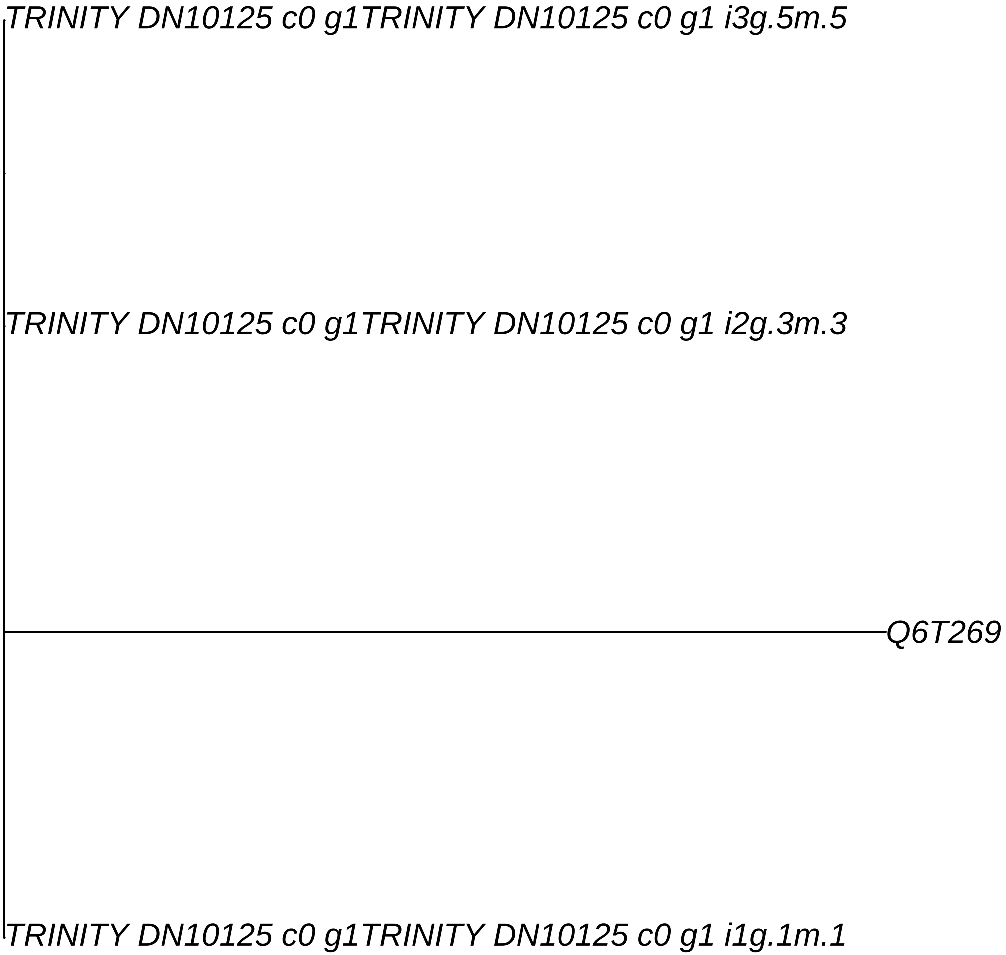
TRINITY DN10125 c0 g1TRINITY DN10125 c0 g1 i3g.5m.5
TRINITY DN10125 c0 g1TRINITY DN10125 c0 g1 i2g.3m.3
Q6T269
TRINITY DN10125 c0 g1TRINITY DN10125 c0 g1 i1g.1m.1
