# Supplementary material for: Venomix: a simple bioinformatic pipeline for identifying and characterizing toxin gene candidates from transcriptomic data
Source: PeerJ. 2018 Jul 31;6:e5361. doi: 10.7717/peerj.5361 (PMC6074769; doi:10.7717/peerj.5361)
Supplement: Supplemental Information 2 [file peerj-06-5361-s002.gz › FinalOutput_1E-6/Kunitz-type_serine_protease_inhibitor_Kunitz-1_1/finaltree.pdf]

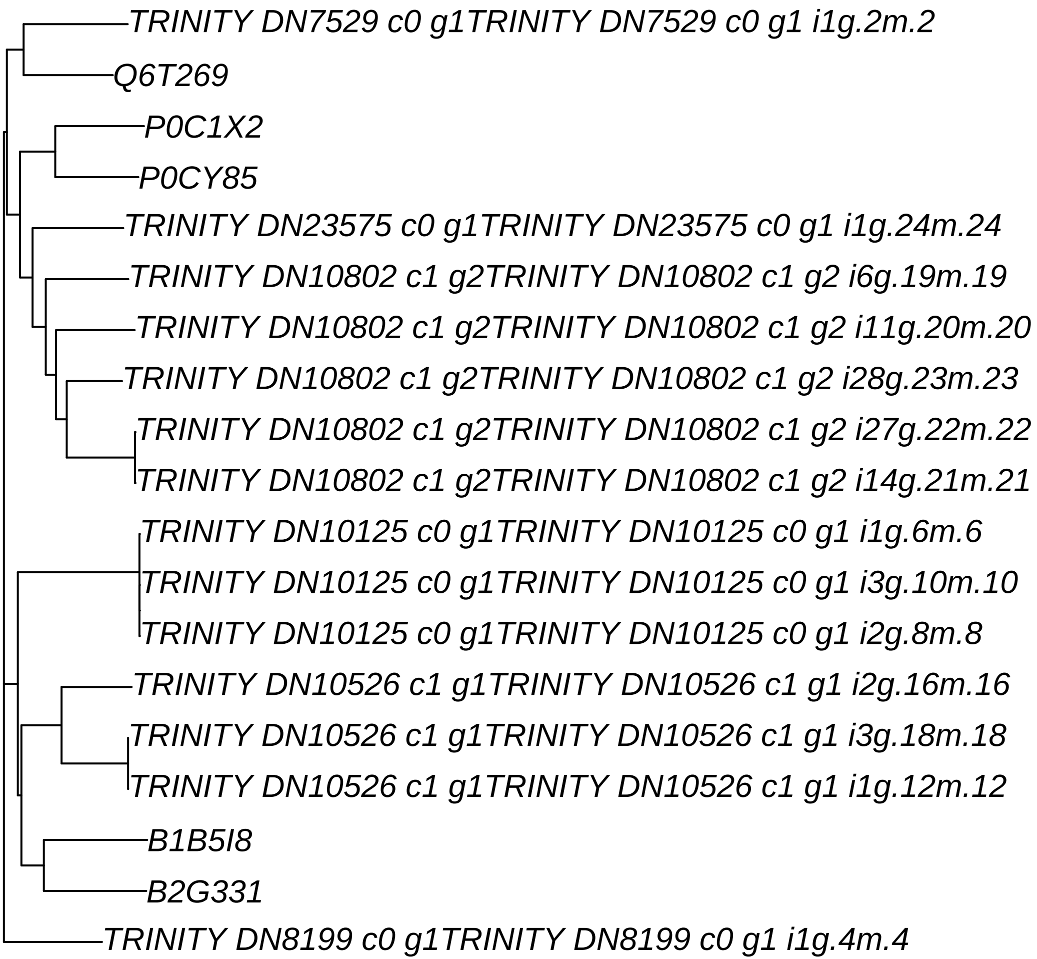
TRINITY DN7529 c0 g1TRINITY DN7529 c0 g1 i1g.2m.2
Q6T269
P0C1X2
P0CY85
TRINITY DN23575 c0 g1TRINITY DN23575 c0 g1 i1g.24m.24
TRINITY DN10802 c1 g2TRINITY DN10802 c1 g2 i6g.19m.19
TRINITY DN10802 c1 g2TRINITY DN10802 c1 g2 i11g.20m.20
TRINITY DN10802 c1 g2TRINITY DN10802 c1 g2 i28g.23m.23
TRINITY DN10802 c1 g2TRINITY DN10802 c1 g2 i27g.22m.22
TRINITY DN10802 c1 g2TRINITY DN10802 c1 g2 i14g.21m.21
TRINITY DN10125 c0 g1TRINITY DN10125 c0 g1 i1g.6m.6
TRINITY DN10125 c0 g1TRINITY DN10125 c0 g1 i3g.10m.10
TRINITY DN10125 c0 g1TRINITY DN10125 c0 g1 i2g.8m.8
TRINITY DN10526 c1 g1TRINITY DN10526 c1 g1 i2g.16m.16
TRINITY DN10526 c1 g1TRINITY DN10526 c1 g1 i3g.18m.18
TRINITY DN10526 c1 g1TRINITY DN10526 c1 g1 i1g.12m.12
B1B5I8
B2G331
TRINITY DN8199 c0 g1TRINITY DN8199 c0 g1 i1g.4m.4
