# Supplementary material for: Venomix: a simple bioinformatic pipeline for identifying and characterizing toxin gene candidates from transcriptomic data
Source: PeerJ. 2018 Jul 31;6:e5361. doi: 10.7717/peerj.5361 (PMC6074769; doi:10.7717/peerj.5361)
Supplement: Supplemental Information 4 [file peerj-06-5361-s004.gz › FinalOutput_E-20/Disintegrin-like_leberagin-C_8/finaltree.pdf]

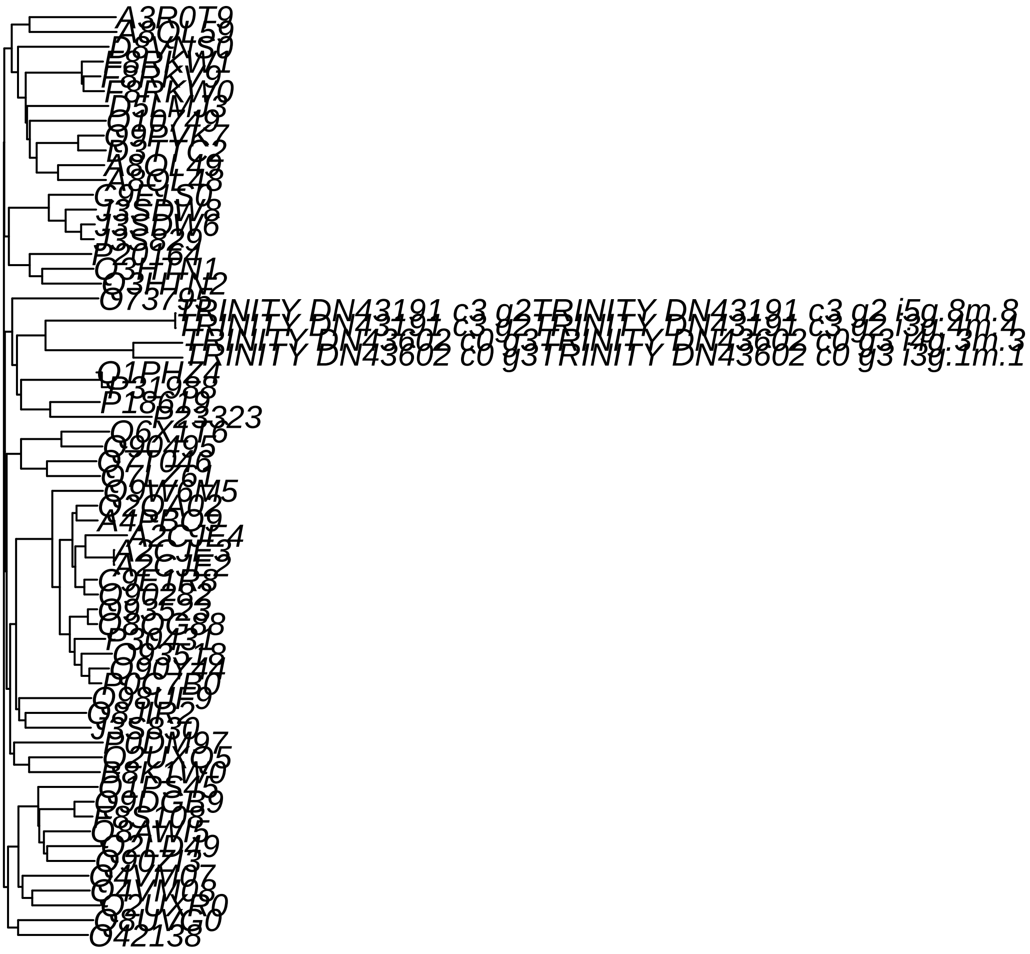
A3R0T9
A8QL59
D8VNS0
F8RKW1
F8RKW0
D5LMJ3
Q1B749
Q9PVK7
D3TTC2
A8QL49
A8QL48
G9EIS0
J3SDW8
J3SDW6
J3S829
P20164
C3HTN1
C3HTN2
O73795
TRINITY DN43191 c3 g2TRINITY DN43191 c3 g2 i5g.8m.8
TRINITY DN43191 c3 g2TRINITY DN43191 c3 g2 i3g.4m.4
TRINITY DN43602 c0 g3TRINITY DN43602 c0 g3 i4g.3m.3
TRINITY DN43602 c0 g3TRINITY DN43602 c0 g3 i3g.1m.1
Q1PHZ4
P31988
P18619
P23323
Q6X1T6
Q90495
Q7T046
Q9W6M5
A4RBQ9
A2CJE4
A2CJE3
A2CJE2
P30431
Q93518
P0C7B0
Q8JIR2
J3S830
P0DM97
B8K1W0
Q9DGB9
Q4VM07
Q4VM08
Q2UXR0
Q8UVG0
O42138
