# Supplementary material for: Venomix: a simple bioinformatic pipeline for identifying and characterizing toxin gene candidates from transcriptomic data
Source: PeerJ. 2018 Jul 31;6:e5361. doi: 10.7717/peerj.5361 (PMC6074769; doi:10.7717/peerj.5361)
Supplement: Supplemental Information 4 [file peerj-06-5361-s004.gz › FinalOutput_E-20/Snake_venom_metalloproteinase_fibrolase_19/finaltree.pdf]

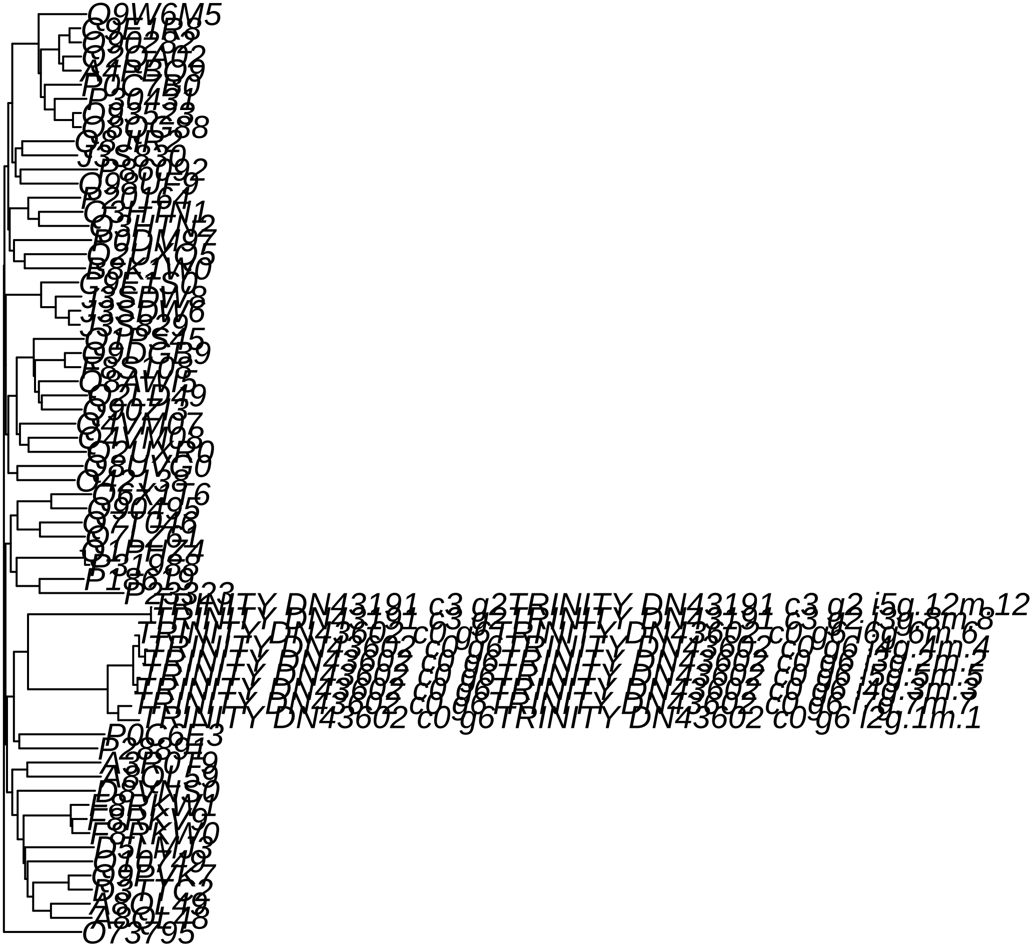
Q9W6M5
TRINITY DN43191 c3 g2TRINITY DN43191 c3 g2 i5g.12m.12
TRINITY DN43602 c0 g6TRINITY DN43602 c0 g6 i2g.1m.1
P0C6E3
O73795
