# Supplementary material for: Venomix: a simple bioinformatic pipeline for identifying and characterizing toxin gene candidates from transcriptomic data
Source: PeerJ. 2018 Jul 31;6:e5361. doi: 10.7717/peerj.5361 (PMC6074769; doi:10.7717/peerj.5361)
Supplement: Supplemental Information 4 [file peerj-06-5361-s004.gz › FinalOutput_E-20/Kunitz-type_serine_protease_inhibitor_bitisilin-3_1/finaltree.pdf]

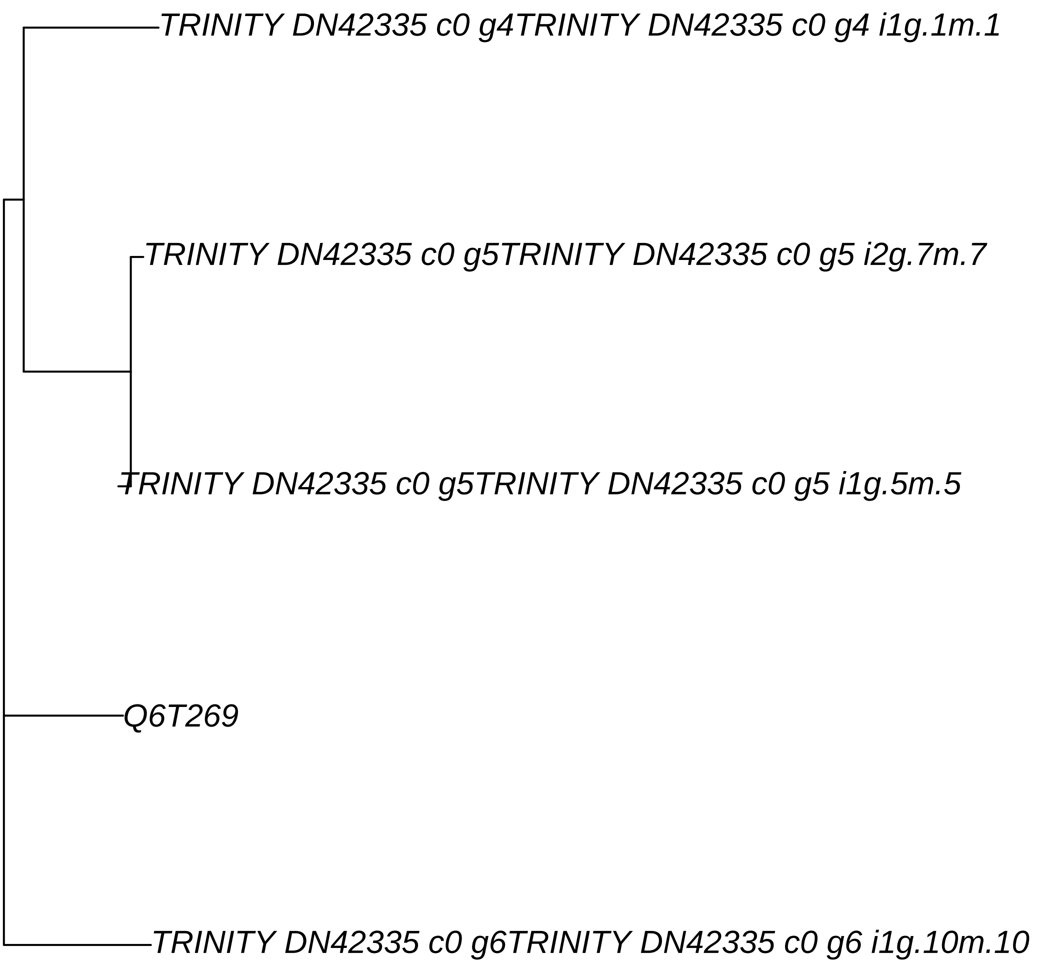
TRINITY DN42335 c0 g4TRINITY DN42335 c0 g4 i1g.1m.1
TRINITY DN42335 c0 g5TRINITY DN42335 c0 g5 i2g.7m.7
TRINITY DN42335 c0 g5TRINITY DN42335 c0 g5 i1g.5m.5
Q6T269
TRINITY DN42335 c0 g6TRINITY DN42335 c0 g6 i1g.10m.10
