# Supplementary material for: Venomix: a simple bioinformatic pipeline for identifying and characterizing toxin gene candidates from transcriptomic data
Source: PeerJ. 2018 Jul 31;6:e5361. doi: 10.7717/peerj.5361 (PMC6074769; doi:10.7717/peerj.5361)
Supplement: Supplemental Information 4 [file peerj-06-5361-s004.gz › FinalOutput_E-20/Astacin-like_metalloprotease_toxin_3_2/finaltree.pdf]

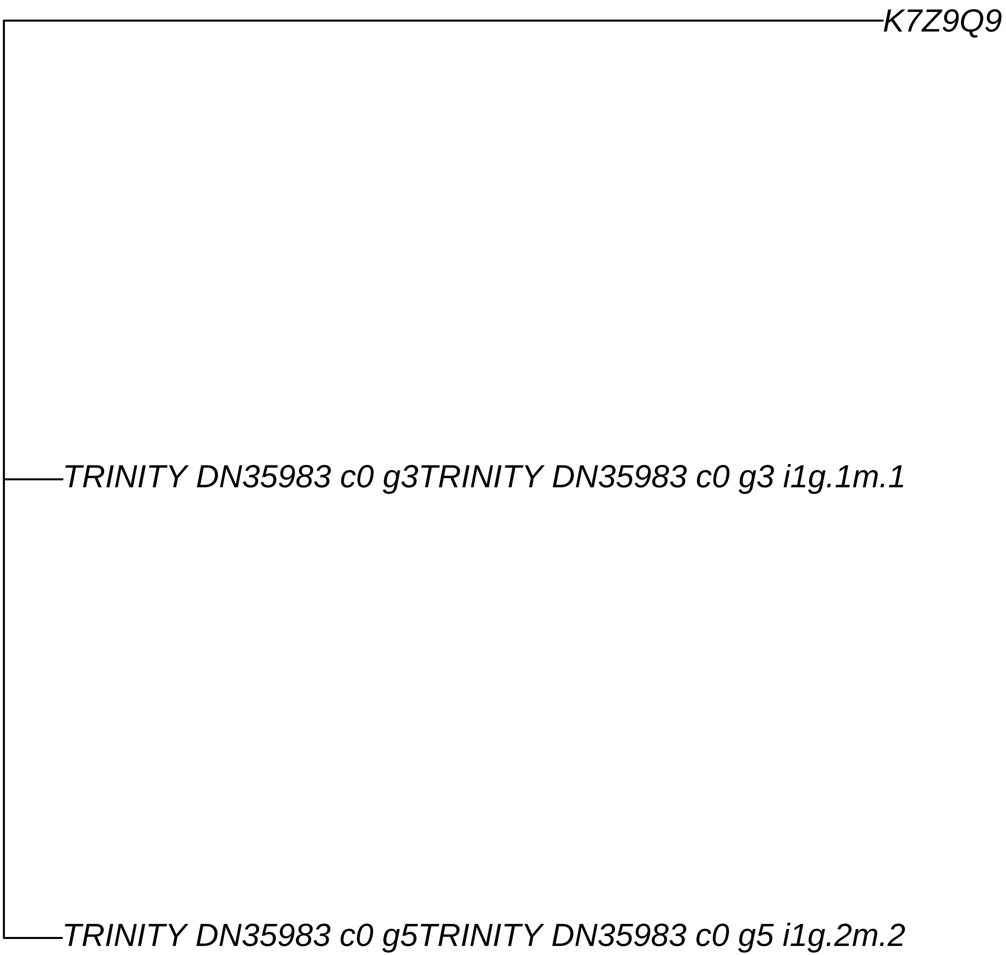
K7Z9Q9
TRINITY DN35983 c0 g3TRINITY DN35983 c0 g3 i1g.1m.1
TRINITY DN35983 c0 g5TRINITY DN35983 c0 g5 i1g.2m.2
