# Supplementary material for: Venomix: a simple bioinformatic pipeline for identifying and characterizing toxin gene candidates from transcriptomic data
Source: PeerJ. 2018 Jul 31;6:e5361. doi: 10.7717/peerj.5361 (PMC6074769; doi:10.7717/peerj.5361)
Supplement: Supplemental Information 4 [file peerj-06-5361-s004.gz › FinalOutput_E-20/Snake_venom_metalloproteinase_Ac1_1/finaltree.pdf]

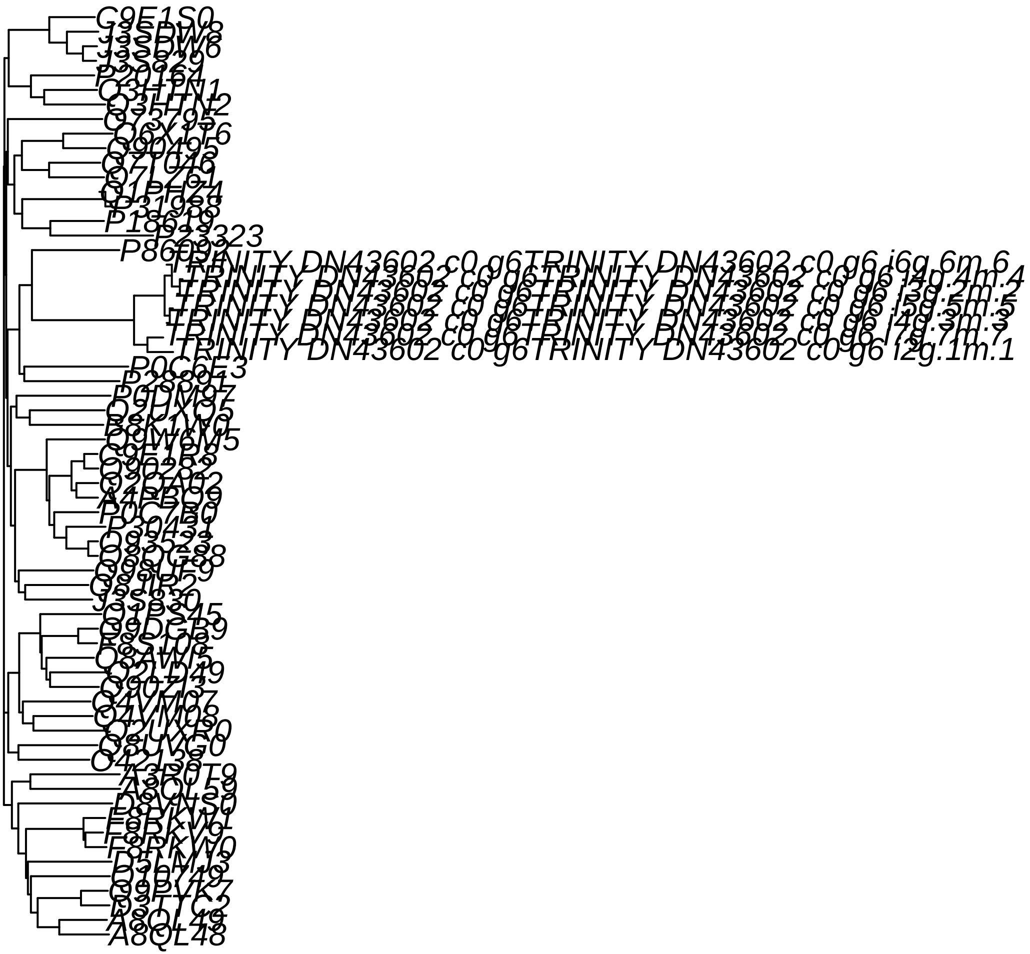
G9E1S0
J3SDW8
J3SDW6
J3S829
P20164
Q3HTN1
Q3HTN2
Q73795
Q6X1T6
Q90495
Q7T046
Q7L261
Q1PHZ4
P31988
P18619
P23323
P86091
TRINITY DN43602 c0 g6TRINITY DN43602 c0 g6 i6g.6m.6
TRINITY DN43602 c0 g6TRINITY DN43602 c0 g6 i4g.4m.4
TRINITY DN43602 c0 g6TRINITY DN43602 c0 g6 i3g.2m.2
TRINITY DN43602 c0 g6TRINITY DN43602 c0 g6 i5g.5m.5
TRINITY DN43602 c0 g6TRINITY DN43602 c0 g6 i4g.3m.3
TRINITY DN43602 c0 g6TRINITY DN43602 c0 g6 i7g.7m.7
TRINITY DN43602 c0 g6TRINITY DN43602 c0 g6 i2g.1m.1
P0C6E3
P28891
P0DM97
Q2UX05
B8K1W0
Q9W6M5
A4PBQ9
P0C7B0
J3S830
Q1PS45
Q9DGB9
Q2LD49
Q4VM07
Q4VM08
Q2UXR0
Q8UVG0
A3R0T9
A8QL59
D8VNS0
F8RKW1
F8RKV9
F8RKW0
D5LMJ3
Q9PVK7
A8QL49
A8QL48
