# Supplementary material for: Venomix: a simple bioinformatic pipeline for identifying and characterizing toxin gene candidates from transcriptomic data
Source: PeerJ. 2018 Jul 31;6:e5361. doi: 10.7717/peerj.5361 (PMC6074769; doi:10.7717/peerj.5361)
Supplement: Supplemental Information 4 [file peerj-06-5361-s004.gz › FinalOutput_E-20/Venom_allergen_3_30/finaltree.pdf]

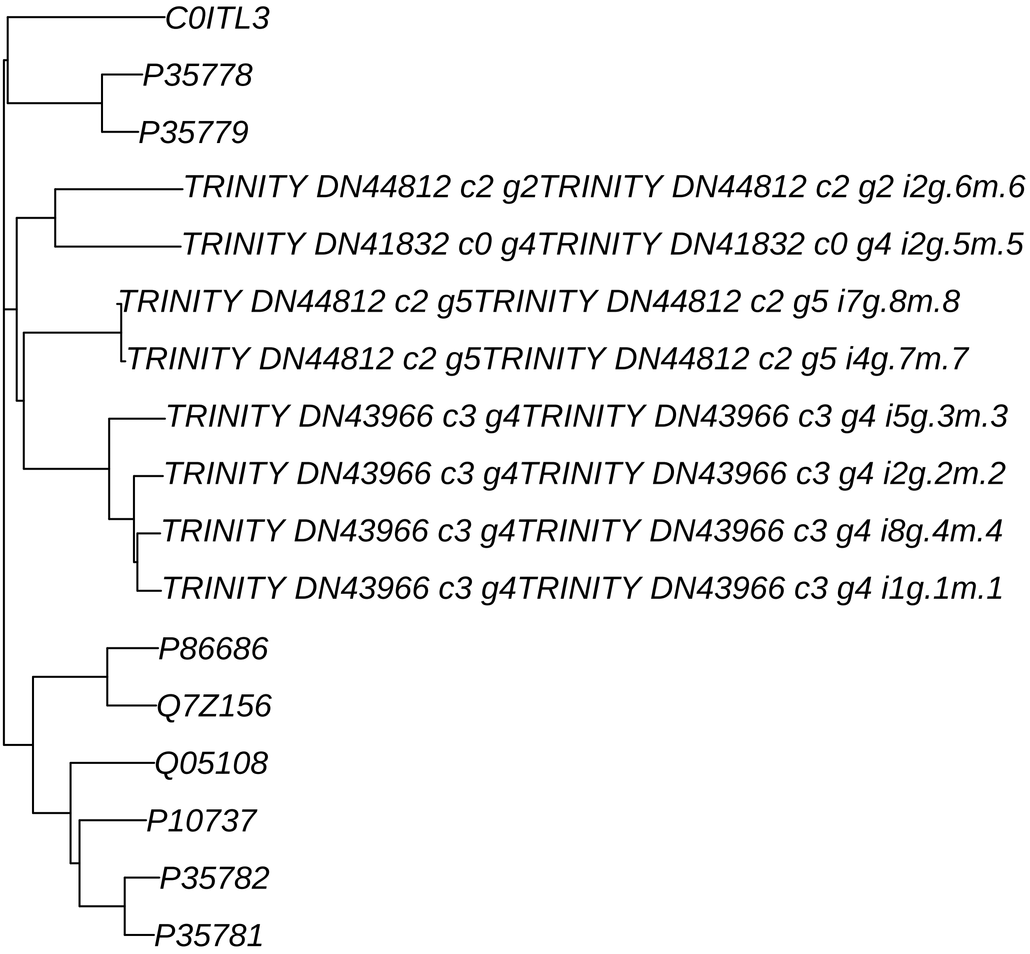
C0ITL3
P35778
P35779
TRINITY DN44812 c2 g2TRINITY DN44812 c2 g2 i2g.6m.6
TRINITY DN41832 c0 g4TRINITY DN41832 c0 g4 i2g.5m.5
TRINITY DN44812 c2 g5TRINITY DN44812 c2 g5 i7g.8m.8
TRINITY DN44812 c2 g5TRINITY DN44812 c2 g5 i4g.7m.7
TRINITY DN43966 c3 g4TRINITY DN43966 c3 g4 i5g.3m.3
TRINITY DN43966 c3 g4TRINITY DN43966 c3 g4 i2g.2m.2
TRINITY DN43966 c3 g4TRINITY DN43966 c3 g4 i8g.4m.4
TRINITY DN43966 c3 g4TRINITY DN43966 c3 g4 i1g.1m.1
P86686
Q7Z156
Q05108
P10737
P35782
P35781
