# Supplementary material for: Venomix: a simple bioinformatic pipeline for identifying and characterizing toxin gene candidates from transcriptomic data
Source: PeerJ. 2018 Jul 31;6:e5361. doi: 10.7717/peerj.5361 (PMC6074769; doi:10.7717/peerj.5361)
Supplement: Supplemental Information 4 [file peerj-06-5361-s004.gz › FinalOutput_E-20/Acetylcholinesterase-1_1/finaltree.pdf]

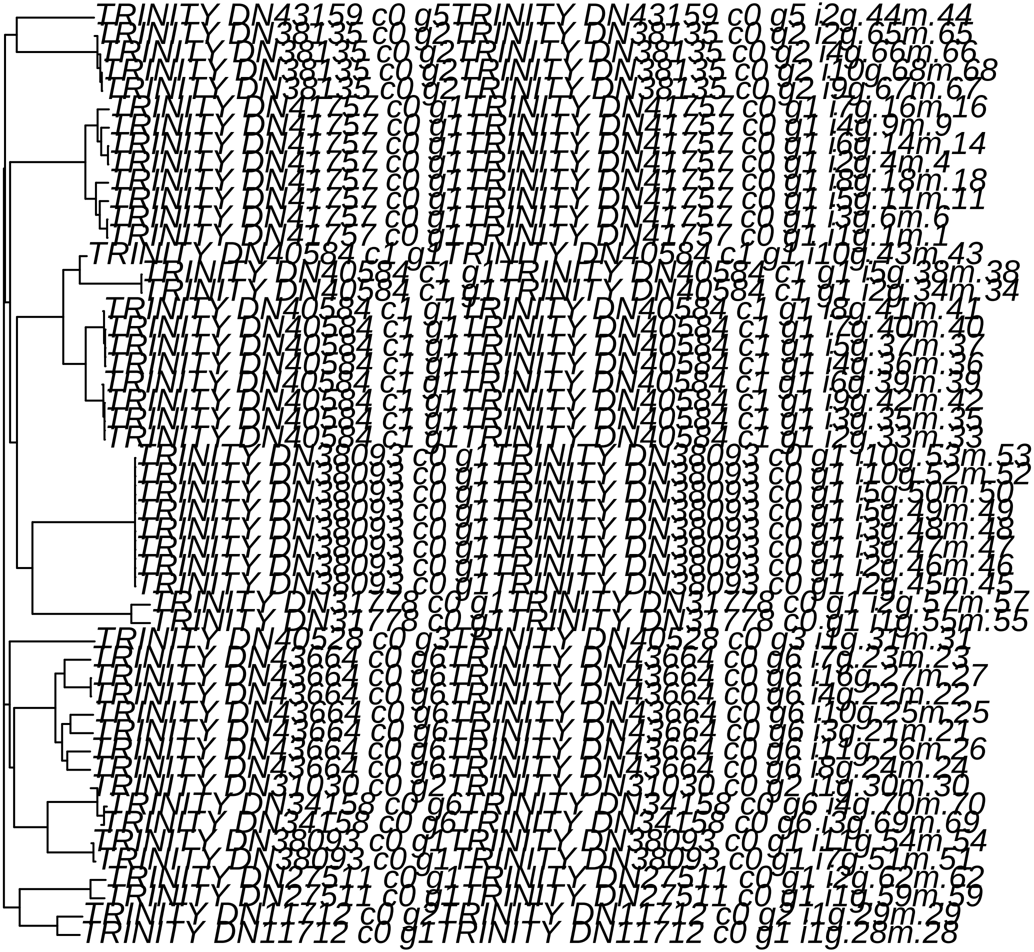
TRINITY DN43159 c0 g5TRINITY DN43159 c0 g5 i2g.44m.44
TRINITY DN38135 c0 g2TRINITY DN38135 c0 g2 i2g.65m.65
TRINITY DN38135 c0 g2TRINITY DN38135 c0 g2 i4g.66m.66
TRINITY DN38135 c0 g2TRINITY DN38135 c0 g2 i10g.68m.68
TRINITY DN38135 c0 g2TRINITY DN38135 c0 g2 i9g.67m.67
TRINITY DN41757 c0 g1TRINITY DN41757 c0 g1 i7g.16m.16
TRINITY DN41757 c0 g1TRINITY DN41757 c0 g1 i4g.9m.9
TRINITY DN41757 c0 g1TRINITY DN41757 c0 g1 i6g.14m.14
TRINITY DN41757 c0 g1TRINITY DN41757 c0 g1 i2g.4m.4
TRINITY DN41757 c0 g1TRINITY DN41757 c0 g1 i8g.18m.18
TRINITY DN41757 c0 g1TRINITY DN41757 c0 g1 i5g.11m.11
TRINITY DN41757 c0 g1TRINITY DN41757 c0 g1 i3g.6m.6
TRINITY DN41757 c0 g1TRINITY DN41757 c0 g1 i1g.1m.1
TRINITY DN40584 c1 g1TRINITY DN40584 c1 g1 i10g.43m.43
TRINITY DN40584 c1 g1TRINITY DN40584 c1 g1 i5g.38m.38
TRINITY DN40584 c1 g1TRINITY DN40584 c1 g1 i2g.34m.34
TRINITY DN40584 c1 g1TRINITY DN40584 c1 g1 i8g.41m.41
TRINITY DN40584 c1 g1TRINITY DN40584 c1 g1 i7g.40m.40
TRINITY DN40584 c1 g1TRINITY DN40584 c1 g1 i5g.37m.37
TRINITY DN40584 c1 g1TRINITY DN40584 c1 g1 i4g.36m.36
TRINITY DN40584 c1 g1TRINITY DN40584 c1 g1 i6g.39m.39
TRINITY DN40584 c1 g1TRINITY DN40584 c1 g1 i9g.42m.42
TRINITY DN40584 c1 g1TRINITY DN40584 c1 g1 i3g.35m.35
TRINITY DN40584 c1 g1TRINITY DN40584 c1 g1 i2g.33m.33
TRINITY DN38093 c0 g1TRINITY DN38093 c0 g1 i10g.53m.53
TRINITY DN38093 c0 g1TRINITY DN38093 c0 g1 i10g.52m.52
TRINITY DN38093 c0 g1TRINITY DN38093 c0 g1 i5g.50m.50
TRINITY DN38093 c0 g1TRINITY DN38093 c0 g1 i5g.49m.49
TRINITY DN38093 c0 g1TRINITY DN38093 c0 g1 i3g.48m.48
TRINITY DN38093 c0 g1TRINITY DN38093 c0 g1 i3g.47m.47
TRINITY DN38093 c0 g1TRINITY DN38093 c0 g1 i2g.46m.46
TRINITY DN38093 c0 g1TRINITY DN38093 c0 g1 i2g.45m.45
TRINITY DN31778 c0 g1TRINITY DN31778 c0 g1 i2g.57m.57
TRINITY DN31778 c0 g1TRINITY DN31778 c0 g1 i1g.55m.55
TRINITY DN40528 c0 g3TRINITY DN40528 c0 g3 i1g.31m.31
TRINITY DN43664 c0 g6TRINITY DN43664 c0 g6 i7g.23m.23
TRINITY DN43664 c0 g6TRINITY DN43664 c0 g6 i16g.27m.27
TRINITY DN43664 c0 g6TRINITY DN43664 c0 g6 i4g.22m.22
TRINITY DN43664 c0 g6TRINITY DN43664 c0 g6 i10g.25m.25
TRINITY DN43664 c0 g6TRINITY DN43664 c0 g6 i3g.21m.21
TRINITY DN43664 c0 g6TRINITY DN43664 c0 g6 i11g.26m.26
TRINITY DN43664 c0 g6TRINITY DN43664 c0 g6 i8g.24m.24
TRINITY DN31030 c0 g2TRINITY DN31030 c0 g2 i1g.30m.30
TRINITY DN34158 c0 g6TRINITY DN34158 c0 g6 i4g.70m.70
TRINITY DN34158 c0 g6TRINITY DN34158 c0 g6 i3g.69m.69
TRINITY DN38093 c0 g1TRINITY DN38093 c0 g1 i11g.54m.54
TRINITY DN38093 c0 g1TRINITY DN38093 c0 g1 i7g.51m.51
TRINITY DN27511 c0 g1TRINITY DN27511 c0 g1 i2g.62m.62
TRINITY DN27511 c0 g1TRINITY DN27511 c0 g1 i1g.59m.59
TRINITY DN11712 c0 g2TRINITY DN11712 c0 g2 i1g.29m.29
TRINITY DN11712 c0 g1TRINITY DN11712 c0 g1 i1g.28m.28
