# Supplementary material for: Venomix: a simple bioinformatic pipeline for identifying and characterizing toxin gene candidates from transcriptomic data
Source: PeerJ. 2018 Jul 31;6:e5361. doi: 10.7717/peerj.5361 (PMC6074769; doi:10.7717/peerj.5361)
Supplement: Supplemental Information 4 [file peerj-06-5361-s004.gz › FinalOutput_E-20/Neprilysin-1_1/finaltree.pdf]

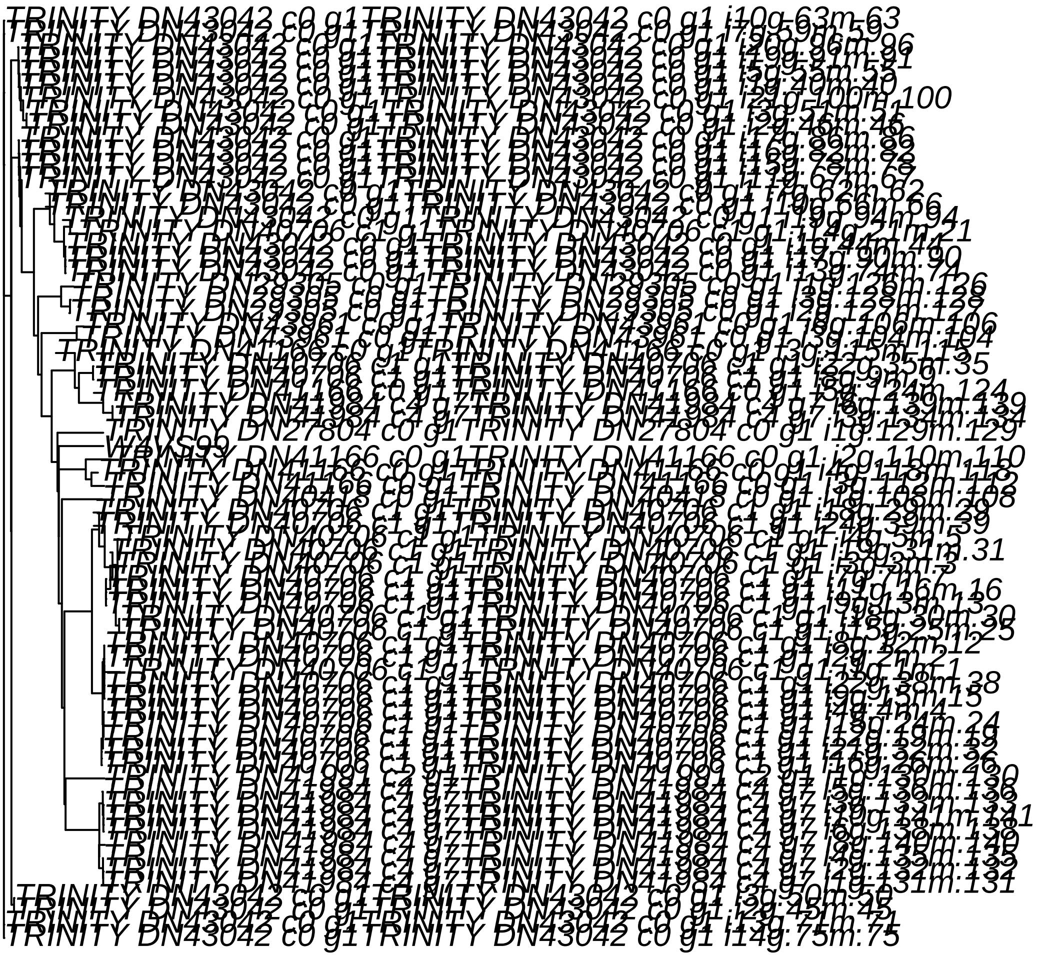
TRINITY DN43042 c0 g1TRINITY DN43042 c0 g1 i10g.63m.63
TRINITY DN27804 c0 g1TRINITY DN27804 c0 g1 i1g.129m.129
TRINITY DN43042 c0 g1TRINITY DN43042 c0 g1 i14g.75m.75
