# Supplementary material for: Venomix: a simple bioinformatic pipeline for identifying and characterizing toxin gene candidates from transcriptomic data
Source: PeerJ. 2018 Jul 31;6:e5361. doi: 10.7717/peerj.5361 (PMC6074769; doi:10.7717/peerj.5361)
Supplement: Supplemental Information 4 [file peerj-06-5361-s004.gz › FinalOutput_E-20/Plancitoxin-1_1/finaltree.pdf]

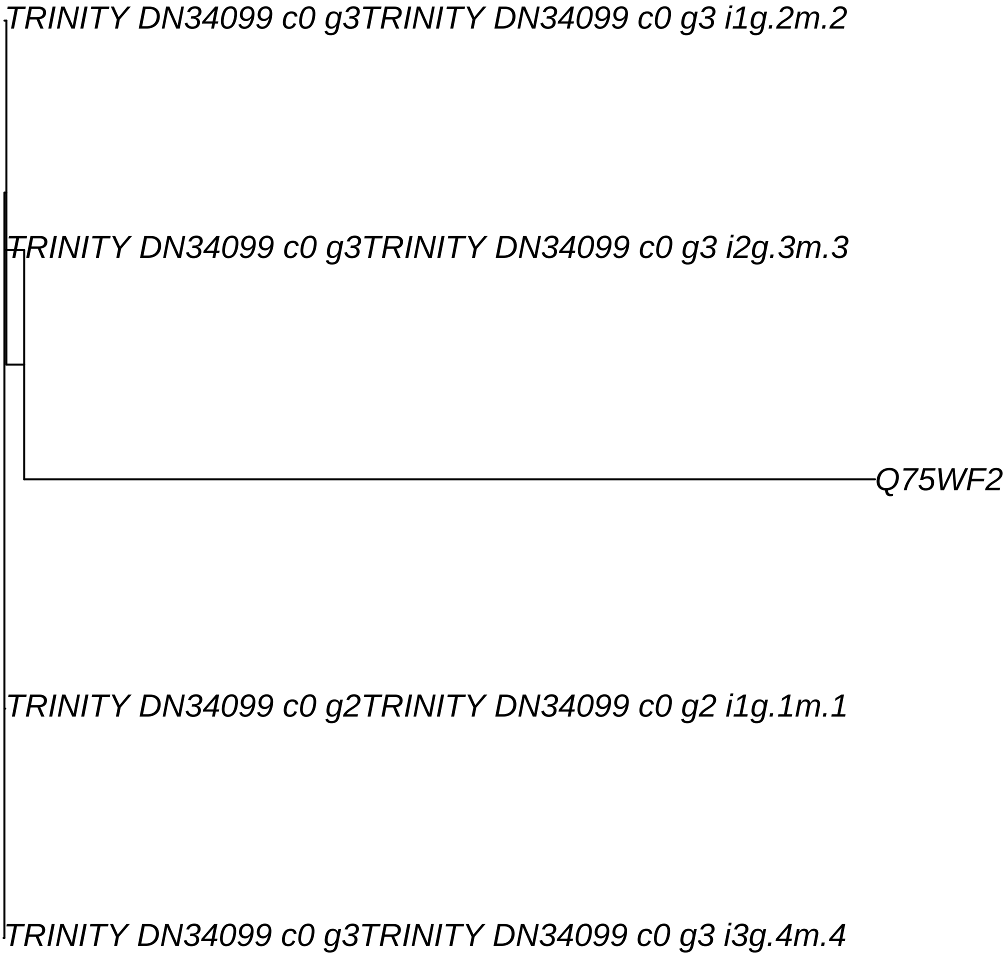
TRINITY DN34099 c0 g3TRINITY DN34099 c0 g3 i1g.2m.2
TRINITY DN34099 c0 g3TRINITY DN34099 c0 g3 i2g.3m.3
Q75WF2
TRINITY DN34099 c0 g2TRINITY DN34099 c0 g2 i1g.1m.1
TRINITY DN34099 c0 g3TRINITY DN34099 c0 g3 i3g.4m.4
