# Supplementary material for: Venomix: a simple bioinformatic pipeline for identifying and characterizing toxin gene candidates from transcriptomic data
Source: PeerJ. 2018 Jul 31;6:e5361. doi: 10.7717/peerj.5361 (PMC6074769; doi:10.7717/peerj.5361)
Supplement: Supplemental Information 4 [file peerj-06-5361-s004.gz › FinalOutput_E-20/Techylectin-like_protein_1/finaltree.pdf]

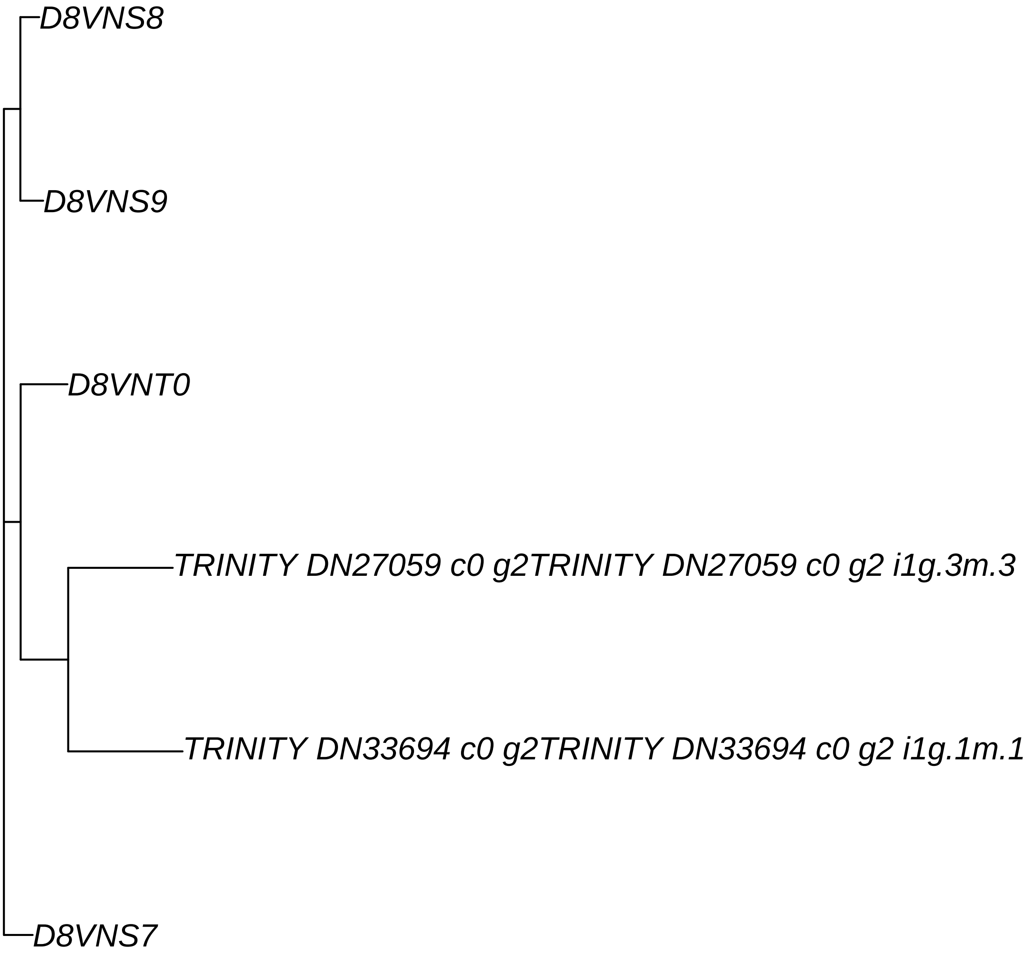
D8VNS8
D8VNS9
D8VNT0
TRINITY DN27059 c0 g2TRINITY DN27059 c0 g2 i1g.3m.3
TRINITY DN33694 c0 g2TRINITY DN33694 c0 g2 i1g.1m.1
D8VNS7
