# Supplementary material for: Venomix: a simple bioinformatic pipeline for identifying and characterizing toxin gene candidates from transcriptomic data
Source: PeerJ. 2018 Jul 31;6:e5361. doi: 10.7717/peerj.5361 (PMC6074769; doi:10.7717/peerj.5361)
Supplement: Supplemental Information 4 [file peerj-06-5361-s004.gz › FinalOutput_E-6/Zinc_metalloproteinase-disintegrin-like_VAP2B_272/finaltree.pdf]

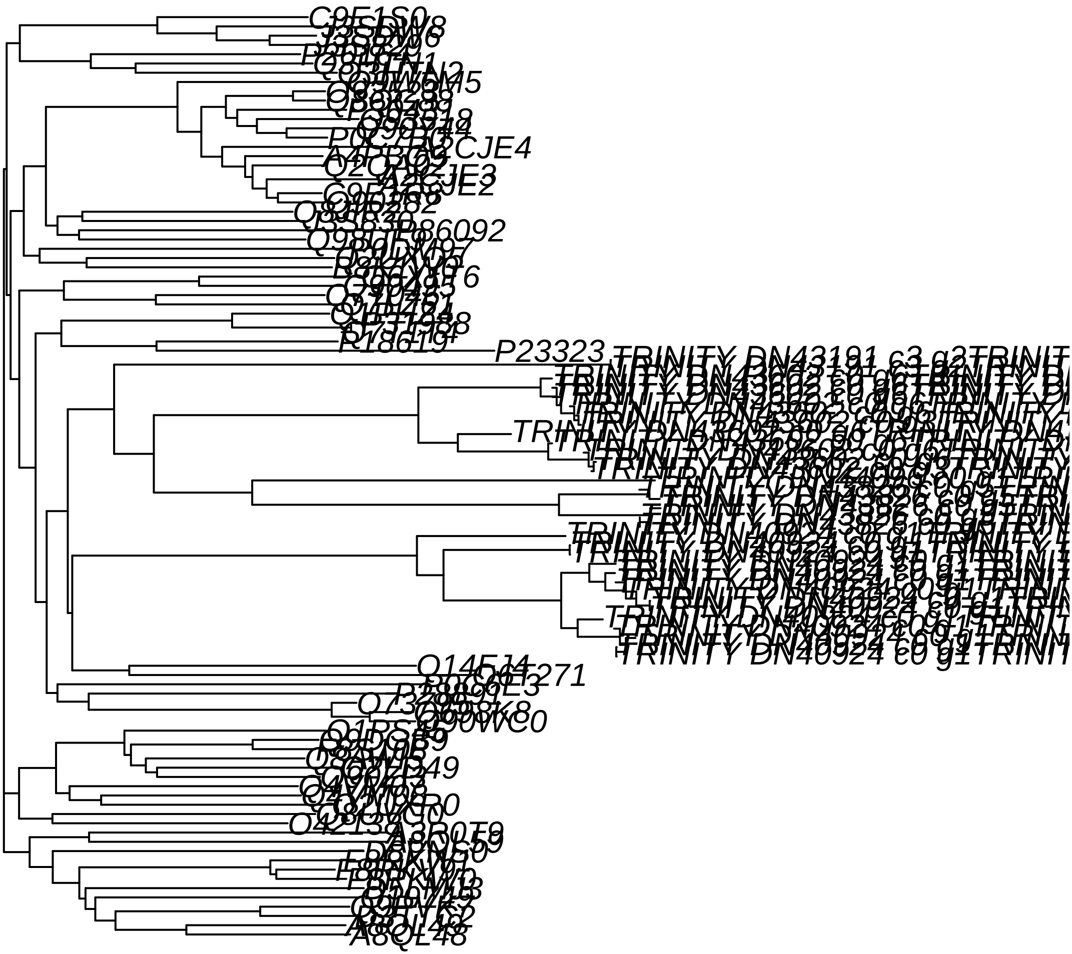

C9E1S0
P23323
Q14514
A8QL48
TRINITY DN40924 c0 g1
