# Supplementary material for: Venomix: a simple bioinformatic pipeline for identifying and characterizing toxin gene candidates from transcriptomic data
Source: PeerJ. 2018 Jul 31;6:e5361. doi: 10.7717/peerj.5361 (PMC6074769; doi:10.7717/peerj.5361)
Supplement: Supplemental Information 4 [file peerj-06-5361-s004.gz › FinalOutput_E-6/Kunitz-type_serine_protease_inhibitor_homolog_dendrotoxin_K_154/finaltree.pdf]

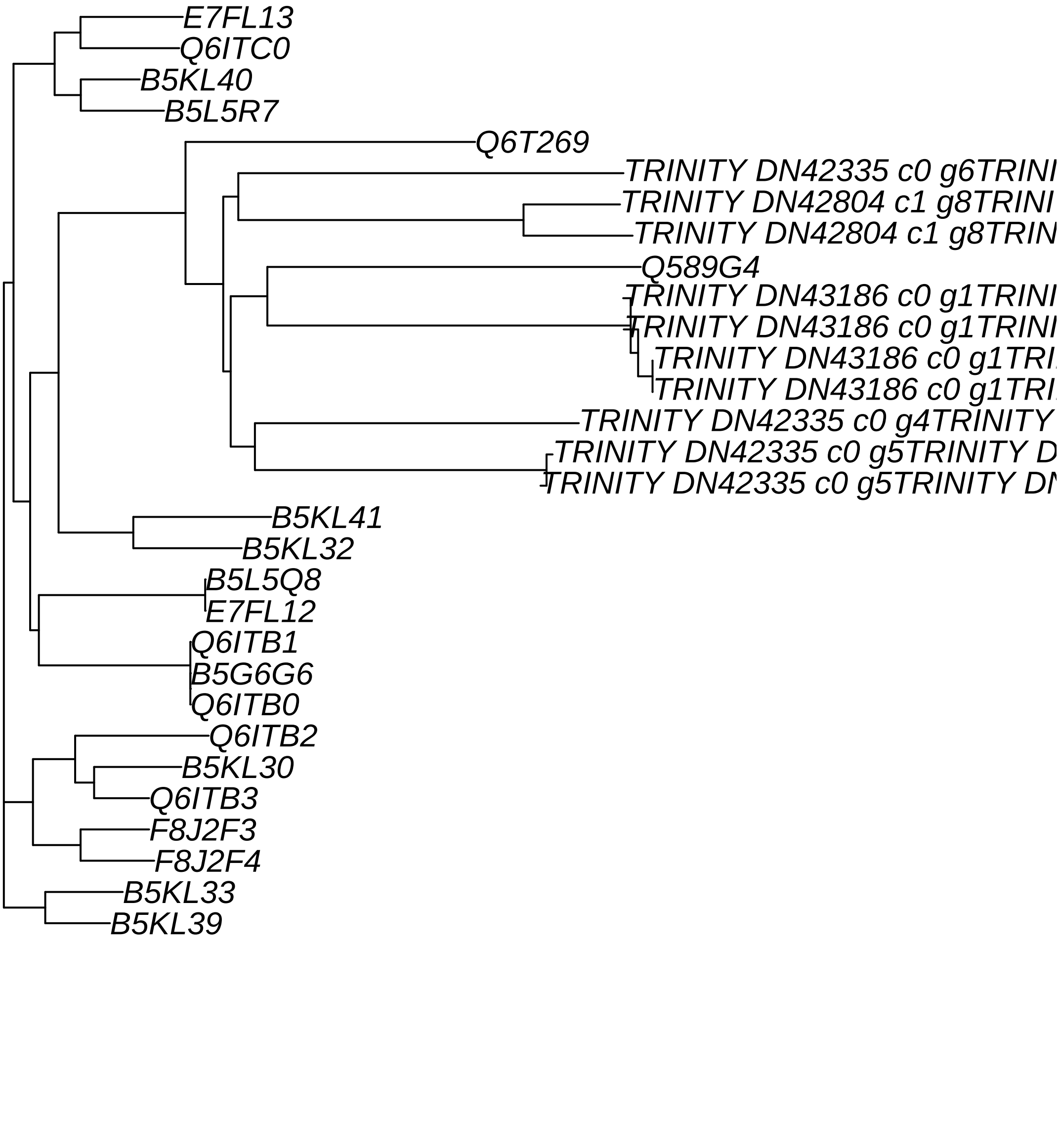
E7FL13
Q6ITC0
B5KL40
B5L5R7
Q6T269
TRINITY DN42335 c0 g6TRINI
TRINITY DN42804 c1 g8TRINI
TRINITY DN42804 c1 g8TRIN
Q589G4
TRINITY DN43186 c0 g1TRINI
TRINITY DN43186 c0 g1TRINI
TRINITY DN43186 c0 g1TRI
TRINITY DN43186 c0 g1TRI
TRINITY DN42335 c0 g4TRINITY
TRINITY DN42335 c0 g5TRINITY D
TRINITY DN42335 c0 g5TRINITY D
B5KL41
B5KL32
B5L5Q8
E7FL12
Q6ITB1
B5G6G6
Q6ITB0
Q6ITB2
B5KL30
Q6ITB3
F8J2F3
F8J2F4
B5KL33
B5KL39
