# Supplementary material for: Venomix: a simple bioinformatic pipeline for identifying and characterizing toxin gene candidates from transcriptomic data
Source: PeerJ. 2018 Jul 31;6:e5361. doi: 10.7717/peerj.5361 (PMC6074769; doi:10.7717/peerj.5361)
Supplement: Supplemental Information 4 [file peerj-06-5361-s004.gz › FinalOutput_E-6/Kunitz-type_serine_protease_inhibitor_conotoxin_Cal9/finaltree.pdf]

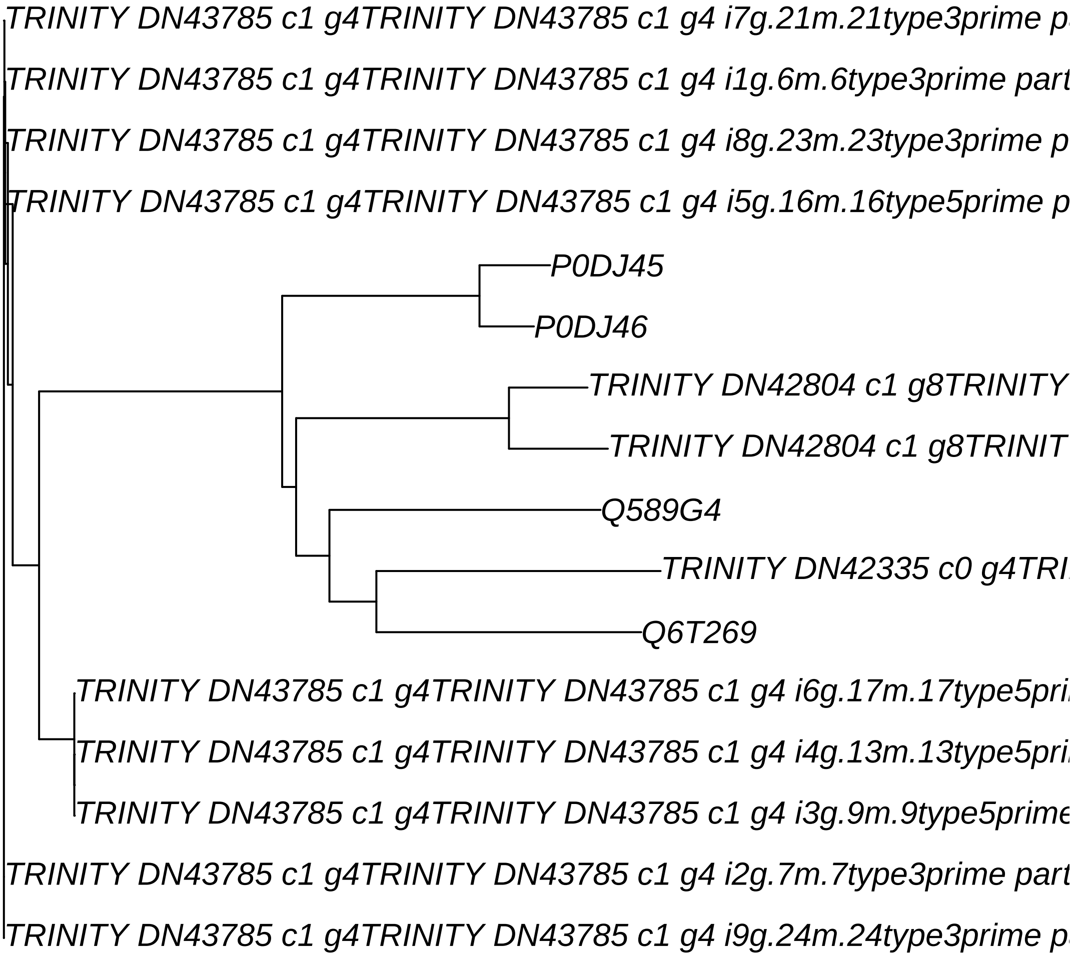
TRINITY DN43785 c1 g4TRINITY DN43785 c1 g4 i7g.21m.21type3prime p
TRINITY DN43785 c1 g4TRINITY DN43785 c1 g4 i1g.6m.6type3prime part
TRINITY DN43785 c1 g4TRINITY DN43785 c1 g4 i8g.23m.23type3prime p
TRINITY DN43785 c1 g4TRINITY DN43785 c1 g4 i5g.16m.16type5prime p
P0DJ45
P0DJ46
TRINITY DN42804 c1 g8TRINITY
TRINITY DN42804 c1 g8TRINIT
Q589G4
TRINITY DN42335 c0 g4TRI
Q6T269
TRINITY DN43785 c1 g4TRINITY DN43785 c1 g4 i6g.17m.17type5pri
TRINITY DN43785 c1 g4TRINITY DN43785 c1 g4 i4g.13m.13type5pri
TRINITY DN43785 c1 g4TRINITY DN43785 c1 g4 i3g.9m.9type5prime
TRINITY DN43785 c1 g4TRINITY DN43785 c1 g4 i2g.7m.7type3prime part
TRINITY DN43785 c1 g4TRINITY DN43785 c1 g4 i9g.24m.24type3prime p
