# Supplementary material for: Venomix: a simple bioinformatic pipeline for identifying and characterizing toxin gene candidates from transcriptomic data
Source: PeerJ. 2018 Jul 31;6:e5361. doi: 10.7717/peerj.5361 (PMC6074769; doi:10.7717/peerj.5361)
Supplement: Supplemental Information 4 [file peerj-06-5361-s004.gz › FinalOutput_E-6/Factor_V_activator_RVV-V_gamma_142/finaltree.pdf]

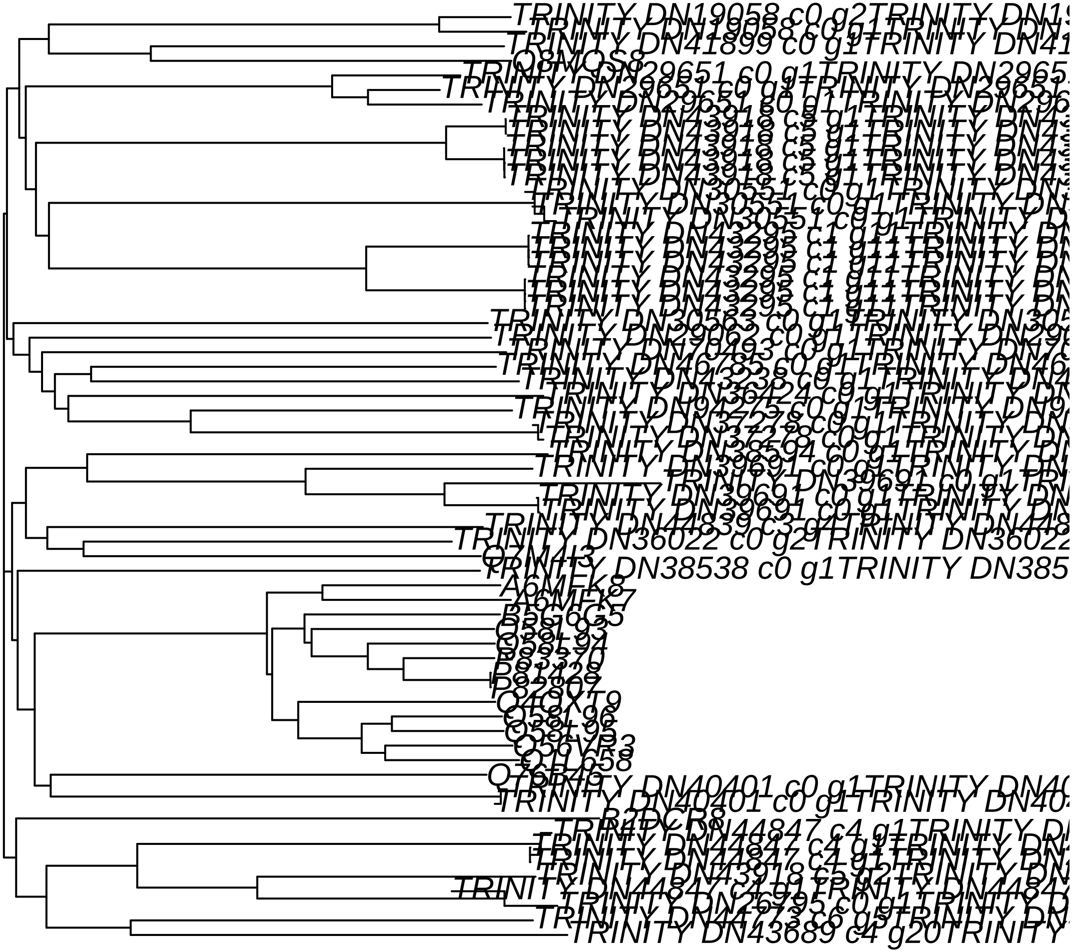
TRINITY DN36022 c0 g2TRINITY DN36022
TRINITY DN38538 c0 g1TRINITY DN385
TRINITY DN43689 c4 g20TRINITY
