# Supplementary material for: Venomix: a simple bioinformatic pipeline for identifying and characterizing toxin gene candidates from transcriptomic data
Source: PeerJ. 2018 Jul 31;6:e5361. doi: 10.7717/peerj.5361 (PMC6074769; doi:10.7717/peerj.5361)
Supplement: Supplemental Information 4 [file peerj-06-5361-s004.gz › FinalOutput_E-6/Thrombin-like_enzyme_TLBm_1/finaltree.pdf]

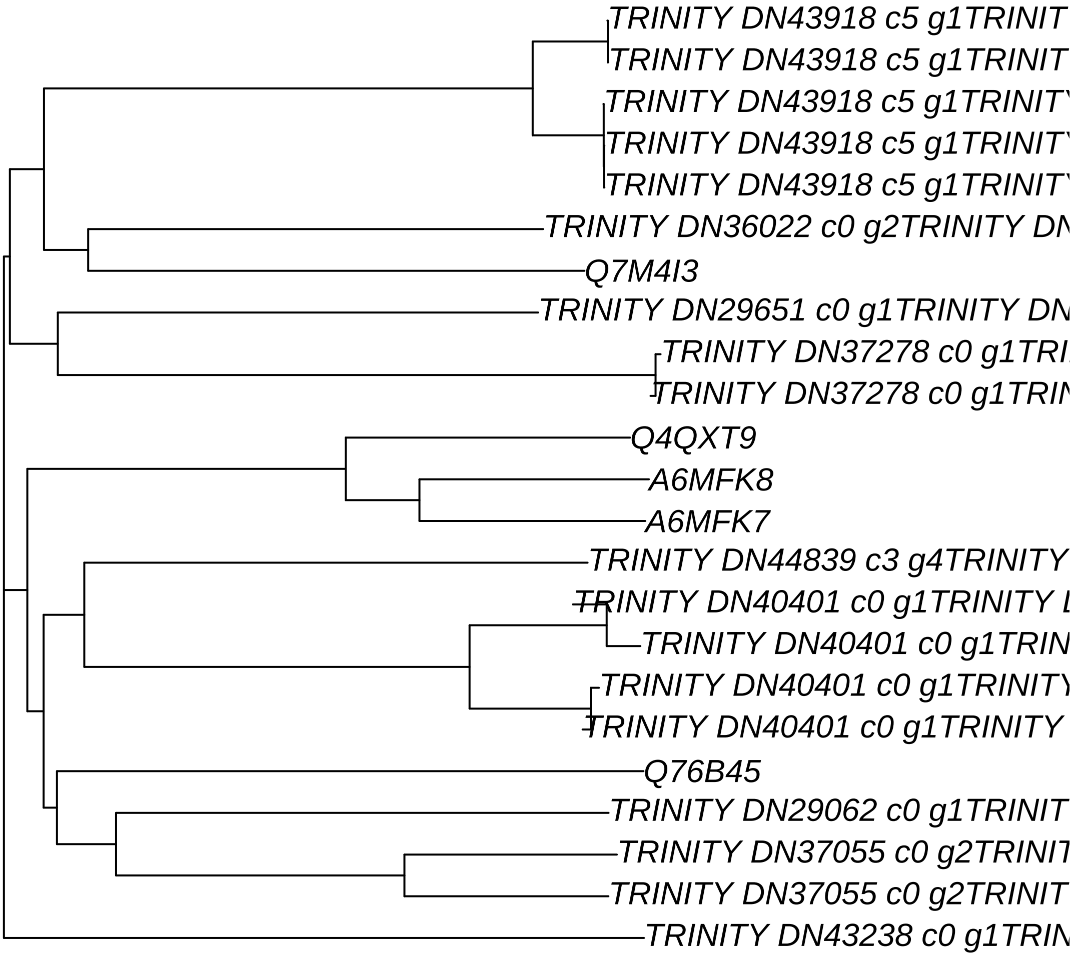
TRINITY DN43918 c5 g1TRINIT
TRINITY DN43918 c5 g1TRINIT
TRINITY DN43918 c5 g1TRINITY
TRINITY DN43918 c5 g1TRINITY
TRINITY DN43918 c5 g1TRINITY
TRINITY DN36022 c0 g2TRINITY DN
Q7M4I3
TRINITY DN29651 c0 g1TRINITY DN
TRINITY DN37278 c0 g1TRI
TRINITY DN37278 c0 g1TRIN
Q4QXT9
A6MFK8
A6MFK7
TRINITY DN44839 c3 g4TRINITY
TRINITY DN40401 c0 g1TRINITY
TRINITY DN40401 c0 g1TRIN
TRINITY DN40401 c0 g1TRINITY
TRINITY DN40401 c0 g1TRINITY
Q76B45
TRINITY DN29062 c0 g1TRINIT
TRINITY DN37055 c0 g2TRINIT
TRINITY DN37055 c0 g2TRINIT
TRINITY DN43238 c0 g1TRIN
