# Supplementary material for: Venomix: a simple bioinformatic pipeline for identifying and characterizing toxin gene candidates from transcriptomic data
Source: PeerJ. 2018 Jul 31;6:e5361. doi: 10.7717/peerj.5361 (PMC6074769; doi:10.7717/peerj.5361)
Supplement: Supplemental Information 4 [file peerj-06-5361-s004.gz › FinalOutput_E-6/Protease_inhibitor_1_6/finaltree.pdf]

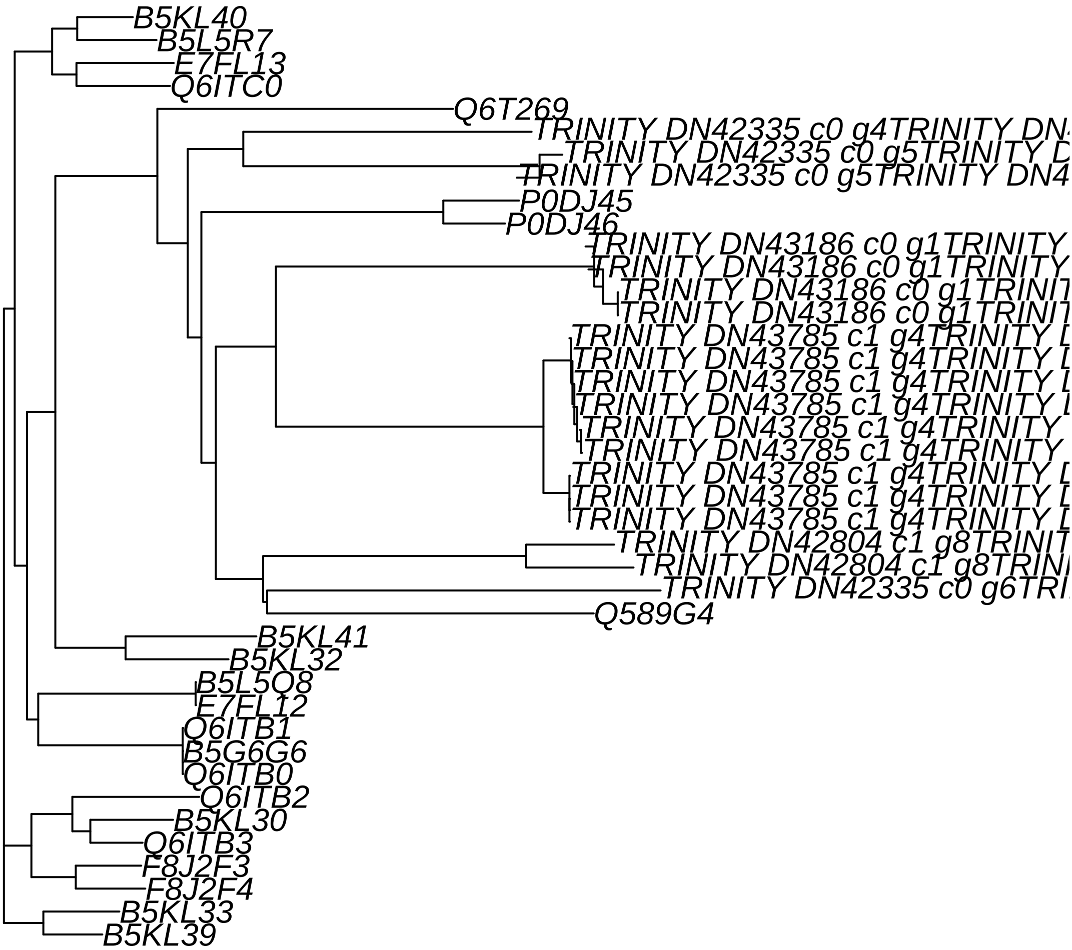
B5KL40
B5L5R7
E7FL13
Q6ITC0
Q6T269
TRINITY DN42335 c0 g4TRINITY DN4
TRINITY DN42335 c0 g5TRINITY D
TRINITY DN42335 c0 g5TRINITY DN4
P0DJ45
P0DJ46
TRINITY DN43186 c0 g1TRINITY
TRINITY DN43186 c0 g1TRINITY
TRINITY DN43186 c0 g1TRINIT
TRINITY DN43186 c0 g1TRINIT
TRINITY DN43785 c1 g4TRINITY D
TRINITY DN43785 c1 g4TRINITY D
TRINITY DN43785 c1 g4TRINITY D
TRINITY DN43785 c1 g4TRINITY D
TRINITY DN43785 c1 g4TRINITY
TRINITY DN43785 c1 g4TRINITY
TRINITY DN43785 c1 g4TRINITY D
TRINITY DN43785 c1 g4TRINITY D
TRINITY DN43785 c1 g4TRINITY D
TRINITY DN42804 c1 g8TRINIT
TRINITY DN42804 c1 g8TRIN
TRINITY DN42335 c0 g6TRI
Q589G4
B5KL41
B5KL32
B5L5Q8
E7FL12
Q6ITB1
B5G6G6
Q6ITB0
Q6ITB2
B5KL30
Q6ITB3
F8J2F3
F8J2F4
B5KL33
B5KL39
