# Supplementary material for: Venomix: a simple bioinformatic pipeline for identifying and characterizing toxin gene candidates from transcriptomic data
Source: PeerJ. 2018 Jul 31;6:e5361. doi: 10.7717/peerj.5361 (PMC6074769; doi:10.7717/peerj.5361)
Supplement: Supplemental Information 4 [file peerj-06-5361-s004.gz › FinalOutput_E-6/Phospholipase_A2_phaiodactylipin_6/finaltree.pdf]

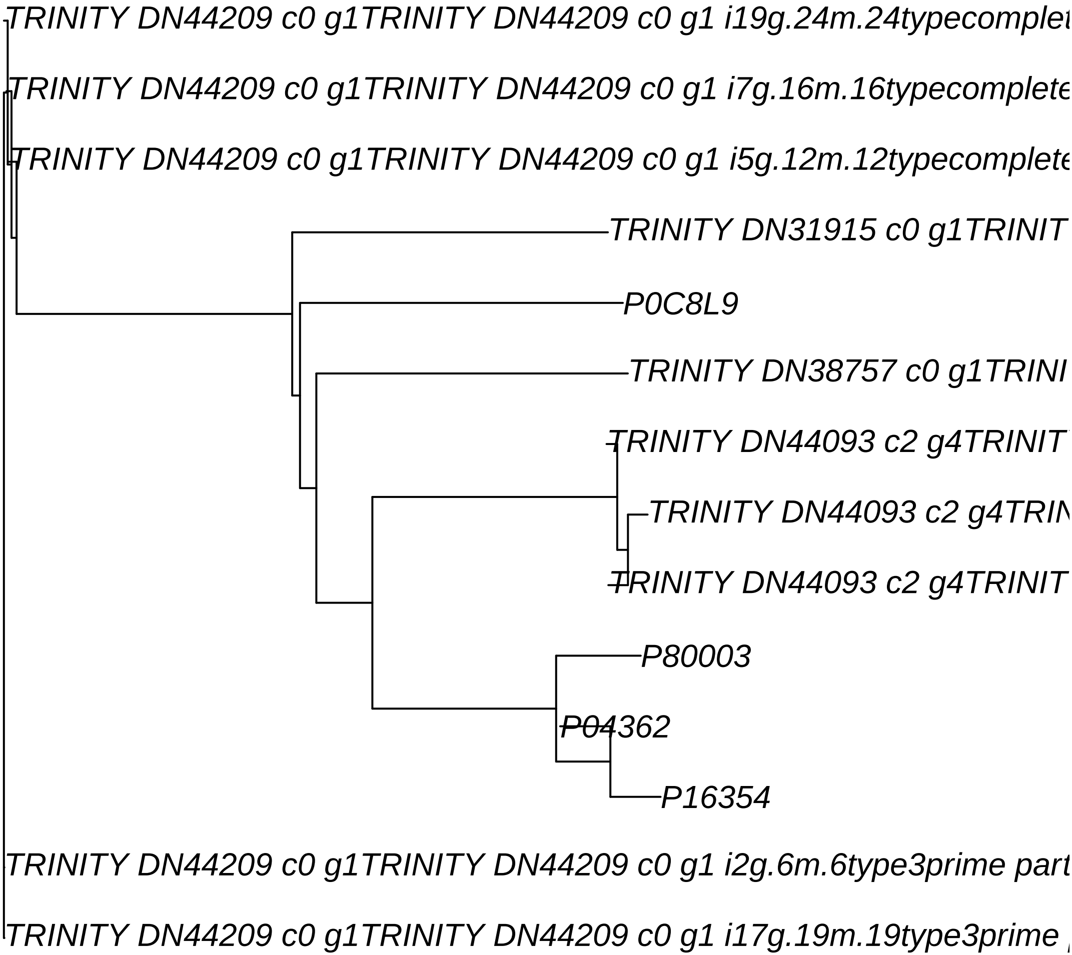
TRINITY DN44209 c0 g1TRINITY DN44209 c0 g1 i19g.24m.24typecomplet
TRINITY DN44209 c0 g1TRINITY DN44209 c0 g1 i7g.16m.16typecomplete
TRINITY DN44209 c0 g1TRINITY DN44209 c0 g1 i5g.12m.12typecomplete
TRINITY DN31915 c0 g1TRINIT
P0C8L9
TRINITY DN38757 c0 g1TRINI
TRINITY DN44093 c2 g4TRINIT
TRINITY DN44093 c2 g4TRIN
TRINITY DN44093 c2 g4TRINIT
P80003
P04362
P16354
TRINITY DN44209 c0 g1TRINITY DN44209 c0 g1 i2g.6m.6type3prime part
TRINITY DN44209 c0 g1TRINITY DN44209 c0 g1 i17g.19m.19type3prime
