# Supplementary material for: Venomix: a simple bioinformatic pipeline for identifying and characterizing toxin gene candidates from transcriptomic data
Source: PeerJ. 2018 Jul 31;6:e5361. doi: 10.7717/peerj.5361 (PMC6074769; doi:10.7717/peerj.5361)
Supplement: Supplemental Information 4 [file peerj-06-5361-s004.gz › FinalOutput_E-6/Kunitz-type_serine_protease_inhibitor_homolog_alpha-dendrotoxin_3/finaltree.pdf]

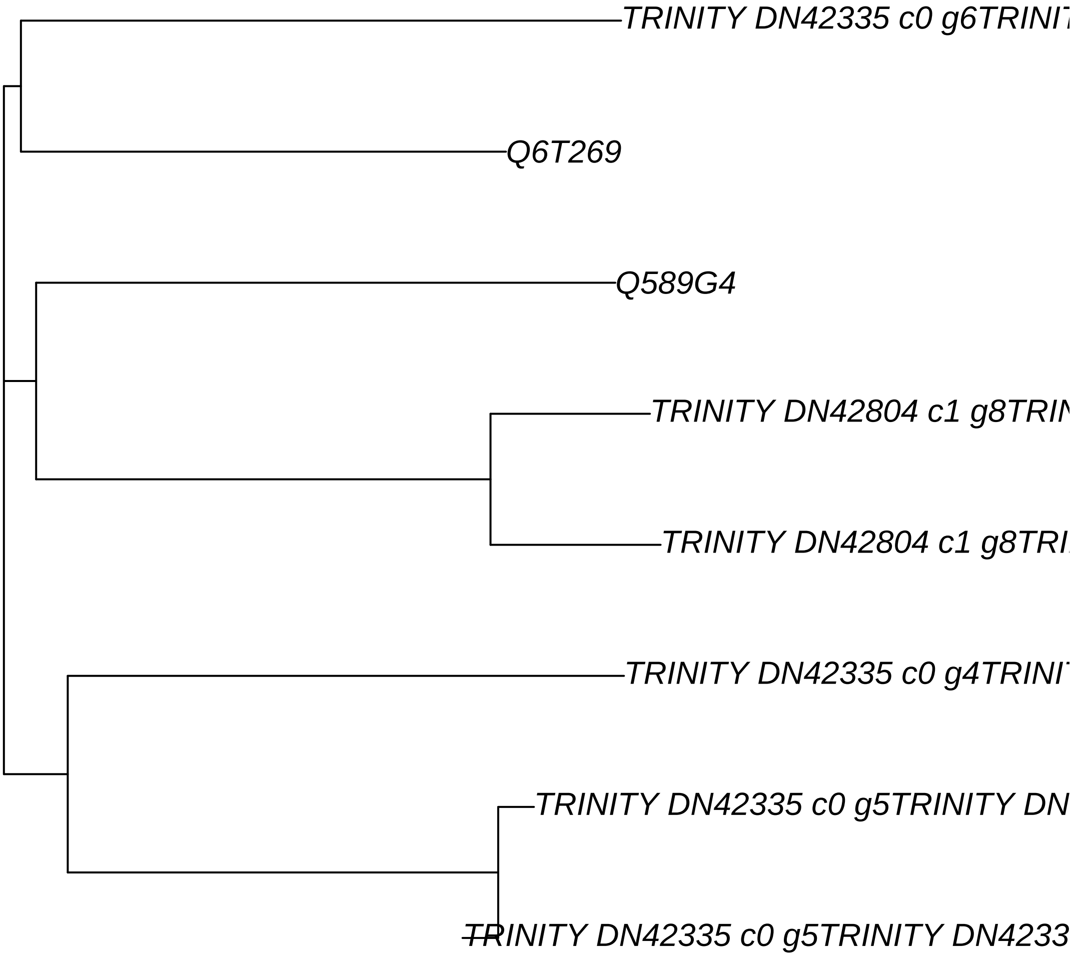

TRINITY DN42335 c0 g6TRINIT
Q6T269
Q589G4
TRINITY DN42804 c1 g8TRIN
TRINITY DN42804 c1 g8TRI
TRINITY DN42335 c0 g4TRINIT
TRINITY DN42335 c0 g5TRINITY DN
TRINITY DN42335 c0 g5TRINITY DN4233
