# Supplementary material for: Venomix: a simple bioinformatic pipeline for identifying and characterizing toxin gene candidates from transcriptomic data
Source: PeerJ. 2018 Jul 31;6:e5361. doi: 10.7717/peerj.5361 (PMC6074769; doi:10.7717/peerj.5361)
Supplement: Supplemental Information 4 [file peerj-06-5361-s004.gz › FinalOutput_E-6/Venom_allergen_4_1/finaltree.pdf]

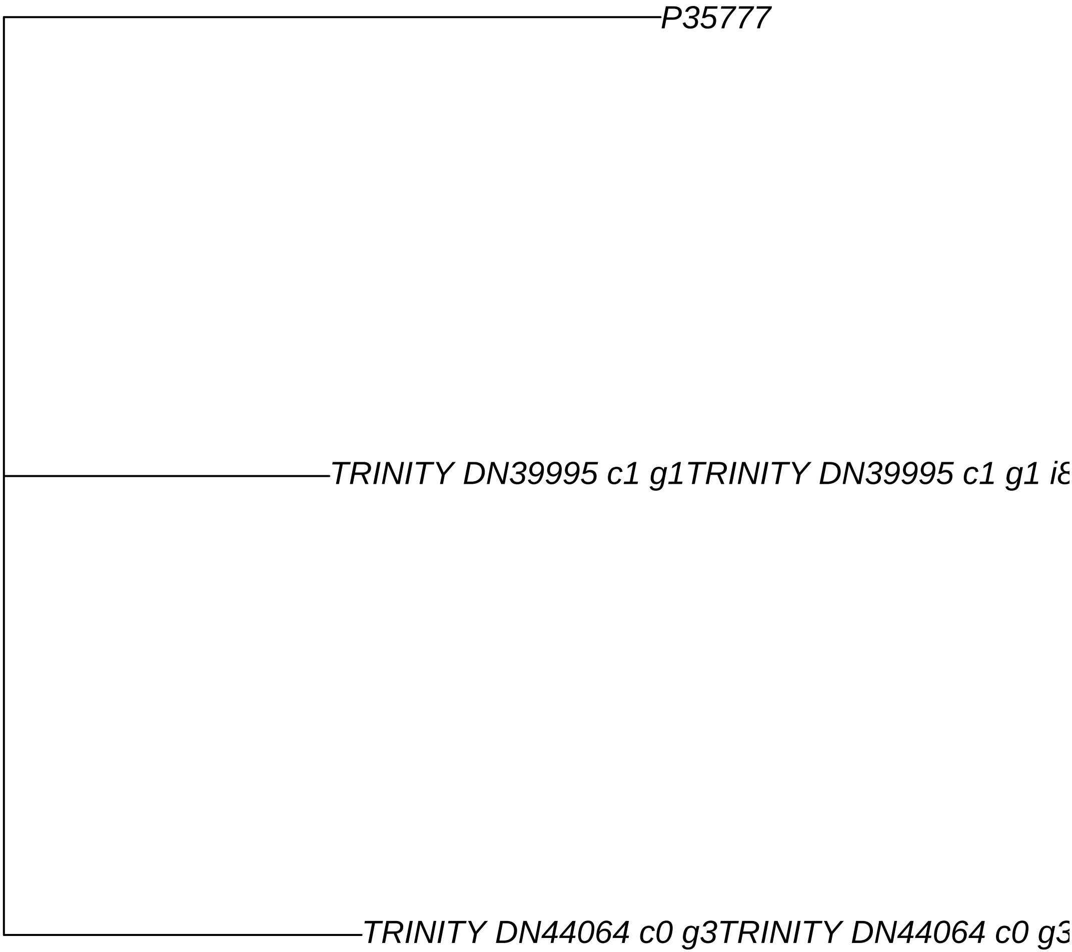
P35777
TRINITY DN39995 c1 g1TRINITY DN39995 c1 g1 i8
TRINITY DN44064 c0 g3TRINITY DN44064 c0 g3
