# Supplementary material for: Venomix: a simple bioinformatic pipeline for identifying and characterizing toxin gene candidates from transcriptomic data
Source: PeerJ. 2018 Jul 31;6:e5361. doi: 10.7717/peerj.5361 (PMC6074769; doi:10.7717/peerj.5361)
Supplement: Supplemental Information 4 [file peerj-06-5361-s004.gz › FinalOutput_E-6/Toxin_Bcs_III_15/finaltree.pdf]

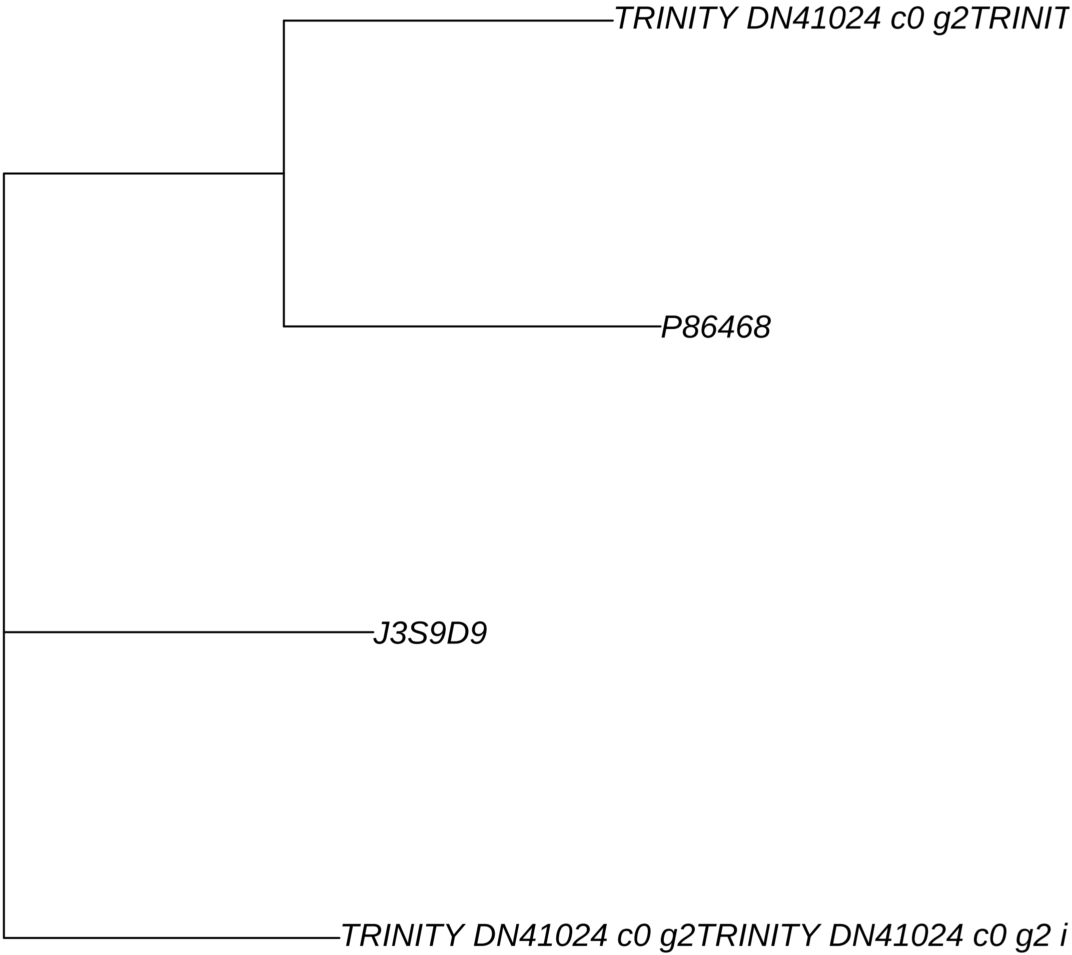
TRINITY DN41024 c0 g2TRINIT
P86468
J3S9D9
TRINITY DN41024 c0 g2TRINITY DN41024 c0 g2 i
