# Supplementary material for: Venomix: a simple bioinformatic pipeline for identifying and characterizing toxin gene candidates from transcriptomic data
Source: PeerJ. 2018 Jul 31;6:e5361. doi: 10.7717/peerj.5361 (PMC6074769; doi:10.7717/peerj.5361)
Supplement: Supplemental Information 4 [file peerj-06-5361-s004.gz › FinalOutput_E-6/Putative_protein-glutamate_O-methyltransferase_1/finaltree.pdf]

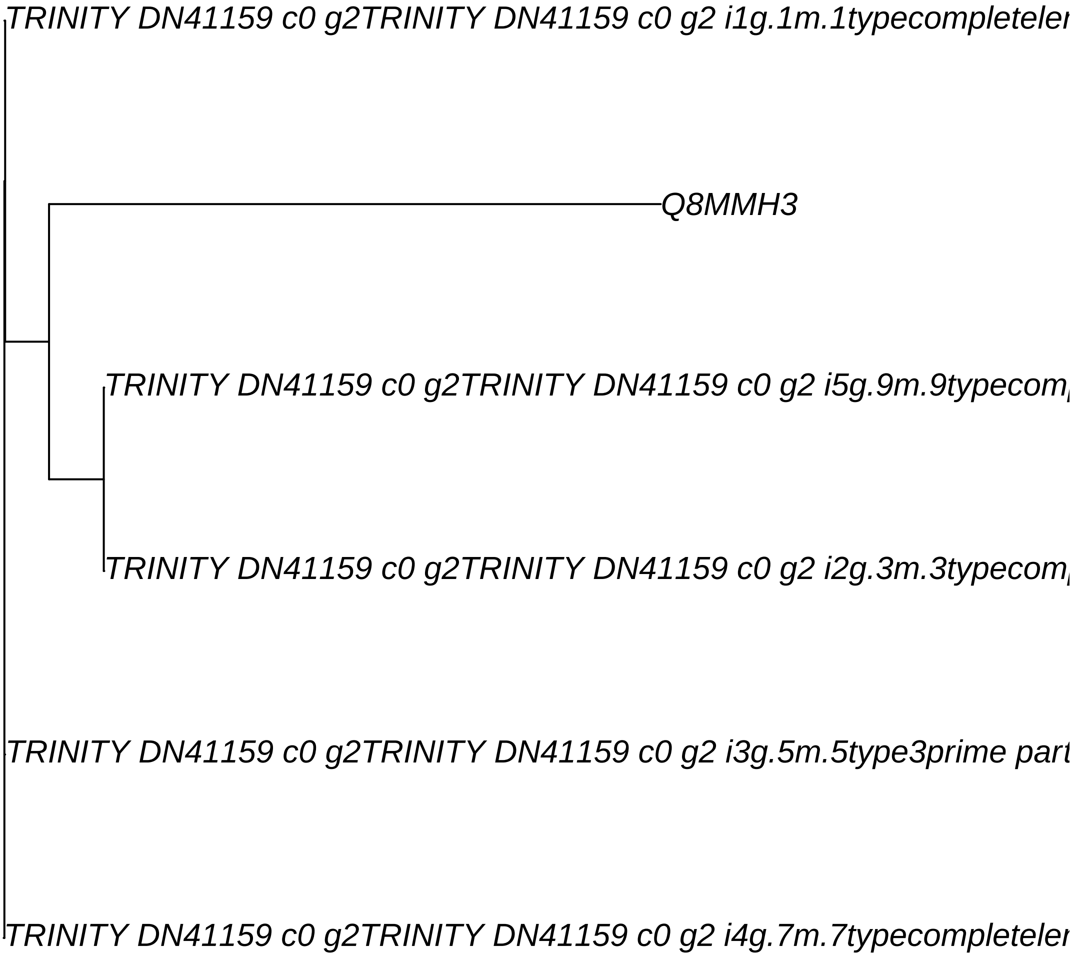
TRINITY DN41159 c0 g2TRINITY DN41159 c0 g2 i1g.1m.1typecompletele
Q8MMH3
TRINITY DN41159 c0 g2TRINITY DN41159 c0 g2 i5g.9m.9typecom
TRINITY DN41159 c0 g2TRINITY DN41159 c0 g2 i2g.3m.3typecom
TRINITY DN41159 c0 g2TRINITY DN41159 c0 g2 i3g.5m.5type3prime par
TRINITY DN41159 c0 g2TRINITY DN41159 c0 g2 i4g.7m.7typecompletele
