# Supplementary material for: Venomix: a simple bioinformatic pipeline for identifying and characterizing toxin gene candidates from transcriptomic data
Source: PeerJ. 2018 Jul 31;6:e5361. doi: 10.7717/peerj.5361 (PMC6074769; doi:10.7717/peerj.5361)
Supplement: Supplemental Information 4 [file peerj-06-5361-s004.gz › FinalOutput_E-6/Snake_venom_metalloproteinase_acutolysin-C_1/finaltree.pdf]

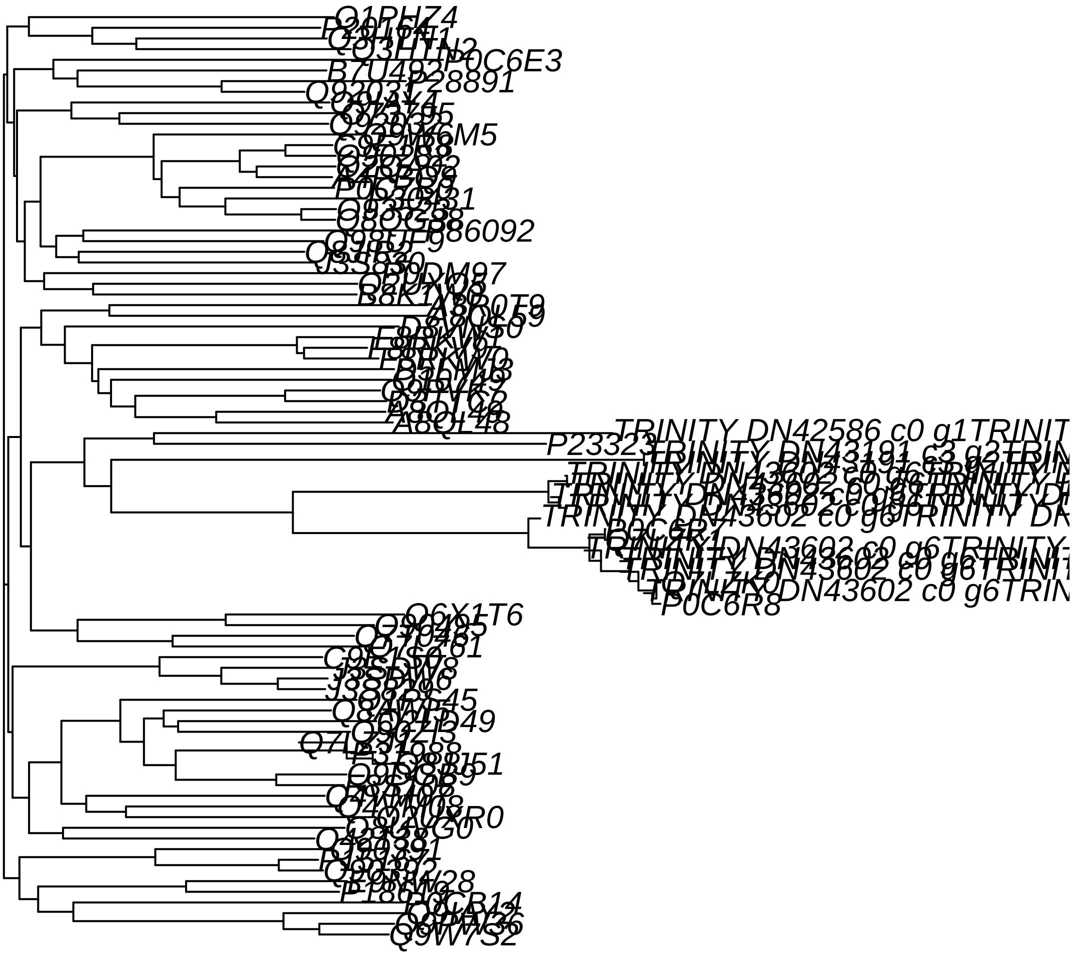
Q1PHZ4
P0C6E3
P28891
P23323
Q6X1T6
P0C6R8
Q9W7S2
