# Supplementary material for: Venomix: a simple bioinformatic pipeline for identifying and characterizing toxin gene candidates from transcriptomic data
Source: PeerJ. 2018 Jul 31;6:e5361. doi: 10.7717/peerj.5361 (PMC6074769; doi:10.7717/peerj.5361)
Supplement: Supplemental Information 4 [file peerj-06-5361-s004.gz › FinalOutput_E-6/Thrombin-like_enzyme_ancrod_11/finaltree.pdf]

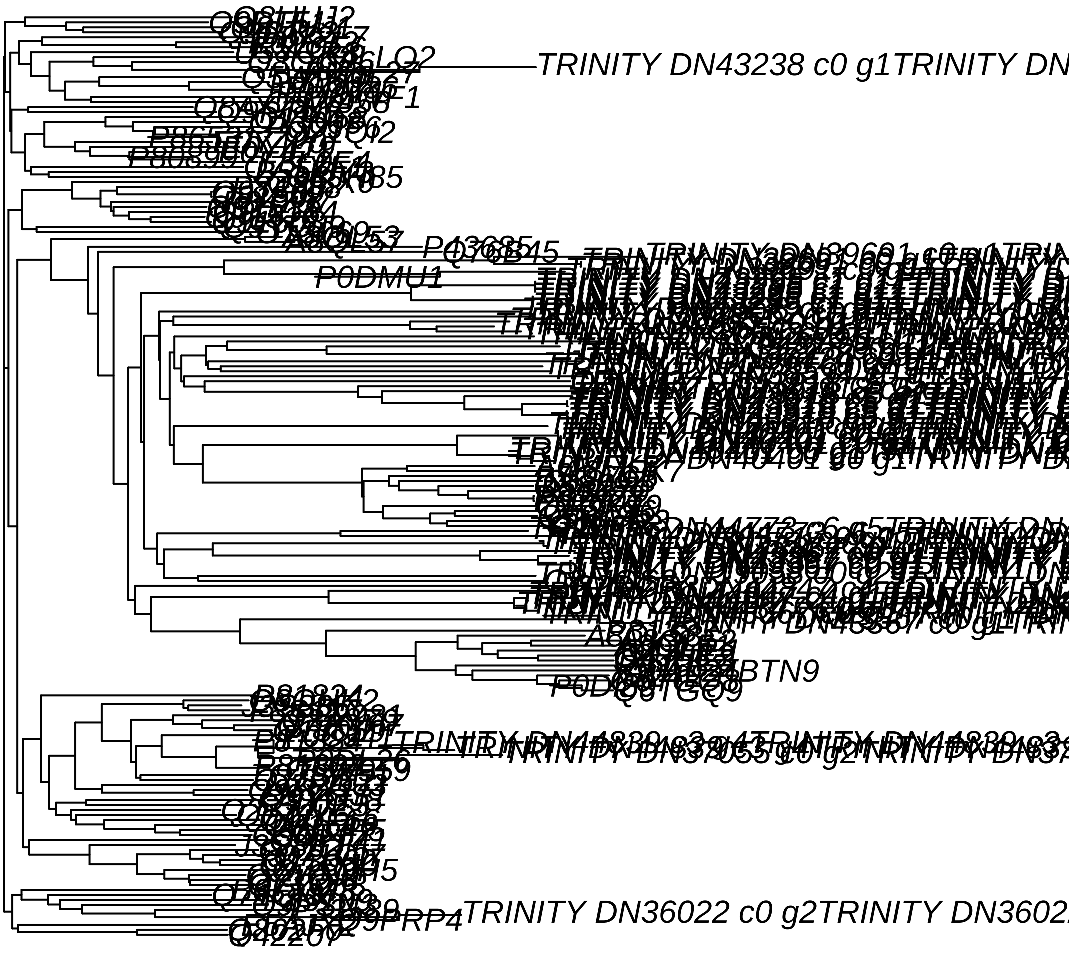

TRINITY DN43238 c0 g1TRINITY DN
P0DMU1
TRINITY DN36022 c0 g2TRINITY DN3602
