# Supplementary material for: Venomix: a simple bioinformatic pipeline for identifying and characterizing toxin gene candidates from transcriptomic data
Source: PeerJ. 2018 Jul 31;6:e5361. doi: 10.7717/peerj.5361 (PMC6074769; doi:10.7717/peerj.5361)
Supplement: Supplemental Information 4 [file peerj-06-5361-s004.gz › FinalOutput_E-6/Zinc_metalloproteinase_recombinant_fibrinogenase_II_1/finaltree.pdf]

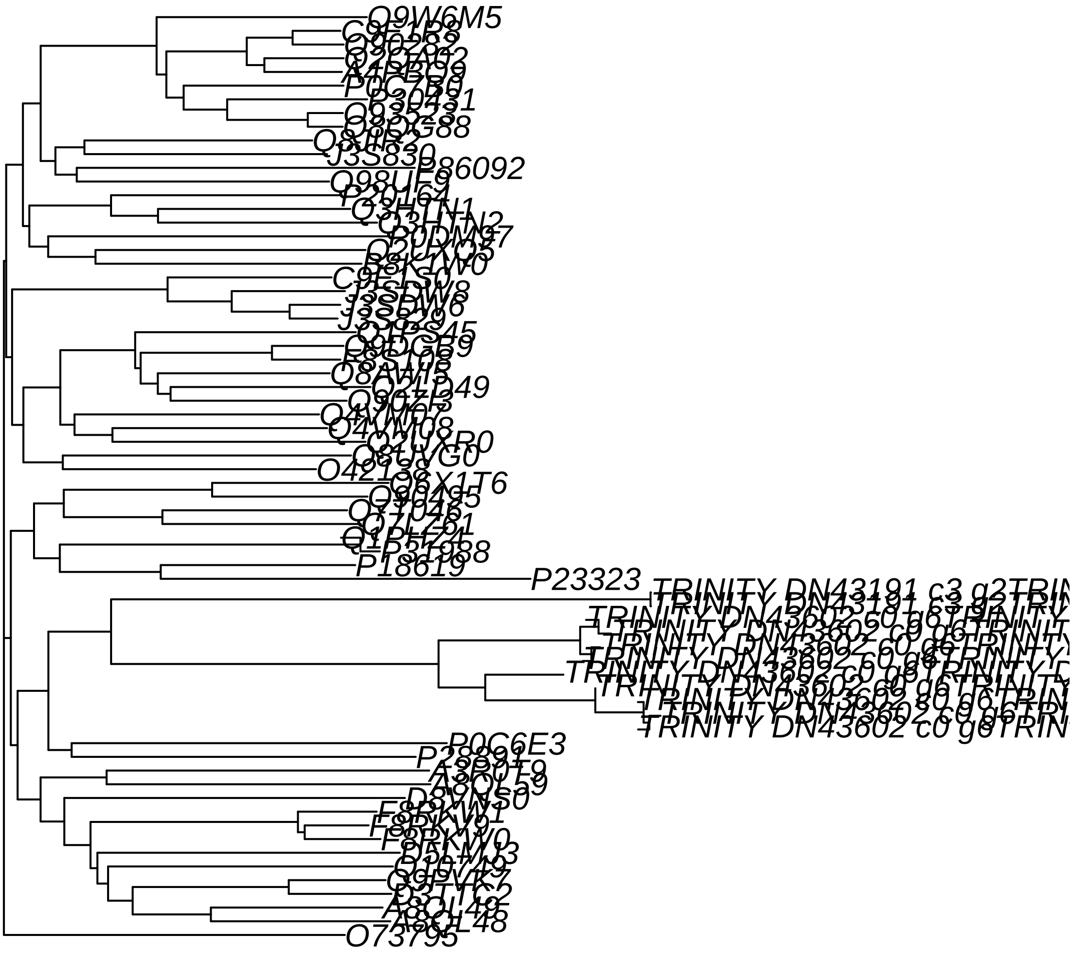
Q9W6M5
P30431
P86092
P0DM97
P23323
P18619
P0C6E3
O73795
