# Supplementary material for: Venomix: a simple bioinformatic pipeline for identifying and characterizing toxin gene candidates from transcriptomic data
Source: PeerJ. 2018 Jul 31;6:e5361. doi: 10.7717/peerj.5361 (PMC6074769; doi:10.7717/peerj.5361)
Supplement: Supplemental Information 4 [file peerj-06-5361-s004.gz › FinalOutput_E-6/Serine_proteinase-like_BMK-CBP_1/finaltree.pdf]

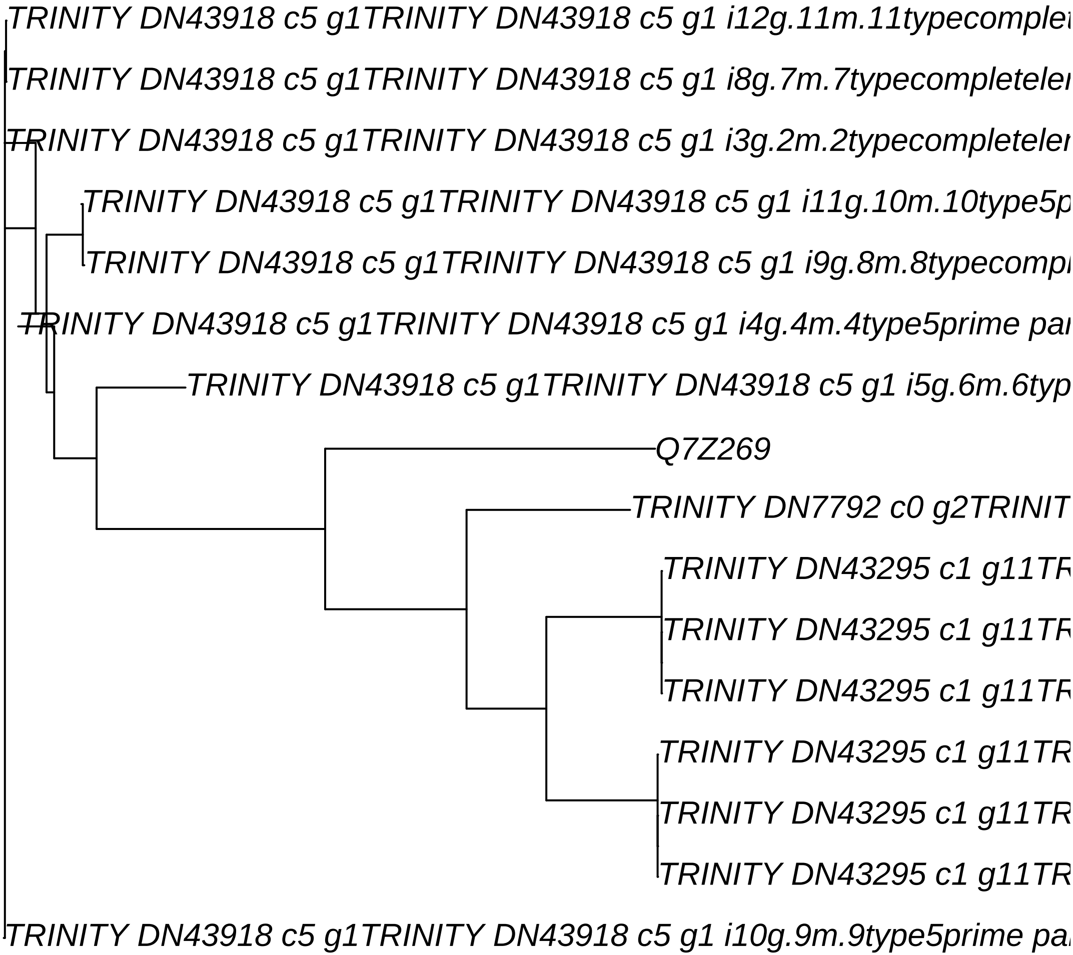
TRINITY DN43918 c5 g1TRINITY DN43918 c5 g1 i12g.11m.11typecomplet
TRINITY DN43918 c5 g1TRINITY DN43918 c5 g1 i8g.7m.7typecompleteler
TRINITY DN43918 c5 g1TRINITY DN43918 c5 g1 i3g.2m.2typecompleteler
TRINITY DN43918 c5 g1TRINITY DN43918 c5 g1 i11g.10m.10type5p
TRINITY DN43918 c5 g1TRINITY DN43918 c5 g1 i9g.8m.8typecompl
TRINITY DN43918 c5 g1TRINITY DN43918 c5 g1 i4g.4m.4type5prime pa
TRINITY DN43918 c5 g1TRINITY DN43918 c5 g1 i5g.6m.6typ
Q7Z269
TRINITY DN7792 c0 g2TRINIT
TRINITY DN43295 c1 g11TR
TRINITY DN43295 c1 g11TR
TRINITY DN43295 c1 g11TR
TRINITY DN43295 c1 g11TR
TRINITY DN43295 c1 g11TR
TRINITY DN43295 c1 g11TR
TRINITY DN43918 c5 g1TRINITY DN43918 c5 g1 i10g.9m.9type5prime pa
