# Supplementary material for: Venomix: a simple bioinformatic pipeline for identifying and characterizing toxin gene candidates from transcriptomic data
Source: PeerJ. 2018 Jul 31;6:e5361. doi: 10.7717/peerj.5361 (PMC6074769; doi:10.7717/peerj.5361)
Supplement: Supplemental Information 4 [file peerj-06-5361-s004.gz › FinalOutput_E-6/Zinc_metalloproteinase@disintegrin_4/finaltree.pdf]

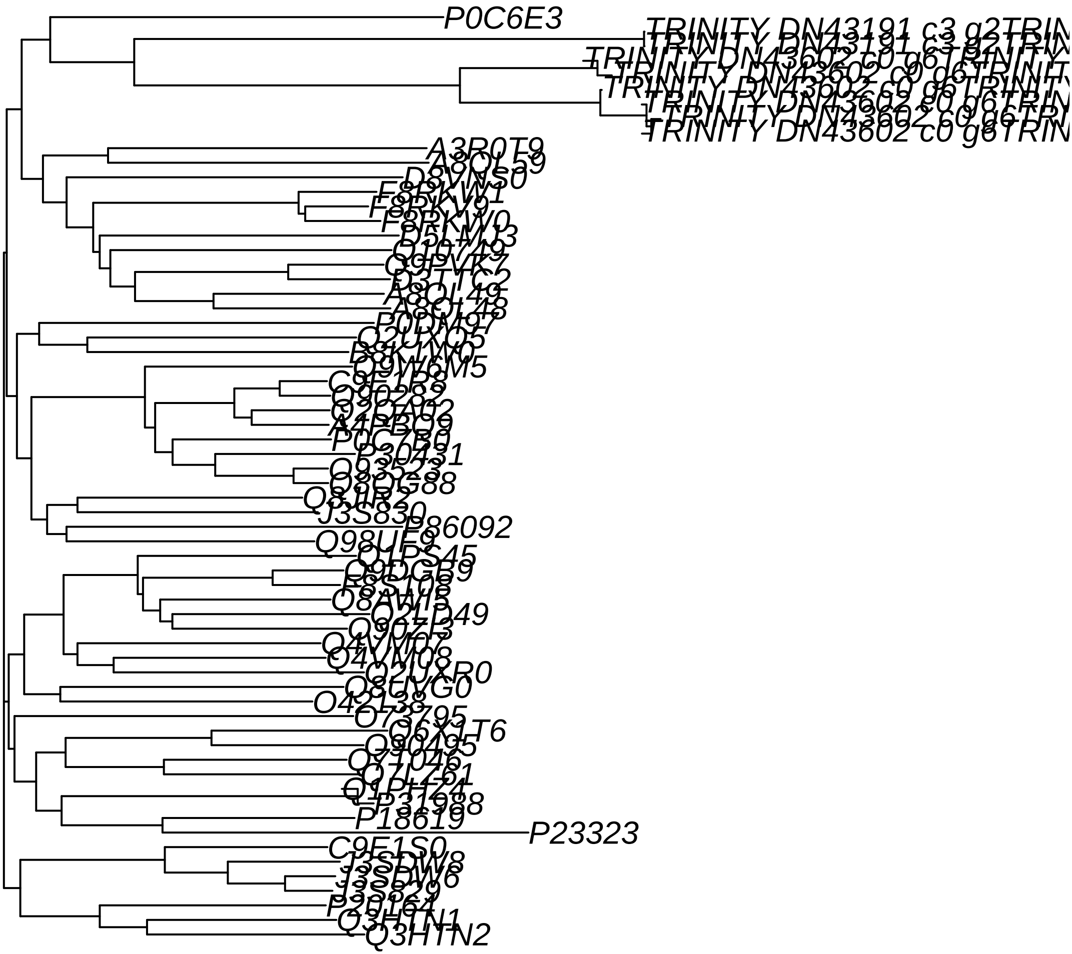
P0C6E3
TRINITY DN43191 c3 g2TRIN
A3R0T9
A9QL59
D6VNS0
F8RKW1
F8RKV9
F8RKW0
D5LM13
Q19TA9
Q9PVK7
D3TTC2
A8QL49
A8QL48
P0DM97
Q2UX05
B8K1W0
Q9W6M5
C9E1R8
Q90282
Q2PAJ2
A1BBC9
P0C7B0
P30431
Q6J523
Q8QG88
Q8JR82
J3S830
P86092
Q98UF9
Q1PS45
Q9DGB9
F8S108
Q8AWI5
Q2LD49
Q90ZI3
Q4VM07
Q4VM08
Q2UXR0
Q8UVG0
O73795
Q6X1T6
Q90495
Q7T046
Q7LZ61
Q1PHZ4
P31988
P18619
P23323
C9E1S0
J3SDW8
J3SDW6
J3S829
Q3HTN1
Q3HTN2
