# Supplementary material for: Venomix: a simple bioinformatic pipeline for identifying and characterizing toxin gene candidates from transcriptomic data
Source: PeerJ. 2018 Jul 31;6:e5361. doi: 10.7717/peerj.5361 (PMC6074769; doi:10.7717/peerj.5361)
Supplement: Supplemental Information 4 [file peerj-06-5361-s004.gz › FinalOutput_E-6/Hyaluronidase_1/finaltree.pdf]

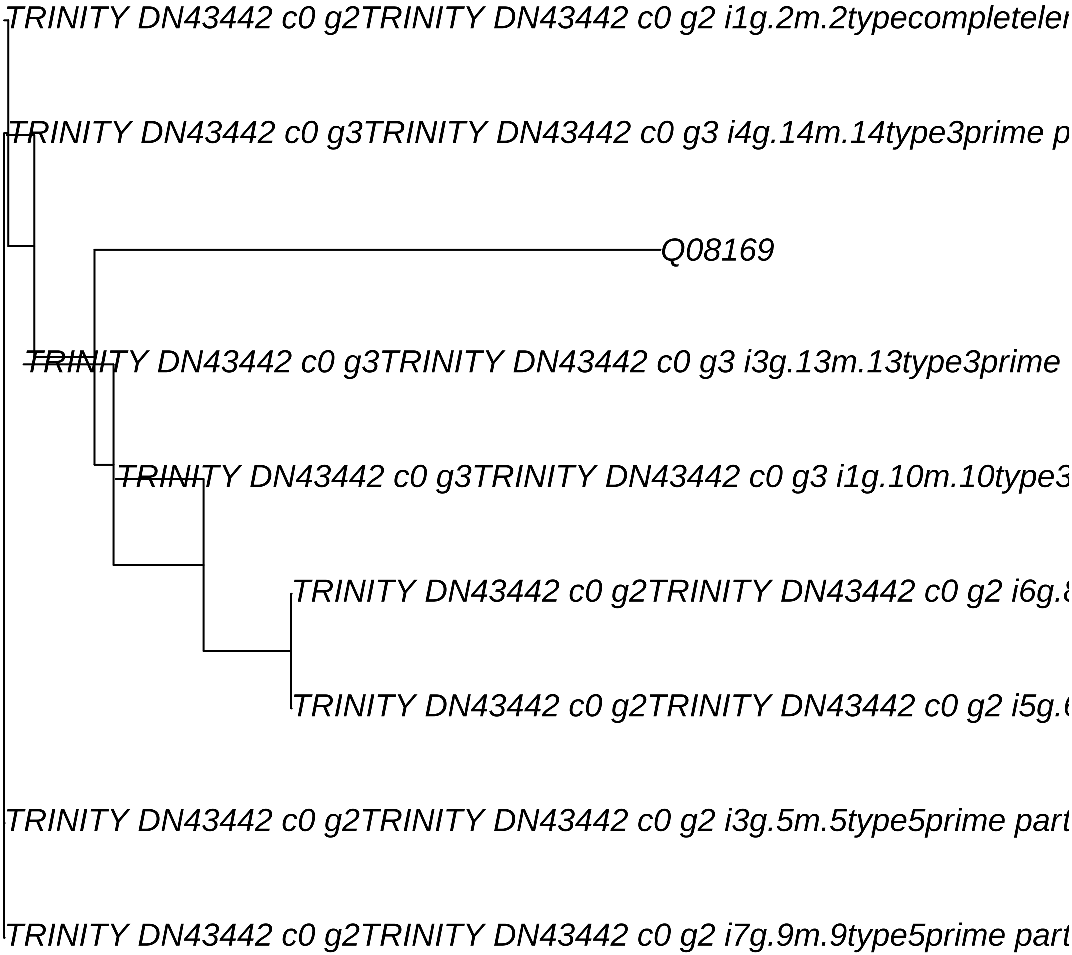
TRINITY DN43442 c0 g2TRINITY DN43442 c0 g2 i1g.2m.2typecompleteler
TRINITY DN43442 c0 g3TRINITY DN43442 c0 g3 i4g.14m.14type3prime p
Q08169
TRINITY DN43442 c0 g3TRINITY DN43442 c0 g3 i3g.13m.13type3prime
TRINITY DN43442 c0 g3TRINITY DN43442 c0 g3 i1g.10m.10type3
TRINITY DN43442 c0 g2TRINITY DN43442 c0 g2 i6g.8
TRINITY DN43442 c0 g2TRINITY DN43442 c0 g2 i5g.6
TRINITY DN43442 c0 g2TRINITY DN43442 c0 g2 i3g.5m.5type5prime part
TRINITY DN43442 c0 g2TRINITY DN43442 c0 g2 i7g.9m.9type5prime part
