# Supplementary material for: Venomix: a simple bioinformatic pipeline for identifying and characterizing toxin gene candidates from transcriptomic data
Source: PeerJ. 2018 Jul 31;6:e5361. doi: 10.7717/peerj.5361 (PMC6074769; doi:10.7717/peerj.5361)
Supplement: Supplemental Information 4 [file peerj-06-5361-s004.gz › FinalOutput_E-6/Venom_allergen_3_30/finaltree.pdf]

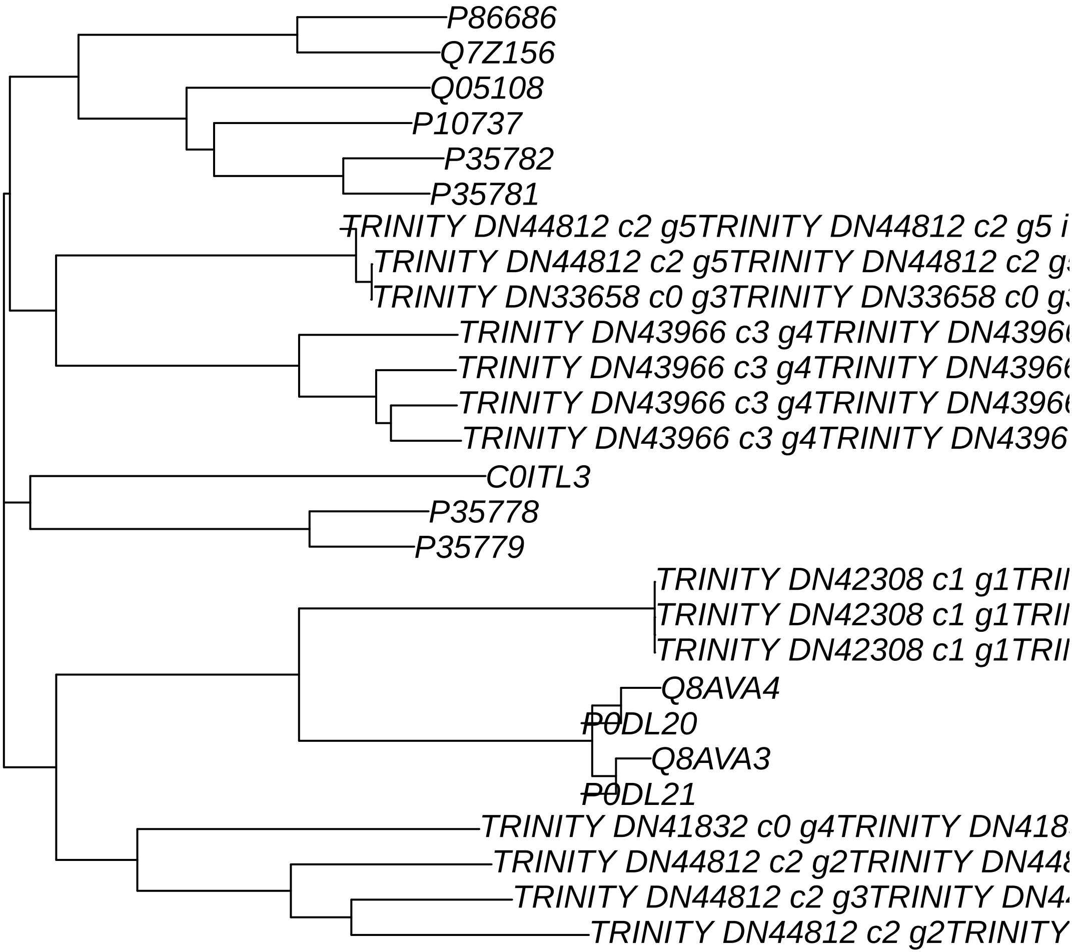
P86686
Q7Z156
Q05108
P10737
P35782
P35781
TRINITY DN44812 c2 g5TRINITY DN44812 c2 g5 i
TRINITY DN44812 c2 g5TRINITY DN44812 c2 g
TRINITY DN33658 c0 g3TRINITY DN33658 c0 g
TRINITY DN43966 c3 g4TRINITY DN4396
TRINITY DN43966 c3 g4TRINITY DN4396
TRINITY DN43966 c3 g4TRINITY DN4396
TRINITY DN43966 c3 g4TRINITY DN4396
C0ITL3
P35778
P35779
TRINITY DN42308 c1 g1TRI
TRINITY DN42308 c1 g1TRI
TRINITY DN42308 c1 g1TRI
Q8AVA4
P0DL20
Q8AVA3
P0DL21
TRINITY DN41832 c0 g4TRINITY DN418
TRINITY DN44812 c2 g2TRINITY DN448
TRINITY DN44812 c2 g3TRINITY DN44
TRINITY DN44812 c2 g2TRINITY
