# Supplementary material for: Venomix: a simple bioinformatic pipeline for identifying and characterizing toxin gene candidates from transcriptomic data
Source: PeerJ. 2018 Jul 31;6:e5361. doi: 10.7717/peerj.5361 (PMC6074769; doi:10.7717/peerj.5361)
Supplement: Supplemental Information 4 [file peerj-06-5361-s004.gz › FinalOutput_E-6/Zinc_metalloproteinase@disintegrin_5/finaltree.pdf]

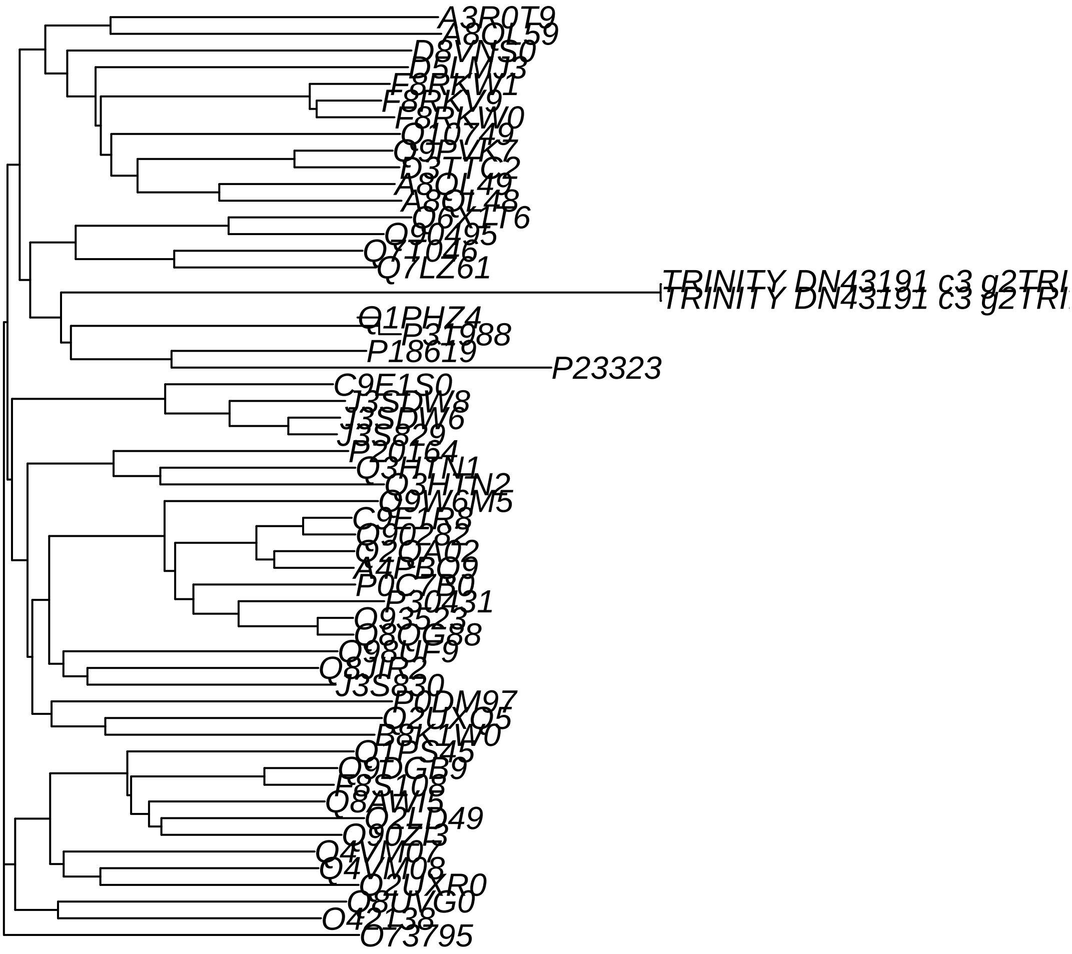
A3R0T9
A8QL59
D8VNS0
D5LMJ3
F8RKW1
F8RKV9
F8RKW0
Q10749
Q9PVK7
D3TTC2
A8QL49
A8QL48
Q6XLT6
Q90495
Q7T046
Q7LZ61
TRINITY DN43191 c3 g2TRI
TRINITY DN43191 c3 g2TRI
Q1PHZ4
P31988
P18619
P23323
C9E1S0
J3SDW8
J3SDW6
J3S829
P20164
Q3HTN1
Q3HTN2
Q9W6M5
C9E1R8
Q90282
Q2QA02
A4PBQ9
P0C7B0
P30431
O93523
Q8QG88
Q98UF9
Q8JIR2
J3S830
P0DM97
Q2UXQ5
B8K1W0
Q1PS45
Q9DGB9
F8S108
O8AWI5
Q2LD49
Q90ZI3
Q4VM07
Q4VM08
Q2UXR0
Q8UVG0
O42138
O73795
