# Supplementary material for: Venomix: a simple bioinformatic pipeline for identifying and characterizing toxin gene candidates from transcriptomic data
Source: PeerJ. 2018 Jul 31;6:e5361. doi: 10.7717/peerj.5361 (PMC6074769; doi:10.7717/peerj.5361)
Supplement: Supplemental Information 4 [file peerj-06-5361-s004.gz › FinalOutput_E-6/Peroxiredoxin-4_1/finaltree.pdf]

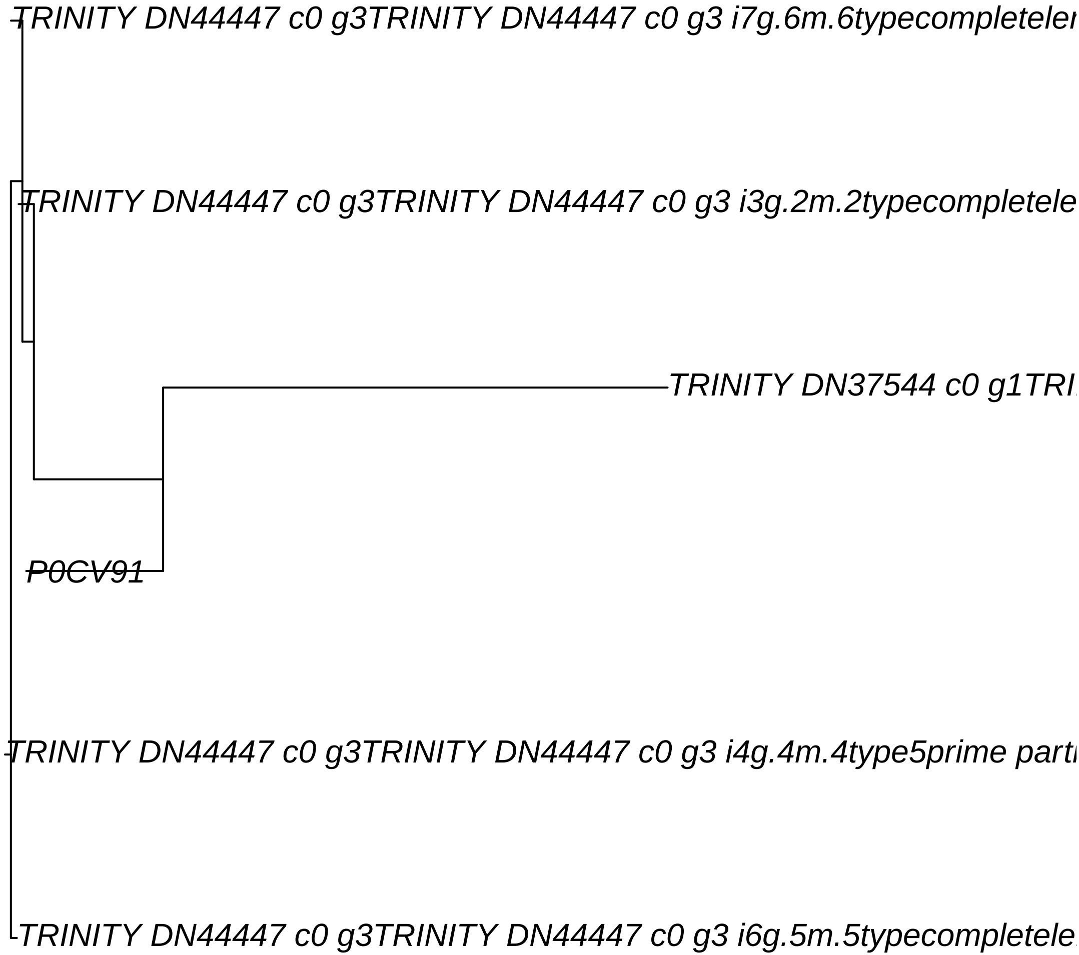
TRINITY DN44447 c0 g3TRINITY DN44447 c0 g3 i7g.6m.6typecompletelen
TRINITY DN44447 c0 g3TRINITY DN44447 c0 g3 i3g.2m.2typecompletele
TRINITY DN37544 c0 g1TRI
P0CV91
TRINITY DN44447 c0 g3TRINITY DN44447 c0 g3 i4g.4m.4type5prime parti
TRINITY DN44447 c0 g3TRINITY DN44447 c0 g3 i6g.5m.5typecompletele
