# Supplementary material for: Venomix: a simple bioinformatic pipeline for identifying and characterizing toxin gene candidates from transcriptomic data
Source: PeerJ. 2018 Jul 31;6:e5361. doi: 10.7717/peerj.5361 (PMC6074769; doi:10.7717/peerj.5361)
Supplement: Supplemental Information 4 [file peerj-06-5361-s004.gz › FinalOutput_E-6/Disintegrin_bitistatin_6/finaltree.pdf]

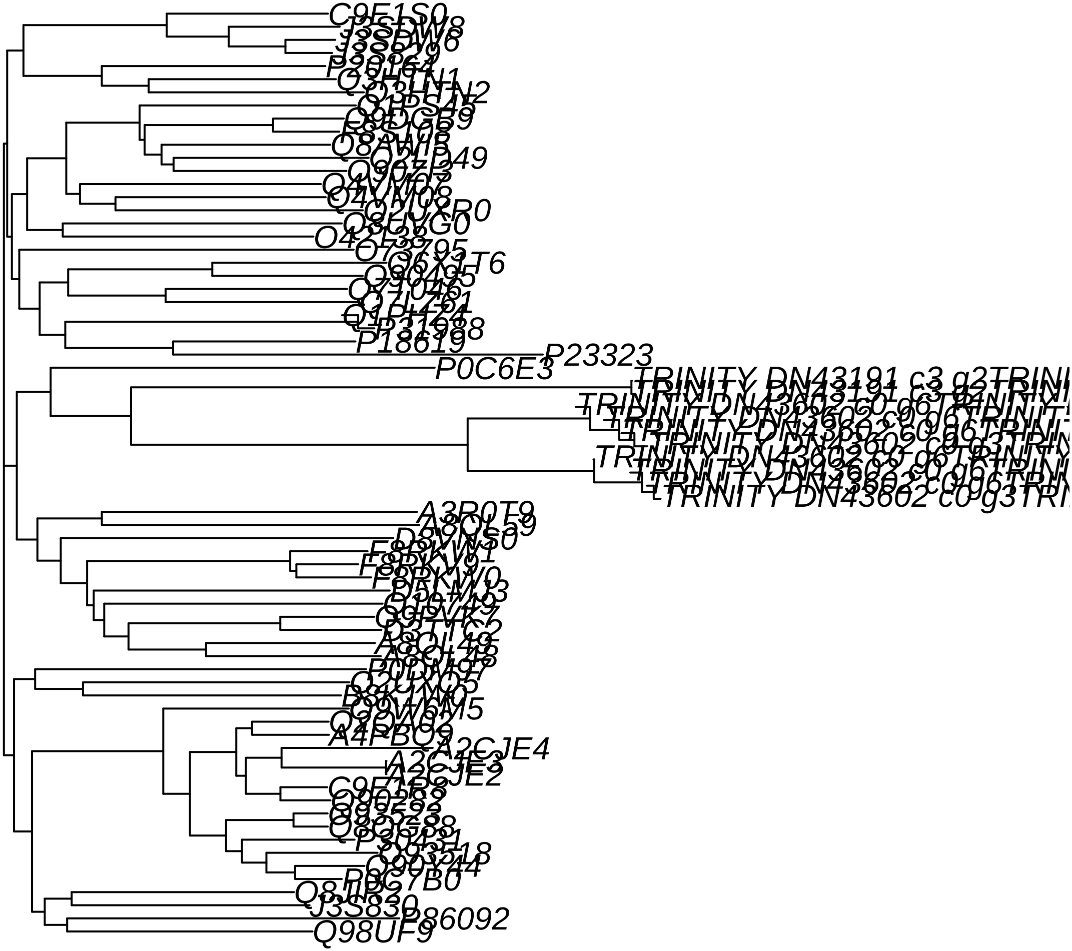
C9E1S0
P23323
P0C6E3
TRINITY DN43602 c0 g3TRI
A3R0T9
D8VNS0
A2CJE4
A4PBQ9
A2CJE2
P86092
Q98UF9
P18619
Q4VM08
