# Supplementary material for: Venomix: a simple bioinformatic pipeline for identifying and characterizing toxin gene candidates from transcriptomic data
Source: PeerJ. 2018 Jul 31;6:e5361. doi: 10.7717/peerj.5361 (PMC6074769; doi:10.7717/peerj.5361)
Supplement: Supplemental Information 4 [file peerj-06-5361-s004.gz › FinalOutput_E-6/Zinc_metalloproteinase-disintegrin-like_ammodytagin_1/finaltree.pdf]

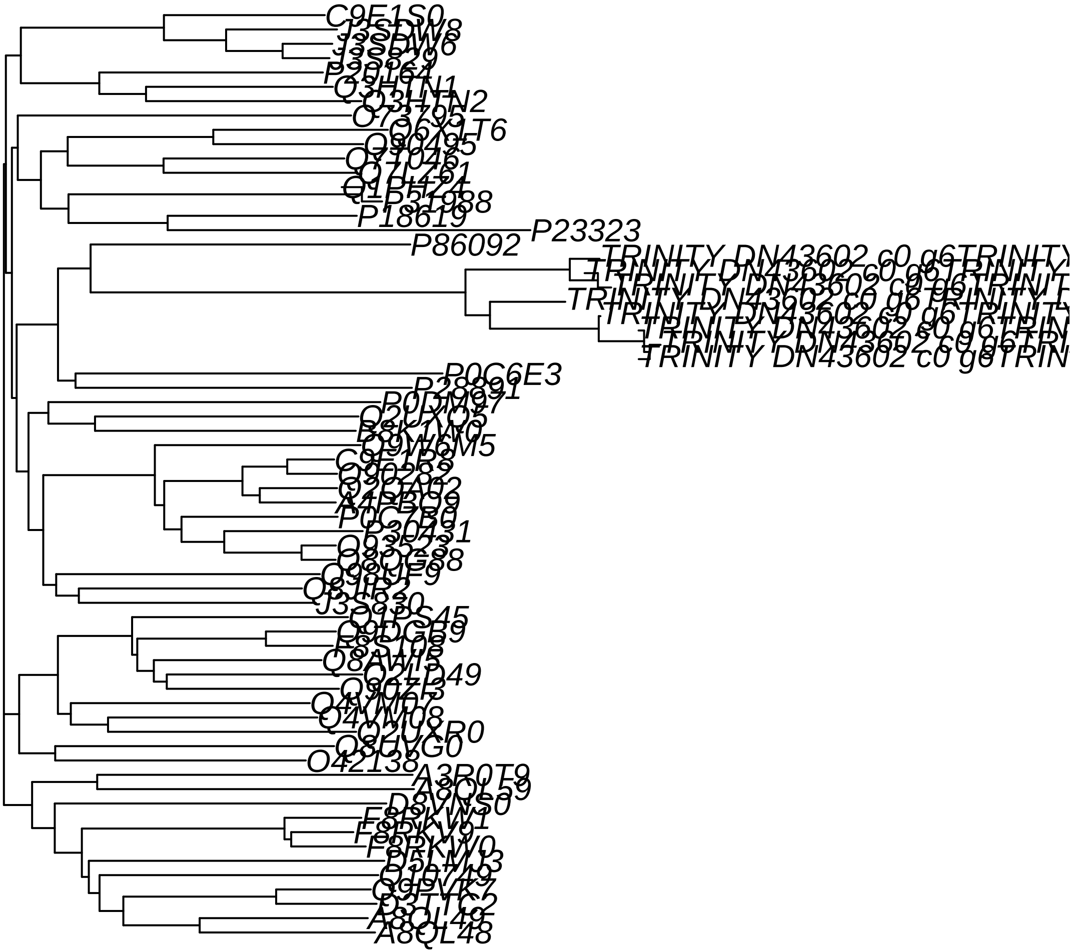

C9E1S0
J3SDW8
J3SDW6
J3S829
P20164
Q3HTN1
Q3HTN2
O73795
Q6X1T6
Q90495
Q7T046
Q1PHZ4
P18619
P23323
P86092
TRINITY DN43602 c0 g6TRIN
P0C6E3
P28891
B8K1W0
Q9W6M5
P0C7R0
P20431
Q8QG88
J3S830
Q1PS45
Q9DGB9
Q8AWI5
Q2LD49
Q4VM08
Q2UXR0
Q8UVG0
O42138
A3R0T9
A8QL59
D8VNS0
F8RKW1
F8RKV9
F8RKW0
Q9PVK7
A8QL49
A8QL48
