# Supplementary material for: Venomix: a simple bioinformatic pipeline for identifying and characterizing toxin gene candidates from transcriptomic data
Source: PeerJ. 2018 Jul 31;6:e5361. doi: 10.7717/peerj.5361 (PMC6074769; doi:10.7717/peerj.5361)
Supplement: Supplemental Information 4 [file peerj-06-5361-s004.gz › FinalOutput_E-6/Hyaluronidase_conohyal-Cn1_1/finaltree.pdf]

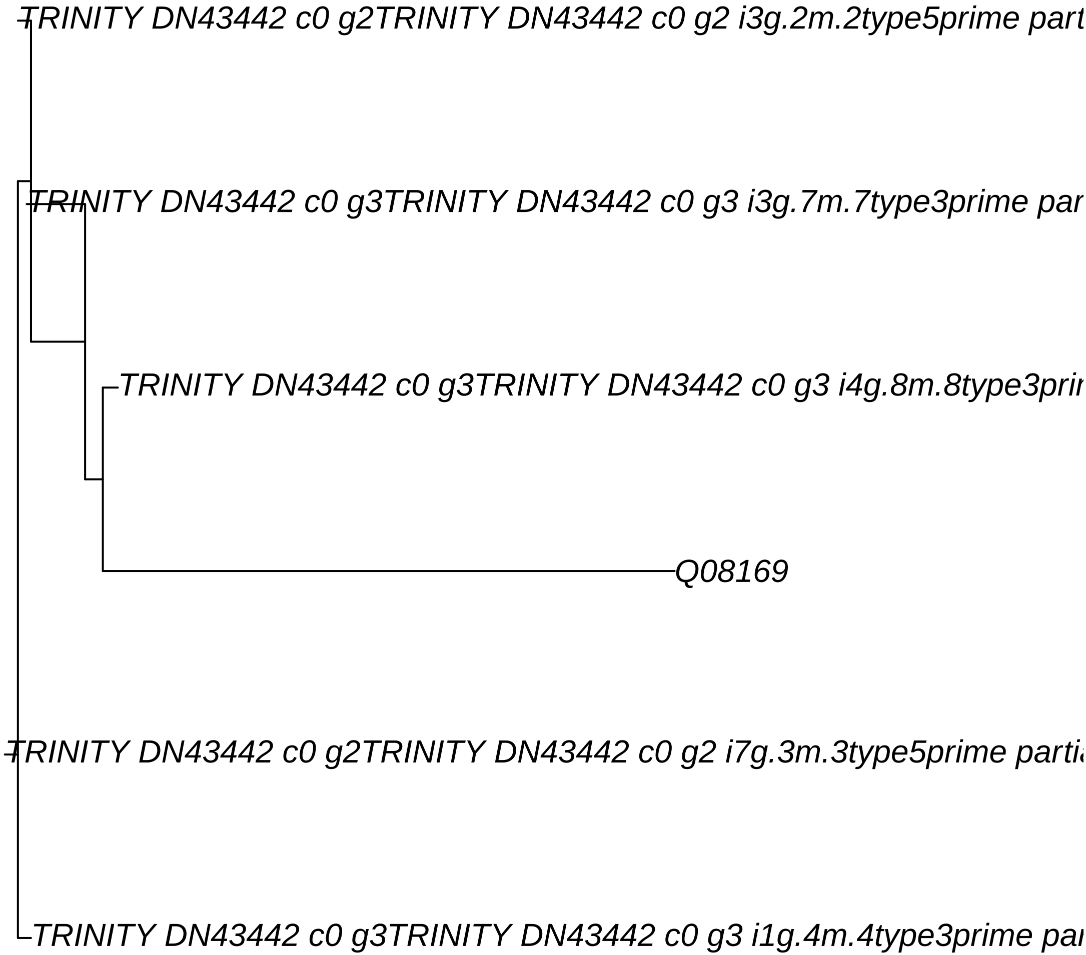
TRINITY DN43442 c0 g2TRINITY DN43442 c0 g2 i3g.2m.2type5prime part
TRINITY DN43442 c0 g3TRINITY DN43442 c0 g3 i3g.7m.7type3prime par
TRINITY DN43442 c0 g3TRINITY DN43442 c0 g3 i4g.8m.8type3prir
Q08169
TRINITY DN43442 c0 g2TRINITY DN43442 c0 g2 i7g.3m.3type5prime partia
TRINITY DN43442 c0 g3TRINITY DN43442 c0 g3 i1g.4m.4type3prime par
