# Supplementary material for: Venomix: a simple bioinformatic pipeline for identifying and characterizing toxin gene candidates from transcriptomic data
Source: PeerJ. 2018 Jul 31;6:e5361. doi: 10.7717/peerj.5361 (PMC6074769; doi:10.7717/peerj.5361)
Supplement: Supplemental Information 4 [file peerj-06-5361-s004.gz › FinalOutput_E-6/Phospholipase_A2_1/finaltree.pdf]

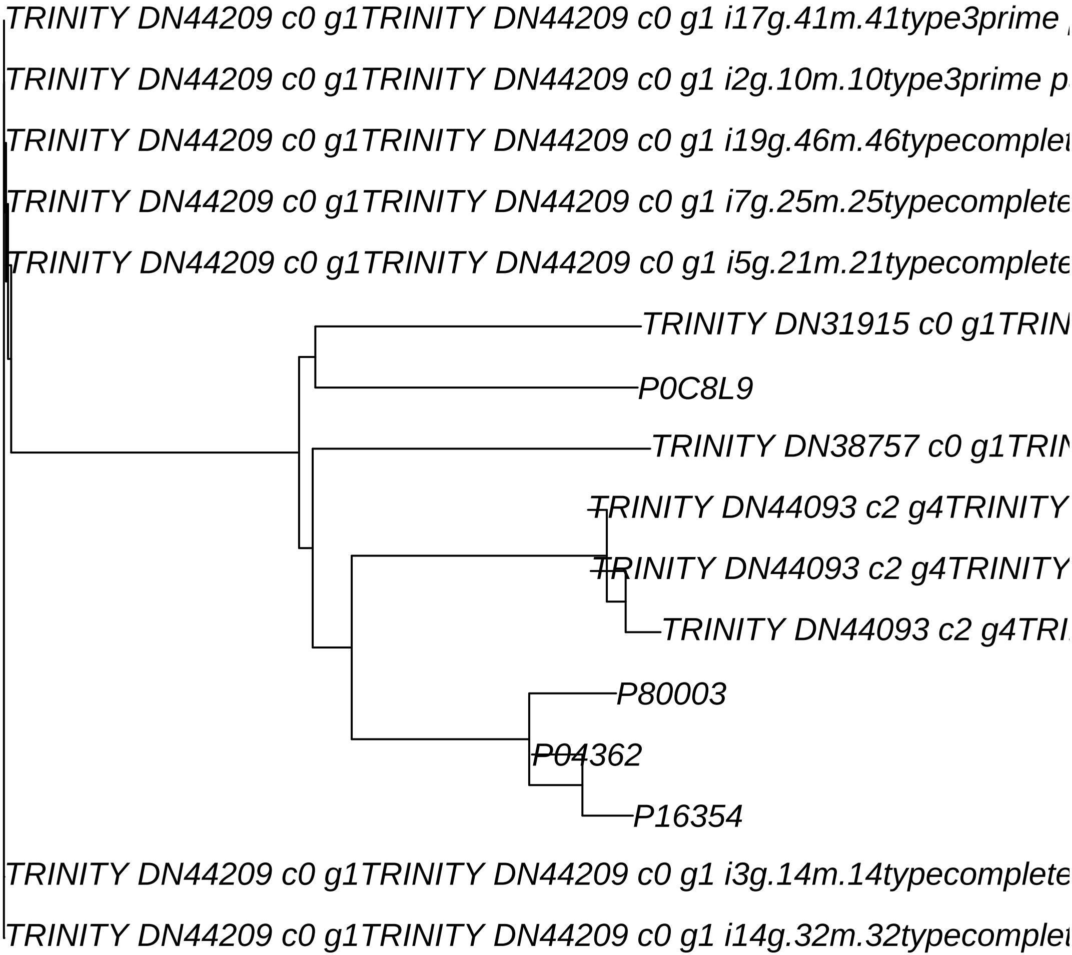

TRINITY DN44209 c0 g1TRINITY DN44209 c0 g1 i17g.41m.41type3prime
TRINITY DN44209 c0 g1TRINITY DN44209 c0 g1 i2g.10m.10type3prime p
TRINITY DN44209 c0 g1TRINITY DN44209 c0 g1 i19g.46m.46typecomplet
TRINITY DN44209 c0 g1TRINITY DN44209 c0 g1 i7g.25m.25typecomplete
TRINITY DN44209 c0 g1TRINITY DN44209 c0 g1 i5g.21m.21typecomplete
TRINITY DN31915 c0 g1TRIN
P0C8L9
TRINITY DN38757 c0 g1TRIN
TRINITY DN44093 c2 g4TRINITY
TRINITY DN44093 c2 g4TRINITY
TRINITY DN44093 c2 g4TRI
P80003
P04362
P16354
TRINITY DN44209 c0 g1TRINITY DN44209 c0 g1 i3g.14m.14typecomplete
TRINITY DN44209 c0 g1TRINITY DN44209 c0 g1 i14g.32m.32typecomplet
