# Supplementary material for: Venomix: a simple bioinformatic pipeline for identifying and characterizing toxin gene candidates from transcriptomic data
Source: PeerJ. 2018 Jul 31;6:e5361. doi: 10.7717/peerj.5361 (PMC6074769; doi:10.7717/peerj.5361)
Supplement: Supplemental Information 4 [file peerj-06-5361-s004.gz › FinalOutput_E-6/Venom_allergen_5_1/finaltree.pdf]

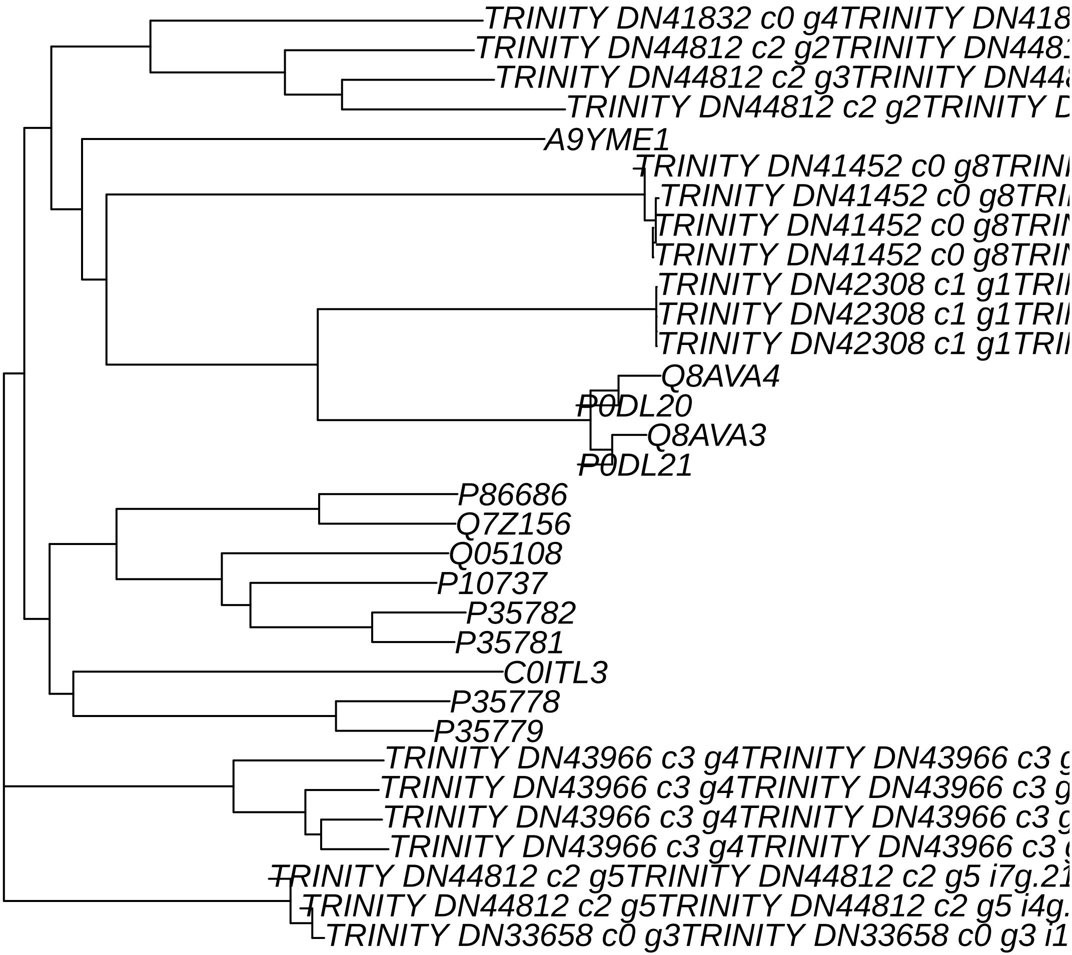
TRINITY DN41832 c0 g4TRINITY DN418
TRINITY DN44812 c2 g2TRINITY DN448
TRINITY DN44812 c2 g3TRINITY DN448
TRINITY DN44812 c2 g2TRINITY D
A9YME1
TRINITY DN41452 c0 g8TRINI
TRINITY DN41452 c0 g8TRI
TRINITY DN41452 c0 g8TRIN
TRINITY DN41452 c0 g8TRIN
TRINITY DN42308 c1 g1TRII
TRINITY DN42308 c1 g1TRII
TRINITY DN42308 c1 g1TRII
Q8AVA4
P0DL20
Q8AVA3
P0DL21
P86686
Q7Z156
Q05108
P10737
P35782
P35781
C0ITL3
P35778
P35779
TRINITY DN43966 c3 g4TRINITY DN43966 c3 g
TRINITY DN43966 c3 g4TRINITY DN43966 c3 g
TRINITY DN43966 c3 g4TRINITY DN43966 c3 g
TRINITY DN43966 c3 g4TRINITY DN43966 c3 g
TRINITY DN44812 c2 g5TRINITY DN44812 c2 g5 i7g.21
TRINITY DN44812 c2 g5TRINITY DN44812 c2 g5 i4g.
TRINITY DN33658 c0 g3TRINITY DN33658 c0 g3 i1
