# Supplementary material for: Venomix: a simple bioinformatic pipeline for identifying and characterizing toxin gene candidates from transcriptomic data
Source: PeerJ. 2018 Jul 31;6:e5361. doi: 10.7717/peerj.5361 (PMC6074769; doi:10.7717/peerj.5361)
Supplement: Supplemental Information 5 [file peerj-06-5361-s005.gz › FinalOutput_GAS_1E-6/Alpha-latroinsectotoxin-Lt1a_2/finaltree.pdf]

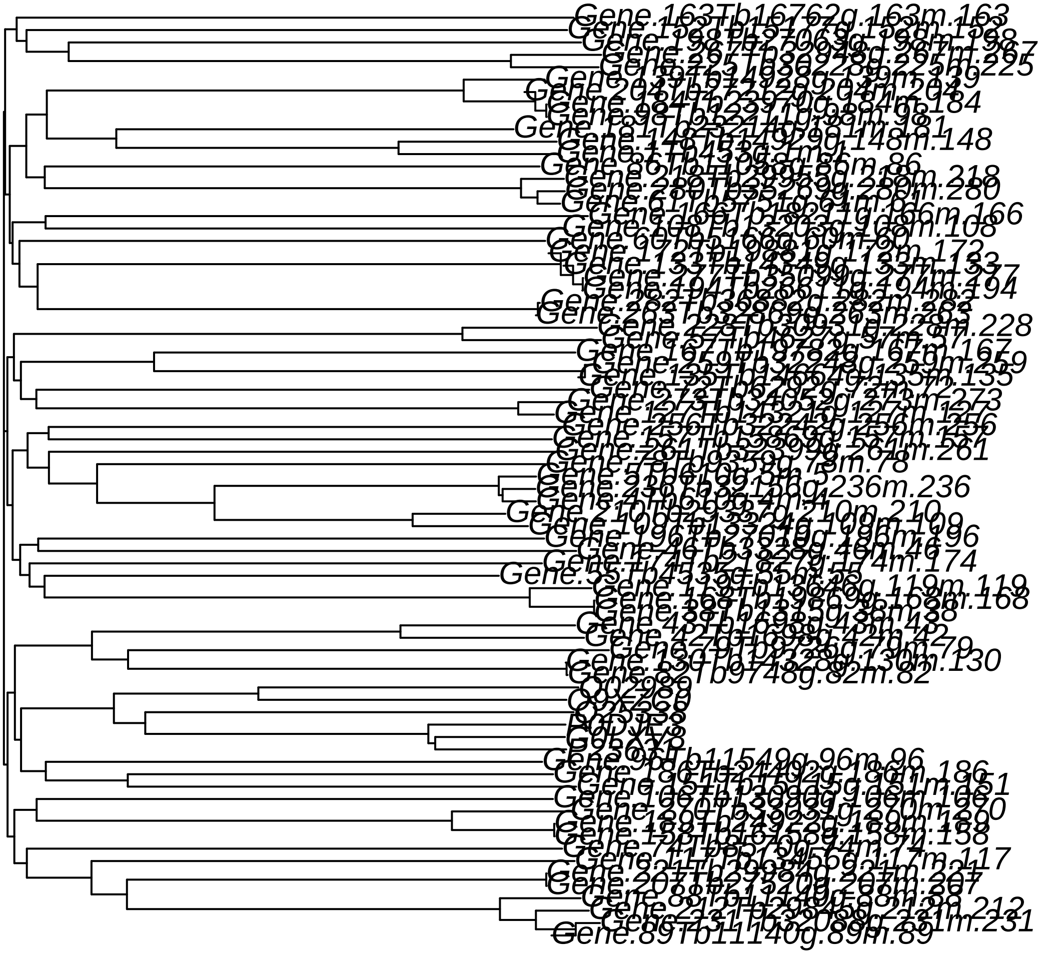
Gene.163Tb16762g.163m.163
Gene.228Tb30931g.228m.228
Gene.236Tb32126g.236m.236
Gene.96Tb11549g.96m.96
Gene.82Tb9748g.82m.82
Gene.89Tb11140g.89m.89
