# Supplementary material for: Venomix: a simple bioinformatic pipeline for identifying and characterizing toxin gene candidates from transcriptomic data
Source: PeerJ. 2018 Jul 31;6:e5361. doi: 10.7717/peerj.5361 (PMC6074769; doi:10.7717/peerj.5361)
Supplement: Supplemental Information 5 [file peerj-06-5361-s005.gz › FinalOutput_GAS_1E-6/Phospholipase_A2_imperatoxin-1_4/finaltree.pdf]

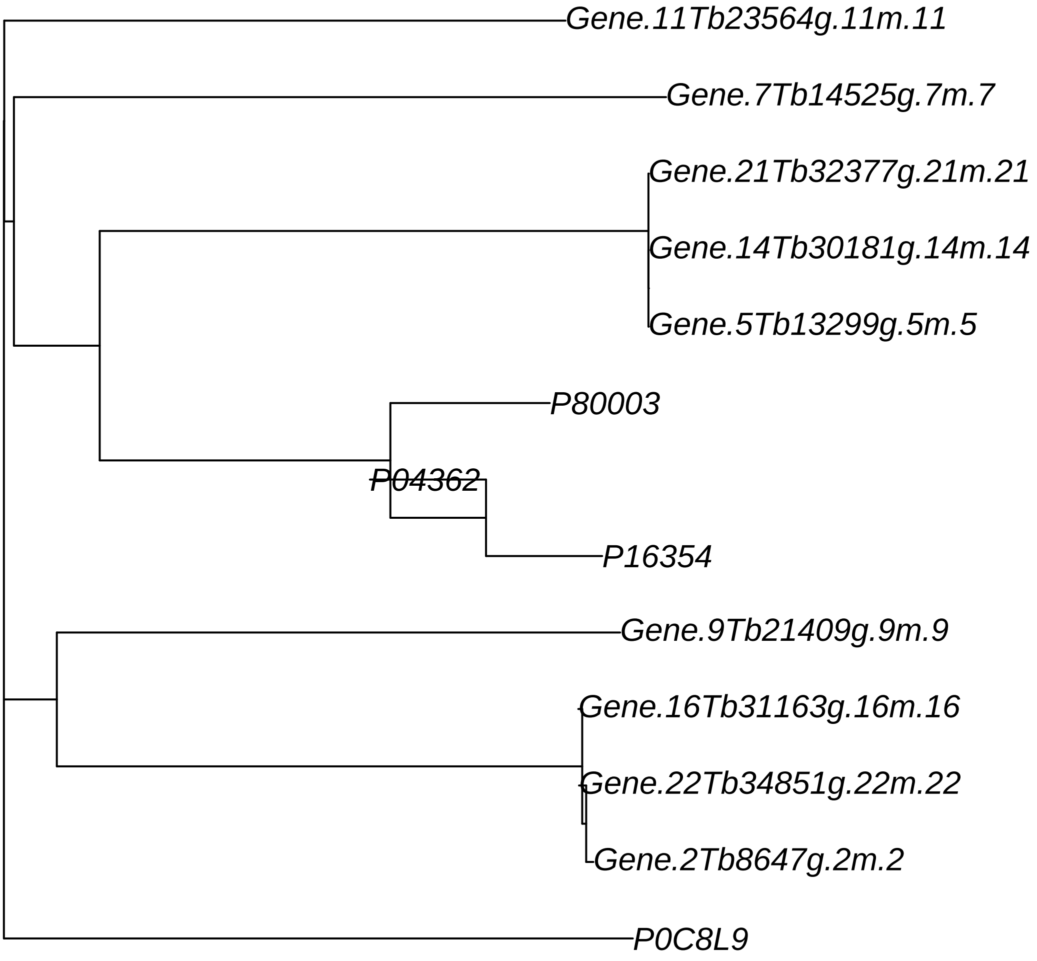
Gene.11Tb23564g.11m.11
Gene.7Tb14525g.7m.7
Gene.21Tb32377g.21m.21
Gene.14Tb30181g.14m.14
Gene.5Tb13299g.5m.5
P80003
P04362
P16354
Gene.9Tb21409g.9m.9
Gene.16Tb31163g.16m.16
Gene.22Tb34851g.22m.22
Gene.2Tb8647g.2m.2
P0C8L9
