# Supplementary material for: Venomix: a simple bioinformatic pipeline for identifying and characterizing toxin gene candidates from transcriptomic data
Source: PeerJ. 2018 Jul 31;6:e5361. doi: 10.7717/peerj.5361 (PMC6074769; doi:10.7717/peerj.5361)
Supplement: Supplemental Information 5 [file peerj-06-5361-s005.gz › FinalOutput_GAS_1E-6/Delta-latroinsectotoxin-Lt1a_1/finaltree.pdf]

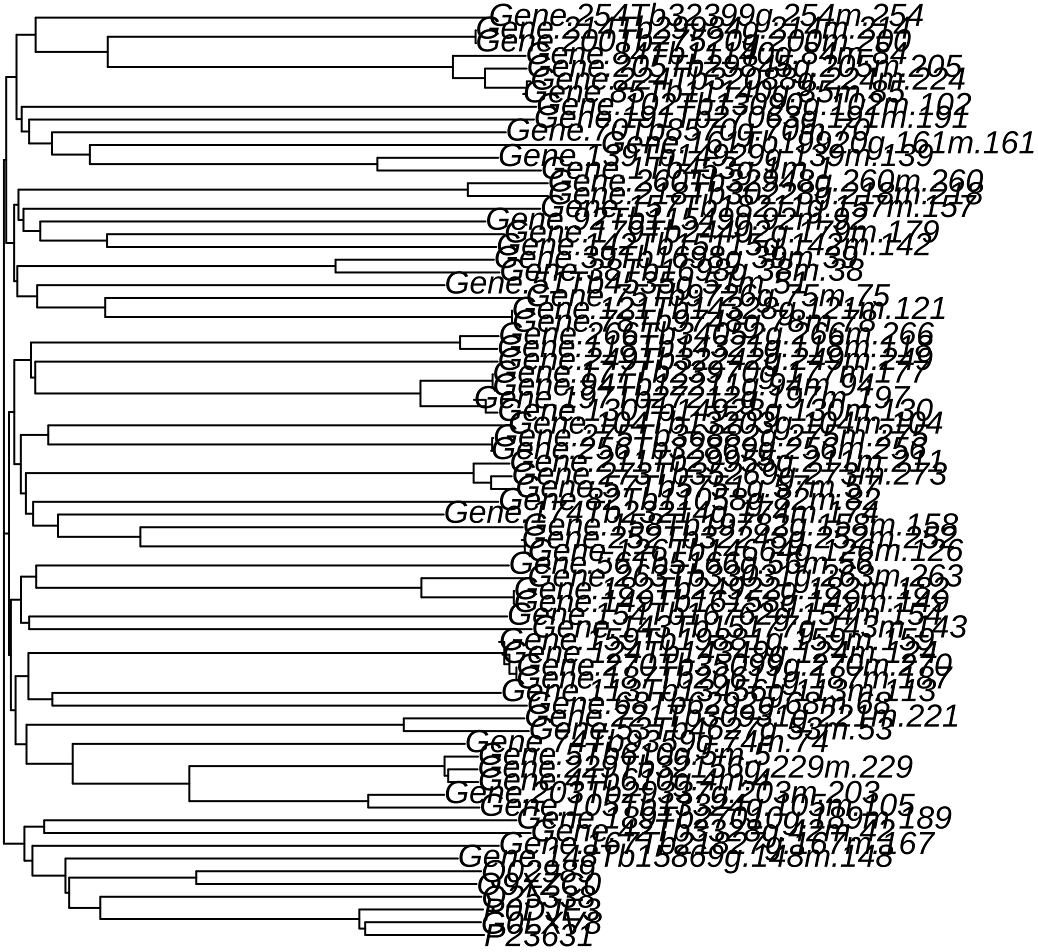
Gene.254Tb32399g.254m.254
Gene.203Tb29337g.203m.203
Gene.148Tb15869g.148m.148
P23631
