# Supplementary material for: Venomix: a simple bioinformatic pipeline for identifying and characterizing toxin gene candidates from transcriptomic data
Source: PeerJ. 2018 Jul 31;6:e5361. doi: 10.7717/peerj.5361 (PMC6074769; doi:10.7717/peerj.5361)
Supplement: Supplemental Information 5 [file peerj-06-5361-s005.gz › FinalOutput_GAS_1E-6/Cysteine-rich_venom_protein_Mr30_2/finaltree.pdf]

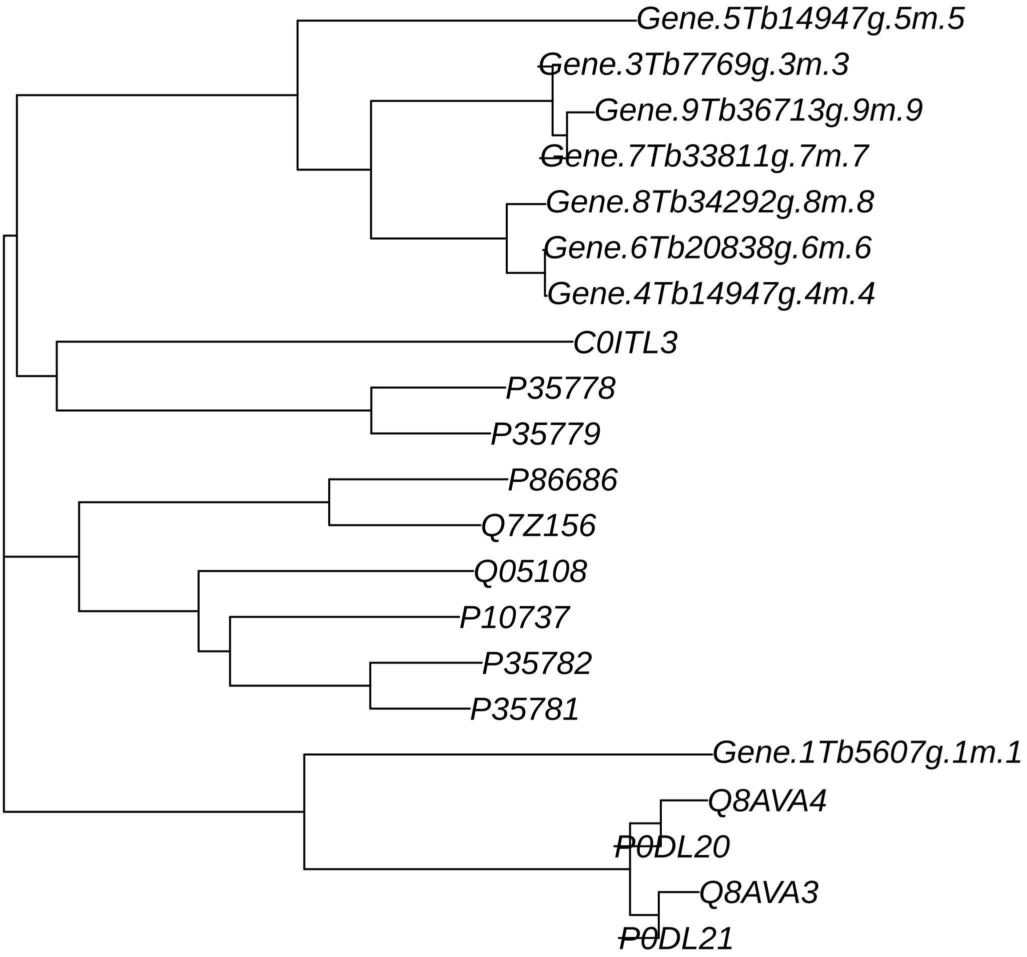
Gene.5Tb14947g.5m.5
Gene.3Tb7769g.3m.3
Gene.9Tb36713g.9m.9
Gene.7Tb33811g.7m.7
Gene.8Tb34292g.8m.8
Gene.6Tb20838g.6m.6
Gene.4Tb14947g.4m.4
C0ITL3
P35778
P35779
P86686
Q7Z156
Q05108
P10737
P35782
P35781
Gene.1Tb5607g.1m.1
Q8AVA4
P0DL20
Q8AVA3
P0DL21
