# Supplementary material for: Venomix: a simple bioinformatic pipeline for identifying and characterizing toxin gene candidates from transcriptomic data
Source: PeerJ. 2018 Jul 31;6:e5361. doi: 10.7717/peerj.5361 (PMC6074769; doi:10.7717/peerj.5361)
Supplement: Supplemental Information 5 [file peerj-06-5361-s005.gz › FinalOutput_GAS_1E-6/Putative_protein-glutamate_O-methyltransferase_1/finaltree.pdf]

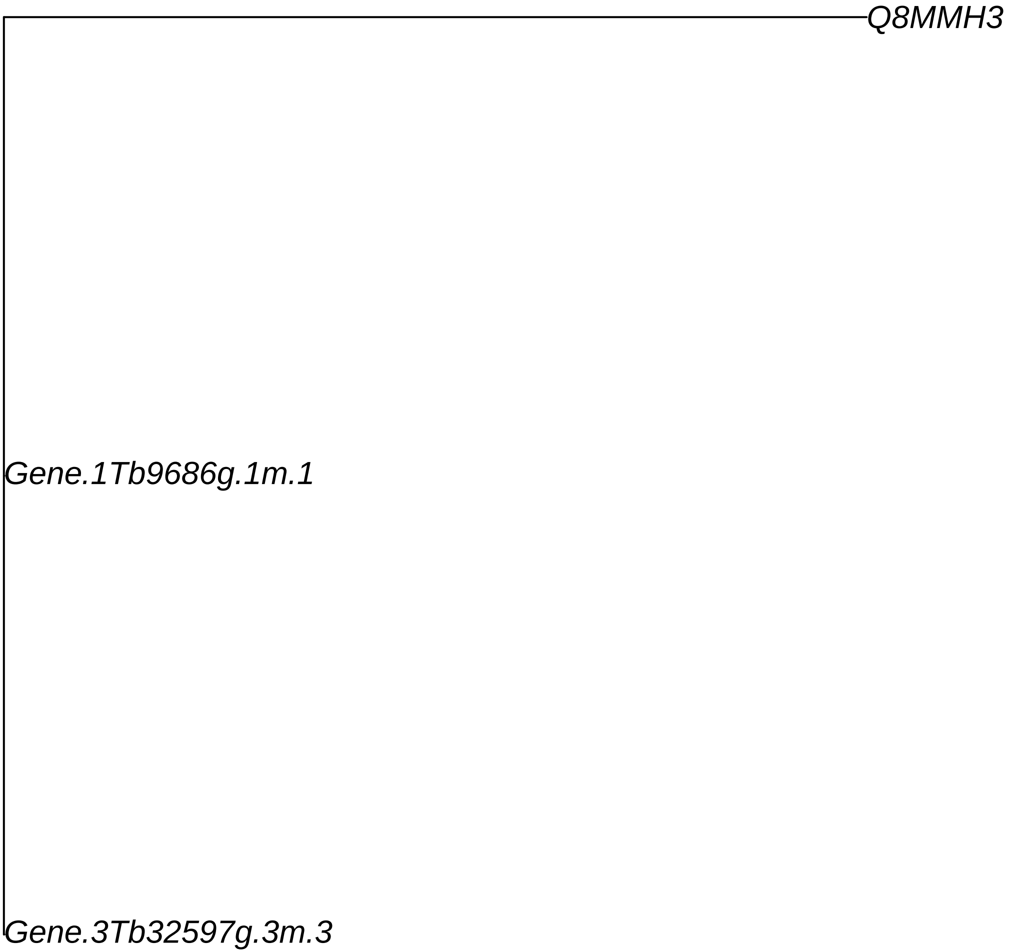
Q8MMH3
Gene.1Tb9686g.1m.1
Gene.3Tb32597g.3m.3
