# Supplementary material for: Venomix: a simple bioinformatic pipeline for identifying and characterizing toxin gene candidates from transcriptomic data
Source: PeerJ. 2018 Jul 31;6:e5361. doi: 10.7717/peerj.5361 (PMC6074769; doi:10.7717/peerj.5361)
Supplement: Supplemental Information 5 [file peerj-06-5361-s005.gz › FinalOutput_GAS_1E-6/Venom_peptide_isomerase_heavy_chain_2/finaltree.pdf]

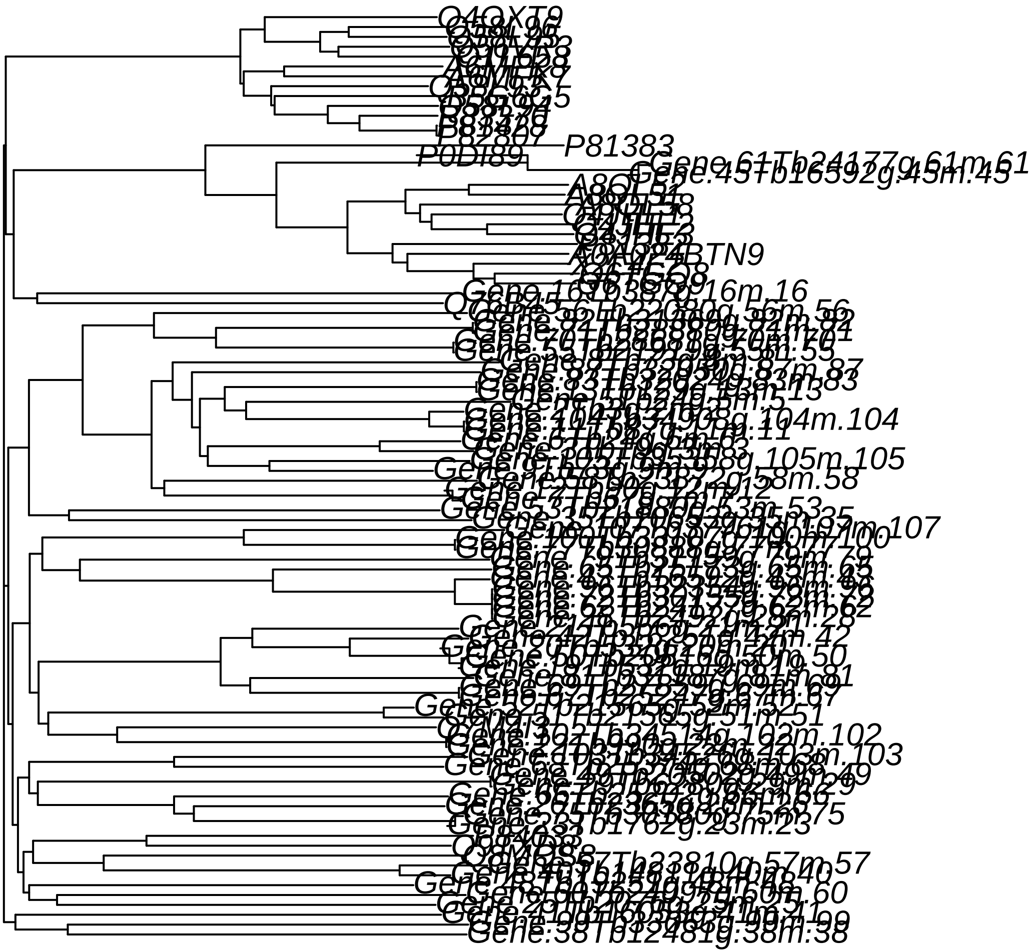

Q4QXT9
P81383
P0DI89
Gene.61Tb24177g.61m.61
Gene.45Tb16592g.45m.45
A0A224BTN9
Gene.104Tb34190g.104m.104
Gene.102Tb34514g.102m.102
Gene.38Tb12481g.38m.38
