# Supplementary material for: Venomix: a simple bioinformatic pipeline for identifying and characterizing toxin gene candidates from transcriptomic data
Source: PeerJ. 2018 Jul 31;6:e5361. doi: 10.7717/peerj.5361 (PMC6074769; doi:10.7717/peerj.5361)
Supplement: Supplemental Information 5 [file peerj-06-5361-s005.gz › FinalOutput_GAS_1E-6/Zinc_metalloproteinase@disintegrin_107/finaltree.pdf]

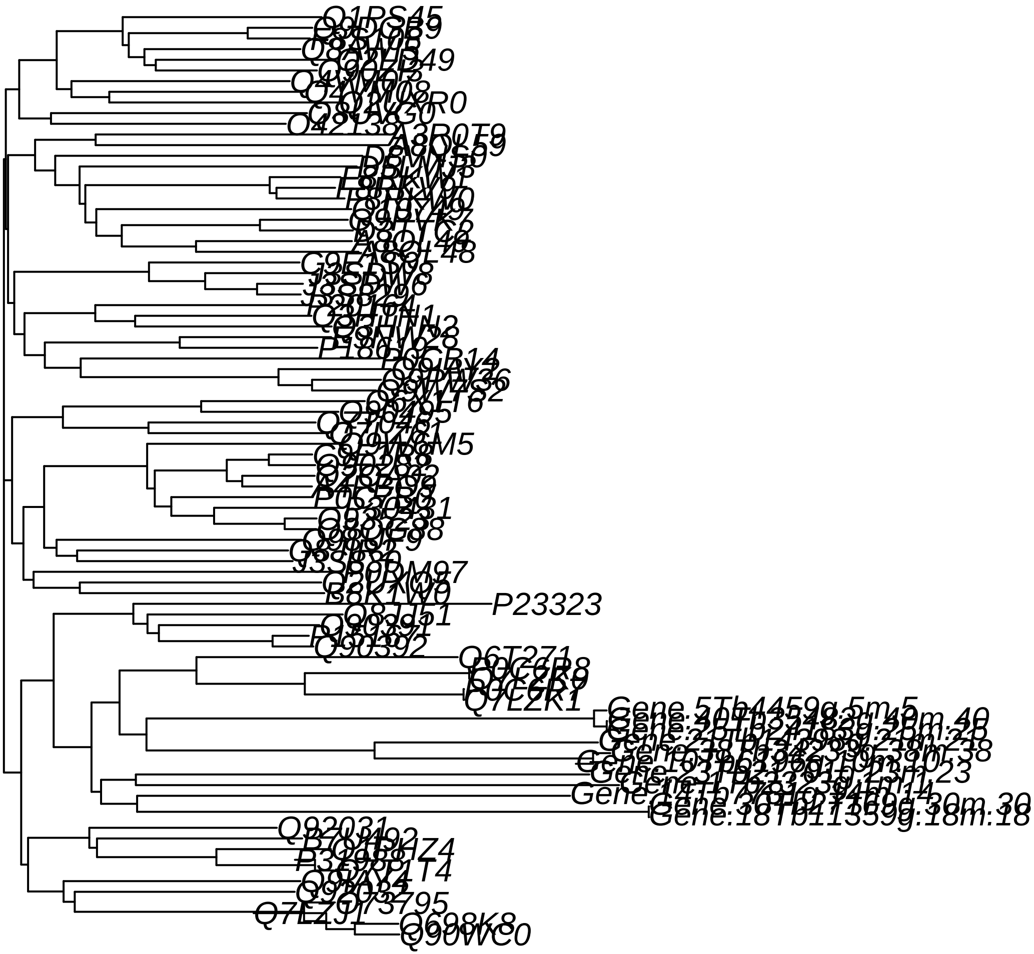
Q1PS45
P23323
Q6T271
Q7LZK1
Gene.5Tb4459g.5m.5
Gene.18Tb11359g.18m.18
Q92031
Q90392
Q7LZJ1
Q698K8
Q90WC0
