# Supplementary material for: Venomix: a simple bioinformatic pipeline for identifying and characterizing toxin gene candidates from transcriptomic data
Source: PeerJ. 2018 Jul 31;6:e5361. doi: 10.7717/peerj.5361 (PMC6074769; doi:10.7717/peerj.5361)
Supplement: Supplemental Information 5 [file peerj-06-5361-s005.gz › FinalOutput_GAS_1E-6/Thrombin-like_enzyme_ancrod_11/finaltree.pdf]

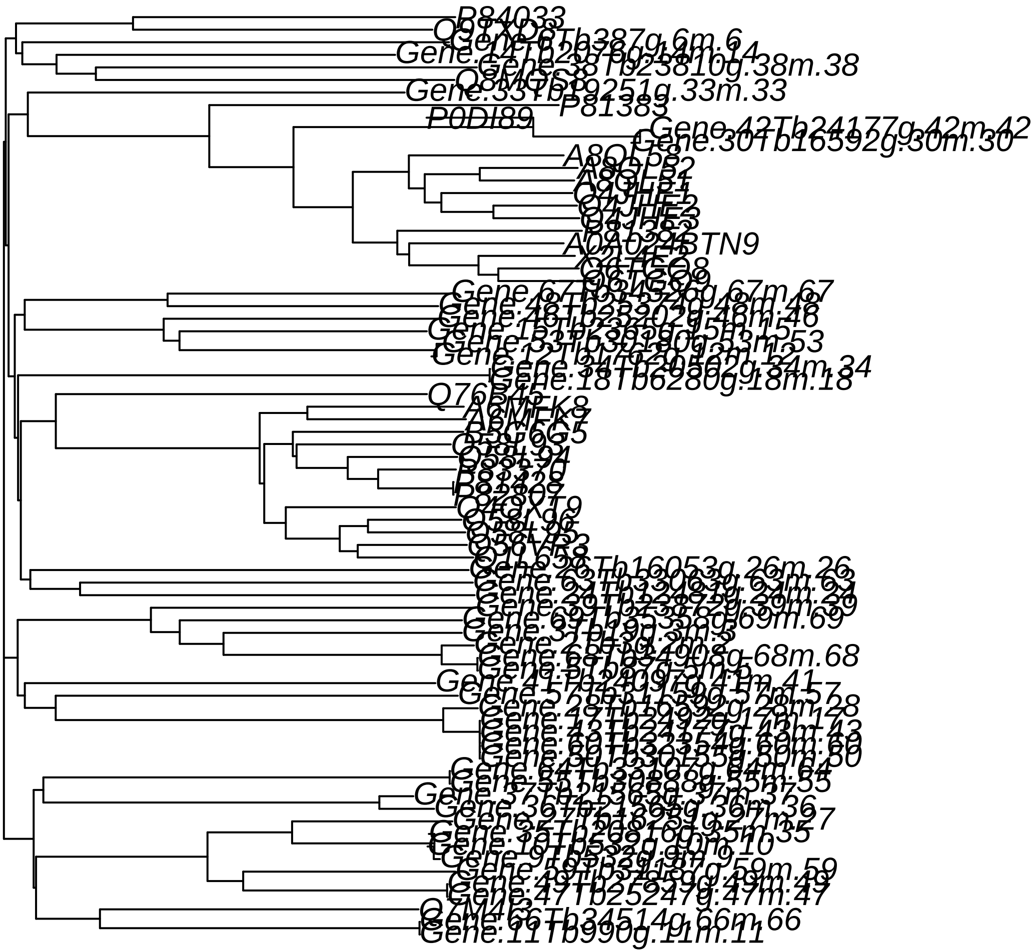
P84033
Gene.38Tb23810g.38m.38
Gene.33Tb19251g.33m.33
P81383
P0DI89
Gene.42Tb24177g.42m.42
Gene.30Tb16592g.30m.30
Gene.18Tb6280g.18m.18
Gene.26Tb16053g.26m.26
Gene.68Tb34908g.68m.68
Gene.59Tb31137g.59m.59
Gene.47Tb25247g.47m.47
Gene.66Tb34514g.66m.66
Gene.11Tb990g.11m.11
