# Supplementary material for: Venomix: a simple bioinformatic pipeline for identifying and characterizing toxin gene candidates from transcriptomic data
Source: PeerJ. 2018 Jul 31;6:e5361. doi: 10.7717/peerj.5361 (PMC6074769; doi:10.7717/peerj.5361)
Supplement: Supplemental Information 5 [file peerj-06-5361-s005.gz › FinalOutput_GAS_1E-6/Zinc_metalloproteinase_recombinant_fibrinogenase_II_1/finaltree.pdf]

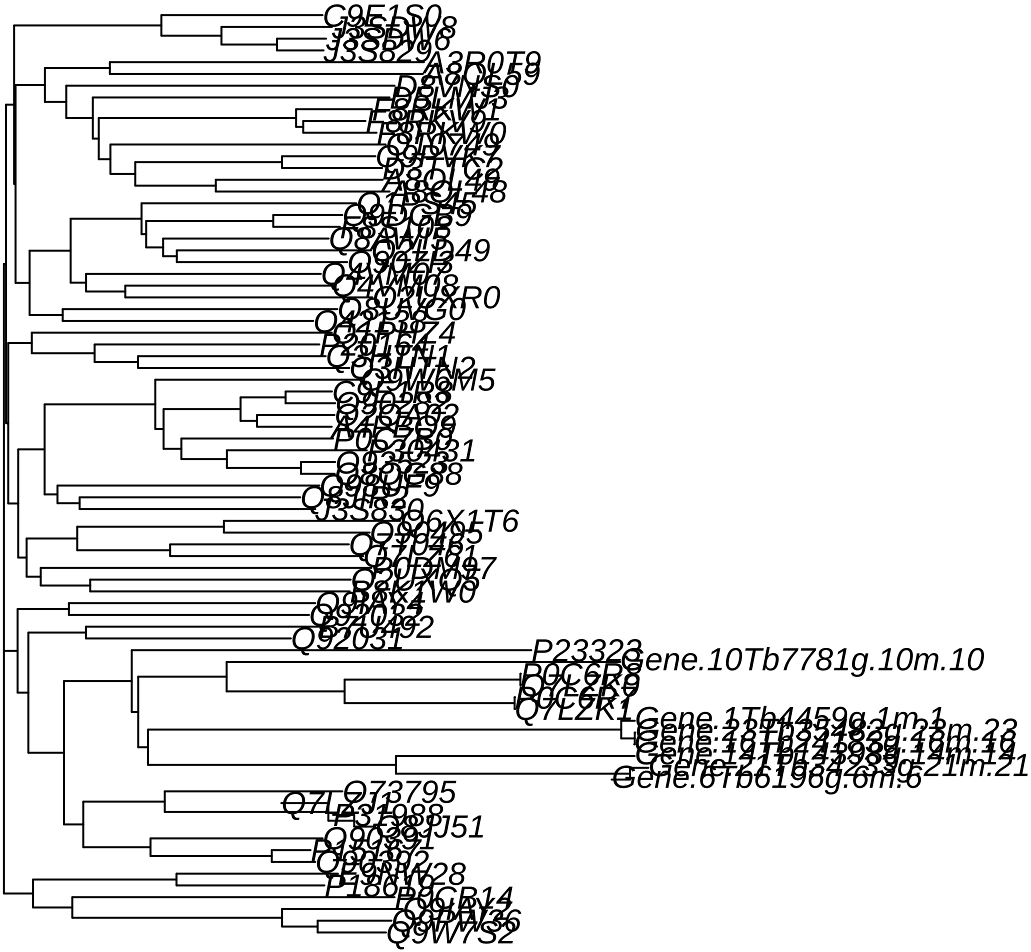
C9F1S0
J3S829
A3R0T9
P23323
Gene.10Tb7781g.10m.10
Q7LZK1
Gene.1Tb4459g.1m.1
Gene.6Tb6196g.6m.6
Q73795
Q92031
Q9W7S2
