# Supplementary material for: Venomix: a simple bioinformatic pipeline for identifying and characterizing toxin gene candidates from transcriptomic data
Source: PeerJ. 2018 Jul 31;6:e5361. doi: 10.7717/peerj.5361 (PMC6074769; doi:10.7717/peerj.5361)
Supplement: Supplemental Information 5 [file peerj-06-5361-s005.gz › FinalOutput_GAS_1E-6/Serine_proteinase-like_BMK-CBP_1/finaltree.pdf]

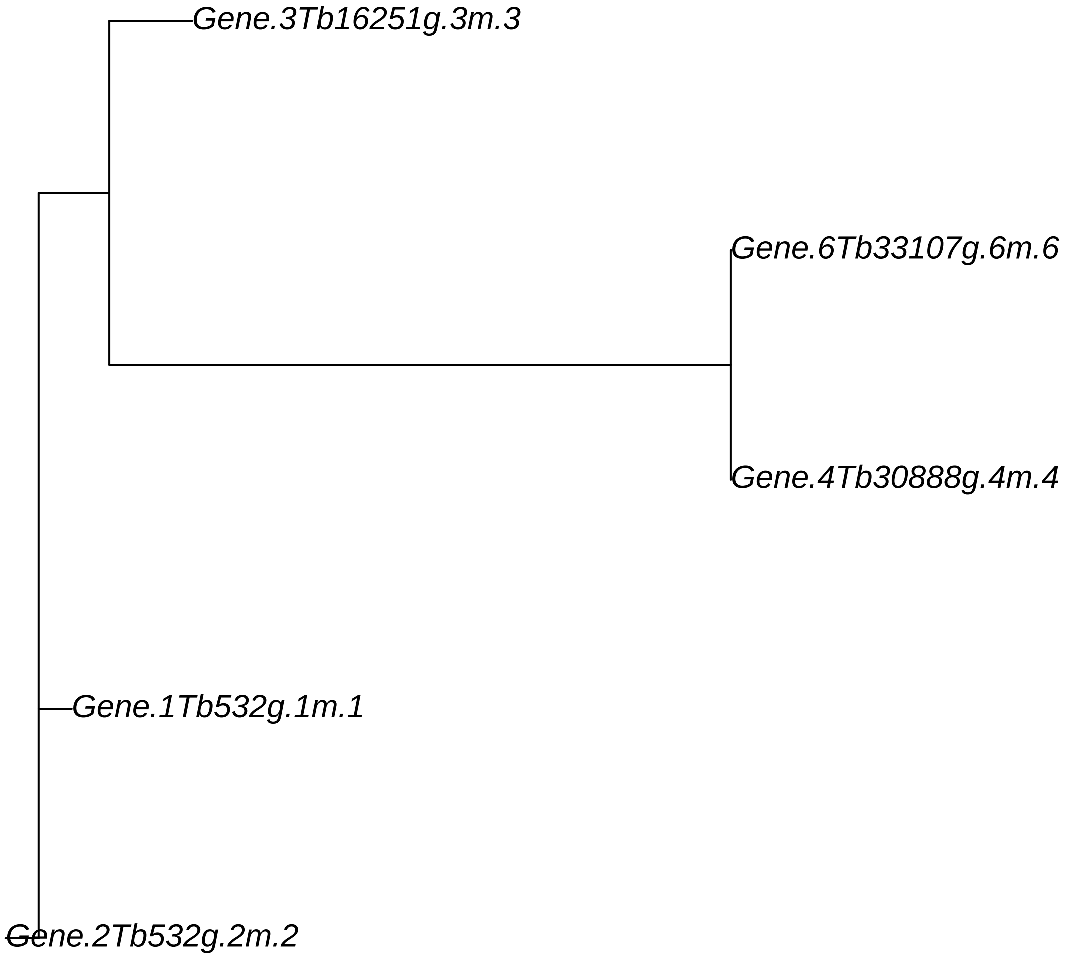

Gene.3Tb16251g.3m.3
Gene.6Tb33107g.6m.6
Gene.4Tb30888g.4m.4
Gene.1Tb532g.1m.1
Gene.2Tb532g.2m.2
