# Supplementary material for: Venomix: a simple bioinformatic pipeline for identifying and characterizing toxin gene candidates from transcriptomic data
Source: PeerJ. 2018 Jul 31;6:e5361. doi: 10.7717/peerj.5361 (PMC6074769; doi:10.7717/peerj.5361)
Supplement: Supplemental Information 5 [file peerj-06-5361-s005.gz › FinalOutput_GAS_1E-6/Venom_prothrombin_activator_pseutarin-C_catalytic_subunit_12/finaltree.pdf]

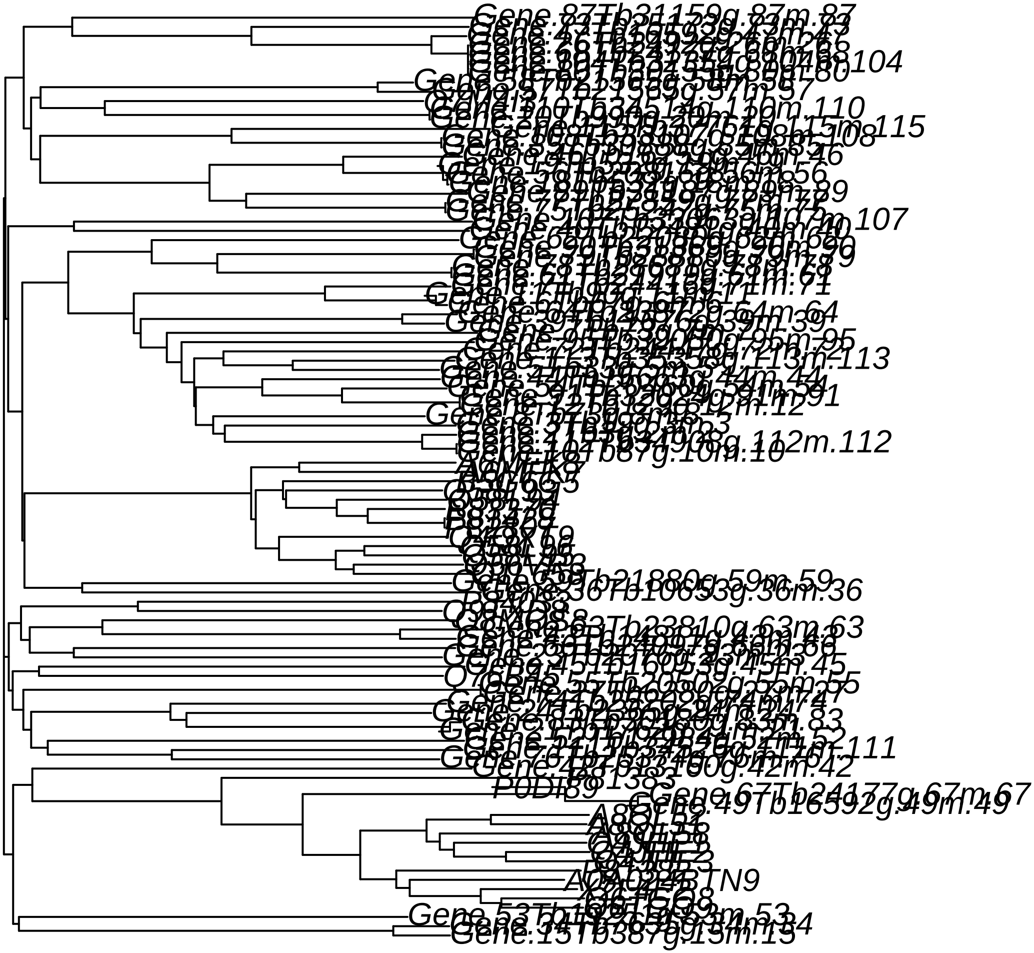
Gene.87Tb31159g.87m.87
Gene.59Tb21880g.59m.59
Gene.36Tb10653g.36m.36
Gene.63Tb23810g.63m.63
P0DI89
Gene.67Tb24177g.67m.67
Gene.49Tb16592g.49m.49
Gene.15Tb387g.15m.15
