# Supplementary material for: Venomix: a simple bioinformatic pipeline for identifying and characterizing toxin gene candidates from transcriptomic data
Source: PeerJ. 2018 Jul 31;6:e5361. doi: 10.7717/peerj.5361 (PMC6074769; doi:10.7717/peerj.5361)
Supplement: Supplemental Information 5 [file peerj-06-5361-s005.gz › FinalOutput_GAS_1E-6/Kunitz-type_serine_protease_inhibitor_homolog_delta-dendrotoxin_2/finaltree.pdf]

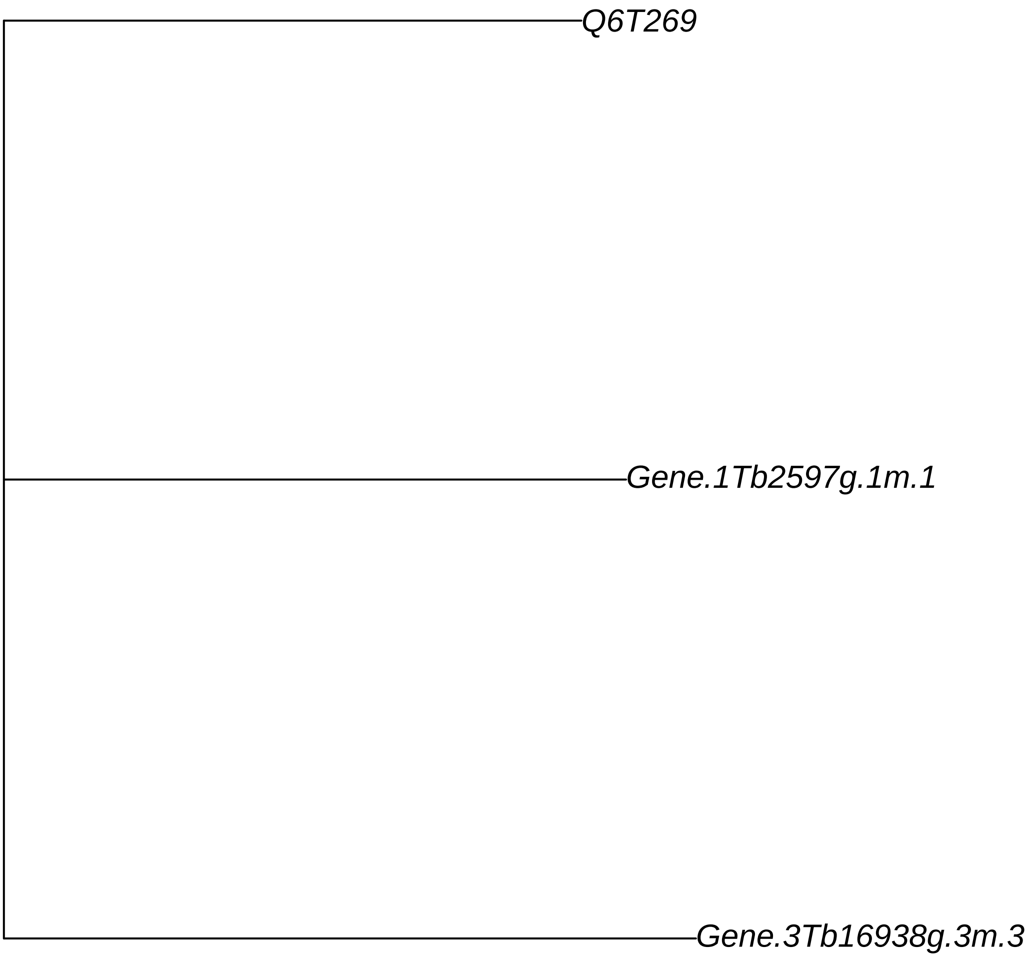
Q6T269
Gene.1Tb2597g.1m.1
Gene.3Tb16938g.3m.3
