# Supplementary material for: Venomix: a simple bioinformatic pipeline for identifying and characterizing toxin gene candidates from transcriptomic data
Source: PeerJ. 2018 Jul 31;6:e5361. doi: 10.7717/peerj.5361 (PMC6074769; doi:10.7717/peerj.5361)
Supplement: Supplemental Information 5 [file peerj-06-5361-s005.gz › FinalOutput_GAS_1E-6/Kunitz-type_serine_protease_inhibitor_Hg1_1/finaltree.pdf]

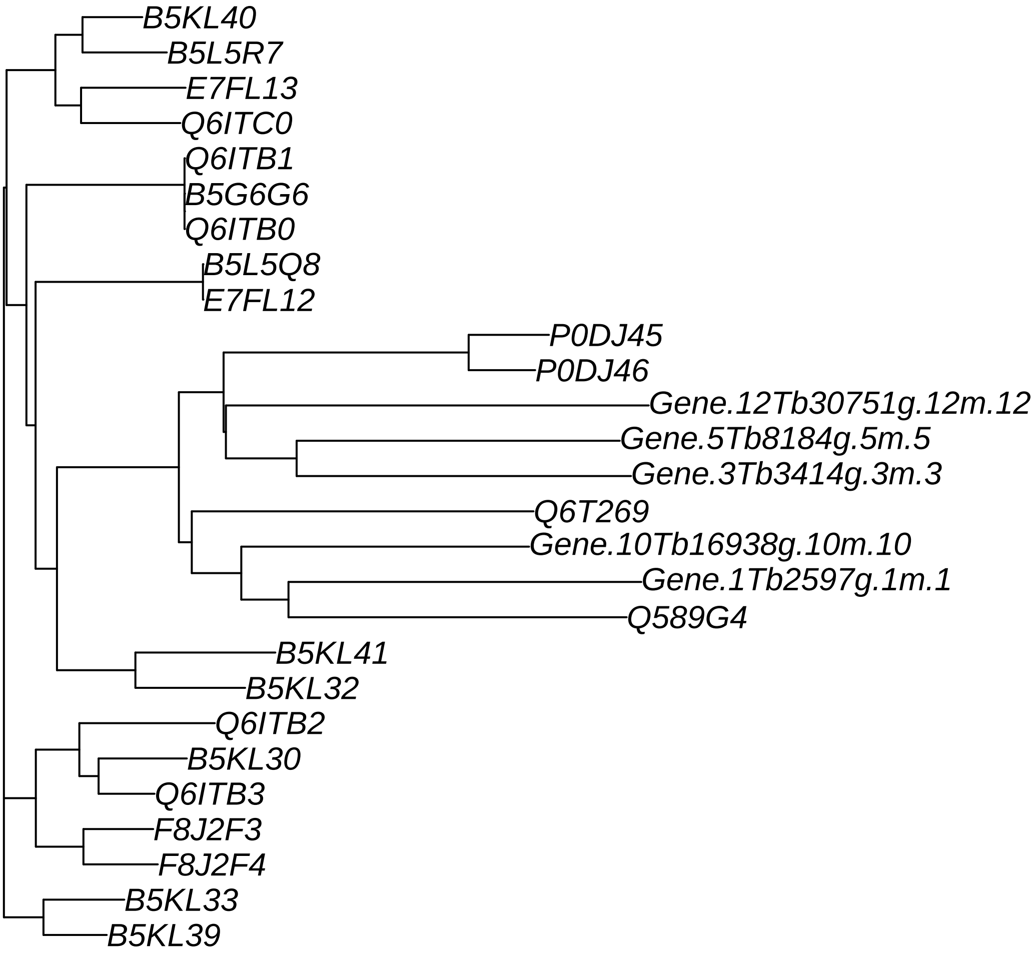
B5KL40
B5L5R7
E7FL13
Q6ITC0
Q6ITB1
B5G6G6
Q6ITB0
B5L5Q8
E7FL12
P0DJ45
P0DJ46
Gene.12Tb30751g.12m.12
Gene.5Tb8184g.5m.5
Gene.3Tb3414g.3m.3
Q6T269
Gene.10Tb16938g.10m.10
Gene.1Tb2597g.1m.1
Q589G4
B5KL41
B5KL32
Q6ITB2
B5KL30
Q6ITB3
F8J2F3
F8J2F4
B5KL33
B5KL39
