# Supplementary material for: Venomix: a simple bioinformatic pipeline for identifying and characterizing toxin gene candidates from transcriptomic data
Source: PeerJ. 2018 Jul 31;6:e5361. doi: 10.7717/peerj.5361 (PMC6074769; doi:10.7717/peerj.5361)
Supplement: Supplemental Information 5 [file peerj-06-5361-s005.gz › FinalOutput_GAS_1E-6/Venom_allergen_3_30/finaltree.pdf]

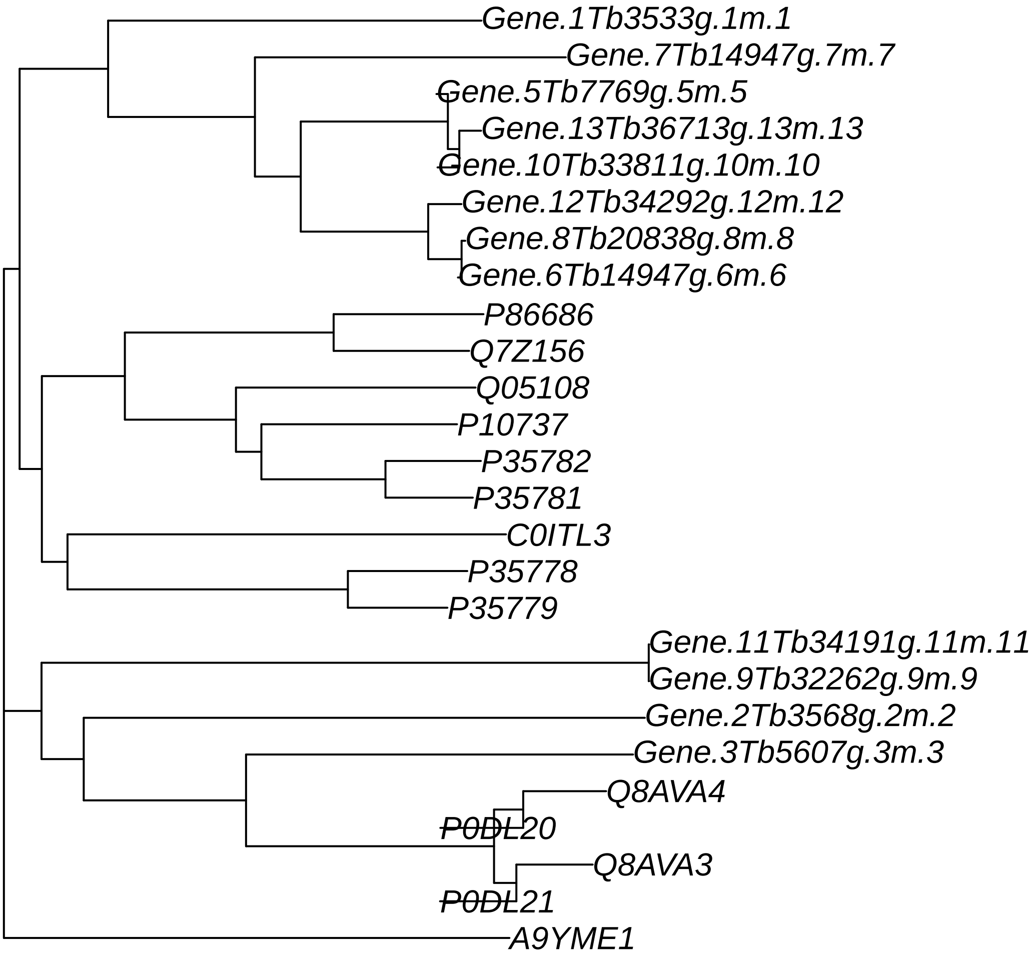

Gene.1Tb3533g.1m.1
Gene.7Tb14947g.7m.7
Gene.5Tb7769g.5m.5
Gene.13Tb36713g.13m.13
Gene.10Tb33811g.10m.10
Gene.12Tb34292g.12m.12
Gene.8Tb20838g.8m.8
Gene.6Tb14947g.6m.6
P86686
Q7Z156
Q05108
P10737
P35782
P35781
C0ITL3
P35778
P35779
Gene.11Tb34191g.11m.11
Gene.9Tb32262g.9m.9
Gene.2Tb3568g.2m.2
Gene.3Tb5607g.3m.3
Q8AVA4
P0DL20
Q8AVA3
P0DL21
A9YME1
