# Supplementary material for: Venomix: a simple bioinformatic pipeline for identifying and characterizing toxin gene candidates from transcriptomic data
Source: PeerJ. 2018 Jul 31;6:e5361. doi: 10.7717/peerj.5361 (PMC6074769; doi:10.7717/peerj.5361)
Supplement: Supplemental Information 5 [file peerj-06-5361-s005.gz › FinalOutput_GAS_1E-6/Acetylcholinesterase-1_1/finaltree.pdf]

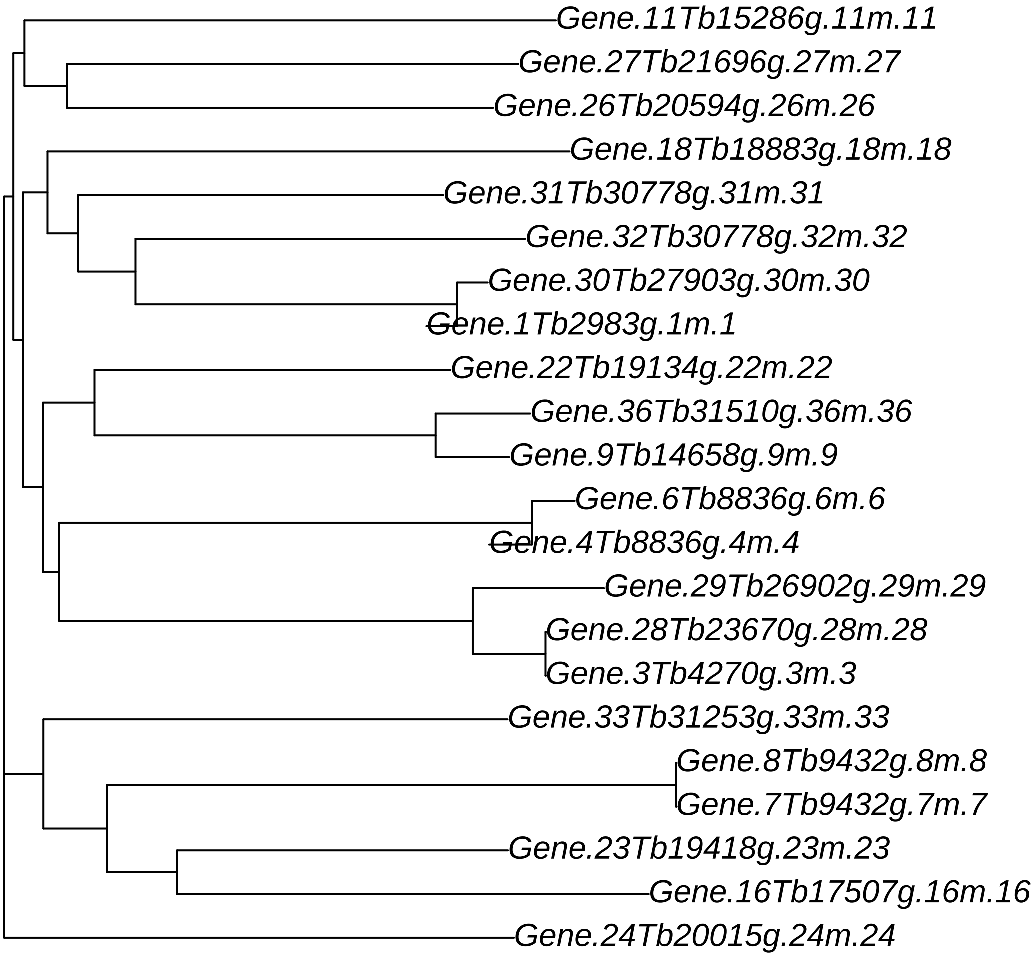
Gene.11Tb15286g.11m.11
Gene.27Tb21696g.27m.27
Gene.26Tb20594g.26m.26
Gene.18Tb18883g.18m.18
Gene.31Tb30778g.31m.31
Gene.32Tb30778g.32m.32
Gene.30Tb27903g.30m.30
Gene.1Tb2983g.1m.1
Gene.22Tb19134g.22m.22
Gene.36Tb31510g.36m.36
Gene.9Tb14658g.9m.9
Gene.6Tb8836g.6m.6
Gene.4Tb8836g.4m.4
Gene.29Tb26902g.29m.29
Gene.28Tb23670g.28m.28
Gene.3Tb4270g.3m.3
Gene.33Tb31253g.33m.33
Gene.8Tb9432g.8m.8
Gene.7Tb9432g.7m.7
Gene.23Tb19418g.23m.23
Gene.16Tb17507g.16m.16
Gene.24Tb20015g.24m.24
