# Supplementary material for: Venomix: a simple bioinformatic pipeline for identifying and characterizing toxin gene candidates from transcriptomic data
Source: PeerJ. 2018 Jul 31;6:e5361. doi: 10.7717/peerj.5361 (PMC6074769; doi:10.7717/peerj.5361)
Supplement: Supplemental Information 5 [file peerj-06-5361-s005.gz › FinalOutput_GAS_1E-6/Neprilysin-1_1/finaltree.pdf]

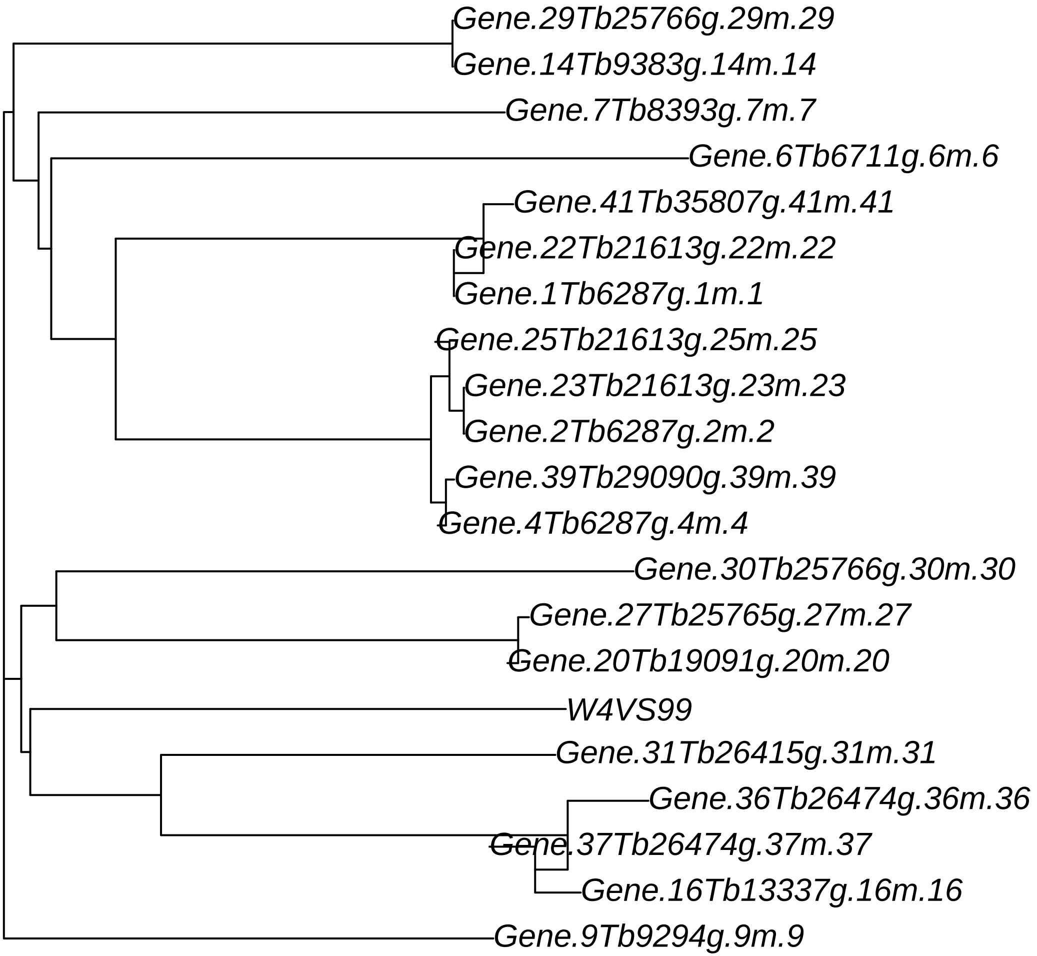
Gene.29Tb25766g.29m.29
Gene.14Tb9383g.14m.14
Gene.7Tb8393g.7m.7
Gene.6Tb6711g.6m.6
Gene.41Tb35807g.41m.41
Gene.22Tb21613g.22m.22
Gene.1Tb6287g.1m.1
Gene.25Tb21613g.25m.25
Gene.23Tb21613g.23m.23
Gene.2Tb6287g.2m.2
Gene.39Tb29090g.39m.39
Gene.4Tb6287g.4m.4
Gene.30Tb25766g.30m.30
Gene.27Tb25765g.27m.27
Gene.20Tb19091g.20m.20
W4VS99
Gene.31Tb26415g.31m.31
Gene.36Tb26474g.36m.36
Gene.37Tb26474g.37m.37
Gene.16Tb13337g.16m.16
Gene.9Tb9294g.9m.9
