# Supplementary material for: Venomix: a simple bioinformatic pipeline for identifying and characterizing toxin gene candidates from transcriptomic data
Source: PeerJ. 2018 Jul 31;6:e5361. doi: 10.7717/peerj.5361 (PMC6074769; doi:10.7717/peerj.5361)
Supplement: Supplemental Information 5 [file peerj-06-5361-s005.gz › FinalOutput_GAS_1E-6/Peroxiredoxin-4_1/finaltree.pdf]

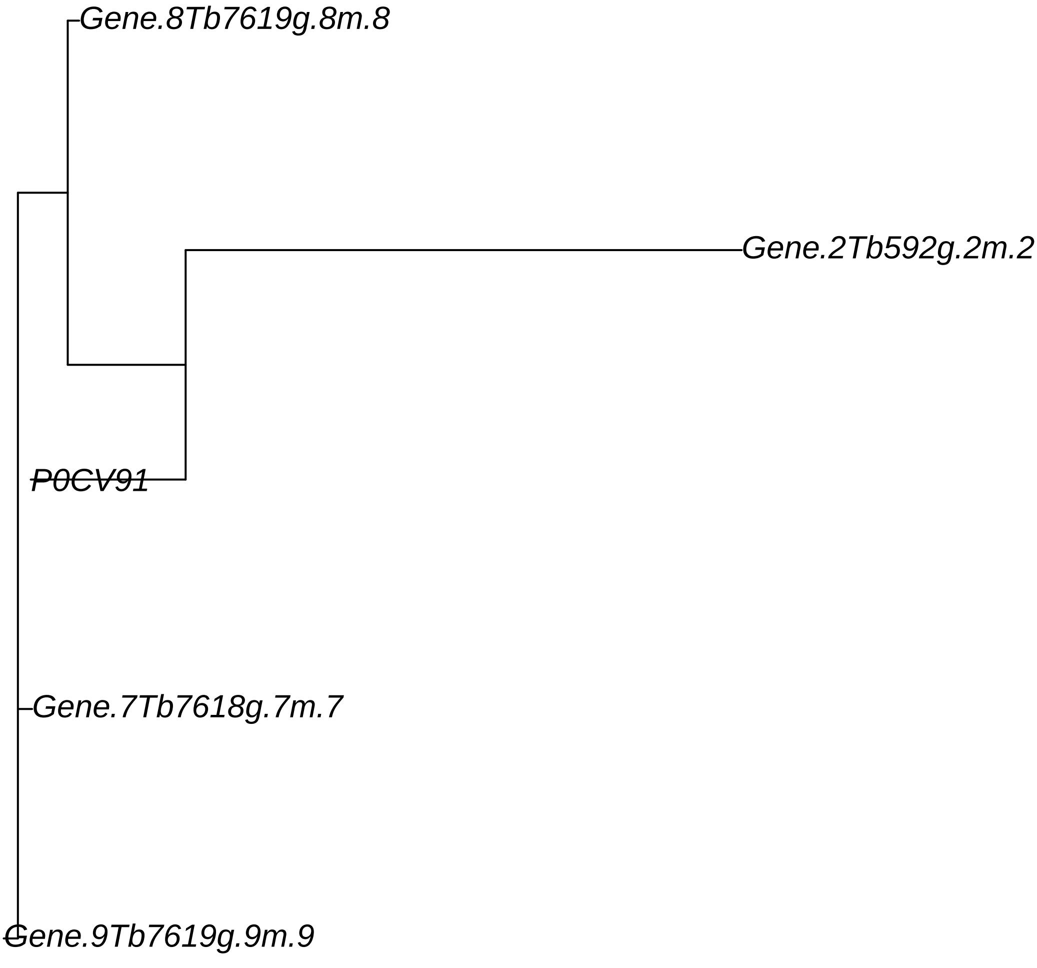

Gene.8Tb7619g.8m.8
Gene.2Tb592g.2m.2
P0CV91
Gene.7Tb7618g.7m.7
Gene.9Tb7619g.9m.9
