# Supplementary material for: Venomix: a simple bioinformatic pipeline for identifying and characterizing toxin gene candidates from transcriptomic data
Source: PeerJ. 2018 Jul 31;6:e5361. doi: 10.7717/peerj.5361 (PMC6074769; doi:10.7717/peerj.5361)
Supplement: Supplemental Information 5 [file peerj-06-5361-s005.gz › FinalOutput_GAS_1E-6/Venom_allergen_5_1/finaltree.pdf]

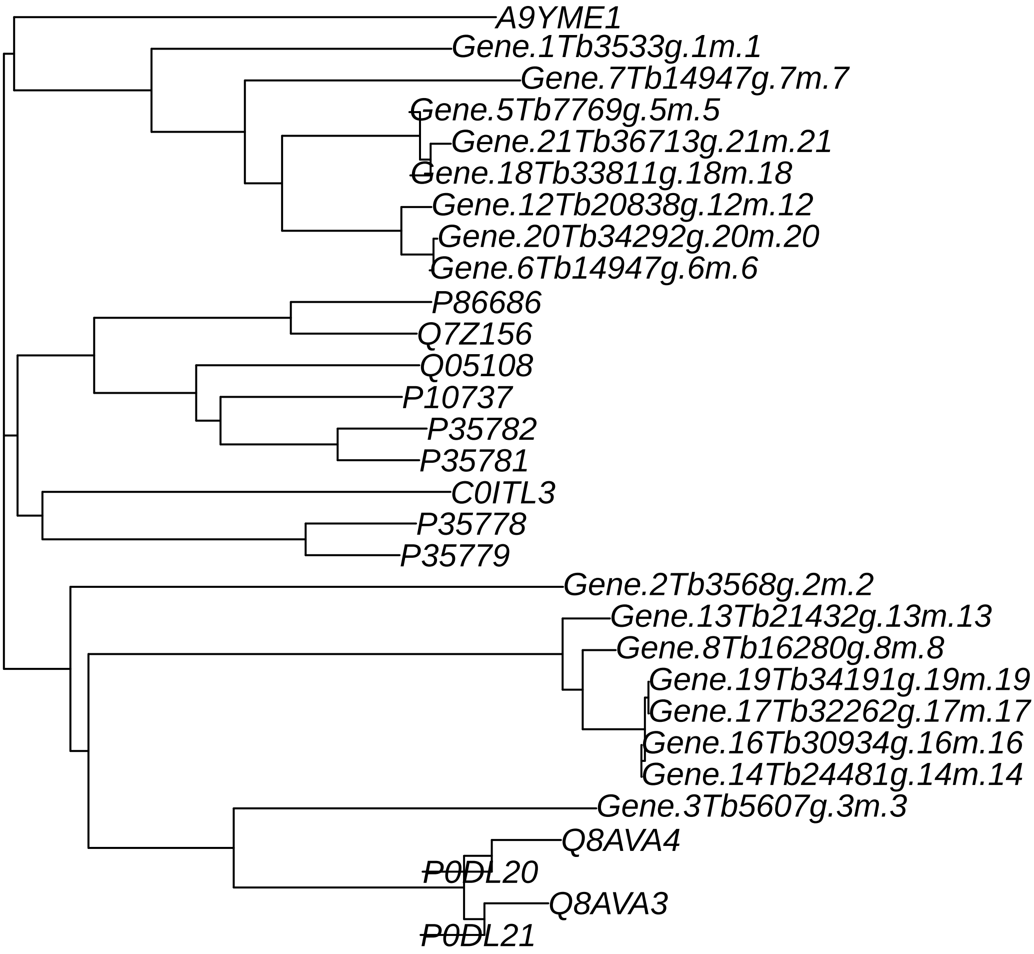
A9YME1
Gene.1Tb3533g.1m.1
Gene.7Tb14947g.7m.7
Gene.5Tb7769g.5m.5
Gene.21Tb36713g.21m.21
Gene.18Tb33811g.18m.18
Gene.12Tb20838g.12m.12
Gene.20Tb34292g.20m.20
Gene.6Tb14947g.6m.6
P86686
Q7Z156
Q05108
P10737
P35782
P35781
C0ITL3
P35778
P35779
Gene.2Tb3568g.2m.2
Gene.13Tb21432g.13m.13
Gene.8Tb16280g.8m.8
Gene.19Tb34191g.19m.19
Gene.17Tb32262g.17m.17
Gene.16Tb30934g.16m.16
Gene.14Tb24481g.14m.14
Gene.3Tb5607g.3m.3
Q8AVA4
P0DL20
Q8AVA3
P0DL21
