# Supplementary material for: Venomix: a simple bioinformatic pipeline for identifying and characterizing toxin gene candidates from transcriptomic data
Source: PeerJ. 2018 Jul 31;6:e5361. doi: 10.7717/peerj.5361 (PMC6074769; doi:10.7717/peerj.5361)
Supplement: Supplemental Information 5 [file peerj-06-5361-s005.gz › FinalOutput_GAS_1E-6/Techylectin-like_protein_1/finaltree.pdf]

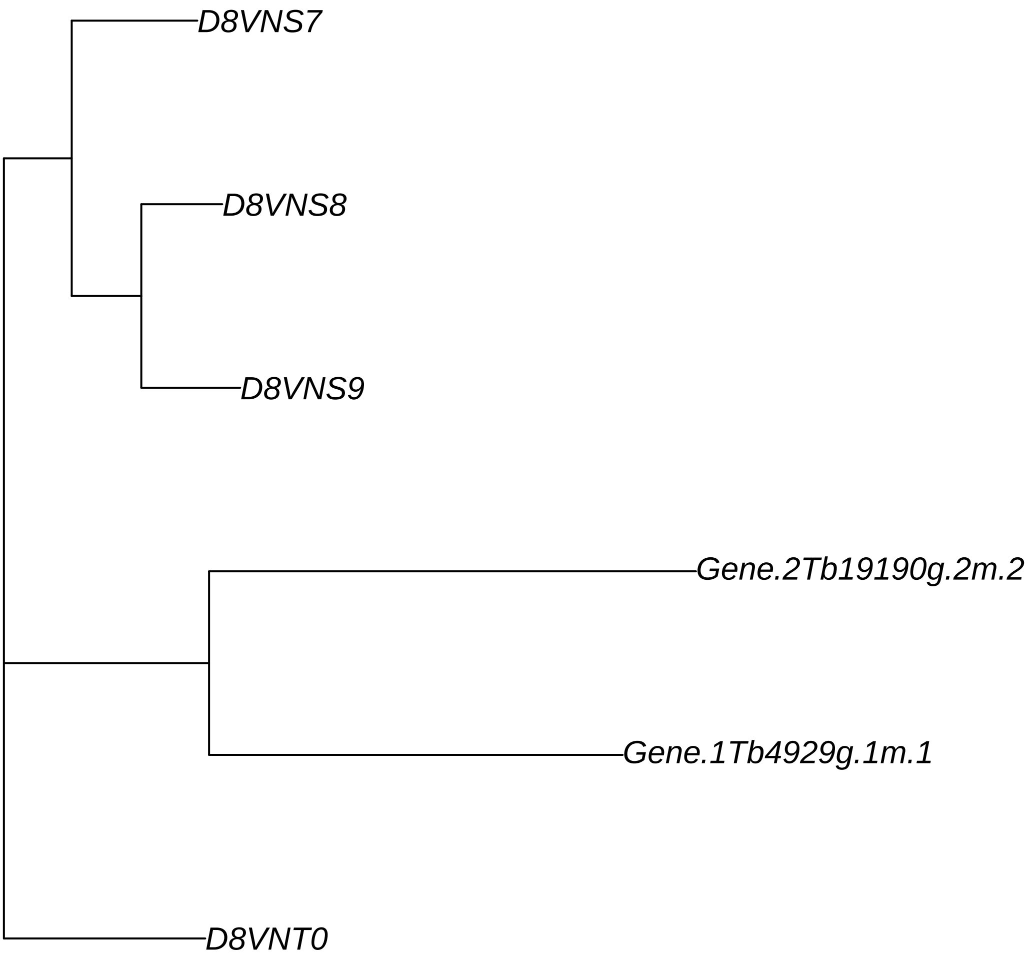
D8VNS7
D8VNS8
D8VNS9
Gene.2Tb19190g.2m.2
Gene.1Tb4929g.1m.1
D8VNT0
